# Performance of large language models in ambiguous treatment scenarios of early breast cancer

**DOI:** 10.1016/j.breast.2026.104932

**Published:** 2026-09-23

**Authors:** Anton Oseledchyk, Walter Paul Weber, Benjamin Kasenda

**Affiliations:** a Medical Oncology, University Hospital Basel, Basel, Switzerland; b Breast Cancer Center, University Hospital Basel, Basel, Switzerland

**Keywords:** St.Gallen international breast cancer conference, SGBCC, Artificial intelligence, Large language models, Early breast cancer

## Abstract

**Background:**

Consensus conferences guide treatment where evidence is ambiguous; lacking ground truth, artificial intelligence is unvalidated there. We evaluated concordance between large language models (LLMs) and expert votes at the 2025 St. Gallen International Breast Cancer Conference.

**Materials and methods:**

Four LLMs (Claude Opus 4.1, GPT-5, Gemini Flash 2.5, DeepSeek V3.2) each answered 81 multiple-choice questions once (324 responses). Outcomes were first-choice agreement (LLM selected the panel's modal option) and top-2 agreement (either of its two leading options; 66 questions with ≥3 options). Normalized consensus strength scored panel agreement 0–100, adjusted for option count. Mixed-effects logistic regression with random intercepts for question modelled agreement on consensus strength, complexity and LLM (Model 1: 324 observations/81 questions; Model 2: 264/66). Agreement was also compared with an option-count-weighted null (permutation, question-level cluster bootstrap). Questions with ≤2 LLMs matching the panel underwent qualitative review.

**Results:**

First-choice agreement was 54–64% and top-2 agreement 77–89%. Agreement exceeded chance overall (58.6% vs 29.8%; +28.9 points, 95% CI 21.4–36.4) and for questions with 3, 4 and ≥ 5 options (42.6–66.1% vs 18.8–33.3% expected), but not binary questions (65.0% vs 50.0%; +15.0, 95% CI −5.0 to 33.3). Consensus strength was the predominant factor associated with concordance (Model 1: odds ratio 1.04/point, 95% CI 1.02–1.06, P < 0.001; Model 2: 1.06, 1.03–1.09, P < 0.001). Qualitative review identified knowledge gaps in 18 questions (22%), defensible alternatives in 14 (17%) and evidence-consistent insights in 8 (10%).

**Conclusions:**

LLMs amplify prevailing evidence but show significant limitations in ambiguous scenarios. Prospective evaluation within consensus processes is needed.

## Highlights

• Large language models answered 81 questions from 2025 St. Gallen consensus.
• LLM–expert first-choice agreement was 54–64%; top-2 agreement 77–89%.
• Expert consensus strength was the main factor associated with agreement.
• Agreement exceeded chance for multi-option but not for binary questions.
• LLMs amplify prevailing evidence rather than reason independently.

## Graphical abstract

**Figure:**
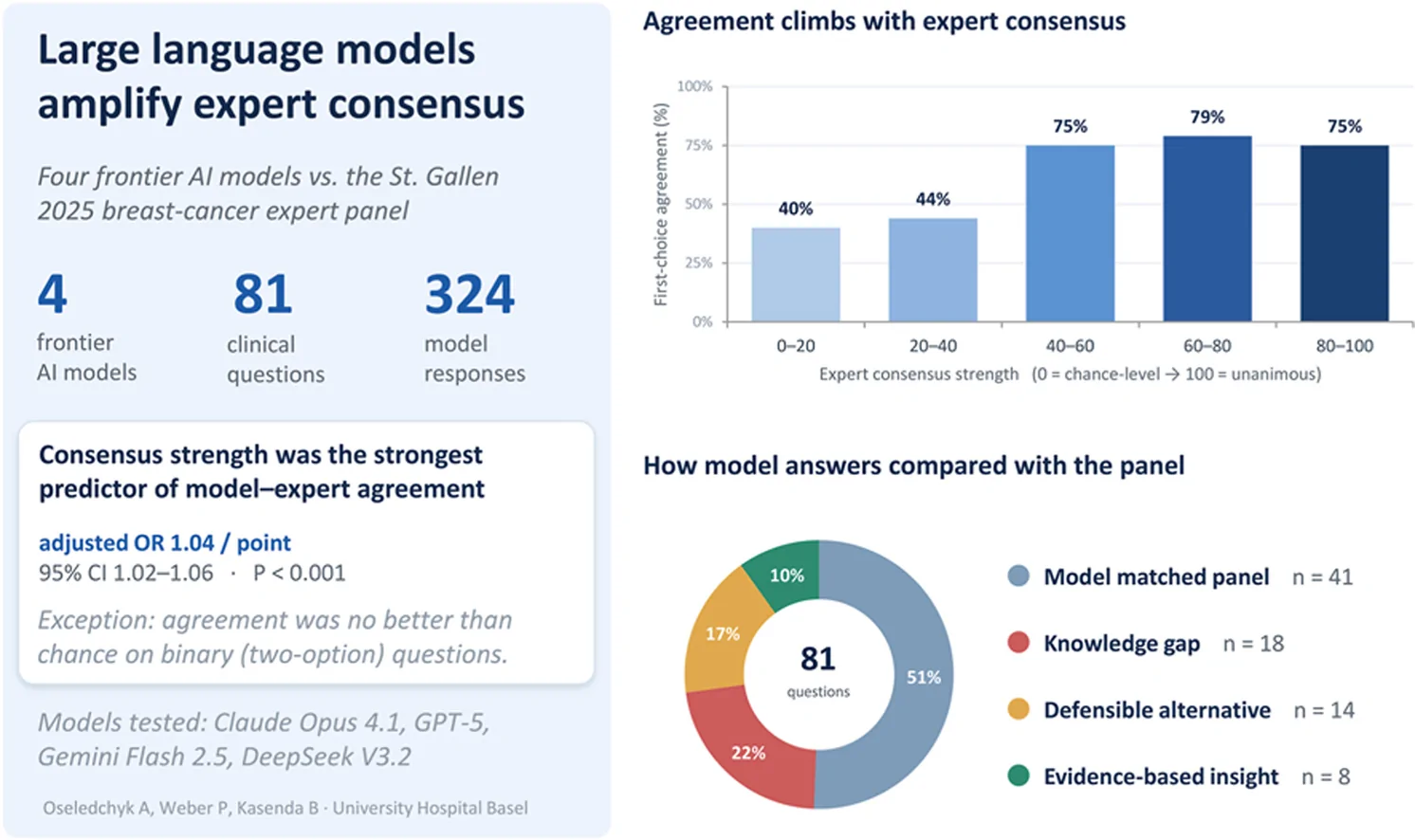

## 1 Introduction

Breast cancer has long served as a testing ground for innovations in oncology. Tamoxifen became the first small-molecule treatment that specifically targeted a molecular driver - the estrogen receptor [1,2]. A few decades later, identification of the HER2 oncogene allowed trastuzumab to emerge as the first monoclonal antibody that selectively targets an oncoprotein in a solid tumor [3] and ado-trastuzumab emtansine (T-DM1) the first antibody-drug conjugate [4]. Breast cancer research was the first to establish neoadjuvant therapy for high-risk disease [5] as standard of care and established pathologic complete response as a key prognostic endpoint [6]. Currently, breast cancer remains the world's most studied cancer maintaining the highest volume of ongoing clinical trials globally [[7], [8], [9]]. This wealth of data presents an increasing challenge for clinical decision-making. The sheer volume and heterogeneity of trial results make evidence synthesis increasingly difficult. In recognition of these challenges, the St. Gallen International Breast Cancer Conference brings together leading breast cancer specialists to vote on treatment recommendations in areas where evidence is ambiguous or conflicting, to provide guidance to the global clinical community [10,11].

Although consensus conferences seek balanced recommendations, they remain subject to human biases. These biases are anchored in personal clinical experiences of individual experts which shape their attitudes toward treatment-related toxicity, risk tolerance and their assessment of patients' capacity to endure treatments [12,13]. Additionally, pharmaceutical marketing and industry relationships may subtly influence expert opinions, even among well-intentioned specialists committed to evidence-based practice [14]. These cognitive biases and external influences can lead to recommendations that reflect the collective predispositions of the panel as much as objective synthesis of available evidence. The integration of artificial intelligence (AI) and large language models (LLM) into expert deliberations may offer a potential mechanism to counterbalance these human biases.

AI has the potential to provide expert panels with a comprehensive, transparent data layer that systematically identifies inconsistencies in trial data and synthesizes evidence-based treatment options [15,16]. However, the integration of AI-driven decision-making is not without risk; critical challenges persist, notably the potential for LLMs to generate plausible but medically incorrect information (“hallucination”) [17,18], limited ability to contextualize findings within real-world clinical constraints, and the challenge of validating AI-generated recommendations [19,20]. There is currently minimal empirical evidence examining whether LLMs can accurately replicate expert consensus in complex clinical scenarios or identifying the specific clinical contexts where agreement or disagreement occurs. To evaluate LLMs performance in complex clinical scenarios, where no single ground truth exists, we analyzed agreement between LLM outputs and expert panel recommendations from the St. Gallen International Breast Cancer Conference 2025 [10,11].

## 2 Methods

### 2.1 Study design

We conducted a cross-sectional analysis evaluating the concordance between large language model (LLM) recommendations and expert votes from the St. Gallen International Breast Cancer Conference 2025 (March 2025, Vienna, Austria). We selected 81 clinical questions spanning six domains: hormone receptor-positive disease (n = 33), axillary management (n = 15), triple-negative breast cancer (n = 10), follow-up care (n = 9), radiation therapy (n = 9), and HER2-positive disease (n = 5). We excluded questions on ductal carcinoma in situ or prophylactic measures in healthy women at risk. The expert panel comprised 70 internationally recognized breast cancer specialists from a range of disciplines and regions [10,11]; between 61 and 69 experts voted on each question by structured electronic polling with given multiple choice answer options.

We evaluated four frontier LLMs: Claude Opus 4.1 (Anthropic, San Francisco, CA, USA; queried August 31, 2025), GPT-5 Thinking with Deep Research (OpenAI, San Francisco, CA, USA; queried September 3, 2025), Gemini Flash 2.5 with Deep Research (Google DeepMind, London, UK; queried September 3, 2025), and DeepSeekV3.2 with DeepThink and Search (DeepSeek AI, Hangzhou, China; queried September 9, 2025). Paid versions (except DeepSeek) were accessed through standard web interfaces using default settings without custom optimization. Each LLM received identical structured prompts instructing them to function as expert physicians synthesizing pre-trained knowledge and current online literature, with evidence restricted to publications through February 28, 2025, explicitly excluding St. Gallen 2025 proceedings. Prompts requested an answer choice, an evidence synthesis, key references, guideline alignment, subgroup considerations, a confidence level, and documentation of web searches performed; complete prompts are in Supplementary Methods. Each query was performed once without iterative refinement.

### 2.2 Outcome measures

We defined two levels of LLM-expert agreement: first-choice agreement, (LLM selected the modal expert panel response, most frequently chosen option) and top-2 agreement (LLM selection matched either of the two most popular expert choices; calculated only for questions with ≥3 answer options). To investigate consensus of the expert panel across questions with varying number of multiple choice answer options, we employed normalized consensus strength using: [(P_max - P_random)/(1 - P_random)] × 100, where: P_max = the proportion of experts selecting the modal response (the most frequently chosen answer) and P_random = 1/k, where k = the number of answer options. This yielded a 0-100 scale where 0 indicates consensus at chance level, 100 indicates unanimity. Questions were grouped by complexity: binary (2 options), simple (3 options), moderate (4 options), and complex (≥5 options).

### 2.3 Statistical analysis

We calculated rates of LLM-expert panel agreement stratified by LLM, clinical domain, and question complexity. To identify factors associated with LLM-expert agreement while accounting for clustered data structure (multiple LLM responses per question), we employed mixed-effects logistic regression with random intercepts for question identification. Model 1 examined first-choice agreement (n = 324: 4 LLMs × 81 questions) with fixed effects for normalized consensus strength (continuous, 0-100), question complexity (binary/simple/moderate/complex) and LLM source (with ChatGPT as reference). Model 2 examined top-2 agreement excluding binary questions (n = 264: 4 LLMs × 66 questions) with identical fixed effects. Results are reported as odds ratios (OR) with 95% confidence intervals (CI). Model assumptions were assessed by variance inflation factors, DHARMa simulated residuals and quantile-quantile plots (all adequate), and robustness by parametric bootstrap resampling. To determine whether agreement exceeded that expected from uninformed selection, we specified an option-count-weighted null model in which, for a question with k options, the chance probability of matching the panel's modal choice is 1/k (2/k for top-2 agreement). Observed agreement was compared with this expectation by Monte-Carlo permutation, with significance assessed by a question-level cluster bootstrap accounting for the four responses nested within each question. We additionally audited the structured web-search documentation and reference fields of all 324 responses for any reference to the 2025 conference or to sources post-dating the evidence cutoff. All analyses used R version 4.3.1 [21]; packages, versions and full diagnostics are provided in the Supplementary Material.

### 2.4 Reporting

This study followed STROBE guidelines for cross-sectional studies [22]. All tests were two-sided with α = 0.05; given the exploratory nature of this investigation, we did not apply corrections for multiple comparisons. The study analyzed publicly available conference voting data and did not involve human subjects; formal ethics approval was not required. Complete datasets, analysis code and prompts are available in the online Supplementary Materials.

### 2.5 Qualitative analysis

All 81 questions were divided into two groups: “AI consensus” in which three or four LLMs selected the expert 1st choice answer, and “no AI consensus” in which two or fewer LLMs matched the expert first choice. All panel votes, LLM selections, and rationales are provided in an interactive table in the online Supplementary Materials. “No AI consensus” questions were reviewed by a dedicated breast cancer medical oncologist (A.O.). Answer options, rationale and references that were provide by the LLMs were evaluated by the investigator and categorized in 3 groups: as negative divergence (inferior reasoning or knowledge gap), neutral divergence (ambiguous or no impact), or positive divergence (insightful reasoning).

### 2.6 LLM-based editorial assistance

LLMs (Claude Opus 4.1, GPT-5) were used as writing aids for clarity and grammar and as programming aids for statistical code, but not to generate concepts, interpret results or create novel content [23]; the authors retain full responsibility for the final text.

## 3 Results

### 3.1 Quantitative analysis of LLM performance

LLM-expert panel agreement varied across clinical domains (Fig. 1A and B). For first-choice overall agreement ranged from 54% to 64%. Agreement rates across clinical domains and LLMs ranged from 20% to 89%, with the follow-up domain showing the highest pooled agreement (77.8%) and the hormone receptor-positive domain the lowest (49.2%) (Fig. 1A); domain estimates warrant caution: the smallest held five questions. When considering top-2 agreement (excluding binary question types), overall agreement rates increased to 77% to 89% and ranging between 40% and 100% across domains (Fig. 1B).

**Fig. 1:**
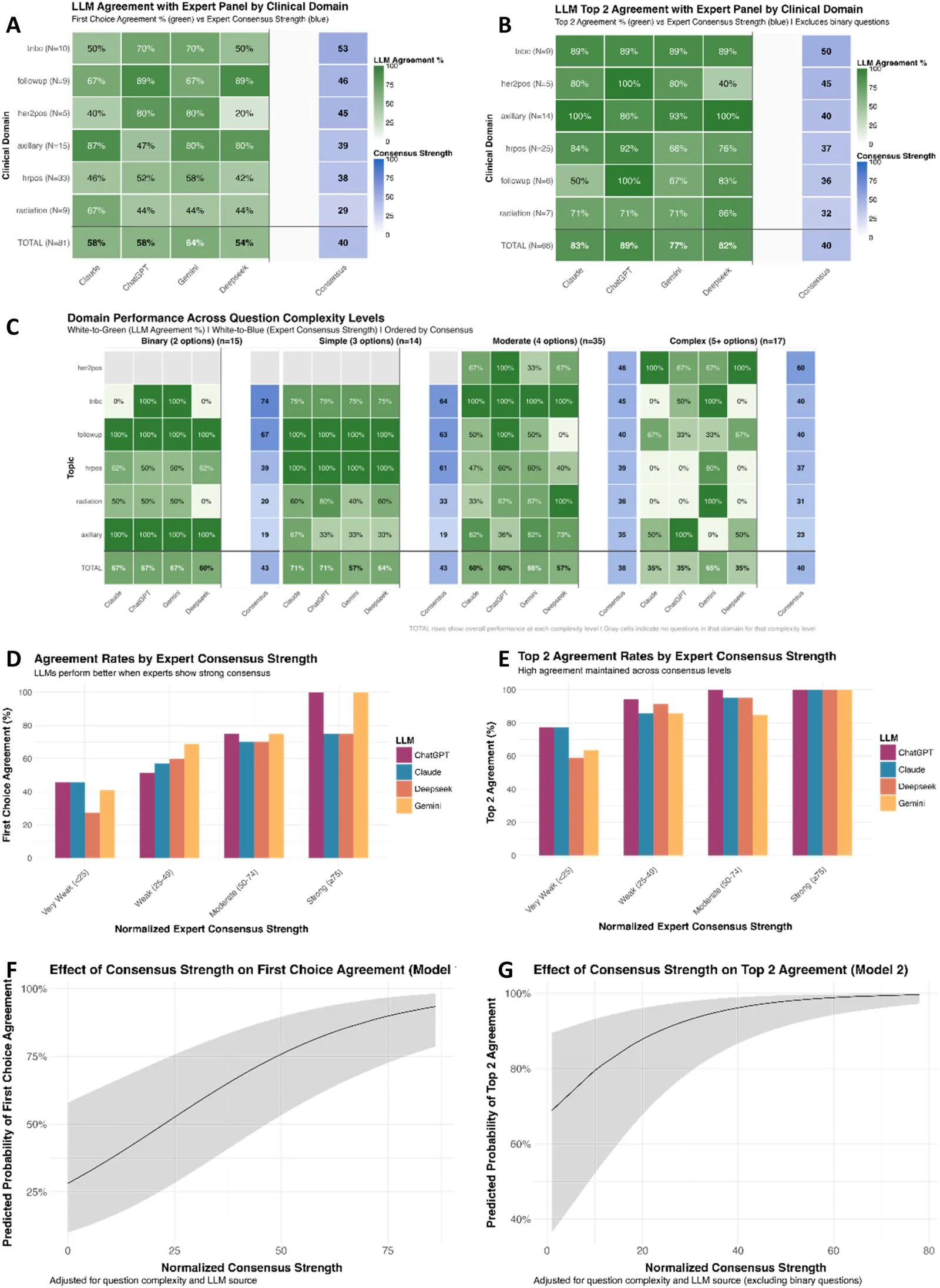
Quantitative analysis of concordance between LLMs and the St. Gallen 2025 expert panel. (A) First-choice agreement by clinical domain (axillary, follow-up, HER2-positive, HR-positive, radiation, triple-negative). (B) Top-2 agreement by domain (excludes binary questions). (C) First-choice agreement stratified by question complexity (binary: 2 options; simple: 3 options; moderate: 4 options; complex: ≥5 options). Association between normalized consensus strength and first-choice agreement (D) and top-2 agreement (E). Predicted probabilities for first-choice (F) and top-2 agreement (G) from mixed-effects logistic regression (error bars/bands denote 95% CIs).

Stratification by question complexity showed variation in LLM performance (Fig. 1C). Binary questions (2 options) achieved first-choice agreement rates of 60-67% across all four LLMs, and agreement declined as the number of options increased (3 options, 57-71%; 4 options, 57-66%; 5-7 options, 35-65%). However, when we consider the option-count-weighted null models, this ordering reverses. Pooled across models, agreement on binary questions did not significantly exceed chance (65.0% vs 50.0% expected; +15.0 percentage points, 95% CI −5.0 to 33.3), whereas agreement exceeded chance for questions with three options (66.1% vs 33.3%), four options (60.7% vs 25.0%), five or more options (42.6% vs 18.8%) and overall (58.6% vs 29.8%; +28.9, 95% CI 21.4 to 36.4; Supplementary Table S1).

There was a strong positive association between normalized expert consensus strength and LLM agreement (Fig. 1D and E): agreement remained below 50% where consensus was weak and reached 75–100% where the panel was near-unanimous. Top-2 agreement followed the same pattern from a higher baseline (Fig. 1E).

In the multivariable mixed-effects logistic regression models, normalized consensus strength was robustly associated with LLM-expert concordance. Each 1-point increase in consensus strength was associated with a 1.04-fold increase in odds of first-choice agreement (95% CI: 1.02-1.06, p < 0.001) and a 1.06-fold increase in odds of top-2 agreement (95% CI: 1.03-1.09, p < 0.001). These relationships are visualized through predicted probability curves (Fig. 1F and G), which demonstrate a gradual increase in LLM-expert concordance probability as expert panel consensus strength increases from chance levels to near-unanimity. Question complexity and LLM source showed weaker and less consistent associations with agreement rates (Table 1). Bootstrap analyses with 5000 iterations confirmed the robustness of these estimates (Table 1).

**Table 1:** Mixed-effects logistic regression analysis of factors associated with LLM-expert panel agreement.

Model	Parameter	OR	Model-based 95% CI	P-value	Bootstrap 95% CI
**Model 1**	Intercept	0.39	0.11-1.44	0.16	0.10-1.28
	Consensus Strength (per unit)	1.04	1.02-1.06	<0.001	1.02-1.07
	Simple vs Binary	1.03	0.28-3.80	0.97	0.27-3.78
	Moderate vs Binary	0.93	0.32-2.74	0.90	0.31-2.63
	Complex vs Binary	0.33	0.10-1.11	0.07	0.09-1.02
	Claude vs ChatGPT	1.0	0.47-2.12	1.00	0.46-2.11
	Deepseek vs ChatGPT	0.8	0.38-1.70	0.57	0.38-1.71
	Gemini vs ChatGPT	1.46	0.68-3.13	0.33	0.69-3.18
**Model 2**	Intercept	2.08	0.53-8.17	0.30	0.56-11.52
	Consensus Strength (per unit)	1.06	1.03-1.09	<0.001	1.04-1.11
	Moderate vs Simple	0.83	0.27-2.57	0.75	0.19-2.48
	Complex vs Simple	0.24	0.07-0.78	0.02	0.05-0.69
	Claude vs ChatGPT	0.54	0.18-1.63	0.27	0.14-1.63
	Deepseek vs ChatGPT	0.47	0.16-1.42	0.18	0.13-1.38
	Gemini vs ChatGPT	0.33	0.11-0.98	0.046	0.08-0.88

Model 1 examines first-choice agreement (n = 324 observations: 4 LLMs × 81 questions). Model 2 examines top-2 agreement excluding binary questions (n = 264 observations: 4 LLMs × 66 questions). Both models include random intercepts for question identification to account for clustering. Odds ratios (OR) are presented with 95% confidence intervals derived from both model-based estimation and parametric bootstrap resampling (5000 iterations). Consensus strength represents normalized expert panel agreement (0-100 scale). Question complexity categories: binary (2 options), simple (3 options), moderate (4 options), complex (≥5 options). ChatGPT serves as the reference category for LLM comparisons. The intercept represents the log-odds of agreement for the reference categories (binary questions, ChatGPT model) when consensus strength equals zero, and is included for model completeness.

No response referred to the St. Gallen 2025 conference, its consensus statement, or any derivative publication; the panel's vote distributions were in any case first published after all queries had been completed. Sources post-dating the stated evidence cutoff were cited in a minority of responses and consisted predominantly of NCCN Breast Cancer guideline versions v3.2025 and v4.2025, which are developed independently of the St. Gallen process (Supplementary Table S2).

### 3.2 Qualitative analysis of LLM performance

“AI consensus” was present in 41 of 81 questions, with three or four LLMs selecting the panels' 1st choice. ”No AI consensus”, which are scenarios in which two or fewer LLMs matched the experts’ first choice, accounted for 40 of 81 questions. All panel votes, LLM choices and LLM rationales are provided in an interactive table in the online Supplementary Materials.

Negative divergence accounted for 18 of 81 (22%) questions (Fig. 2). These errors reflected outdated or missing evidence and misinterpretation, including inappropriate extrapolation: LLMs assumed efficacy of adjuvant PD-1 blockade in early triple-negative breast cancer based on neoadjuvant data, despite existing negative adjuvant trials [24]. The majority of errors occurred in systemic therapy for HR-positive breast cancer (13/18; 72%), and 9 of these concerned adjuvant CDK4/6 administration; in 7 scenarios none of the LLMs referenced the NATALEE trial [25], although the same data were applied correctly by one model in other scenarios, indicating that the omission was not one of data accessibility. Remaining divergences reflected difficulties in risk appraisal based on stage and genomic risk.

**Fig. 2:**
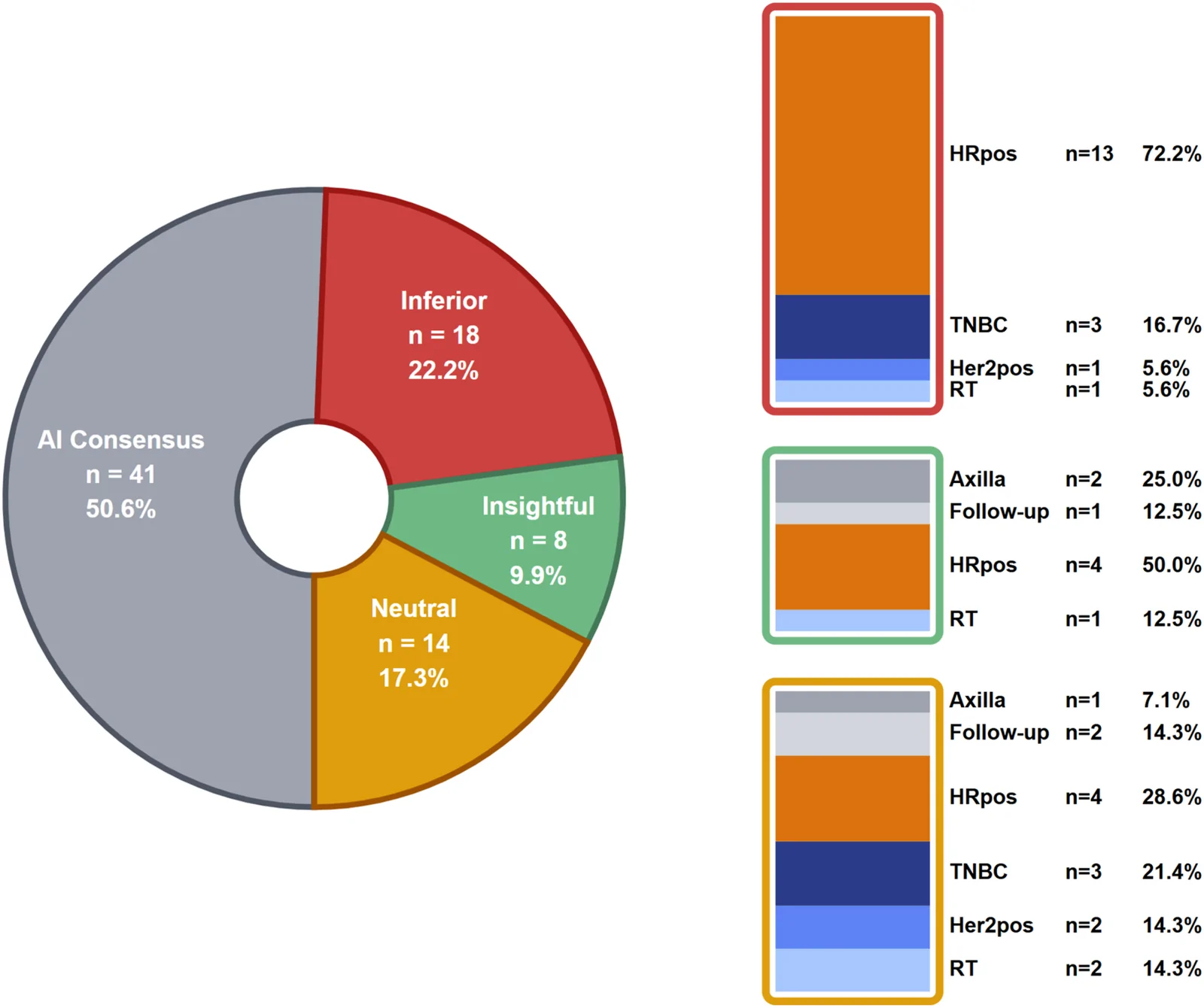
Qualitative analysis of LLM–expert divergences. Circular plot (left) depicts distribution of LLM-answer categories: AI Consensus, inferior = negative divergence (evidence omissions or misinterpretation, red), neutral divergence (defensible alternatives within ambiguous scenarios, orange), and insightful = positive divergence (evidence-consistent options, green). Bar plots (right) show proportions of domains among the three divergence-categories.

Neutral divergence comprised 14 of 81 (17%) questions. Here, LLM rationales were technically correct but prioritized different, still acceptable, considerations. LLMs’ choices reflected genuine clinical ambiguity, with an even distribution of neutral divergence scenarios across all six domains (Fig. 2).

Positive divergence occurred in 8 of 81 (10%) questions. In two scenarios LLMs favored neoadjuvant endocrine therapy over chemotherapy for Luminal A tumors [26,27]. In others they recommended omission of axillary radiotherapy for residual isolated tumor cells in line with National Comprehensive Cancer Network guidance [28], argued for adjuvant chemotherapy in a scenario of intermediate Oncotype Recurrence Score and 4 positive axillary lymph nodes not tested in RxPONDER [29], and cautioned that evidence from the CALOR trial is weak [30]. In one scenario all four LLMs used post-neoadjuvant rather than pre-treatment stage to judge eligibility for adjuvant CDK4/6 inhibition, inconsistent with the inclusion criteria of monarchE [31] and NATALEE [25], yet raising a defensible de-escalation perspective after pathological complete response. Individual scenarios are described in an interactive table in the online Supplementary Materials.

## 4 Discussion

Our findings suggest that LLM performance is strongly associated with the consensus among experts. This implies that LLMs, at least in their current form, function less as creative reasoners and more as amplifiers of the prevailing evidence: when the evidence base is coherent and professional consensus is strong, LLMs reliably converge; when evidence is fragmentary or contested, their answers can disperse. This pattern aligns with systematic evaluations demonstrating that LLMs struggle with complex clinical decision-making when evidence is fragmentary. Hager et al. [19] showed that current LLMs fail to consistently follow diagnostic and treatment guidelines and perform significantly worse than physicians in realistic clinical scenarios. A systematic review by Shan et al. [32] comparing 19 LLMs against clinicians found that while LLM diagnostic accuracy ranged widely (25-97.8%), it generally remained below physician performance, particularly in complex or ambiguous clinical scenarios. The chance-referenced analysis qualifies this picture: absolute agreement was highest for binary questions but did not exceed what uninformed selection would produce, whereas the most complex questions, with the lowest absolute agreement, departed most clearly from chance. Reporting raw agreement without reference to option count therefore flatters performance on simple questions and understates it on complex ones.

Our investigation is different from prior studies of AI-assisted medical decision-making that have assessed performance against an established ground truth [33,34]. The St. Gallen consensus conference, by design, addresses clinical scenarios where such ground truth does not exist – clinical scenarios characterized by equipoise, conflicting evidence, or even insufficient data. Therefore, our analysis evaluates LLM performance in scenarios where AI assistance may be most needed, but yet least validated: decisions that cannot be definitively classified as 'correct' or 'incorrect' at the time they are made. This shifts the core question of this analysis from accuracy of AI, to whether AI improves the quality and transparency of expert deliberation.

Our qualitative review showed that roughly one fifth of questions revealed negative divergence – choices based on incomplete evidence or misinterpretation: models could omit pivotal trials, misweigh risk (e.g., stage plus genomic score), and base treatment decisions solely on guidelines without critical analysis of the underlying evidence. These flaws reflect known limitations of current LLMs: they may lack up-to-date knowledge and can confidently present incorrect or guideline-non-adherent answers [19,35]. This is especially concerning given the risk of automation bias, whereby clinicians overtrust AI outputs and may select a less effective or riskier treatment [36], through both omission and commission errors [37].

We categorized 17% of clinical scenarios as neutral divergence, with LLM rationales often defensible despite deviating from the panel's majority. Positive divergence, in which models suggested evidence-consistent options the panel had underweighted, was in the minority (10%) but warrants systematic evaluation in prospective studies. Rather than direct inclusion in voting panels, LLMs may serve as 'sparring partners' to expert groups; such an approach has been tested in a randomized controlled trial in which GPT-4 assistance improved physicians' chest-pain triage accuracy [33].

Two further points bear on how this work should be read. First, commercial large language models are continuously revised and withdrawn, typically within months; all four models evaluated here have since been superseded or retired. Such evaluations are therefore not fully reproducible, and reported performance should be read as a time-stamped observation rather than a stable property of the model. Second, because these scenarios have by construction no ground truth, a confirmatory study cannot use diagnostic accuracy as its endpoint. We propose a randomized, within-conference design in which panelists vote either normally or after receiving a structured, source-linked AI synthesis, with the change in vote dispersion as the primary endpoint against a pre-specified minimal important difference.

Our study has limitations. First, it analyzed a single conference and four contemporary models; results may differ with other models, prompts or forums, and agreement with the panel is an imperfect proxy for clinical correctness because consensus itself reflects evolving evidence and value judgements. Second, each question was submitted to each model only once. LLM outputs are stochastic, and the sampling parameters, retrieval behavior and model routing of commercial interfaces are neither controlled nor observable. A single response therefore characterizes one realization of model behavior rather than the behavior of the model, and the apparent ordering of models must not be read as a ranking; indeed the four models selected the same option in only 29 of 81 questions (Fleiss' κ 0.46). The association between consensus strength and agreement, estimated across 81 questions and four model families, is robust to this limitation, whereas the between-model comparisons are not. Third, although we instructed models to restrict evidence to publications through February 2025 and audited their self-reported search documentation, this audit rests on what the models reported, and one model recorded only that a search had been performed; we found citations to guideline versions post-dating the cutoff and cannot exclude undocumented retrieval. Fourth, the classification of divergence in the 40 “no AI consensus” scenarios is subjective, and additional reviewers would not remove the bias of knowing both panel and LLM answers. Finally, this study is exploratory: a single-arm, non-interventional comparison against a completed vote, without a pre-registered hypothesis or a control condition; its findings are hypothesis-generating and do not estimate the clinical value of LLM assistance.

## 5 Conclusions

The strong association between consensus strength and LLM agreement suggests that current models mainly amplify existing evidence. Selective integration of AI-generated perspectives as discussion prompts – rather than voting participants – might stimulate more explicit articulation of expert reasoning in ambiguous scenarios. We propose to systematically investigate how LLMs can impact expert decision making by integrating AI suggestions in the voting procedure of future consensus conferences.

## CRediT authorship contribution statement

**Anton Oseledchyk:** Writing – review & editing, Writing – original draft, Methodology, Formal analysis, Data curation, Conceptualization. **Walter Paul Weber:** Writing – review & editing, Conceptualization. **Benjamin Kasenda:** Writing – review & editing, Writing – original draft, Visualization, Software, Methodology, Conceptualization.

## Funding/support

This work received no direct financial support. Anton Oseledchyk is personally supported by the Geistlich Stucki Stiftung (protected research time).

## Role of the funder/sponsor

The funders had no role in the design and conduct of the study; collection, management, analysis, and interpretation of the data; preparation, review, or approval of the manuscript; and decision to submit the manuscript for publication.

## Declaration of competing interest

The authors declare the following financial interests/personal relationships which may be considered as potential competing interests: Anton Oseledchyk reports a relationship with Merck & Co Inc that includes: consulting or advisory. Anton Oseledchyk reports a relationship with Stemline Therapeutics Inc that includes: consulting or advisory. Anton Oseledchyk reports a relationship with Daiichi Sankyo Inc that includes: travel reimbursement. Anton Oseledchyk reports a relationship with Gilead Sciences Inc that includes: consulting or advisory and travel reimbursement. Anton Oseledchyk reports a relationship with Pfizer Inc that includes: travel reimbursement. Anton Oseledchyk reports a relationship with AstraZeneca Pharmaceuticals LP that includes: speaking and lecture fees and travel reimbursement. Benjamin Kasenda reports a relationship with Astellas Pharma Inc that includes: consulting or advisory. Benjamin Kasenda reports a relationship with F Hoffmann-La Roche Ltd that includes: consulting or advisory. Benjamin Kasenda reports a relationship with AbbVie Inc that includes: consulting or advisory. Benjamin Kasenda reports a relationship with Incyte Corporation that includes: consulting or advisory. Anton Oseledchyk reports a relationship with F Hoffmann-La Roche Ltd that includes: travel reimbursement. Walter Weber reports a relationship with Agendia Inc that includes: funding grants. Walter Weber reports a relationship with Merck & Co Inc that includes: funding grants. If there are other authors, they declare that they have no known competing financial interests or personal relationships that could have appeared to influence the work reported in this paper.

## Appendix A Supplementary data

supplementary tables

interactive table of LLM responses
