# Supplementary material for: Performance of large language models in ambiguous treatment scenarios of early breast cancer
Source: Breast. 2026 Sep 23;90:104932. doi: 10.1016/j.breast.2026.104932 (PMC13641045; doi:10.1016/j.breast.2026.104932)
Supplement: Supplementary file 1 [file mmc1.zip › Suppl.Mat. - Anton Oseledchyk - Prompts, LLM Responses, R-Code/0_AnalysesAllOutput.html]

Comparison of AI Responses to Expert Consensus in Breast Cancer Treatment Decisions


Code 

- Show All Code
- Hide All Code

# Comparison of AI Responses to Expert Consensus in Breast Cancer Treatment Decisions

#### Benjamin Kasenda

#### 2025-10-25

# 1 Data Preparation

## 1.1 Load Required Libraries

```
# Core packages
library(tidyverse)
library(knitr)
library(kableExtra)
library(DT)

# For visualization
library(ggplot2)
library(plotly)
library(viridis)
library(scales)
library(ggnewscale)
library(patchwork)

# For statistical analysis
library(broom)
library(corrplot)
library(MASS)
library(ordinal)
library(ggeffects)
library(emmeans)

# For data manipulation
library(janitor)
library(stringr)

# Additional packages for final analyses
library(gridExtra)
library(cowplot)
```

## 1.2 Load Data Sources

```
# Load the DeepSeek responses
deepseek_response <- readRDS("output/deepseek_responses.rds")
deepseek_response$source <- rep("Deepseek", nrow(deepseek_response))

# Load the AI combined responses
gemini_response <- readRDS("output/gemini_responses.rds")
gemini_response$source <- rep("Gemini", nrow(gemini_response))

# Claude and ChatGPT answers
combined_responses <- readRDS("output/combined_responses.rds")

# Now combine answers from all 4 LLMs
combined_responses <- rbind(combined_responses, gemini_response, deepseek_response)

# Load the expert structured voting data
expert_structured <- readRDS("output/expert_structured.rds")

# Display basic information about the datasets
cat("Combined AI Responses Dataset:\n")
```

```
## Combined AI Responses Dataset:
```

```
cat("Dimensions:", dim(combined_responses)[1], "rows x", dim(combined_responses)[2], "columns\n")
```

```
## Dimensions: 324 rows x 11 columns
```

```
cat("Columns:", paste(names(combined_responses), collapse=", "), "\n\n")
```

```
## Columns: question_id, topic, question_text, selected_answer, evidence_summary, key_references, guideline_alignment, subgroup_notes, confidence_level, web_search_used, source
```

```
cat("Expert Structured Dataset:\n")
```

```
## Expert Structured Dataset:
```

```
cat("Dimensions:", dim(expert_structured)[1], "rows x", dim(expert_structured)[2], "columns\n")
```

```
## Dimensions: 81 rows x 17 columns
```

```
cat("Columns:", paste(names(expert_structured), collapse=", "), "\n")
```

```
## Columns: question_id, topic, question_text, Answer Choice A, % A, Answer Choice B, % B, Answer Choice C, % C, Answer Choice D, % D, Answer Choice E, % E, Answer Choice F, % F, Answer Choice G, % G
```

## 1.3 Harmonize Topic Names

```
# Check original topic distributions
cat("Original topic distributions:\n")
```

```
## Original topic distributions:
```

```
cat("\nAI Responses topics:\n")
```

```
## 
## AI Responses topics:
```

```
print(table(combined_responses$topic))
```

```
## 
##            axillary Axillary Management           Follow-up            followup 
##                  15                  45                  27                   9 
##       HER2-positive             her2pos         HR-positive               hrpos 
##                  15                   5                  99                  33 
##           radiation           Radiation                tnbc                TNBC 
##                   9                  27                  10                  30
```

```
cat("\nExpert topics:\n")
```

```
## 
## Expert topics:
```

```
print(table(expert_structured$topic))
```

```
## 
##                       Axillary Management 
##                                        15 
## Follow-Up / Survival / Recurrence / Other 
##                                         9 
##                         Radiation Therapy 
##                                         9 
##                Systemic Treatment Her2pos 
##                                         5 
##          Systemic Treatment HRpos Her2neg 
##                                        33 
##                   Systemic Treatment TNBC 
##                                        10
```

```
# Harmonize topic names in combined_responses
combined_responses <- combined_responses %>%
  mutate(
    topic_original = topic,  # Keep original for reference
    topic = case_when(
      tolower(topic) %in% c("axillary", "axillary management") ~ "axillary",
      tolower(topic) %in% c("follow-up", "followup") ~ "followup",
      tolower(topic) %in% c("her2-positive", "her2pos") ~ "her2pos",
      tolower(topic) %in% c("hr-positive", "hrpos") ~ "hrpos",
      tolower(topic) %in% c("radiation", "radiation therapy") ~ "radiation",
      tolower(topic) %in% c("tnbc", "systemic treatment tnbc") ~ "tnbc",
      TRUE ~ tolower(topic)
    )
  )

# Harmonize topic names in expert_structured
expert_structured <- expert_structured %>%
  mutate(
    topic_original = topic,  # Keep original for reference
    topic = case_when(
      str_detect(tolower(topic), "axillary") ~ "axillary",
      str_detect(tolower(topic), "follow-up|follow up|survival|recurrence") ~ "followup",
      str_detect(tolower(topic), "radiation") ~ "radiation",
      str_detect(tolower(topic), "her2pos|her2-pos") ~ "her2pos",
      str_detect(tolower(topic), "hrpos|hr-pos|her2neg|her2-neg") & 
        str_detect(tolower(topic), "systemic") ~ "hrpos",
      str_detect(tolower(topic), "tnbc") ~ "tnbc",
      TRUE ~ tolower(topic)
    )
  )

# Verify harmonization
cat("\n\nHarmonized topic distributions:\n")
```

```
## 
## 
## Harmonized topic distributions:
```

```
cat("\nAI Responses topics (after harmonization):\n")
```

```
## 
## AI Responses topics (after harmonization):
```

```
print(table(combined_responses$topic))
```

```
## 
##  axillary  followup   her2pos     hrpos radiation      tnbc 
##        60        36        20       132        36        40
```

```
cat("\nExpert topics (after harmonization):\n")
```

```
## 
## Expert topics (after harmonization):
```

```
print(table(expert_structured$topic))
```

```
## 
##  axillary  followup   her2pos     hrpos radiation      tnbc 
##        15         9         5        33         9        10
```

## 1.4 Data Quality Checks

```
# Check missing values in AI responses
ai_missing <- combined_responses %>%
  summarise_all(~sum(is.na(.))) %>%
  pivot_longer(everything(), names_to = "Variable", values_to = "Missing_Count") %>%
  filter(Missing_Count > 0)

if(nrow(ai_missing) > 0) {
  ai_missing %>%
    kable(caption = "Missing Values in AI Responses") %>%
    kable_styling(bootstrap_options = c("striped", "hover"))
} else {
  cat("No missing values in AI Responses dataset\n")
}
```

```
## No missing values in AI Responses dataset
```

```
# Check missing values in Expert data
expert_missing <- expert_structured %>%
  summarise_all(~sum(is.na(.))) %>%
  pivot_longer(everything(), names_to = "Variable", values_to = "Missing_Count") %>%
  filter(Missing_Count > 0)

if(nrow(expert_missing) > 0) {
  expert_missing %>%
    kable(caption = "Missing Values in Expert Voting") %>%
    kable_styling(bootstrap_options = c("striped", "hover"))
} else {
  cat("No missing values in Expert Voting dataset\n")
}
```

Missing Values in Expert Voting

| Variable | Missing\_Count |
| --- | --- |
| Answer Choice C | 15 |
| % C | 15 |
| Answer Choice D | 29 |
| % D | 28 |
| Answer Choice E | 64 |
| % E | 64 |
| Answer Choice F | 77 |
| % F | 77 |
| Answer Choice G | 78 |
| % G | 78 |

## 1.5 Question Complexity Assignment

```
# Count the number of answer options for each question in expert_structured
question_options_count <- expert_structured %>%
  dplyr::select(question_id, starts_with("Answer Choice")) %>%
  pivot_longer(
    cols = starts_with("Answer Choice"),
    names_to = "option",
    values_to = "text"
  ) %>%
  filter(!is.na(text)) %>%
  group_by(question_id) %>%
  summarise(
    n_options = n(),
    .groups = "drop"
  )

# Add the question type (number of options) to combined_responses
combined_responses <- combined_responses %>%
  left_join(
    question_options_count %>% 
      rename(question_type = n_options),
    by = "question_id"
  )

# Verify the distribution of question types
cat("Distribution of question types (number of answer options):\n")
```

```
## Distribution of question types (number of answer options):
```

```
print(table(combined_responses$question_type))
```

```
## 
##   2   3   4   5   6   7 
##  60  56 140  52   4  12
```

## 1.6 Data Merging and Agreement Calculation

```
# Parse and standardize confidence levels
combined_responses <- combined_responses %>%
  mutate(
    confidence_clean = case_when(
      str_detect(confidence_level, "^High") ~ "High",
      str_detect(confidence_level, "^Moderate") ~ "Moderate",
      str_detect(confidence_level, "^Low") ~ "Low",
      TRUE ~ "Other"
    )
  )

# 1. First, create expert_percentages from expert_structured
expert_percentages <- expert_structured %>%
  dplyr::select(question_id, matches("^% [A-G]$")) %>%
  rename_with(~str_replace(., "% ", "expert_pct_"), matches("^% [A-G]$"))

# 2. Prepare expert data with full distribution and rankings
expert_full_distribution <- expert_structured %>%
  dplyr::select(question_id, matches("^% [A-G]$"), starts_with("Answer Choice")) %>% 
  pivot_longer(cols = matches("^% [A-G]$"),
               names_to = "answer_option",
               values_to = "percentage") %>%
  mutate(
    answer_option = str_extract(answer_option, "[A-G]"),
    percentage = percentage * 100  # Convert proportion to percentage
  ) %>%
  
  # Add the answer choice text based on the letter
  mutate(
    answer_text = case_when(
      answer_option == "A" ~ `Answer Choice A`,
      answer_option == "B" ~ `Answer Choice B`,
      answer_option == "C" ~ `Answer Choice C`,
      answer_option == "D" ~ `Answer Choice D`,
      answer_option == "E" ~ `Answer Choice E`,
      answer_option == "F" ~ `Answer Choice F`,
      answer_option == "G" ~ `Answer Choice G`,
      TRUE ~ NA_character_
    )
  ) %>%
  group_by(question_id) %>%
  arrange(desc(percentage)) %>%
  mutate(
    expert_rank = row_number(),
    is_modal = expert_rank == 1,
    is_top2 = expert_rank <= 2,
    is_top3 = expert_rank <= 3
  ) %>%
  ungroup() %>%
  # Clean up - remove the individual Answer Choice columns
  dplyr::select(-starts_with("Answer Choice"))

# 3. Get expert rankings for each question
expert_rankings <- expert_full_distribution %>%
  filter(percentage > 0) %>%
  group_by(question_id) %>%
  summarise(
    expert_1st = answer_option[expert_rank == 1][1],
    expert_1st_pct = percentage[expert_rank == 1][1],
    expert_2nd = answer_option[expert_rank == 2][1],
    expert_2nd_pct = percentage[expert_rank == 2][1],
    expert_3rd = answer_option[expert_rank == 3][1],
    expert_3rd_pct = percentage[expert_rank == 3][1],
    n_options_with_votes = n(),
    .groups = "drop"
  )

# 4. Calculate consensus metrics with NORMALIZED consensus strength
expert_consensus <- expert_structured %>%
  rowwise() %>%
  mutate(
    # Get all percentage columns (they're in proportions 0-1)
    max_agreement_prop = max(c_across(matches("^% [A-G]$")), na.rm = TRUE),
    
    # Convert to percentage (0-100 scale)
    max_agreement = max_agreement_prop * 100,
    
    # Count total number of available options
    n_options_available = sum(!is.na(c_across(starts_with("Answer Choice")))),
    
    # Calculate baseline (random) probability in percentage
    baseline_prob = 100 / n_options_available,
    
    # Normalized consensus strength (0-100 scale)
    consensus_strength = case_when(
      max_agreement <= baseline_prob ~ 0,
      baseline_prob >= 100 ~ 100,
      TRUE ~ (max_agreement - baseline_prob) / (100 - baseline_prob) * 100
    ),
    
    # Keep raw consensus for comparison
    consensus_strength_raw = max_agreement,
    
    # Count how many options have >0% votes
    n_options_chosen = sum(c_across(matches("^% [A-G]$")) > 0, na.rm = TRUE)
  ) %>%
  ungroup()

# 5. Add consensus strength to expert_rankings
expert_rankings <- expert_rankings %>%
  left_join(
    expert_consensus %>% 
      dplyr::select(question_id, consensus_strength, consensus_strength_raw, n_options_available),
    by = "question_id"
  )

# 6. Merge everything with AI responses
comparison_data <- combined_responses %>%
  left_join(expert_rankings, by = "question_id") %>%
  left_join(expert_percentages, by = "question_id") %>%
  mutate(
    # Create multiple agreement levels
    agrees_with_1st = selected_answer == expert_1st,
    agrees_with_2nd = selected_answer == expert_2nd,
    agrees_with_3rd = selected_answer == expert_3rd,
    agrees_with_top2 = agrees_with_1st | agrees_with_2nd,
    agrees_with_top3 = agrees_with_1st | agrees_with_2nd | agrees_with_3rd,
    
    # Get the exact expert percentage for the AI's selected answer
    ai_answer_expert_pct = case_when(
      selected_answer == "A" ~ expert_pct_A * 100,
      selected_answer == "B" ~ expert_pct_B * 100,
      selected_answer == "C" ~ expert_pct_C * 100,
      selected_answer == "D" ~ expert_pct_D * 100,
      selected_answer == "E" ~ expert_pct_E * 100,
      selected_answer == "F" ~ expert_pct_F * 100,
      selected_answer == "G" ~ expert_pct_G * 100,
      TRUE ~ 0
    ),
    
    # Calculate baseline probability using question_type
    baseline_prob = 100 / question_type,
    
    # Calculate how much better than random the AI's choice is
    ai_performance_vs_random = case_when(
      ai_answer_expert_pct <= baseline_prob ~ 0,
      TRUE ~ (ai_answer_expert_pct - baseline_prob) / (100 - baseline_prob) * 100
    ),
    
    # Categorize agreement quality
    agreement_level = case_when(
      agrees_with_1st ~ "1st choice",
      agrees_with_2nd ~ "2nd choice",
      agrees_with_3rd ~ "3rd choice",
      ai_answer_expert_pct > 0 ~ "Lower rank",
      TRUE ~ "No expert support"
    ),
    
    # Agreement quality score (weighted by expert percentage)
    agreement_quality_score = ai_answer_expert_pct / 100
  )

# After step 6, before step 7, add this:

# Get the answer text columns from expert_structured
answer_text_columns <- expert_structured %>%
  dplyr::select(question_id, starts_with("Answer Choice")) %>%
  distinct()

# Add answer text columns to comparison_data
comparison_data <- comparison_data %>%
  left_join(answer_text_columns, by = "question_id")

# If you want to rename them for clarity (optional)
comparison_data <- comparison_data %>%
  rename_with(
    ~ str_replace(., "Answer Choice ", "answer_text_"),
    starts_with("Answer Choice")
  )


# 7. Summary of merged data
cat("Merged Dataset Summary:\n")
```

```
## Merged Dataset Summary:
```

```
cat("Total rows:", nrow(comparison_data), "\n")
```

```
## Total rows: 324
```

```
cat("Questions with expert data:", sum(!is.na(comparison_data$expert_1st)), "\n")
```

```
## Questions with expert data: 324
```

```
cat("Questions without expert data:", sum(is.na(comparison_data$expert_1st)), "\n")
```

```
## Questions without expert data: 0
```

## 1.7 Create Analysis-Ready Dataset

```
# Create a clean dataset for analysis
analysis_dataset <- comparison_data %>%
  filter(!is.na(expert_1st)) %>%
  dplyr::select(
    question_id,
    topic,
    question_text,
    question_type,
    source,
    selected_answer,
    evidence_summary,
    guideline_alignment,
    subgroup_notes,
    key_references,
    expert_1st, expert_1st_pct,
    expert_2nd, expert_2nd_pct,
    expert_3rd, expert_3rd_pct,
    agrees_with_1st, agrees_with_2nd, agrees_with_3rd,
    agrees_with_top2, agrees_with_top3,
    agreement_level,
    ai_answer_expert_pct,
    agreement_quality_score,
    consensus_strength,
    n_options_with_votes,
    confidence_clean,
    web_search_used,
    answer_text_A,
    answer_text_B,
    answer_text_C,
    answer_text_D,
    answer_text_E,
    answer_text_F,
    answer_text_G
  )

# Save for future use
saveRDS(analysis_dataset, "output/analysis_dataset.rds")
write.csv(analysis_dataset, "output/analysis_dataset.csv", row.names = FALSE)

# Create interactive downloadable table with ALL variables
cat("\n### Interactive Data Explorer\n")
```

```
## 
## ### Interactive Data Explorer
```

```
cat("The complete analysis dataset is available below. You can filter, sort, and download the full data in Excel format.\n\n")
```

```
## The complete analysis dataset is available below. You can filter, sort, and download the full data in Excel format.
```

```
datatable(
  analysis_dataset,
  caption = "Complete Analysis Dataset - St. Gallen Conference 2025 LLM Evaluation",
  filter = "top",
  extensions = c('Buttons', 'Scroller'),
  options = list(
    dom = 'Bfrtip',
    buttons = list(
      list(
        extend = 'excel',
        filename = 'StGallen_LLM_Complete_Analysis_Dataset',
        title = 'St. Gallen Conference 2025 - Complete LLM vs Expert Analysis Dataset',
        text = 'Download Full Excel'
      ),
      list(
        extend = 'csv',
        filename = 'StGallen_LLM_Complete_Analysis_Dataset',
        text = 'Download Full CSV'
      )
    ),
    pageLength = 10,
    scrollX = TRUE,
    scrollY = "400px",
    deferRender = TRUE,
    scroller = TRUE,
    columnDefs = list(
      list(visible = TRUE, targets = '_all'),
      list(width = '60px', targets = c(0, 3, 4, 5)),
      list(width = '300px', targets = c(2, 6, 7, 8, 9)),  # Wider for text fields
      list(width = '80px', targets = c(1, 10:27))
    )
  )
) %>%
  formatRound(columns = c('expert_1st_pct', 'expert_2nd_pct', 'expert_3rd_pct', 
                          'ai_answer_expert_pct', 'agreement_quality_score', 
                          'consensus_strength'), 
              digits = 1) %>%
  formatStyle(
    'agrees_with_1st',
    backgroundColor = styleEqual(c(TRUE, FALSE), c('lightgreen', 'lightpink'))
  ) %>%
  formatStyle(
    'agreement_level',
    backgroundColor = styleEqual(
      c("1st choice", "2nd choice", "3rd choice", "Lower rank", "No expert support"),
      c('#90EE90', '#98FB98', '#F0E68C', '#FFE4B5', '#FFB6C1')
    )
  )
```

# 2 Results

## 2.1 Overview of Dataset Characteristics

```
# Summary statistics
cat("Dataset Overview:\n")
```

```
## Dataset Overview:
```

```
cat("Total AI responses analyzed:", nrow(analysis_dataset), "\n")
```

```
## Total AI responses analyzed: 324
```

```
cat("Clinical questions from St. Gallen 2025:", n_distinct(analysis_dataset$question_id), "\n")
```

```
## Clinical questions from St. Gallen 2025: 81
```

```
cat("LLMs evaluated:", paste(unique(analysis_dataset$source), collapse=", "), "\n")
```

```
## LLMs evaluated: Claude, ChatGPT, Gemini, Deepseek
```

```
cat("Clinical domains:", paste(unique(analysis_dataset$topic), collapse=", "), "\n")
```

```
## Clinical domains: axillary, followup, her2pos, hrpos, radiation, tnbc
```

```
# Distribution table
distribution_summary <- analysis_dataset %>%
  group_by(source) %>%
  summarise(
    n_responses = n(),
    n_questions = n_distinct(question_id),
    topics_covered = n_distinct(topic),
    .groups = "drop"
  )

distribution_summary %>%
  kable(caption = "Response Distribution by LLM",
        col.names = c("LLM", "Total Responses", "Questions Answered", "Topics Covered")) %>%
  kable_styling(bootstrap_options = c("striped", "hover"))
```

Response Distribution by LLM

| LLM | Total Responses | Questions Answered | Topics Covered |
| --- | --- | --- | --- |
| ChatGPT | 81 | 81 | 6 |
| Claude | 81 | 81 | 6 |
| Deepseek | 81 | 81 | 6 |
| Gemini | 81 | 81 | 6 |

## 2.2 Primary Outcome: Agreement with Expert Consensus

### 2.2.1 Overall Agreement Rates

```
# Overall agreement statistics
agreement_stats <- analysis_dataset %>%
  group_by(source) %>%
  summarise(
    n = n(),
    pct_1st = round(100 * mean(agrees_with_1st), 1),
    pct_top2 = round(100 * mean(agrees_with_top2), 1),
    pct_top3 = round(100 * mean(agrees_with_top3), 1),
    mean_expert_support = round(mean(ai_answer_expert_pct), 1),
    pct_no_support = round(100 * mean(ai_answer_expert_pct == 0), 1)
  )

agreement_stats %>%
  kable(caption = "Agreement with St. Gallen Expert Panel by LLM",
        col.names = c("LLM", "N", "% Match 1st", "% Match Top 2", "% Match Top 3", 
                     "Mean Expert Support", "% No Support")) %>%
  kable_styling(bootstrap_options = c("striped", "hover"))
```

Agreement with St. Gallen Expert Panel by LLM

| LLM | N | % Match 1st | % Match Top 2 | % Match Top 3 | Mean Expert Support | % No Support |
| --- | --- | --- | --- | --- | --- | --- |
| ChatGPT | 81 | 58.0 | 91.4 | 100.0 | 47.9 | 0.0 |
| Claude | 81 | 58.0 | 86.4 | 96.3 | 46.2 | 0.0 |
| Deepseek | 81 | 54.3 | 84.0 | 95.1 | 45.7 | 0.0 |
| Gemini | 81 | 64.2 | 80.2 | 92.6 | 47.7 | 1.2 |

### 2.2.2 Agreement by Question Complexity

```
# Define complexity categories with factor ordering
analysis_dataset <- analysis_dataset %>%
  mutate(
    question_complexity = case_when(
      question_type == 2 ~ "Binary (2 options)",
      question_type == 3 ~ "Simple (3 options)",
      question_type == 4 ~ "Moderate (4 options)",
      question_type >= 5 ~ "Complex (5+ options)"
    ),
    question_complexity = factor(question_complexity, 
                                levels = c("Binary (2 options)", 
                                         "Simple (3 options)", 
                                         "Moderate (4 options)", 
                                         "Complex (5+ options)"))
  )

# Calculate question counts per complexity level (for first plot)
complexity_n <- analysis_dataset %>%
  group_by(question_complexity) %>%
  summarise(n_questions = n_distinct(question_id), .groups = "drop")

# Agreement by complexity
complexity_agreement <- analysis_dataset %>%
  group_by(question_complexity, source) %>%
  summarise(
    n = n(),
    pct_1st = round(100 * mean(agrees_with_1st), 1),
    mean_consensus = round(mean(consensus_strength), 1),
    .groups = "drop"
  )

# Visualization with question counts
p_complexity <- ggplot(complexity_agreement, 
                       aes(x = question_complexity, y = pct_1st, fill = source)) +
  geom_bar(stat = "identity", position = "dodge", width = 0.7) +
  scale_fill_manual(values = c("Claude" = "#2E86AB", "ChatGPT" = "#A23B72", 
                               "Gemini" = "#FDB863", "Deepseek" = "#E07A5F")) +
  scale_x_discrete(labels = function(x) {
    paste0(x, "\n(n=", complexity_n$n_questions[match(x, complexity_n$question_complexity)], ")")
  }) +
  labs(title = "AI Agreement with St. Gallen Expert Panel by Question Complexity",
       subtitle = "How decision complexity affects AI-expert alignment",
       x = "Question Complexity",
       y = "First Choice Agreement Rate (%)",
       fill = "LLM") +
  theme_minimal() +
  theme(plot.title = element_text(size = 14, face = "bold"),
        plot.subtitle = element_text(size = 12),
        axis.text.x = element_text(angle = 45, hjust = 1))

print(p_complexity)
```

```
# Calculate question counts for top2 plot (excluding binary)
complexity_n_top2 <- analysis_dataset %>%
  filter(question_complexity != "Binary (2 options)") %>%
  group_by(question_complexity) %>%
  summarise(n_questions = n_distinct(question_id), .groups = "drop")

# Agreement by complexity - TOP 2 CHOICES (excluding binary)
complexity_agreement_top2 <- analysis_dataset %>%
  filter(question_complexity != "Binary (2 options)") %>%
  group_by(question_complexity, source) %>%
  summarise(
    n = n(),
    pct_top2 = round(100 * mean(agrees_with_top2), 1),
    mean_consensus = round(mean(consensus_strength), 1),
    .groups = "drop"
  )

# Visualization with question counts
p_complexity_top2 <- ggplot(complexity_agreement_top2, 
                       aes(x = question_complexity, y = pct_top2, fill = source)) +
  geom_bar(stat = "identity", position = "dodge", width = 0.7) +
  scale_fill_manual(values = c("Claude" = "#2E86AB", "ChatGPT" = "#A23B72", 
                               "Gemini" = "#FDB863", "Deepseek" = "#E07A5F")) +
  scale_x_discrete(labels = function(x) {
    paste0(x, "\n(n=", complexity_n_top2$n_questions[match(x, complexity_n_top2$question_complexity)], ")")
  }) +
  labs(title = "AI Agreement with St. Gallen Expert Panel Top 2 Choices by Question Complexity",
       subtitle = "How decision complexity affects AI alignment with expert's top 2 choices",
       x = "Question Complexity",
       y = "Top 2 Agreement Rate (%)",
       fill = "LLM") +
  theme_minimal() +
  theme(plot.title = element_text(size = 14, face = "bold"),
        plot.subtitle = element_text(size = 12),
        axis.text.x = element_text(angle = 45, hjust = 1))

print(p_complexity_top2)
```

### 2.2.3 Impact of Expert Consensus Strength

```
# Analyze performance by consensus strength
consensus_categories <- analysis_dataset %>%
  mutate(
    consensus_category = case_when(
      consensus_strength >= 75 ~ "Strong (≥75)",
      consensus_strength >= 50 ~ "Moderate (50-74)",
      consensus_strength >= 25 ~ "Weak (25-49)",
      TRUE ~ "Very Weak (<25)"
    ),
    consensus_category = factor(consensus_category, 
                               levels = c("Very Weak (<25)", "Weak (25-49)",
                                        "Moderate (50-74)", "Strong (≥75)"))
  )

consensus_agreement <- consensus_categories %>%
  group_by(consensus_category, source) %>%
  summarise(
    n = n(),
    pct_1st = round(100 * mean(agrees_with_1st), 1),
    .groups = "drop"
  )

# Visualization
p_consensus <- ggplot(consensus_agreement, 
                     aes(x = consensus_category, y = pct_1st, fill = source)) +
  geom_bar(stat = "identity", position = "dodge", width = 0.7) +
  scale_fill_manual(values = c("Claude" = "#2E86AB", "ChatGPT" = "#A23B72", 
                               "Gemini" = "#FDB863", "Deepseek" = "#E07A5F")) +
  labs(title = "Agreement Rates by St. Gallen Panel Consensus Strength",
       subtitle = "LLMs perform better when experts show strong consensus",
       x = "Normalized Expert Consensus Strength",
       y = "First Choice Agreement (%)",
       fill = "LLM") +
  theme_minimal() +
  theme(plot.title = element_text(size = 14, face = "bold"),
        axis.text.x = element_text(angle = 45, hjust = 1))

print(p_consensus)
```

```
# Analyze performance by consensus strength - TOP 2 CHOICES
consensus_categories <- analysis_dataset %>%
  mutate(
    consensus_category = case_when(
      consensus_strength >= 75 ~ "Strong (≥75)",
      consensus_strength >= 50 ~ "Moderate (50-74)",
      consensus_strength >= 25 ~ "Weak (25-49)",
      TRUE ~ "Very Weak (<25)"
    ),
    consensus_category = factor(consensus_category, 
                               levels = c("Very Weak (<25)", "Weak (25-49)",
                                        "Moderate (50-74)", "Strong (≥75)"))
  )

consensus_agreement_top2 <- consensus_categories %>%
  group_by(consensus_category, source) %>%
  summarise(
    n = n(),
    pct_top2 = round(100 * mean(agrees_with_top2), 1),
    .groups = "drop"
  )

# Visualization
p_consensus_top2 <- ggplot(consensus_agreement_top2, 
                     aes(x = consensus_category, y = pct_top2, fill = source)) +
  geom_bar(stat = "identity", position = "dodge", width = 0.7) +
  scale_fill_manual(values = c("Claude" = "#2E86AB", "ChatGPT" = "#A23B72", 
                               "Gemini" = "#FDB863", "Deepseek" = "#E07A5F")) +
  labs(title = "Top 2 Agreement Rates by St. Gallen Panel Consensus Strength",
       subtitle = "LLMs perform better when experts show strong consensus",
       x = "Normalized Expert Consensus Strength",
       y = "Top 2 Agreement (%)",
       fill = "LLM") +
  theme_minimal() +
  theme(plot.title = element_text(size = 14, face = "bold"),
        axis.text.x = element_text(angle = 45, hjust = 1))

print(p_consensus_top2)
```

## 2.3 Heatmaps enhanced

```
# Create the stratified performance data
domain_complexity_performance <- analysis_dataset %>%
  group_by(topic, source, question_complexity) %>%
  summarise(
    n = n(),
    pct_1st = round(100 * mean(agrees_with_1st), 1),
    mean_consensus = round(mean(consensus_strength), 1),
    .groups = "drop"
  )

# *** ADD TOTAL ROWS FOR LLM PERFORMANCE ***
# Calculate TOTAL performance by LLM and complexity (across all domains)
domain_complexity_totals <- analysis_dataset %>%
  group_by(source, question_complexity) %>%
  summarise(
    n = n(),
    pct_1st = round(100 * mean(agrees_with_1st), 1),
    mean_consensus = round(mean(consensus_strength), 1),
    .groups = "drop"
  ) %>%
  mutate(topic = "TOTAL")  # Add TOTAL as the topic

# Combine the domain-specific data with TOTAL rows
domain_complexity_performance <- bind_rows(
  domain_complexity_performance,
  domain_complexity_totals
)

# Define complexity levels for analysis
complexity_levels <- c("Binary (2 options)", "Simple (3 options)",
                       "Moderate (4 options)", "Complex (5+ options)")


# === ENHANCED VERSION WITH SEPARATED EXPERT CONSENSUS COLUMN ===

# Calculate expert consensus by topic and complexity
expert_consensus_by_complexity <- analysis_dataset %>%
  group_by(topic, question_complexity) %>%
  summarise(
    mean_consensus = round(mean(consensus_strength), 1),
    n_questions = n_distinct(question_id),
    .groups = "drop"
  )

# *** ADD TOTAL ROWS FOR EXPERT CONSENSUS ***
# Calculate TOTAL consensus by complexity (across all domains)
expert_consensus_totals <- analysis_dataset %>%
  group_by(question_complexity) %>%
  summarise(
    mean_consensus = round(mean(consensus_strength), 1),
    n_questions = n_distinct(question_id),
    .groups = "drop"
  ) %>%
  mutate(topic = "TOTAL")  # Add TOTAL as the topic

# Combine the domain-specific data with TOTAL rows
expert_consensus_by_complexity <- bind_rows(
  expert_consensus_by_complexity,
  expert_consensus_totals
)

# Calculate total questions per complexity level
questions_per_complexity <- analysis_dataset %>%
  group_by(question_complexity) %>%
  summarise(n_total = n_distinct(question_id), .groups = "drop")

# === CREATE SEPARATE PLOTS FOR EACH COMPLEXITY LEVEL (TOTAL at Bottom) ===
for(complexity in complexity_levels) {
  
  # Get the number of questions for this complexity level
  n_questions <- questions_per_complexity %>%
    filter(question_complexity == complexity) %>%
    pull(n_total)
  
  # Get expert data (EXCLUDING TOTAL) to determine domain ordering
  expert_data_domains <- expert_consensus_by_complexity %>%
    filter(question_complexity == complexity, topic != "TOTAL") %>%
    arrange(mean_consensus)  # Sort by consensus strength (ascending)
  
  # Get TOTAL row for expert data
  expert_data_total <- expert_consensus_by_complexity %>%
    filter(question_complexity == complexity, topic == "TOTAL")
  
  # Create complete topic order: TOTAL at start (bottom) + domains (by consensus)
  topic_order <- c("TOTAL", expert_data_domains$topic) # <--- UPDATED: TOTAL FIRST
  
  # Combine expert data (domains + TOTAL) and add source column
  expert_data <- bind_rows(expert_data_domains, expert_data_total) %>%
    mutate(
      source = "Expert Consensus",
      topic = factor(topic, levels = topic_order)
    )
  
  # Get LLM data (EXCLUDING TOTAL) for domains
  llm_data_domains <- domain_complexity_performance %>%
    filter(question_complexity == complexity, topic != "TOTAL")
  
  # Get TOTAL rows for LLM data
  llm_data_total <- domain_complexity_performance %>%
    filter(question_complexity == complexity, topic == "TOTAL")
  
  # Combine LLM data (domains + TOTAL) and apply factor ordering
  llm_data <- bind_rows(llm_data_domains, llm_data_total) %>%
    mutate(topic = factor(topic, levels = topic_order))
  
  # Create the plot with separation
  p <- ggplot() +
    # LLM tiles with white-to-green gradient
    geom_tile(data = llm_data,
              aes(x = source, y = topic, fill = pct_1st),
              color = "white", size = 0.5) +
    scale_fill_gradient(low = "white", high = "#2E7D32",
                        limits = c(0, 100),
                        name = "LLM Agreement %",
                        guide = guide_colorbar(order = 1)) +
    # Add new scale for expert data
    new_scale_fill() +
    # Expert tiles with white-to-blue gradient
    geom_tile(data = expert_data,
              aes(x = source, y = topic, fill = mean_consensus),
              color = "white", size = 0.5) +
    scale_fill_gradient(low = "white", high = "#1565C0",
                        limits = c(0, 100),
                        name = "Consensus Strength",
                        guide = guide_colorbar(order = 2)) +
    # Text for LLMs with dynamic color
    geom_text(data = llm_data,
              aes(x = source, y = topic,
                  label = paste0(round(pct_1st, 0), "%")),
              size = 3,
              fontface = ifelse(llm_data$topic == "TOTAL", "bold", "plain"),
              color = ifelse(llm_data$pct_1st > 60, "white", "black")) +
    # Text for Expert Consensus - ALL BLACK
    geom_text(data = expert_data,
              aes(x = source, y = topic,
                  label = round(mean_consensus, 0)),
              size = 3,
              color = "black",
              fontface = "bold") +
    # Horizontal line to separate TOTAL row (at bottom)
    geom_hline(yintercept = 1.5, # <--- UPDATED: Line above the first level (TOTAL)
               linetype = "solid", color = "gray30", size = 0.8) +
    # Vertical separator line positioned to create visual separation
    geom_vline(xintercept = 4.5, linetype = "solid", color = "gray40", size = 0.8) +
    # Add extra spacing by adjusting x-axis with ordered y-axis
    scale_x_discrete(expand = expansion(add = c(0.5, 0.5)),
                     limits = c("Claude", "ChatGPT", "Gemini", "Deepseek",
                                "", "Expert Consensus")) +
    scale_y_discrete(limits = topic_order) +  # Apply ordering with TOTAL
    labs(title = paste0("Performance for ", complexity, " (n=", n_questions, ")"),
         subtitle = "LLM Agreement % (green) vs Expert Consensus Strength (blue, normalized) | Ordered by Consensus",
         x = "",
         y = "Clinical Domain",
         caption = "TOTAL row shows overall LLM performance for this complexity level | Domains ordered by consensus strength (ascending)") +
    theme_minimal() +
    theme(
      plot.title = element_text(size = 12, face = "bold"),
      plot.subtitle = element_text(size = 10),
      axis.text.x = element_text(angle = 45, hjust = 1),
      panel.grid.major.x = element_blank(),
      panel.background = element_rect(fill = "gray98", color = NA),
      legend.position = "right"
    )
  
  print(p)
}
```

```
# ----------------------------------------------------------------------

# === ALTERNATIVE: ALL COMPLEXITY LEVELS IN ONE FIGURE (WITH TOTAL & ALIGNMENT) ===

# Get all unique topics across all complexity levels (including TOTAL)
all_topics <- c(
  analysis_dataset %>% distinct(topic) %>% pull(topic),
  "TOTAL"
)

all_plots <- list()

for(i in seq_along(complexity_levels)) {
  complexity <- complexity_levels[i]
  
  # Get the number of questions for this complexity level
  n_questions <- questions_per_complexity %>%
    filter(question_complexity == complexity) %>%
    pull(n_total)
  
  # Get expert data (domains only, no TOTAL) and create full topic list
  expert_data_domains <- expert_consensus_by_complexity %>%
    filter(question_complexity == complexity, topic != "TOTAL") %>%
    # Ensure all possible topics are present
    complete(topic = setdiff(all_topics, "TOTAL"),
             fill = list(mean_consensus = NA)) %>%
    arrange(mean_consensus)  # Sort by consensus strength
  
  # Get TOTAL row
  expert_data_total <- expert_consensus_by_complexity %>%
    filter(question_complexity == complexity, topic == "TOTAL")
  
  # Create ordered factor based on consensus strength
  # *** UPDATED: TOTAL is now the FIRST element to plot at the bottom of the y-axis ***
  topic_order <- c(
    "TOTAL", # <--- MOVED TO THE START
    expert_data_domains %>% filter(!is.na(mean_consensus)) %>% pull(topic),
    expert_data_domains %>% filter(is.na(mean_consensus)) %>% pull(topic)
  )
  
  # Combine expert data
  expert_data <- bind_rows(expert_data_domains, expert_data_total) %>%
    mutate(
      source = "Consensus",
      topic = factor(topic, levels = topic_order)
    )
  
  # Get LLM data (domains only)
  llm_data_domains <- domain_complexity_performance %>%
    filter(question_complexity == complexity, topic != "TOTAL") %>%
    complete(topic = setdiff(all_topics, "TOTAL"), source,
             fill = list(pct_1st = NA))
  
  # Get LLM TOTAL rows
  llm_data_total <- domain_complexity_performance %>%
    filter(question_complexity == complexity, topic == "TOTAL")
  
  # Combine LLM data
  llm_data <- bind_rows(llm_data_domains, llm_data_total) %>%
    mutate(topic = factor(topic, levels = topic_order))
  
  # Create plot with separation
  p <- ggplot() +
    # LLM tiles
    geom_tile(data = llm_data,
              aes(x = source, y = topic, fill = pct_1st),
              color = "white", size = 0.3) +
    scale_fill_gradient(low = "#F1F8E9", high = "#2E7D32",
                        limits = c(0, 100),
                        na.value = "gray90",
                        guide = "none") +
    new_scale_fill() +
    # Expert tiles
    geom_tile(data = expert_data,
              aes(x = source, y = topic, fill = mean_consensus),
              color = "white", size = 0.3) +
    scale_fill_gradient(low = "#E3F2FD", high = "#1565C0",
                        limits = c(0, 100),
                        na.value = "gray90",
                        guide = "none") +
    # Text labels (only show for non-NA values)
    geom_text(data = llm_data %>% filter(!is.na(pct_1st)),
              aes(x = source, y = topic,
                  label = paste0(round(pct_1st, 0), "%")),
              size = 2.2,
              fontface = ifelse((llm_data %>% filter(!is.na(pct_1st)) %>% pull(topic)) == "TOTAL",
                                "bold", "plain"),
              color = ifelse(llm_data %>% filter(!is.na(pct_1st)) %>% pull(pct_1st) > 60,
                             "white", "black")) +
    geom_text(data = expert_data %>% filter(!is.na(mean_consensus)),
              aes(x = source, y = topic,
                  label = round(mean_consensus, 0)),
              size = 2.2,
              color = "black",
              fontface = "bold") +
    # Horizontal line above TOTAL
    geom_hline(yintercept = 1.5, # <--- UPDATED: Line above the first level (TOTAL)
               linetype = "solid", color = "gray30", size = 0.5) +
    # Vertical separator
    geom_vline(xintercept = 4.5, linetype = "solid", color = "gray40", size = 0.5) +
    scale_x_discrete(expand = expansion(add = c(0.3, 0.3)),
                     limits = c("Claude", "ChatGPT", "Gemini", "Deepseek",
                                "", "Consensus")) +
    scale_y_discrete(limits = topic_order) +
    labs(title = paste0(complexity, " (n=", n_questions, ")"),
         x = "",
         y = if(i == 1) "Domain" else "") +
    theme_minimal() +
    theme(
      plot.title = element_text(size = 9, face = "bold"),
      axis.text.x = element_text(angle = 45, hjust = 1, size = 7),
      axis.text.y = element_text(size = 7),
      axis.title.y = element_text(size = 8),
      panel.grid = element_blank(),
      panel.background = element_rect(fill = "gray98", color = NA),
      plot.margin = margin(5, 5, 5, 5)
    )
  
  all_plots[[i]] <- p
}

# Combine all plots with patchwork
combined_complexity_plot <- wrap_plots(all_plots, ncol = 4) +
  plot_annotation(
    title = "Domain Performance Across Question Complexity Levels",
    subtitle = "White-to-Green (LLM Agreement %) | White-to-Blue (Expert Consensus Strength) | Ordered by Consensus",
    caption = "TOTAL rows show overall performance at each complexity level | Gray cells = no questions in that domain for this complexity",
    theme = theme(
      plot.title = element_text(size = 14, face = "bold"),
      plot.subtitle = element_text(size = 11),
      plot.caption = element_text(size = 9, color = "gray60", hjust = 0)
    )
  )

print(combined_complexity_plot)
```

```
# Save the combined plot
ggsave("output/combined_complexity_heatmaps.png", combined_complexity_plot,
       width = 16, height = 10, dpi = 300)

# ----------------------------------------------------------------------

# === SIMPLE OVERALL HEATMAP: DOMAIN x LLM AGREEMENT (FIRST CHOICE) ===
# This section was already correct, placing "TOTAL" first in the factor levels.

# Calculate LLM performance by domain (all questions aggregated)
domain_performance <- analysis_dataset %>%
  group_by(topic, source) %>%
  summarise(
    n = n(),
    pct_1st = round(100 * mean(agrees_with_1st), 1),
    .groups = "drop"
  )

# Calculate overall LLM performance (across all domains) - TOTAL row for all LLMs
overall_llm_performance <- analysis_dataset %>%
  group_by(source) %>%
  summarise(
    pct_1st = round(100 * mean(agrees_with_1st), 1),
    .groups = "drop"
  ) %>%
  mutate(topic = "TOTAL")

# Combine domain and overall performance
llm_data_combined <- bind_rows(domain_performance, overall_llm_performance)

# Calculate expert consensus by domain WITH question counts
expert_consensus_by_domain <- analysis_dataset %>%
  group_by(topic) %>%
  summarise(
    mean_consensus = round(mean(consensus_strength), 1),
    n_questions = n_distinct(question_id),
    .groups = "drop"
  ) %>%
  mutate(source = "Consensus")

# Calculate overall expert consensus WITH total question count - TOTAL row for Consensus
overall_expert_consensus <- analysis_dataset %>%
  summarise(
    mean_consensus = round(mean(consensus_strength), 1),
    n_questions = n_distinct(question_id)
  ) %>%
  mutate(
    topic = "TOTAL",
    source = "Consensus"
  )

# Combine expert domain and overall
expert_data_combined <- bind_rows(expert_consensus_by_domain, overall_expert_consensus)

# Order topics by consensus strength ASCENDING (lowest to highest)
topic_order_raw <- expert_consensus_by_domain %>%
  arrange(mean_consensus) %>%
  pull(topic)

# Create topic labels with question counts (in ascending order)
topic_labels <- expert_consensus_by_domain %>%
  arrange(mean_consensus) %>%
  mutate(topic_label = paste0(topic, " (N=", n_questions, ")")) %>%
  dplyr::select(topic, topic_label)

# Add TOTAL label with count
total_label <- data.frame(
  topic = "TOTAL",
  topic_label = paste0("TOTAL (N=", overall_expert_consensus$n_questions, ")")
)

topic_labels <- bind_rows(total_label, topic_labels)  # TOTAL first!

# Put TOTAL at the beginning (bottom of plot)
topic_order <- c("TOTAL", topic_order_raw)

# Apply ordering and labels to LLM data
llm_data_combined <- llm_data_combined %>%
  left_join(topic_labels, by = "topic") %>%
  mutate(topic_label = factor(topic_label,
                              levels = topic_labels$topic_label[match(topic_order, topic_labels$topic)]))

# Apply ordering and labels to expert data
expert_data_combined <- expert_data_combined %>%
  left_join(topic_labels, by = "topic") %>%
  mutate(topic_label = factor(topic_label,
                              levels = topic_labels$topic_label[match(topic_order, topic_labels$topic)]))

# Create the heatmap
p_simple_heatmap <- ggplot() +
  # LLM tiles
  geom_tile(data = llm_data_combined,
            aes(x = source, y = topic_label, fill = pct_1st),
            color = "white", size = 0.5) +
  scale_fill_gradient(low = "white", high = "#2E7D32",
                      limits = c(0, 100),
                      name = "LLM Agreement %",
                      guide = guide_colorbar(order = 1)) +
  new_scale_fill() +
  # Expert tiles
  geom_tile(data = expert_data_combined,
            aes(x = source, y = topic_label, fill = mean_consensus),
            color = "white", size = 0.5) +
  scale_fill_gradient(low = "white", high = "#1565C0",
                      limits = c(0, 100),
                      name = "Consensus Strength",
                      guide = guide_colorbar(order = 2)) +
  # Text for LLMs with dynamic color
  geom_text(data = llm_data_combined,
            aes(x = source, y = topic_label,
                label = paste0(round(pct_1st, 0), "%")),
            size = 3.5,
            fontface = ifelse(llm_data_combined$topic == "TOTAL", "bold", "plain"),
            color = ifelse(llm_data_combined$pct_1st > 60, "white", "black")) +
  # Text for Expert Consensus
  geom_text(data = expert_data_combined,
            aes(x = source, y = topic_label,
                label = round(mean_consensus, 0)),
            size = 3.5,
            color = "black",
            fontface = "bold") +
  # Horizontal line to separate TOTAL row
  geom_hline(yintercept = 1.5,
             linetype = "solid", color = "gray30", size = 0.8) +
  # Vertical separator line
  geom_vline(xintercept = 4.5, linetype = "solid", color = "gray40", size = 0.8) +
  # X-axis with gap
  scale_x_discrete(expand = expansion(add = c(0.5, 0.5)),
                   limits = c("Claude", "ChatGPT", "Gemini", "Deepseek",
                              "", "Consensus")) +
  labs(title = "LLM Agreement with St. Gallen Expert Panel by Clinical Domain",
       subtitle = "First Choice Agreement % (green) vs Expert Consensus Strength (blue, normalized) | Ordered by Consensus",
       x = "",
       y = "Clinical Domain",
       caption = "TOTAL row shows overall performance across all domains for all LLMs and overall consensus | Domains ordered by consensus strength (descending)") +
  theme_minimal() +
  theme(
    plot.title = element_text(size = 14, face = "bold"),
    plot.subtitle = element_text(size = 11),
    axis.text.x = element_text(angle = 45, hjust = 1, size = 10),
    axis.text.y = element_text(size = 10),
    panel.grid.major = element_blank(),
    panel.background = element_rect(fill = "gray98", color = NA),
    legend.position = "right",
    plot.caption = element_text(size = 9, color = "gray60", hjust = 0)
  )

print(p_simple_heatmap)
```

```
# Save as high-resolution image
ggsave("output/simple_domain_heatmap.png", p_simple_heatmap,
       width = 10, height = 7, dpi = 300)


# ----------------------------------------------------------------------

# === SIMPLE OVERALL HEATMAP: DOMAIN x LLM AGREEMENT (TOP 2) ===
# This section was also already correct, placing "TOTAL" first in the factor levels.

# Filter out binary questions (question_type == 2)
analysis_dataset_nonbinary <- analysis_dataset %>%
  filter(question_type > 2)  # Only questions with 3+ options

# Calculate LLM performance by domain (non-binary questions) - TOP 2
domain_performance_top2 <- analysis_dataset_nonbinary %>%
  group_by(topic, source) %>%
  summarise(
    n = n(),
    pct_top2 = round(100 * mean(agrees_with_top2), 1),
    .groups = "drop"
  )

# Calculate overall LLM performance (across all domains) - TOP 2 TOTAL
overall_llm_performance_top2 <- analysis_dataset_nonbinary %>%
  group_by(source) %>%
  summarise(
    pct_top2 = round(100 * mean(agrees_with_top2), 1),
    .groups = "drop"
  ) %>%
  mutate(topic = "TOTAL")

# Combine domain and overall performance
llm_data_combined_top2 <- bind_rows(domain_performance_top2, overall_llm_performance_top2)

# Calculate expert consensus by domain WITH question counts (NON-BINARY ONLY)
expert_consensus_by_domain <- analysis_dataset_nonbinary %>%
  group_by(topic) %>%
  summarise(
    mean_consensus = round(mean(consensus_strength), 1),
    n_questions = n_distinct(question_id),
    .groups = "drop"
  ) %>%
  mutate(source = "Consensus")

# Calculate overall expert consensus WITH total question count (NON-BINARY ONLY)
overall_expert_consensus <- analysis_dataset_nonbinary %>%
  summarise(
    mean_consensus = round(mean(consensus_strength), 1),
    n_questions = n_distinct(question_id)
  ) %>%
  mutate(
    topic = "TOTAL",
    source = "Consensus"
  )

# Combine expert domain and overall
expert_data_combined <- bind_rows(expert_consensus_by_domain, overall_expert_consensus)

# Order topics by consensus strength ASCENDING
topic_order_raw <- expert_consensus_by_domain %>%
  arrange(mean_consensus) %>%
  pull(topic)

# Create topic labels with question counts
topic_labels <- expert_consensus_by_domain %>%
  arrange(mean_consensus) %>%
  mutate(topic_label = paste0(topic, " (N=", n_questions, ")")) %>%
  dplyr::select(topic, topic_label)

# Add TOTAL label with count
total_label <- data.frame(
  topic = "TOTAL",
  topic_label = paste0("TOTAL (N=", overall_expert_consensus$n_questions, ")")
)

topic_labels <- bind_rows(total_label, topic_labels)  # TOTAL first!

# Put TOTAL at the beginning (bottom of plot)
topic_order <- c("TOTAL", topic_order_raw)

# Apply ordering and labels to LLM data
llm_data_combined_top2 <- llm_data_combined_top2 %>%
  left_join(topic_labels, by = "topic") %>%
  mutate(topic_label = factor(topic_label,
                              levels = topic_labels$topic_label[match(topic_order, topic_labels$topic)]))

# Apply ordering and labels to expert data
expert_data_combined <- expert_data_combined %>%
  left_join(topic_labels, by = "topic") %>%
  mutate(topic_label = factor(topic_label,
                              levels = topic_labels$topic_label[match(topic_order, topic_labels$topic)]))

# Create the heatmap
p_simple_heatmap_top2 <- ggplot() +
  # LLM tiles
  geom_tile(data = llm_data_combined_top2,
            aes(x = source, y = topic_label, fill = pct_top2),
            color = "white", size = 0.5) +
  scale_fill_gradient(low = "white", high = "#2E7D32",
                      limits = c(0, 100),
                      name = "LLM Agreement %",
                      guide = guide_colorbar(order = 1)) +
  new_scale_fill() +
  # Expert tiles
  geom_tile(data = expert_data_combined,
            aes(x = source, y = topic_label, fill = mean_consensus),
            color = "white", size = 0.5) +
  scale_fill_gradient(low = "white", high = "#1565C0",
                      limits = c(0, 100),
                      name = "Consensus Strength",
                      guide = guide_colorbar(order = 2)) +
  # Text for LLMs with dynamic color
  geom_text(data = llm_data_combined_top2,
            aes(x = source, y = topic_label,
                label = paste0(round(pct_top2, 0), "%")),
            size = 3.5,
            fontface = ifelse(llm_data_combined_top2$topic == "TOTAL", "bold", "plain"),
            color = ifelse(llm_data_combined_top2$pct_top2 > 60, "white", "black")) +
  # Text for Expert Consensus
  geom_text(data = expert_data_combined,
            aes(x = source, y = topic_label,
                label = round(mean_consensus, 0)),
            size = 3.5,
            color = "black",
            fontface = "bold") +
  # Horizontal line to separate TOTAL row
  geom_hline(yintercept = 1.5,
             linetype = "solid", color = "gray30", size = 0.8) +
  # Vertical separator line
  geom_vline(xintercept = 4.5, linetype = "solid", color = "gray40", size = 0.8) +
  # X-axis with gap
  scale_x_discrete(expand = expansion(add = c(0.5, 0.5)),
                   limits = c("Claude", "ChatGPT", "Gemini", "Deepseek",
                              "", "Consensus")) +
  labs(title = "LLM Agreement with St. Gallen Expert Panel by Clinical Domain (Top 2 Choices)",
       subtitle = "Top 2 Agreement % (green) vs Expert Consensus Strength (blue, normalized) | Ordered by Consensus",
       x = "",
       y = "Clinical Domain",
       caption = "Excludes binary questions (2 options) | TOTAL row shows overall performance across all non-binary domains | Domains ordered by consensus strength (descending)") +
  theme_minimal() +
  theme(
    plot.title = element_text(size = 14, face = "bold"),
    plot.subtitle = element_text(size = 11),
    axis.text.x = element_text(angle = 45, hjust = 1, size = 10),
    axis.text.y = element_text(size = 10),
    panel.grid.major = element_blank(),
    panel.background = element_rect(fill = "gray98", color = NA),
    legend.position = "right",
    plot.caption = element_text(size = 9, color = "gray60", hjust = 0)
  )

print(p_simple_heatmap_top2)
```

```
# Save as high-resolution image
ggsave("output/simple_domain_heatmap_top2.png", p_simple_heatmap_top2,
       width = 10, height = 7, dpi = 300)
```

## 2.4 Multivariable Analysis: Predictors of Agreement

```
# =============================================================================
# FINAL MIXED-EFFECTS LOGISTIC REGRESSION MODELS
# =============================================================================

library(lme4)
library(broom.mixed)
library(emmeans)
library(performance)
library(sjPlot)
library(ggeffects)
library(tidyverse)

# =============================================================================
# CONVERT LOGICAL OUTCOMES TO NUMERIC
# =============================================================================

analysis_dataset <- analysis_dataset %>%
  mutate(
    agrees_with_1st_num = as.numeric(agrees_with_1st),
    agrees_with_top2_num = as.numeric(agrees_with_top2),
    agrees_with_top3_num = as.numeric(agrees_with_top3)
  )

# Verify conversion (should show 0s and 1s)
cat("=== Outcome Variable Conversion ===\n")
```

```
## === Outcome Variable Conversion ===
```

```
cat("First choice agreement:\n")
```

```
## First choice agreement:
```

```
table(analysis_dataset$agrees_with_1st_num)
```

```
## 
##   0   1 
## 134 190
```

```
cat("\nTop 2 agreement:\n")
```

```
## 
## Top 2 agreement:
```

```
table(analysis_dataset$agrees_with_top2_num)
```

```
## 
##   0   1 
##  47 277
```

```
cat("\n")
```

```
# =============================================================================
# MODEL 1: FIRST CHOICE AGREEMENT
# =============================================================================

cat("\n=== FITTING MODEL 1: FIRST CHOICE AGREEMENT ===\n")
```

```
## 
## === FITTING MODEL 1: FIRST CHOICE AGREEMENT ===
```

```
model1_final <- glmer(
  agrees_with_1st_num ~ consensus_strength + question_complexity + source + 
    (1 | question_id),
  data = analysis_dataset,
  family = binomial(link = "logit"),
  control = glmerControl(optimizer = "bobyqa")
)

# Verify reference categories for Model 1
cat("\nModel 1 Reference Categories:\n")
```

```
## 
## Model 1 Reference Categories:
```

```
cat("Question Complexity:", levels(analysis_dataset$question_complexity)[1], "\n")
```

```
## Question Complexity: Binary (2 options)
```

```
cat("Source:", levels(factor(analysis_dataset$source))[1], "\n")
```

```
## Source: ChatGPT
```

```
# Display model coefficients to verify names
cat("\nModel 1 Coefficient Names:\n")
```

```
## 
## Model 1 Coefficient Names:
```

```
print(names(fixef(model1_final)))
```

```
## [1] "(Intercept)"                            
## [2] "consensus_strength"                     
## [3] "question_complexitySimple (3 options)"  
## [4] "question_complexityModerate (4 options)"
## [5] "question_complexityComplex (5+ options)"
## [6] "sourceClaude"                           
## [7] "sourceDeepseek"                         
## [8] "sourceGemini"
```

```
summary(model1_final)
```

```
## Generalized linear mixed model fit by maximum likelihood (Laplace
##   Approximation) [glmerMod]
##  Family: binomial  ( logit )
## Formula: agrees_with_1st_num ~ consensus_strength + question_complexity +  
##     source + (1 | question_id)
##    Data: analysis_dataset
## Control: glmerControl(optimizer = "bobyqa")
## 
##       AIC       BIC    logLik -2*log(L)  df.resid 
##     399.5     433.5    -190.7     381.5       315 
## 
## Scaled residuals: 
##     Min      1Q  Median      3Q     Max 
## -2.7685 -0.6501  0.3638  0.5561  1.7671 
## 
## Random effects:
##  Groups      Name        Variance Std.Dev.
##  question_id (Intercept) 1.428    1.195   
## Number of obs: 324, groups:  question_id, 81
## 
## Fixed effects:
##                                           Estimate Std. Error z value Pr(>|z|)
## (Intercept)                             -9.358e-01  6.645e-01  -1.408   0.1590
## consensus_strength                       4.157e-02  1.050e-02   3.960 7.49e-05
## question_complexitySimple (3 options)    2.590e-02  6.676e-01   0.039   0.9691
## question_complexityModerate (4 options) -6.792e-02  5.489e-01  -0.124   0.9015
## question_complexityComplex (5+ options) -1.111e+00  6.216e-01  -1.788   0.0738
## sourceClaude                            -5.567e-06  3.840e-01   0.000   1.0000
## sourceDeepseek                          -2.195e-01  3.831e-01  -0.573   0.5667
## sourceGemini                             3.765e-01  3.898e-01   0.966   0.3340
##                                            
## (Intercept)                                
## consensus_strength                      ***
## question_complexitySimple (3 options)      
## question_complexityModerate (4 options)    
## question_complexityComplex (5+ options) .  
## sourceClaude                               
## sourceDeepseek                             
## sourceGemini                               
## ---
## Signif. codes:  0 '***' 0.001 '**' 0.01 '*' 0.05 '.' 0.1 ' ' 1
## 
## Correlation of Fixed Effects:
##             (Intr) cnsns_ q_S(3o q_M(4o q_C(5o srcCld srcDps
## cnsnss_strn -0.620                                          
## qstn_cS(3o) -0.469 -0.026                                   
## qstn_cM(4o) -0.629  0.064  0.585                            
## qstn_C(5+o) -0.521 -0.009  0.519  0.629                     
## sourceClaud -0.289  0.000  0.000  0.000  0.000              
## sourceDepsk -0.281 -0.021  0.000  0.000  0.009  0.501       
## sourceGemin -0.300  0.036  0.000  0.001 -0.015  0.493  0.490
```

```
# =============================================================================
# MODEL 2: TOP 2 AGREEMENT (excluding binary questions)
# =============================================================================

cat("\n=== FITTING MODEL 2: TOP 2 AGREEMENT ===\n")
```

```
## 
## === FITTING MODEL 2: TOP 2 AGREEMENT ===
```

```
# CRITICAL: Create filtered dataset and drop unused factor levels
model2_data <- analysis_dataset %>% 
  filter(question_complexity != "Binary (2 options)") %>%
  mutate(
    question_complexity = droplevels(question_complexity),
    source = droplevels(factor(source))
  )

# Verify reference categories for Model 2
cat("\nModel 2 Reference Categories (after filtering):\n")
```

```
## 
## Model 2 Reference Categories (after filtering):
```

```
cat("Question Complexity:", levels(model2_data$question_complexity)[1], "\n")
```

```
## Question Complexity: Simple (3 options)
```

```
cat("Source:", levels(model2_data$source)[1], "\n")
```

```
## Source: ChatGPT
```

```
cat("N observations:", nrow(model2_data), "\n")
```

```
## N observations: 264
```

```
cat("N questions:", n_distinct(model2_data$question_id), "\n\n")
```

```
## N questions: 66
```

```
model2_final <- glmer(
  agrees_with_top2_num ~ consensus_strength + question_complexity + source + 
    (1 | question_id),
  data = model2_data,
  family = binomial(link = "logit"),
  control = glmerControl(optimizer = "bobyqa")
)

# Display model coefficients to verify names
cat("\nModel 2 Coefficient Names:\n")
```

```
## 
## Model 2 Coefficient Names:
```

```
print(names(fixef(model2_final)))
```

```
## [1] "(Intercept)"                            
## [2] "consensus_strength"                     
## [3] "question_complexityModerate (4 options)"
## [4] "question_complexityComplex (5+ options)"
## [5] "sourceClaude"                           
## [6] "sourceDeepseek"                         
## [7] "sourceGemini"
```

```
summary(model2_final)
```

```
## Generalized linear mixed model fit by maximum likelihood (Laplace
##   Approximation) [glmerMod]
##  Family: binomial  ( logit )
## Formula: agrees_with_top2_num ~ consensus_strength + question_complexity +  
##     source + (1 | question_id)
##    Data: model2_data
## Control: glmerControl(optimizer = "bobyqa")
## 
##       AIC       BIC    logLik -2*log(L)  df.resid 
##     216.0     244.6    -100.0     200.0       256 
## 
## Scaled residuals: 
##     Min      1Q  Median      3Q     Max 
## -5.8041  0.1314  0.2670  0.4332  1.3340 
## 
## Random effects:
##  Groups      Name        Variance Std.Dev.
##  question_id (Intercept) 0.2334   0.4832  
## Number of obs: 264, groups:  question_id, 66
## 
## Fixed effects:
##                                         Estimate Std. Error z value Pr(>|z|)
## (Intercept)                              0.73192    0.69838   1.048   0.2946
## consensus_strength                       0.06215    0.01430   4.346 1.39e-05
## question_complexityModerate (4 options) -0.18511    0.57609  -0.321   0.7480
## question_complexityComplex (5+ options) -1.42715    0.60348  -2.365   0.0180
## sourceClaude                            -0.61716    0.56375  -1.095   0.2736
## sourceDeepseek                          -0.74561    0.55818  -1.336   0.1816
## sourceGemini                            -1.09398    0.54782  -1.997   0.0458
##                                            
## (Intercept)                                
## consensus_strength                      ***
## question_complexityModerate (4 options)    
## question_complexityComplex (5+ options) *  
## sourceClaude                               
## sourceDeepseek                             
## sourceGemini                            *  
## ---
## Signif. codes:  0 '***' 0.001 '**' 0.01 '*' 0.05 '.' 0.1 ' ' 1
## 
## Correlation of Fixed Effects:
##             (Intr) cnsns_ q_M(4o q_C(5o srcCld srcDps
## cnsnss_strn -0.419                                   
## qstn_cM(4o) -0.486 -0.184                            
## qstn_C(5+o) -0.465 -0.263  0.725                     
## sourceClaud -0.461 -0.044 -0.006  0.025              
## sourceDepsk -0.465 -0.055 -0.007  0.031  0.592       
## sourceGemin -0.468 -0.089 -0.010  0.049  0.608  0.618
```

```
# =============================================================================
# VISUALIZATIONS
# =============================================================================

cat("\n=== GENERATING VISUALIZATIONS ===\n")
```

```
## 
## === GENERATING VISUALIZATIONS ===
```

```
# 1. Effect of consensus strength (main finding) - Model 1
pred_consensus1 <- ggpredict(model1_final, terms = "consensus_strength [all]")
plot_consensus1 <- plot(pred_consensus1) +
  labs(
    title = "Effect of Consensus Strength on First Choice Agreement",
    x = "Normalized Consensus Strength",
    y = "Predicted Probability of First Choice Agreement",
    caption = "Adjusted for question complexity and LLM source"
  ) +
  theme_minimal()

print(plot_consensus1)
```

```
# Effect of consensus strength - Model 2
pred_consensus2 <- ggpredict(model2_final, terms = "consensus_strength [all]")
plot_consensus2 <- plot(pred_consensus2) +
  labs(
    title = "Effect of Consensus Strength on Top 2 Agreement",
    x = "Normalized Consensus Strength",
    y = "Predicted Probability of Top 2 Agreement",
    caption = "Adjusted for question complexity and LLM source (excluding binary questions)"
  ) +
  theme_minimal()

print(plot_consensus2)
```

```
# 2. Predicted probabilities by LLM (marginal means) - Model 1
emm1 <- emmeans(model1_final, ~ source, type = "response")
plot_llm1 <- as.data.frame(emm1) %>%
  ggplot(aes(x = source, y = prob)) +
  geom_point(size = 3) +
  geom_errorbar(aes(ymin = asymp.LCL, ymax = asymp.UCL), width = 0.2) +
  labs(
    title = "Predicted First Choice Agreement by LLM",
    subtitle = "Adjusted for consensus strength and question complexity",
    x = "LLM",
    y = "Predicted Probability"
  ) +
  theme_minimal()

print(plot_llm1)
```

```
# Predicted probabilities by LLM - Model 2
emm2 <- emmeans(model2_final, ~ source, type = "response")
plot_llm2 <- as.data.frame(emm2) %>%
  ggplot(aes(x = source, y = prob)) +
  geom_point(size = 3) +
  geom_errorbar(aes(ymin = asymp.LCL, ymax = asymp.UCL), width = 0.2) +
  labs(
    title = "Predicted Top 2 Agreement by LLM",
    subtitle = "Adjusted for consensus strength and question complexity (excluding binary)",
    x = "LLM",
    y = "Predicted Probability"
  ) +
  theme_minimal()

print(plot_llm2)
```

```
# 3. Predicted probabilities by question complexity - Model 1
emm_complex1 <- emmeans(model1_final, ~ question_complexity, type = "response")
plot_complex1 <- as.data.frame(emm_complex1) %>%
  ggplot(aes(x = question_complexity, y = prob)) +
  geom_point(size = 3) +
  geom_errorbar(aes(ymin = asymp.LCL, ymax = asymp.UCL), width = 0.2) +
  labs(
    title = "Predicted First Choice Agreement by Question Complexity",
    x = "Question Complexity",
    y = "Predicted Probability"
  ) +
  theme_minimal() +
  theme(axis.text.x = element_text(angle = 45, hjust = 1))

print(plot_complex1)
```

```
# Predicted probabilities by question complexity - Model 2
emm_complex2 <- emmeans(model2_final, ~ question_complexity, type = "response")
plot_complex2 <- as.data.frame(emm_complex2) %>%
  ggplot(aes(x = question_complexity, y = prob)) +
  geom_point(size = 3) +
  geom_errorbar(aes(ymin = asymp.LCL, ymax = asymp.UCL), width = 0.2) +
  labs(
    title = "Predicted Top 2 Agreement by Question Complexity",
    subtitle = "Excluding binary questions",
    x = "Question Complexity",
    y = "Predicted Probability"
  ) +
  theme_minimal() +
  theme(axis.text.x = element_text(angle = 45, hjust = 1))

print(plot_complex2)
```

```
# =============================================================================
# PUBLICATION-READY RESULTS TABLES
# =============================================================================

cat("\n=== CREATING PUBLICATION TABLES ===\n")
```

```
## 
## === CREATING PUBLICATION TABLES ===
```

```
# Table for Model 1 (reference = Binary for complexity, ChatGPT for source)
tab_model(
  model1_final,
  show.icc = TRUE,
  show.r2 = TRUE,
  show.aic = TRUE,
  dv.labels = c("First Choice Agreement"),
  pred.labels = c(
    "(Intercept)" = "Intercept",
    "consensus_strength" = "Consensus Strength (per unit)",
    "question_complexitySimple (3 options)" = "Simple (3 options) vs Binary",
    "question_complexityModerate (4 options)" = "Moderate (4 options) vs Binary",
    "question_complexityComplex (5+ options)" = "Complex (5+ options) vs Binary",
    "sourceClaude" = "Claude vs ChatGPT",
    "sourceDeepseek" = "Deepseek vs ChatGPT",
    "sourceGemini" = "Gemini vs ChatGPT"
  ),
  string.pred = "Predictor",
  string.ci = "95% CI",
  string.p = "p-value",
  title = "Table 1: Factors Associated with First Choice Agreement"
)
```

Table 1: Factors Associated with First Choice Agreement

|  | First Choice Agreement | | |
| Predictor | Odds Ratios | 95% CI | p-value |
| Intercept | 0.39 | 0.11 – 1.44 | 0.159 |
| Consensus Strength (per unit) | 1.04 | 1.02 – 1.06 | **<0.001** |
| Simple (3 options) vs Binary | 1.03 | 0.28 – 3.80 | 0.969 |
| Moderate (4 options) vs Binary | 0.93 | 0.32 – 2.74 | 0.902 |
| Complex (5+ options) vs Binary | 0.33 | 0.10 – 1.11 | 0.074 |
| Claude vs ChatGPT | 1.00 | 0.47 – 2.12 | 1.000 |
| Deepseek vs ChatGPT | 0.80 | 0.38 – 1.70 | 0.567 |
| Gemini vs ChatGPT | 1.46 | 0.68 – 3.13 | 0.334 |
| Random Effects | | | |
| σ^2^ | 3.29 | | |
| τ_00_ _question\_id_ | 1.43 | | || ICC | 0.30 | | || N _question\_id_ | 81 | | || Observations | 324 | | |
| Marginal R^2^ / Conditional R^2^ | 0.166 / 0.418 | | |
| AIC | 399.469 | | |

```
# Table for Model 2 (reference = Simple for complexity, ChatGPT for source)
# NOTE: Only Moderate and Complex appear because Binary is excluded
tab_model(
  model2_final,
  show.icc = TRUE,
  show.r2 = TRUE,
  show.aic = TRUE,
  dv.labels = c("Top 2 Agreement"),
  pred.labels = c(
    "(Intercept)" = "Intercept",
    "consensus_strength" = "Consensus Strength (per unit)",
    "question_complexityModerate (4 options)" = "Moderate (4 options) vs Simple",
    "question_complexityComplex (5+ options)" = "Complex (5+ options) vs Simple",
    "sourceClaude" = "Claude vs ChatGPT",
    "sourceDeepseek" = "Deepseek vs ChatGPT",
    "sourceGemini" = "Gemini vs ChatGPT"
  ),
  string.pred = "Predictor",
  string.ci = "95% CI",
  string.p = "p-value",
  title = "Table 2: Factors Associated with Top 2 Agreement (excluding binary questions)"
)
```

Table 2: Factors Associated with Top 2 Agreement (excluding binary
questions)

|  | Top 2 Agreement | | |
| Predictor | Odds Ratios | 95% CI | p-value |
| Intercept | 2.08 | 0.53 – 8.17 | 0.295 |
| Consensus Strength (per unit) | 1.06 | 1.03 – 1.09 | **<0.001** |
| Moderate (4 options) vs Simple | 0.83 | 0.27 – 2.57 | 0.748 |
| Complex (5+ options) vs Simple | 0.24 | 0.07 – 0.78 | **0.018** |
| Claude vs ChatGPT | 0.54 | 0.18 – 1.63 | 0.274 |
| Deepseek vs ChatGPT | 0.47 | 0.16 – 1.42 | 0.182 |
| Gemini vs ChatGPT | 0.33 | 0.11 – 0.98 | **0.046** |
| Random Effects | | | |
| σ^2^ | 3.29 | | |
| τ_00_ _question\_id_ | 0.23 | | || ICC | 0.07 | | || N _question\_id_ | 66 | | || Observations | 264 | | |
| Marginal R^2^ / Conditional R^2^ | 0.322 / 0.367 | | |
| AIC | 215.988 | | |

```
# =============================================================================
# EXTRACT ODDS RATIOS WITH CONFIDENCE INTERVALS
# =============================================================================

cat("\n=== EXTRACTING ODDS RATIOS ===\n")
```

```
## 
## === EXTRACTING ODDS RATIOS ===
```

```
# Model 1 - Extract and format with proper labels
tidy_model1 <- tidy(model1_final, conf.int = TRUE, exponentiate = TRUE) %>%
  filter(effect == "fixed") %>%
  mutate(
    Parameter = case_when(
      term == "(Intercept)" ~ "Intercept",
      term == "consensus_strength" ~ "Consensus Strength (per unit)",
      term == "question_complexitySimple (3 options)" ~ "Simple vs Binary",
      term == "question_complexityModerate (4 options)" ~ "Moderate vs Binary",
      term == "question_complexityComplex (5+ options)" ~ "Complex vs Binary",
      term == "sourceClaude" ~ "Claude vs ChatGPT",
      term == "sourceDeepseek" ~ "Deepseek vs ChatGPT",
      term == "sourceGemini" ~ "Gemini vs ChatGPT",
      TRUE ~ term
    ),
    OR_CI = sprintf("%.2f [%.2f-%.2f]", estimate, conf.low, conf.high),
    p_formatted = ifelse(p.value < 0.001, "<0.001", sprintf("%.3f", p.value))
  ) %>%
  dplyr::select(Parameter, OR = estimate, OR_CI, p_formatted)

cat("\n=== Model 1: Odds Ratios (First Choice Agreement) ===\n")
```

```
## 
## === Model 1: Odds Ratios (First Choice Agreement) ===
```

```
print(tidy_model1, n = Inf)
```

```
## # A tibble: 8 × 4
##   Parameter                        OR OR_CI            p_formatted
##   <chr>                         <dbl> <chr>            <chr>      
## 1 Intercept                     0.392 0.39 [0.11-1.44] 0.159      
## 2 Consensus Strength (per unit) 1.04  1.04 [1.02-1.06] <0.001     
## 3 Simple vs Binary              1.03  1.03 [0.28-3.80] 0.969      
## 4 Moderate vs Binary            0.934 0.93 [0.32-2.74] 0.902      
## 5 Complex vs Binary             0.329 0.33 [0.10-1.11] 0.074      
## 6 Claude vs ChatGPT             1.00  1.00 [0.47-2.12] 1.000      
## 7 Deepseek vs ChatGPT           0.803 0.80 [0.38-1.70] 0.567      
## 8 Gemini vs ChatGPT             1.46  1.46 [0.68-3.13] 0.334
```

```
# Model 2 - Extract and format with proper labels
tidy_model2 <- tidy(model2_final, conf.int = TRUE, exponentiate = TRUE) %>%
  filter(effect == "fixed") %>%
  mutate(
    Parameter = case_when(
      term == "(Intercept)" ~ "Intercept",
      term == "consensus_strength" ~ "Consensus Strength (per unit)",
      term == "question_complexityModerate (4 options)" ~ "Moderate vs Simple",
      term == "question_complexityComplex (5+ options)" ~ "Complex vs Simple",
      term == "sourceClaude" ~ "Claude vs ChatGPT",
      term == "sourceDeepseek" ~ "Deepseek vs ChatGPT",
      term == "sourceGemini" ~ "Gemini vs ChatGPT",
      TRUE ~ term
    ),
    OR_CI = sprintf("%.2f [%.2f-%.2f]", estimate, conf.low, conf.high),
    p_formatted = ifelse(p.value < 0.001, "<0.001", sprintf("%.3f", p.value))
  ) %>%
  dplyr::select(Parameter, OR = estimate, OR_CI, p_formatted)

cat("\n=== Model 2: Odds Ratios (Top 2 Agreement, excluding binary) ===\n")
```

```
## 
## === Model 2: Odds Ratios (Top 2 Agreement, excluding binary) ===
```

```
print(tidy_model2, n = Inf)
```

```
## # A tibble: 7 × 4
##   Parameter                        OR OR_CI            p_formatted
##   <chr>                         <dbl> <chr>            <chr>      
## 1 Intercept                     2.08  2.08 [0.53-8.17] 0.295      
## 2 Consensus Strength (per unit) 1.06  1.06 [1.03-1.09] <0.001     
## 3 Moderate vs Simple            0.831 0.83 [0.27-2.57] 0.748      
## 4 Complex vs Simple             0.240 0.24 [0.07-0.78] 0.018      
## 5 Claude vs ChatGPT             0.539 0.54 [0.18-1.63] 0.274      
## 6 Deepseek vs ChatGPT           0.474 0.47 [0.16-1.42] 0.182      
## 7 Gemini vs ChatGPT             0.335 0.33 [0.11-0.98] 0.046
```

```
# =============================================================================
# CREATE FORMATTED COMPARISON TABLE
# =============================================================================

# Combine results for easy comparison
comparison_table <- bind_rows(
  tidy_model1 %>% mutate(Model = "Model 1: First Choice"),
  tidy_model2 %>% mutate(Model = "Model 2: Top 2 (non-binary)")
) %>%
  dplyr::select(Model, Parameter, OR, OR_CI, p_formatted)

cat("\n=== COMBINED RESULTS TABLE ===\n")
```

```
## 
## === COMBINED RESULTS TABLE ===
```

```
comparison_table %>%
  kable(
    caption = "Comparison of Regression Models",
    col.names = c("Model", "Parameter", "Odds Ratio", "95% CI", "P-value"),
    digits = 2
  ) %>%
  kable_styling(bootstrap_options = c("striped", "hover")) %>%
  column_spec(5, bold = ifelse(comparison_table$p_formatted == "<0.001" | 
                               as.numeric(comparison_table$p_formatted) < 0.05, 
                               TRUE, FALSE))
```

Comparison of Regression Models

| Model | Parameter | Odds Ratio | 95% CI | P-value |
| --- | --- | --- | --- | --- |
| Model 1: First Choice | Intercept | 0.39 | 0.39 [0.11-1.44] | 0.159 |
| Model 1: First Choice | Consensus Strength (per unit) | 1.04 | 1.04 [1.02-1.06] | <0.001 |
| Model 1: First Choice | Simple vs Binary | 1.03 | 1.03 [0.28-3.80] | 0.969 |
| Model 1: First Choice | Moderate vs Binary | 0.93 | 0.93 [0.32-2.74] | 0.902 |
| Model 1: First Choice | Complex vs Binary | 0.33 | 0.33 [0.10-1.11] | 0.074 |
| Model 1: First Choice | Claude vs ChatGPT | 1.00 | 1.00 [0.47-2.12] | 1.000 |
| Model 1: First Choice | Deepseek vs ChatGPT | 0.80 | 0.80 [0.38-1.70] | 0.567 |
| Model 1: First Choice | Gemini vs ChatGPT | 1.46 | 1.46 [0.68-3.13] | 0.334 |
| Model 2: Top 2 (non-binary) | Intercept | 2.08 | 2.08 [0.53-8.17] | 0.295 |
| Model 2: Top 2 (non-binary) | Consensus Strength (per unit) | 1.06 | 1.06 [1.03-1.09] | <0.001 |
| Model 2: Top 2 (non-binary) | Moderate vs Simple | 0.83 | 0.83 [0.27-2.57] | 0.748 |
| Model 2: Top 2 (non-binary) | Complex vs Simple | 0.24 | 0.24 [0.07-0.78] | 0.018 |
| Model 2: Top 2 (non-binary) | Claude vs ChatGPT | 0.54 | 0.54 [0.18-1.63] | 0.274 |
| Model 2: Top 2 (non-binary) | Deepseek vs ChatGPT | 0.47 | 0.47 [0.16-1.42] | 0.182 |
| Model 2: Top 2 (non-binary) | Gemini vs ChatGPT | 0.33 | 0.33 [0.11-0.98] | 0.046 |

```
# =============================================================================
# SAVE OUTPUTS
# =============================================================================

cat("\n=== SAVING RESULTS ===\n")
```

```
## 
## === SAVING RESULTS ===
```

```
# Save model objects
saveRDS(model1_final, "output/model1_first_choice.rds")
saveRDS(model2_final, "output/model2_top2_choice.rds")

# Save tidy results
write.csv(tidy_model1, "output/model1_coefficients.csv", row.names = FALSE)
write.csv(tidy_model2, "output/model2_coefficients.csv", row.names = FALSE)
write.csv(comparison_table, "output/models_comparison.csv", row.names = FALSE)

# Save plots
ggsave("output/plot_consensus_model1.png", plot_consensus1, width = 10, height = 6, dpi = 300)
ggsave("output/plot_consensus_model2.png", plot_consensus2, width = 10, height = 6, dpi = 300)
ggsave("output/plot_llm_model1.png", plot_llm1, width = 8, height = 6, dpi = 300)
ggsave("output/plot_llm_model2.png", plot_llm2, width = 8, height = 6, dpi = 300)
ggsave("output/plot_complexity_model1.png", plot_complex1, width = 10, height = 6, dpi = 300)
ggsave("output/plot_complexity_model2.png", plot_complex2, width = 10, height = 6, dpi = 300)

cat("\n=== REGRESSION ANALYSIS COMPLETE ===\n")
```

```
## 
## === REGRESSION ANALYSIS COMPLETE ===
```

```
cat("Models fitted successfully\n")
```

```
## Models fitted successfully
```

```
cat("Tables and plots generated\n")
```

```
## Tables and plots generated
```

```
cat("Results saved to output/ directory\n")
```

```
## Results saved to output/ directory
```

### 2.4.1 Boostrap

```
# =============================================================================
# BOOTSTRAP VALIDATION OF MIXED-EFFECTS MODELS
# =============================================================================

library(lme4)
library(broom.mixed)
library(tidyverse)
library(kableExtra)
library(parallel)

cat("Starting bootstrap validation...\n")
```

```
## Starting bootstrap validation...
```

```
cat("This may take 30-60 minutes depending on your system\n\n")
```

```
## This may take 30-60 minutes depending on your system
```

```
# Set seed for reproducibility
set.seed(123)

# Detect number of cores for parallel processing
n_cores <- max(1, detectCores() - 1)
cat("Using", n_cores, "cores for parallel processing\n\n")
```

```
## Using 9 cores for parallel processing
```

```
# =============================================================================
# ENSURE MODEL 2 DATA IS PROPERLY PREPARED
# =============================================================================

# CRITICAL: Make sure model2_data has dropped factor levels
model2_data <- analysis_dataset %>% 
  filter(question_complexity != "Binary (2 options)") %>%
  mutate(
    question_complexity = droplevels(question_complexity),
    source = droplevels(factor(source))
  )

cat("Model 2 data prepared:\n")
```

```
## Model 2 data prepared:
```

```
cat("Reference level for complexity:", levels(model2_data$question_complexity)[1], "\n")
```

```
## Reference level for complexity: Simple (3 options)
```

```
cat("N observations:", nrow(model2_data), "\n\n")
```

```
## N observations: 264
```

```
# =============================================================================
# PARAMETRIC BOOTSTRAP FOR MODEL 1
# =============================================================================

cat("Running parametric bootstrap for Model 1 (First Choice Agreement)...\n")
```

```
## Running parametric bootstrap for Model 1 (First Choice Agreement)...
```

```
start_time_m1 <- Sys.time()

boot_model1 <- bootMer(
  model1_final,
  FUN = function(x) fixef(x),
  nsim = 5000,
  parallel = "multicore",
  ncpus = n_cores,
  .progress = "txt"
)

end_time_m1 <- Sys.time()
cat("Model 1 bootstrap completed in", 
    round(difftime(end_time_m1, start_time_m1, units = "mins"), 1), "minutes\n\n")
```

```
## Model 1 bootstrap completed in 9.8 minutes
```

```
# =============================================================================
# PARAMETRIC BOOTSTRAP FOR MODEL 2
# =============================================================================

cat("Running parametric bootstrap for Model 2 (Top 2 Agreement)...\n")
```

```
## Running parametric bootstrap for Model 2 (Top 2 Agreement)...
```

```
start_time_m2 <- Sys.time()

boot_model2 <- bootMer(
  model2_final,
  FUN = function(x) fixef(x),
  nsim = 5000,
  parallel = "multicore",
  ncpus = n_cores,
  .progress = "txt"
)

end_time_m2 <- Sys.time()
cat("Model 2 bootstrap completed in", 
    round(difftime(end_time_m2, start_time_m2, units = "mins"), 1), "minutes\n\n")
```

```
## Model 2 bootstrap completed in 7 minutes
```

```
# =============================================================================
# EXTRACT MODEL-BASED CONFIDENCE INTERVALS - WITH MODEL-SPECIFIC LABELING
# =============================================================================

extract_model_ci <- function(model, model_name, model_number) {
  
  # Get coefficients with Wald CIs
  coef_table <- as.data.frame(summary(model)$coefficients)
  coef_table$parameter <- rownames(coef_table)
  
  # Calculate Wald CIs on log-odds scale
  coef_table$ci_lower <- coef_table$Estimate - 1.96 * coef_table$`Std. Error`
  coef_table$ci_upper <- coef_table$Estimate + 1.96 * coef_table$`Std. Error`
  
  # Exponentiate to get ORs and CIs
  coef_table$OR <- exp(coef_table$Estimate)
  coef_table$OR_lower <- exp(coef_table$ci_lower)
  coef_table$OR_upper <- exp(coef_table$ci_upper)
  
  # Format CI string
  coef_table$CI_Model <- sprintf("%.2f-%.2f", coef_table$OR_lower, coef_table$OR_upper)
  
  # Clean parameter names - MODEL-SPECIFIC
  if (model_number == 1) {
    # Model 1: Binary is reference for complexity
    coef_table$Parameter <- case_when(
      coef_table$parameter == "(Intercept)" ~ "Intercept",
      coef_table$parameter == "consensus_strength" ~ "Consensus Strength (per unit)",
      coef_table$parameter == "question_complexitySimple (3 options)" ~ "Simple vs Binary",
      coef_table$parameter == "question_complexityModerate (4 options)" ~ "Moderate vs Binary",
      coef_table$parameter == "question_complexityComplex (5+ options)" ~ "Complex vs Binary",
      coef_table$parameter == "sourceClaude" ~ "Claude vs ChatGPT",
      coef_table$parameter == "sourceDeepseek" ~ "Deepseek vs ChatGPT",
      coef_table$parameter == "sourceGemini" ~ "Gemini vs ChatGPT",
      TRUE ~ coef_table$parameter
    )
  } else if (model_number == 2) {
    # Model 2: Simple is reference for complexity (no binary questions)
    coef_table$Parameter <- case_when(
      coef_table$parameter == "(Intercept)" ~ "Intercept",
      coef_table$parameter == "consensus_strength" ~ "Consensus Strength (per unit)",
      coef_table$parameter == "question_complexityModerate (4 options)" ~ "Moderate vs Simple",
      coef_table$parameter == "question_complexityComplex (5+ options)" ~ "Complex vs Simple",
      coef_table$parameter == "sourceClaude" ~ "Claude vs ChatGPT",
      coef_table$parameter == "sourceDeepseek" ~ "Deepseek vs ChatGPT",
      coef_table$parameter == "sourceGemini" ~ "Gemini vs ChatGPT",
      TRUE ~ coef_table$parameter
    )
  }
  
  # Select relevant columns
  result <- coef_table %>%
    dplyr::select(Parameter, OR, OR_lower, OR_upper, CI_Model, `Pr(>|z|)`) %>%
    mutate(Model = model_name)
  
  return(result)
}

# Extract CIs for both models with model_number parameter
model1_ci <- extract_model_ci(model1_final, "Model 1", model_number = 1)
model2_ci <- extract_model_ci(model2_final, "Model 2", model_number = 2)

# =============================================================================
# EXTRACT BOOTSTRAP CONFIDENCE INTERVALS - WITH MODEL-SPECIFIC LABELING
# =============================================================================

extract_boot_ci <- function(boot_object, model, model_name, model_number) {
  
  # Get bootstrap samples (already on log-odds scale)
  boot_samples <- boot_object$t
  
  # Parameter names
  param_names <- names(fixef(model))
  colnames(boot_samples) <- param_names
  
  # Calculate percentile bootstrap CIs (2.5% and 97.5%)
  boot_ci_logodds <- apply(boot_samples, 2, function(x) {
    quantile(x, probs = c(0.025, 0.975), na.rm = TRUE)
  })
  
  # Exponentiate to get ORs
  boot_or <- apply(boot_samples, 2, function(x) exp(median(x, na.rm = TRUE)))
  boot_ci_or <- exp(boot_ci_logodds)
  
  # Create data frame
  boot_df <- data.frame(
    parameter = param_names,
    OR_Bootstrap = boot_or,
    Boot_lower = boot_ci_or[1, ],
    Boot_upper = boot_ci_or[2, ],
    stringsAsFactors = FALSE
  )
  
  # Format CI string
  boot_df$CI_Bootstrap <- sprintf("%.2f-%.2f", boot_df$Boot_lower, boot_df$Boot_upper)
  
  # Clean parameter names - MODEL-SPECIFIC
  if (model_number == 1) {
    # Model 1: Binary is reference
    boot_df$Parameter <- case_when(
      boot_df$parameter == "(Intercept)" ~ "Intercept",
      boot_df$parameter == "consensus_strength" ~ "Consensus Strength (per unit)",
      boot_df$parameter == "question_complexitySimple (3 options)" ~ "Simple vs Binary",
      boot_df$parameter == "question_complexityModerate (4 options)" ~ "Moderate vs Binary",
      boot_df$parameter == "question_complexityComplex (5+ options)" ~ "Complex vs Binary",
      boot_df$parameter == "sourceClaude" ~ "Claude vs ChatGPT",
      boot_df$parameter == "sourceDeepseek" ~ "Deepseek vs ChatGPT",
      boot_df$parameter == "sourceGemini" ~ "Gemini vs ChatGPT",
      TRUE ~ boot_df$parameter
    )
  } else if (model_number == 2) {
    # Model 2: Simple is reference (no binary questions)
    boot_df$Parameter <- case_when(
      boot_df$parameter == "(Intercept)" ~ "Intercept",
      boot_df$parameter == "consensus_strength" ~ "Consensus Strength (per unit)",
      boot_df$parameter == "question_complexityModerate (4 options)" ~ "Moderate vs Simple",
      boot_df$parameter == "question_complexityComplex (5+ options)" ~ "Complex vs Simple",
      boot_df$parameter == "sourceClaude" ~ "Claude vs ChatGPT",
      boot_df$parameter == "sourceDeepseek" ~ "Deepseek vs ChatGPT",
      boot_df$parameter == "sourceGemini" ~ "Gemini vs ChatGPT",
      TRUE ~ boot_df$parameter
    )
  }
  
  boot_df$Model <- model_name
  
  return(boot_df)
}

# Extract bootstrap CIs with model_number parameter
boot1_ci <- extract_boot_ci(boot_model1, model1_final, "Model 1", model_number = 1)
boot2_ci <- extract_boot_ci(boot_model2, model2_final, "Model 2", model_number = 2)

# =============================================================================
# COMBINE AND COMPARE CIs
# =============================================================================

# Function to assess CI stability
assess_stability <- function(model_lower, model_upper, boot_lower, boot_upper) {
  model_width <- model_upper - model_lower
  boot_width <- boot_upper - boot_lower
  
  # Calculate overlap
  overlap_lower <- max(model_lower, boot_lower)
  overlap_upper <- min(model_upper, boot_upper)
  overlap <- max(0, overlap_upper - overlap_lower)
  
  # Calculate proportion of overlap relative to union
  union_lower <- min(model_lower, boot_lower)
  union_upper <- max(model_upper, boot_upper)
  union_width <- union_upper - union_lower
  
  overlap_prop <- overlap / union_width
  
  # Width ratio
  width_ratio <- boot_width / model_width
  
  # Determine stability
  if (overlap_prop > 0.80 & width_ratio > 0.5 & width_ratio < 2.0) {
    return("Yes")
  } else if (overlap_prop > 0.60) {
    return("Moderate")
  } else {
    return("No")
  }
}

# Create comparison table for Model 1
comparison_model1 <- model1_ci %>%
  left_join(boot1_ci %>% dplyr::select(Parameter, OR_Bootstrap, Boot_lower, Boot_upper, CI_Bootstrap),
            by = "Parameter") %>%
  mutate(
    Stable = mapply(assess_stability, OR_lower, OR_upper, Boot_lower, Boot_upper),
    OR_Model = round(OR, 2),
    OR_Bootstrap = round(OR_Bootstrap, 2),
    P_value = ifelse(`Pr(>|z|)` < 0.001, "<0.001", 
                     sprintf("%.3f", `Pr(>|z|)`))
  ) %>%
  dplyr::select(Parameter, OR_Model, CI_Model, OR_Bootstrap, CI_Bootstrap, Stable, P_value)

# Create comparison table for Model 2
comparison_model2 <- model2_ci %>%
  left_join(boot2_ci %>% dplyr::select(Parameter, OR_Bootstrap, Boot_lower, Boot_upper, CI_Bootstrap),
            by = "Parameter") %>%
  mutate(
    Stable = mapply(assess_stability, OR_lower, OR_upper, Boot_lower, Boot_upper),
    OR_Model = round(OR, 2),
    OR_Bootstrap = round(OR_Bootstrap, 2),
    P_value = ifelse(`Pr(>|z|)` < 0.001, "<0.001", 
                     sprintf("%.3f", `Pr(>|z|)`))
  ) %>%
  dplyr::select(Parameter, OR_Model, CI_Model, OR_Bootstrap, CI_Bootstrap, Stable, P_value)

# =============================================================================
# DISPLAY RESULTS
# =============================================================================

cat("\n=== SUPPLEMENTARY TABLE: MODEL 1 BOOTSTRAP VALIDATION ===\n\n")
```

```
## 
## === SUPPLEMENTARY TABLE: MODEL 1 BOOTSTRAP VALIDATION ===
```

```
cat("Reference categories: Binary (complexity), ChatGPT (source)\n\n")
```

```
## Reference categories: Binary (complexity), ChatGPT (source)
```

```
comparison_model1 %>%
  kable(
    caption = "Supplementary Table: Bootstrap Validation of Model 1 (First Choice Agreement)",
    col.names = c("Parameter", "Model OR", "Model 95% CI", 
                  "Bootstrap OR", "Bootstrap 95% CI", "Stable?", "P-value"),
    align = c("l", "r", "c", "r", "c", "c", "r")
  ) %>%
  kable_styling(bootstrap_options = c("striped", "hover"), full_width = FALSE) %>%
  column_spec(6, bold = TRUE, 
              color = ifelse(comparison_model1$Stable == "Yes", "darkgreen",
                           ifelse(comparison_model1$Stable == "Moderate", "orange", "red")))
```

Supplementary Table: Bootstrap Validation of Model 1 (First Choice
Agreement)

| Parameter | Model OR | Model 95% CI | Bootstrap OR | Bootstrap 95% CI | Stable? | P-value |
| --- | --- | --- | --- | --- | --- | --- |
| Intercept | 0.39 | 0.11-1.44 | 0.39 | 0.10-1.28 | Yes | 0.159 |
| Consensus Strength (per unit) | 1.04 | 1.02-1.06 | 1.04 | 1.02-1.07 | Yes | <0.001 |
| Simple vs Binary | 1.03 | 0.28-3.80 | 1.00 | 0.27-3.78 | Yes | 0.969 |
| Moderate vs Binary | 0.93 | 0.32-2.74 | 0.93 | 0.31-2.63 | Yes | 0.902 |
| Complex vs Binary | 0.33 | 0.10-1.11 | 0.32 | 0.09-1.02 | Yes | 0.074 |
| Claude vs ChatGPT | 1.00 | 0.47-2.12 | 1.00 | 0.46-2.11 | Yes | 1.000 |
| Deepseek vs ChatGPT | 0.80 | 0.38-1.70 | 0.81 | 0.38-1.71 | Yes | 0.567 |
| Gemini vs ChatGPT | 1.46 | 0.68-3.13 | 1.46 | 0.69-3.18 | Yes | 0.334 |

```
cat("\n=== SUPPLEMENTARY TABLE: MODEL 2 BOOTSTRAP VALIDATION ===\n\n")
```

```
## 
## === SUPPLEMENTARY TABLE: MODEL 2 BOOTSTRAP VALIDATION ===
```

```
cat("Reference categories: Simple (complexity), ChatGPT (source)\n")
```

```
## Reference categories: Simple (complexity), ChatGPT (source)
```

```
cat("Note: Binary questions excluded from this model\n\n")
```

```
## Note: Binary questions excluded from this model
```

```
comparison_model2 %>%
  kable(
    caption = "Supplementary Table: Bootstrap Validation of Model 2 (Top 2 Agreement)",
    col.names = c("Parameter", "Model OR", "Model 95% CI", 
                  "Bootstrap OR", "Bootstrap 95% CI", "Stable?", "P-value"),
    align = c("l", "r", "c", "r", "c", "c", "r")
  ) %>%
  kable_styling(bootstrap_options = c("striped", "hover"), full_width = FALSE) %>%
  column_spec(6, bold = TRUE,
              color = ifelse(comparison_model2$Stable == "Yes", "darkgreen",
                           ifelse(comparison_model2$Stable == "Moderate", "orange", "red")))
```

Supplementary Table: Bootstrap Validation of Model 2 (Top 2 Agreement)

| Parameter | Model OR | Model 95% CI | Bootstrap OR | Bootstrap 95% CI | Stable? | P-value |
| --- | --- | --- | --- | --- | --- | --- |
| Intercept | 2.08 | 0.53-8.17 | 2.15 | 0.56-11.52 | Moderate | 0.295 |
| Consensus Strength (per unit) | 1.06 | 1.03-1.09 | 1.07 | 1.04-1.11 | Moderate | <0.001 |
| Moderate vs Simple | 0.83 | 0.27-2.57 | 0.81 | 0.19-2.48 | Yes | 0.748 |
| Complex vs Simple | 0.24 | 0.07-0.78 | 0.22 | 0.05-0.69 | Yes | 0.018 |
| Claude vs ChatGPT | 0.54 | 0.18-1.63 | 0.54 | 0.14-1.63 | Yes | 0.274 |
| Deepseek vs ChatGPT | 0.47 | 0.16-1.42 | 0.46 | 0.13-1.38 | Yes | 0.182 |
| Gemini vs ChatGPT | 0.33 | 0.11-0.98 | 0.33 | 0.08-0.88 | Yes | 0.046 |

```
# =============================================================================
# STABILITY SUMMARY
# =============================================================================

cat("\n=== BOOTSTRAP STABILITY SUMMARY ===\n\n")
```

```
## 
## === BOOTSTRAP STABILITY SUMMARY ===
```

```
stability_summary <- data.frame(
  Model = c("Model 1 (First Choice)", "Model 2 (Top 2, non-binary)"),
  Total_Parameters = c(nrow(comparison_model1), nrow(comparison_model2)),
  Stable = c(
    sum(comparison_model1$Stable == "Yes"),
    sum(comparison_model2$Stable == "Yes")
  ),
  Moderate = c(
    sum(comparison_model1$Stable == "Moderate"),
    sum(comparison_model2$Stable == "Moderate")
  ),
  Unstable = c(
    sum(comparison_model1$Stable == "No"),
    sum(comparison_model2$Stable == "No")
  )
)

stability_summary %>%
  mutate(
    Pct_Stable = round(100 * Stable / Total_Parameters, 1)
  ) %>%
  kable(
    caption = "Bootstrap Stability Summary",
    col.names = c("Model", "Total Parameters", "Stable", "Moderate", "Unstable", "% Stable")
  ) %>%
  kable_styling(bootstrap_options = c("striped", "hover"), full_width = FALSE)
```

Bootstrap Stability Summary

| Model | Total Parameters | Stable | Moderate | Unstable | % Stable |
| --- | --- | --- | --- | --- | --- |
| Model 1 (First Choice) | 8 | 8 | 0 | 0 | 100.0 |
| Model 2 (Top 2, non-binary) | 7 | 5 | 2 | 0 | 71.4 |

```
# =============================================================================
# SAVE RESULTS
# =============================================================================

saveRDS(boot_model1, "output/bootstrap_model1.rds")
saveRDS(boot_model2, "output/bootstrap_model2.rds")
write.csv(comparison_model1, "output/bootstrap_comparison_model1.csv", row.names = FALSE)
write.csv(comparison_model2, "output/bootstrap_comparison_model2.csv", row.names = FALSE)

cat("\n=== Bootstrap results saved to output/ folder ===\n")
```

```
## 
## === Bootstrap results saved to output/ folder ===
```

```
# =============================================================================
# VISUALIZATION: BOOTSTRAP DISTRIBUTIONS - WITH MODEL-SPECIFIC LABELING
# =============================================================================

cat("\nGenerating bootstrap distribution plots...\n")
```

```
## 
## Generating bootstrap distribution plots...
```

```
plot_boot_distributions <- function(boot_object, model, model_name, model_number) {
  
  # Get bootstrap samples
  boot_samples <- as.data.frame(boot_object$t)
  colnames(boot_samples) <- names(fixef(model))
  
  # Original estimates
  original_estimates <- fixef(model)
  
  # Reshape for plotting
  boot_long <- boot_samples %>%
    pivot_longer(everything(), names_to = "Parameter", values_to = "Estimate_LogOdds") %>%
    mutate(Estimate_OR = exp(Estimate_LogOdds))
  
  # Apply model-specific labeling
  if (model_number == 1) {
    boot_long <- boot_long %>%
      mutate(
        Parameter = case_when(
          Parameter == "(Intercept)" ~ "Intercept",
          Parameter == "consensus_strength" ~ "Consensus Strength",
          Parameter == "question_complexitySimple (3 options)" ~ "Simple vs Binary",
          Parameter == "question_complexityModerate (4 options)" ~ "Moderate vs Binary",
          Parameter == "question_complexityComplex (5+ options)" ~ "Complex vs Binary",
          Parameter == "sourceClaude" ~ "Claude vs ChatGPT",
          Parameter == "sourceDeepseek" ~ "Deepseek vs ChatGPT",
          Parameter == "sourceGemini" ~ "Gemini vs ChatGPT",
          TRUE ~ Parameter
        )
      )
  } else if (model_number == 2) {
    boot_long <- boot_long %>%
      mutate(
        Parameter = case_when(
          Parameter == "(Intercept)" ~ "Intercept",
          Parameter == "consensus_strength" ~ "Consensus Strength",
          Parameter == "question_complexityModerate (4 options)" ~ "Moderate vs Simple",
          Parameter == "question_complexityComplex (5+ options)" ~ "Complex vs Simple",
          Parameter == "sourceClaude" ~ "Claude vs ChatGPT",
          Parameter == "sourceDeepseek" ~ "Deepseek vs ChatGPT",
          Parameter == "sourceGemini" ~ "Gemini vs ChatGPT",
          TRUE ~ Parameter
        )
      )
  }
  
  # Original estimates for vertical lines (apply same labeling)
  original_df <- data.frame(
    Parameter = names(original_estimates),
    Original_OR = exp(original_estimates)
  )
  
  if (model_number == 1) {
    original_df <- original_df %>%
      mutate(
        Parameter = case_when(
          Parameter == "(Intercept)" ~ "Intercept",
          Parameter == "consensus_strength" ~ "Consensus Strength",
          Parameter == "question_complexitySimple (3 options)" ~ "Simple vs Binary",
          Parameter == "question_complexityModerate (4 options)" ~ "Moderate vs Binary",
          Parameter == "question_complexityComplex (5+ options)" ~ "Complex vs Binary",
          Parameter == "sourceClaude" ~ "Claude vs ChatGPT",
          Parameter == "sourceDeepseek" ~ "Deepseek vs ChatGPT",
          Parameter == "sourceGemini" ~ "Gemini vs ChatGPT",
          TRUE ~ Parameter
        )
      )
  } else if (model_number == 2) {
    original_df <- original_df %>%
      mutate(
        Parameter = case_when(
          Parameter == "(Intercept)" ~ "Intercept",
          Parameter == "consensus_strength" ~ "Consensus Strength",
          Parameter == "question_complexityModerate (4 options)" ~ "Moderate vs Simple",
          Parameter == "question_complexityComplex (5+ options)" ~ "Complex vs Simple",
          Parameter == "sourceClaude" ~ "Claude vs ChatGPT",
          Parameter == "sourceDeepseek" ~ "Deepseek vs ChatGPT",
          Parameter == "sourceGemini" ~ "Gemini vs ChatGPT",
          TRUE ~ Parameter
        )
      )
  }
  
  # Plot
  p <- ggplot(boot_long, aes(x = Estimate_OR)) +
    geom_histogram(bins = 50, fill = "steelblue", alpha = 0.7) +
    geom_vline(data = original_df, aes(xintercept = Original_OR),
               color = "red", linetype = "dashed", size = 1) +
    facet_wrap(~Parameter, scales = "free", ncol = 2) +
    labs(
      title = paste("Bootstrap Distributions:", model_name),
      subtitle = "Red dashed line = original model estimate | Blue = bootstrap distribution",
      x = "Odds Ratio",
      y = "Frequency"
    ) +
    theme_minimal() +
    theme(
      plot.title = element_text(size = 14, face = "bold"),
      strip.text = element_text(size = 10, face = "bold")
    )
  
  return(p)
}

# Generate plots with model_number parameter
p_boot1 <- plot_boot_distributions(boot_model1, model1_final, 
                                    "Model 1 (First Choice)", model_number = 1)
p_boot2 <- plot_boot_distributions(boot_model2, model2_final, 
                                    "Model 2 (Top 2)", model_number = 2)

print(p_boot1)
```

```
print(p_boot2)
```

```
# Save plots
ggsave("output/bootstrap_distributions_model1.png", p_boot1, 
       width = 12, height = 10, dpi = 300)
ggsave("output/bootstrap_distributions_model2.png", p_boot2, 
       width = 12, height = 10, dpi = 300)

cat("\nBootstrap distribution plots saved\n")
```

```
## 
## Bootstrap distribution plots saved
```

```
# =============================================================================
#  TEST "SOLE PREDICTOR CLAIM"
# =============================================================================

cat("\n=== Testing 'Sole Predictor' Claim ===\n\n")
```

```
## 
## === Testing 'Sole Predictor' Claim ===
```

```
test_sole_predictor <- function(boot_object, model) {
  boot_samples <- boot_object$t
  param_names <- names(fixef(model))
  
  results <- list()
  
  for (i in 1:ncol(boot_samples)) {
    param_name <- param_names[i]
    ci <- quantile(boot_samples[, i], probs = c(0.025, 0.975), na.rm = TRUE)
    significant <- !(ci[1] < 0 & ci[2] > 0)
    results[[param_name]] <- significant
  }
  
  return(results)
}

sole_pred_m1 <- test_sole_predictor(boot_model1, model1_final)
sole_pred_m2 <- test_sole_predictor(boot_model2, model2_final)

cat("Model 1 - Bootstrap-based significance:\n")
```

```
## Model 1 - Bootstrap-based significance:
```

```
print(data.frame(
  Parameter = names(sole_pred_m1),
  Significant = unlist(sole_pred_m1)
))
```

```
##                                                                            Parameter
## (Intercept).2.5%                                                         (Intercept)
## consensus_strength.2.5%                                           consensus_strength
## question_complexitySimple (3 options).2.5%     question_complexitySimple (3 options)
## question_complexityModerate (4 options).2.5% question_complexityModerate (4 options)
## question_complexityComplex (5+ options).2.5% question_complexityComplex (5+ options)
## sourceClaude.2.5%                                                       sourceClaude
## sourceDeepseek.2.5%                                                   sourceDeepseek
## sourceGemini.2.5%                                                       sourceGemini
##                                              Significant
## (Intercept).2.5%                                   FALSE
## consensus_strength.2.5%                             TRUE
## question_complexitySimple (3 options).2.5%         FALSE
## question_complexityModerate (4 options).2.5%       FALSE
## question_complexityComplex (5+ options).2.5%       FALSE
## sourceClaude.2.5%                                  FALSE
## sourceDeepseek.2.5%                                FALSE
## sourceGemini.2.5%                                  FALSE
```

```
cat("\nModel 2 - Bootstrap-based significance:\n")
```

```
## 
## Model 2 - Bootstrap-based significance:
```

```
print(data.frame(
  Parameter = names(sole_pred_m2),
  Significant = unlist(sole_pred_m2)
))
```

```
##                                                                            Parameter
## (Intercept).2.5%                                                         (Intercept)
## consensus_strength.2.5%                                           consensus_strength
## question_complexityModerate (4 options).2.5% question_complexityModerate (4 options)
## question_complexityComplex (5+ options).2.5% question_complexityComplex (5+ options)
## sourceClaude.2.5%                                                       sourceClaude
## sourceDeepseek.2.5%                                                   sourceDeepseek
## sourceGemini.2.5%                                                       sourceGemini
##                                              Significant
## (Intercept).2.5%                                   FALSE
## consensus_strength.2.5%                             TRUE
## question_complexityModerate (4 options).2.5%       FALSE
## question_complexityComplex (5+ options).2.5%        TRUE
## sourceClaude.2.5%                                  FALSE
## sourceDeepseek.2.5%                                FALSE
## sourceGemini.2.5%                                   TRUE
```

```
cat("\n=== BOOTSTRAP ANALYSIS COMPLETE ===\n")
```

```
## 
## === BOOTSTRAP ANALYSIS COMPLETE ===
```

## 2.5 Model diagnostics

```
library(car)
library(DHARMa)
library(performance)

cat("=== MODEL DIAGNOSTICS ===\n\n")
```

```
## === MODEL DIAGNOSTICS ===
```

```
# -----------------------------------------------------------------------------
# 1. MULTICOLLINEARITY CHECK
# -----------------------------------------------------------------------------
cat("Variance Inflation Factors (Model 1):\n")
```

```
## Variance Inflation Factors (Model 1):
```

```
vif1 <- car::vif(model1_final)
print(vif1)
```

```
##                         GVIF Df GVIF^(1/(2*Df))
## consensus_strength  1.015758  1        1.007848
## question_complexity 1.013522  3        1.002241
## source              1.004232  3        1.000704
```

```
cat("\nVariance Inflation Factors (Model 2):\n")
```

```
## 
## Variance Inflation Factors (Model 2):
```

```
vif2 <- car::vif(model2_final)
print(vif2)
```

```
##                         GVIF Df GVIF^(1/(2*Df))
## consensus_strength  1.081406  1        1.039907
## question_complexity 1.080124  2        1.019456
## source              1.013383  3        1.002218
```

```
cat("\nAll VIF values should be < 5 (ideally < 3)\n\n")
```

```
## 
## All VIF values should be < 5 (ideally < 3)
```

```
# -----------------------------------------------------------------------------
# 2. RESIDUAL DIAGNOSTICS (DHARMa)
# -----------------------------------------------------------------------------
# Model 1
cat("Simulating residuals for Model 1...\n")
```

```
## Simulating residuals for Model 1...
```

```
sim1 <- simulateResiduals(fittedModel = model1_final, n = 1000)
plot(sim1, main = "Model 1: DHARMa Residual Diagnostics")
```

```
cat("\nModel 1 Residual Tests:\n")
```

```
## 
## Model 1 Residual Tests:
```

```
cat("Uniformity test: ", testUniformity(sim1, plot = FALSE)$p.value, "\n")
```

```
## Uniformity test:  0.6236182
```

```
cat("Dispersion test: ", testDispersion(sim1, plot = FALSE)$p.value, "\n")
```

```
## Dispersion test:  0.886
```

```
cat("Outlier test: ", testOutliers(sim1, plot = FALSE)$p.value, "\n\n")
```

```
## Outlier test:  1
```

```
# Model 2
cat("Simulating residuals for Model 2...\n")
```

```
## Simulating residuals for Model 2...
```

```
sim2 <- simulateResiduals(fittedModel = model2_final, n = 1000)
plot(sim2, main = "Model 2: DHARMa Residual Diagnostics")
```

```
cat("\nModel 2 Residual Tests:\n")
```

```
## 
## Model 2 Residual Tests:
```

```
cat("Uniformity test: ", testUniformity(sim2, plot = FALSE)$p.value, "\n")
```

```
## Uniformity test:  0.1818288
```

```
cat("Dispersion test: ", testDispersion(sim2, plot = FALSE)$p.value, "\n")
```

```
## Dispersion test:  0.992
```

```
cat("Outlier test: ", testOutliers(sim2, plot = FALSE)$p.value, "\n\n")
```

```
## Outlier test:  1
```

```
# -----------------------------------------------------------------------------
# 3. RANDOM EFFECTS ASSUMPTIONS
# -----------------------------------------------------------------------------
# Check normality of random intercepts
ranef1 <- ranef(model1_final)$question_id
ggplot(data.frame(intercept = ranef1[,1]), aes(sample = intercept)) +
  stat_qq() + stat_qq_line() +
  labs(title = "Model 1: Normality of Random Intercepts",
       subtitle = "Q-Q plot should follow diagonal line") +
  theme_minimal()
```

```
ranef2 <- ranef(model2_final)$question_id
ggplot(data.frame(intercept = ranef2[,1]), aes(sample = intercept)) +
  stat_qq() + stat_qq_line() +
  labs(title = "Model 2: Normality of Random Intercepts",
       subtitle = "Q-Q plot should follow diagonal line") +
  theme_minimal()
```

```
# -----------------------------------------------------------------------------
# 4. MODEL FIT STATISTICS
# -----------------------------------------------------------------------------
cat("=== MODEL FIT STATISTICS ===\n\n")
```

```
## === MODEL FIT STATISTICS ===
```

```
# Model 1
cat("Model 1 (First Choice Agreement):\n")
```

```
## Model 1 (First Choice Agreement):
```

```
r2_model1 <- r2(model1_final)
icc_model1 <- icc(model1_final)
cat("  Marginal R² (fixed effects):", round(r2_model1$R2_marginal, 3), "\n")
```

```
##   Marginal R² (fixed effects): 0.166
```

```
cat("  Conditional R² (fixed + random):", round(r2_model1$R2_conditional, 3), "\n")
```

```
##   Conditional R² (fixed + random): 0.418
```

```
cat("  ICC (question-level clustering):", round(icc_model1$ICC_adjusted, 3), "\n")
```

```
##   ICC (question-level clustering): 0.303
```

```
cat("  AIC:", round(AIC(model1_final), 1), "\n\n")
```

```
##   AIC: 399.5
```

```
# Model 2
cat("Model 2 (Top 2 Agreement):\n")
```

```
## Model 2 (Top 2 Agreement):
```

```
r2_model2 <- r2(model2_final)
icc_model2 <- icc(model2_final)
cat("  Marginal R² (fixed effects):", round(r2_model2$R2_marginal, 3), "\n")
```

```
##   Marginal R² (fixed effects): 0.322
```

```
cat("  Conditional R² (fixed + random):", round(r2_model2$R2_conditional, 3), "\n")
```

```
##   Conditional R² (fixed + random): 0.367
```

```
cat("  ICC (question-level clustering):", round(icc_model2$ICC_adjusted, 3), "\n")
```

```
##   ICC (question-level clustering): 0.066
```

```
cat("  AIC:", round(AIC(model2_final), 1), "\n\n")
```

```
##   AIC: 216
```

```
# Model comparison table
comparison_table <- data.frame(
  Model = c("First Choice", "Top 2 Choice"),
  N_observations = c(nobs(model1_final), nobs(model2_final)),
  N_questions = c(
    length(unique(analysis_dataset$question_id)),
    length(unique(analysis_dataset %>% 
      filter(question_complexity != "Binary (2 options)") %>% 
      pull(question_id)))
  ),
  AIC = round(c(AIC(model1_final), AIC(model2_final)), 1),
  ICC = round(c(icc_model1$ICC_adjusted, icc_model2$ICC_adjusted), 3),
  Marginal_R2 = round(c(r2_model1$R2_marginal, r2_model2$R2_marginal), 3),
  Conditional_R2 = round(c(r2_model1$R2_conditional, r2_model2$R2_conditional), 3)
)

comparison_table %>%
  kable(caption = "Model Fit Comparison",
        col.names = c("Model", "N obs", "N questions", "AIC", "ICC", 
                     "Marginal R²", "Conditional R²")) %>%
  kable_styling(bootstrap_options = c("striped", "hover"))
```

Model Fit Comparison

| Model | N obs | N questions | AIC | ICC | Marginal R² | Conditional R² |
| --- | --- | --- | --- | --- | --- | --- |
| First Choice | 324 | 81 | 399.5 | 0.303 | 0.166 | 0.418 |
| Top 2 Choice | 264 | 66 | 216.0 | 0.066 | 0.322 | 0.367 |

```
# -----------------------------------------------------------------------------
# SAVE RESULTS
# -----------------------------------------------------------------------------
saveRDS(model1_final, "output/model1_first_choice.rds")
saveRDS(model2_final, "output/model2_top2_choice.rds")
write.csv(tidy_model1, "output/model1_coefficients.csv", row.names = FALSE)
write.csv(tidy_model2, "output/model2_coefficients.csv", row.names = FALSE)
write.csv(comparison_table, "output/model_comparison.csv", row.names = FALSE)

cat("\n=== DIAGNOSTICS COMPLETE ===\n")
```

```
## 
## === DIAGNOSTICS COMPLETE ===
```

```
cat("All diagnostic tests passed acceptable thresholds\n")
```

```
## All diagnostic tests passed acceptable thresholds
```

```
cat("Models and results saved to output/ folder\n")
```

```
## Models and results saved to output/ folder
```

```
# Documentation
```

```
# Source the poster plots code
source("poster_plots_appendix.R")
```

```
## === GENERATING ALL POSTER-READY PLOTS WITH LARGER TEXT ===
## 
## 1. Creating agreement by complexity plot (first choice)...
```

```
## 2. Creating agreement by complexity plot (top 2)...
```

```
## 3. Creating consensus strength impact plot (first choice)...
```

```
## 4. Creating consensus strength impact plot (top 2)...
```

```
## 5. Creating domain heatmap (first choice)...
```

```
## 6. Creating domain heatmap (top 2)...
```

```
## 7. Creating complexity-stratified heatmaps with TOTAL rows...
```

```
## 8. Creating regression effect plots (Model 1)...
```

```
## 9. Creating regression effect plots (Model 2)...
```

```
## 10. Creating bootstrap distribution plots...
```

```
## 
## === ALL POSTER PLOTS GENERATED SUCCESSFULLY (WITH 30% LARGER TEXT) ===
## 
## Files saved to output/ directory:
##   1. poster_complexity_first.png
##   2. poster_complexity_top2.png
##   3. poster_consensus_first.png
##   4. poster_consensus_top2.png
##   5. poster_heatmap_first.png
##   6. poster_heatmap_top2.png
##   7. poster_complexity_heatmap_1_Binary_2_options_.png
##   8. poster_complexity_heatmap_2_Simple_3_options_.png
##   9. poster_complexity_heatmap_3_Moderate_4_options_.png
##  10. poster_complexity_heatmap_4_Complex_5+_options_.png
##  11. poster_complexity_heatmap_combined.png (WITH TOTAL ROWS)
##  12. poster_regression_model1_consensus.png
##  13. poster_regression_model1_llm.png
##  14. poster_regression_model1_complexity.png
##  15. poster_regression_model2_consensus.png
##  16. poster_regression_model2_llm.png
##  17. poster_regression_model2_complexity.png
##  18. poster_bootstrap_model1.png
##  19. poster_bootstrap_model2.png
## 
## Total: 19 high-resolution plots (300 dpi)
## All plots are formatted for poster presentation with 30% larger text sizes
## Complexity heatmaps now include TOTAL rows showing overall performance
## 
## === POSTER PLOTS APPENDIX COMPLETE ===
```

```
print(sessionInfo())
```

```
## R version 4.5.0 (2025-04-11)
## Platform: aarch64-apple-darwin20
## Running under: macOS Sequoia 15.6.1
## 
## Matrix products: default
## BLAS:   /Library/Frameworks/R.framework/Versions/4.5-arm64/Resources/lib/libRblas.0.dylib 
## LAPACK: /Library/Frameworks/R.framework/Versions/4.5-arm64/Resources/lib/libRlapack.dylib;  LAPACK version 3.12.1
## 
## locale:
## [1] en_US.UTF-8/en_US.UTF-8/en_US.UTF-8/C/en_US.UTF-8/en_US.UTF-8
## 
## time zone: Europe/Zurich
## tzcode source: internal
## 
## attached base packages:
## [1] parallel  stats     graphics  grDevices utils     datasets  methods  
## [8] base     
## 
## other attached packages:
##  [1] DHARMa_0.4.7        car_3.1-3           carData_3.0-5      
##  [4] sjPlot_2.9.0        performance_0.15.1  broom.mixed_0.2.9.6
##  [7] lme4_1.1-37         Matrix_1.7-3        cowplot_1.1.3      
## [10] gridExtra_2.3       janitor_2.2.1       emmeans_1.11.2-8   
## [13] ggeffects_2.3.1     ordinal_2023.12-4.1 MASS_7.3-65        
## [16] corrplot_0.95       broom_1.0.8         patchwork_1.3.0    
## [19] ggnewscale_0.5.2    scales_1.4.0        viridis_0.6.5      
## [22] viridisLite_0.4.2   plotly_4.10.4       DT_0.33            
## [25] kableExtra_1.4.0    knitr_1.50          lubridate_1.9.4    
## [28] forcats_1.0.0       stringr_1.5.1       dplyr_1.1.4        
## [31] purrr_1.0.4         readr_2.1.5         tidyr_1.3.1        
## [34] tibble_3.2.1        ggplot2_3.5.2       tidyverse_2.0.0    
## 
## loaded via a namespace (and not attached):
##  [1] RColorBrewer_1.1-3  rstudioapi_0.17.1   jsonlite_2.0.0     
##  [4] datawizard_1.2.0    magrittr_2.0.3      estimability_1.5.1 
##  [7] farver_2.1.2        nloptr_2.2.1        rmarkdown_2.29     
## [10] ragg_1.4.0          vctrs_0.6.5         minqa_1.2.8        
## [13] effectsize_1.0.1    htmltools_0.5.8.1   haven_2.5.4        
## [16] Formula_1.2-5       sjmisc_2.8.11       sass_0.4.10        
## [19] parallelly_1.44.0   bslib_0.9.0         htmlwidgets_1.6.4  
## [22] plyr_1.8.9          cachem_1.1.0        mime_0.13          
## [25] lifecycle_1.0.4     iterators_1.0.14    pkgconfig_2.0.3    
## [28] gap_1.6             sjlabelled_1.2.0    R6_2.6.1           
## [31] fastmap_1.2.0       rbibutils_2.3       future_1.49.0      
## [34] shiny_1.10.0        snakecase_0.11.1    digest_0.6.37      
## [37] numDeriv_2016.8-1.1 furrr_0.3.1         textshaping_1.0.1  
## [40] crosstalk_1.2.1     qgam_2.0.0          labeling_0.4.3     
## [43] timechange_0.3.0    httr_1.4.7          abind_1.4-8        
## [46] mgcv_1.9-3          compiler_4.5.0      withr_3.0.2        
## [49] doParallel_1.0.17   backports_1.5.0     ucminf_1.2.2       
## [52] tools_4.5.0         httpuv_1.6.16       glue_1.8.0         
## [55] nlme_3.1-168        promises_1.3.2      grid_4.5.0         
## [58] generics_0.1.4      gtable_0.3.6        tzdb_0.5.0         
## [61] data.table_1.17.0   hms_1.1.3           xml2_1.3.8         
## [64] utf8_1.2.5          foreach_1.5.2       pillar_1.10.2      
## [67] later_1.4.2         splines_4.5.0       lattice_0.22-7     
## [70] tidyselect_1.2.1    reformulas_0.4.1    svglite_2.2.1      
## [73] xfun_0.52           stringi_1.8.7       lazyeval_0.2.2     
## [76] yaml_2.3.10         boot_1.3-31         evaluate_1.0.3     
## [79] codetools_0.2-20    cli_3.6.5           xtable_1.8-4       
## [82] parameters_0.28.2   systemfonts_1.2.3   Rdpack_2.6.4       
## [85] jquerylib_0.1.4     Rcpp_1.0.14         globals_0.18.0     
## [88] coda_0.19-4.1       bayestestR_0.17.0   gap.datasets_0.0.6 
## [91] listenv_0.9.1       mvtnorm_1.3-3       insight_1.4.2      
## [94] rlang_1.1.6
```

---

*Report generated on 2025-10-25*
