# Supplementary material for: Performance of large language models in ambiguous treatment scenarios of early breast cancer
Source: Breast. 2026 Sep 23;90:104932. doi: 10.1016/j.breast.2026.104932 (PMC13641045; doi:10.1016/j.breast.2026.104932)
Supplement: Supplementary file 1 [file mmc1.zip › Suppl.Mat. - Anton Oseledchyk - Prompts, LLM Responses, R-Code/axillary_response_chatgpt.pdf]

QUESTION_ID: 1
SELECTED_ANSWER: C
EVIDENCE_SUMMARY: For a 60-year-old with a strongly ER+/PR+, HER2−, early breast cancer and clinically node-negative axilla, omission of sentinel lymph node biopsy (SLNB) is considered safe up to a 2 cm tumor size. Recent randomized trials (SOUND and INSEMA) showed that in patients ≥50 years with T1 tumors (≤2 cm) that are hormone receptor-positive, HER2-negative, and have negative axillary imaging, skipping SLNB did not compromise 5-year outcomes
breastsurgeons.org
breastsurgeons.org
. Axillary recurrence rates were very low (~0.4% at 5 years) despite ~14% occult nodal metastases in this group, likely due to effective systemic therapy and whole-breast irradiation
breastsurgeons.org
. This evidence supports omitting SLNB for cT1 luminal tumors when nodal status won't change adjuvant therapy.
KEY_REFERENCES: SOUND trial (Gentilini et al., JAMA Oncol 2023) – noninferiority of no SLNB in ≤2 cm, cN0, HR+ tumors
breastsurgeons.org
; INSEMA trial (Reimer et al., NEJM 2024) – confirmed no survival detriment omitting SLNB in node-negative, ≤5 cm (mostly ≤2 cm) tumors
breastsurgeons.org
.
GUIDELINE_ALIGNMENT: 2023–early 2025 guidelines (ASCO, NCCN) endorse SLNB omission in postmenopausal patients ≥50 years with cT1, grade 1–2, HR+/HER2− cancers and negative axillary ultrasound, undergoing breast-conserving therapy
ascopost.com
. This aligns with Choosing Wisely/SSO and Cancer Care Ontario recommendations to forgo SLNB in low-risk tumors, historically applied to age ≥70 but now extended to younger postmenopausal women with these favorable features
pmc.ncbi.nlm.nih.gov
.
SUBGROUP_NOTES: Omission is most appropriate for strongly ER+ tumors with low proliferation (luminal A/B) since endocrine therapy will be given, and the SLNB result would not alter chemotherapy decisions
breastsurgeons.org
. Patients with high-grade (grade 3) disease or those who might need chemotherapy if nodes are positive (e.g. younger patients, large tumor or high genomic score) were underrepresented in trials and may still benefit from staging
breastsurgeons.org
. An interdisciplinary discussion is recommended to ensure skipping SLNB won't deprive the patient of additional therapies (e.g. chemotherapy, CDK4/6 inhibitors, extended endocrine therapy) that positive nodes might indicate
breastsurgeons.org
breastsurgeons.org
.
CONFIDENCE_LEVEL: High – Based on two large randomized trials with 5–6 year follow-up (Level I evidence) and consistent guideline

updates up to Feb 2025 supporting this practice in the defined subgroup
breastsurgeons.org
ascopost.com
.
WEB_SEARCH_USED: Yes – Searched for “SOUND trial axillary surgery omission” and “ASCO guideline SLNB 2025”. Found SOUND (JAMA Oncol 2023) and INSEMA (NEJM 2024) results confirming noninferiority of no SLNB in ≤2 cm HR+ tumors
breastsurgeons.org
breastsurgeons.org
, and ASCO 2025 guideline update recommending SLNB omission in postmenopausal women with ≤2 cm, grade 1–2, HR+/HER2– cancers
ascopost.com
.
---END---

QUESTION_ID: 2
SELECTED_ANSWER: A
EVIDENCE_SUMMARY: For a 60-year-old with a strongly ER+/PR+, HER2-positive tumor, SLNB should generally be performed for tumors >1 cm, as omission of SLNB is not well supported in HER2-positive disease beyond very small sizes. Trials of SLNB omission primarily included HER2-negative cancers; only ~3–4% of patients in INSEMA had HER2-positive tumors
breastsurgeons.org
, and the omission recommendations were targeted to HR+/HER2– cases
breastsurgeons.org
. Thus, for HER2-positive tumors, especially ≥1 cm (where adjuvant chemotherapy + anti-HER2 therapy is standard), pathologic nodal status is important for guiding regional radiotherapy and systemic treatment decisions. Omitting SLNB might only be considered for subcentimeter (T1a–T1b ≤1.0 cm) HER2+ tumors in select low-risk scenarios, but otherwise sentinel surgery is advised.
KEY_REFERENCES: INSEMA trial (Reimer et al., NEJM 2024) – showed SLNB omission was noninferior in predominantly HR+/HER2– tumors up to 5 cm, but only 3.6% were HER2+, limiting conclusions for HER2+ cases
breastsurgeons.org
breastsurgeons.org
; ASCO/SSO guidelines (2017–2024) – recommend SLNB omission only in HR+/HER2– elderly patients, implying HER2+ tumors are excluded
pmc.ncbi.nlm.nih.gov
.
GUIDELINE_ALIGNMENT: Up to Feb 2025, no major guideline endorses routine SLNB omission for HER2-positive breast cancers. ASCO and NCCN guidelines restrict SLNB omission to HR+/HER2– disease in older patients or select postmenopausal women
pmc.ncbi.nlm.nih.gov
ascopost.com
. For HER2-positive tumors ≥1 cm, guidelines recommend SLNB for accurate staging, as nodal status can influence radiation fields and some systemic therapy decisions (e.g. duration of anti-HER2 therapy if high nodal burden).

SUBGROUP_NOTES: HER2-positive cancers, even if strongly ER+, have higher risk of nodal spread and typically warrant chemotherapy with HER2-targeted therapy for tumors >1 cm. Knowing nodal involvement is important for considering regional nodal irradiation and additional therapy (e.g., extended adjuvant anti-HER2 in node-positive disease). In an older or frail patient with a very small HER2+ tumor (<5 mm) who would not change management based on nodes, omission of SLNB could be discussed on a case-by-case basis, but data are very limited.
CONFIDENCE_LEVEL: High – There is consistent consensus that outside of extremely low-risk, small tumors, HER2-positive disease should be staged with SLNB. The evidence supporting SLNB omission (SOUND/INSEMA) largely excluded HER2+ cases
breastsurgeons.org
, so extrapolation to HER2+ is unsupported, yielding strong agreement to perform SLNB for tumors >1 cm.
WEB_SEARCH_USED: Yes – Queried "SLNB omission HER2 positive breast cancer SOUND" and reviewed trial data. Found that SOUND and INSEMA trials predominantly enrolled HER2-negative patients
breastsurgeons.org
and that ASCO/Cancer Care Ontario guidelines limit SLNB omission to HR+ HER2– tumors in older age
pmc.ncbi.nlm.nih.gov
. No new evidence (up to Feb 2025) supports skipping SLNB in HER2+ beyond possibly tiny tumors, confirming the need for SLNB if >1 cm.
---END---

QUESTION_ID: 3
SELECTED_ANSWER: G
EVIDENCE_SUMMARY: In a 60-year-old with triple-negative breast cancer, SLNB should be performed regardless of tumor size (barring perhaps truly minimal disease) because triple-negative tumors have higher nodal metastasis risk and lack less toxic adjuvant options to compensate for unknown nodal status. Trials of SLNB omission did not specifically support skipping axillary staging in TNBC – they enrolled very few grade 3 or triple-negative cases (only ~3–4% grade 3 in INSEMA)
breastsurgeons.org
and focused on lower-risk biology. Moreover, triple-negative tumors ≥0.5 cm usually indicate chemotherapy, but accurate nodal staging guides regional radiation. Therefore, no clear tumor size cutoff exists below which SLNB can be broadly omitted for TNBC; even small TNBC (≥T1b) often get systemic therapy, and undetected nodal disease could lead to undertreatment of regional nodes if SLNB is omitted.
KEY_REFERENCES: SOUND trial (Gentilini et al., 2023) – included predominantly ER+ tumors; only ~18% were grade 3 and presumably very few triple-negatives
breastsurgeons.org
breastsurgeons.org
, so findings don't generalize to TNBC; NCCN Guidelines v4.2022 – suggest SLNB is standard for cN0 patients <70, with omission only optional in "particularly favorable" tumors unlikely to change therapy
pmc.ncbi.nlm.nih.gov

(which does not include triple-negative disease).
GUIDELINE_ALIGNMENT: Current guidelines (NCCN, ASCO up to 2024) do not recommend SLNB omission for triple-negative breast cancers. The guideline exceptions for skipping SLNB apply to elderly patients with HR+ HER2– tumors; triple-negative cancers are high-risk and are excluded from omission recommendations
pmc.ncbi.nlm.nih.gov
. Thus, standard of care is to perform SLNB for clinically node-negative TNBC of essentially any appreciable size.
SUBGROUP_NOTES: Triple-negative patients derive substantial benefit from chemotherapy even when node-negative, but knowing nodal status is crucial for radiation planning – e.g., a node-positive TNBC patient after surgery should receive regional nodal irradiation. While a very small TNBC (<5 mm) in a patient unfit for chemo could prompt a case-by-case discussion to avoid SLNB, this is unusual. Generally, TNBC's aggressive nature and lack of targeted therapy mean aggressive local-regional staging and treatment are warranted.
CONFIDENCE_LEVEL: High – There is strong consensus and indirect evidence that TNBC should not be managed with axillary omission outside rare scenarios. The omission trials focused on biologically favorable cancers, and experts caution against extrapolating those results to triple-negative tumors
breastsurgeons.org
. No new data through Feb 2025 suggest safety of skipping SLNB in TNBC, so confidence is high that SLNB is required regardless of tumor size beyond microinvasion.
WEB_SEARCH_USED: Yes – Researched "SLNB omission triple-negative breast cancer" and reviewed SOUND/INSEMA trial subgroups. Confirmed that few TNBC patients were included (trials limited to favorable biology)
breastsurgeons.org
breastsurgeons.org
. Also checked guidelines (SSO Choosing Wisely, ASCO) which restrict SLNB omission to HR+ cases, effectively excluding TNBC
pmc.ncbi.nlm.nih.gov
. No new 2024 evidence supports SLNB omission in TNBC, reinforcing the need for SLNB in all cN0 TNBC.
---END---

QUESTION_ID: 4
SELECTED_ANSWER: B
EVIDENCE_SUMMARY: We should not automatically apply the same SLNB omission criteria to invasive lobular carcinoma (ILC) as to invasive ductal carcinoma (IDC) because data specific to ILC are limited. While the SOUND and INSEMA trials did include some lobular cancers (~8.5% in SOUND, ~12.7% in INSEMA)
ascopubs.org
, the majority of patients had ductal tumors, and no separate analysis was reported for lobular outcomes. ILC often presents as diffuse, multifocal disease that can be underestimated on imaging, potentially increasing the risk of occult nodal involvement. Given the paucity of lobular-specific evidence, many experts are cautious about omitting SLNB for ILC until further data confirm safety in this subgroup.

KEY_REFERENCES: SOUND trial (Gentilini 2023) & INSEMA trial (Reimer 2024) – enrolled typical proportions of lobular carcinoma (≈10%)
ascopubs.org
but did not specifically address ILC outcomes, so omission recommendations were not tailored to ILC; SSO Choosing Wisely 2016 – suggested SLNB omission for certain elderly patients but acknowledged lack of ILC-specific evidence and the need to validate omission in lobular cancers
pmc.ncbi.nlm.nih.gov
.
GUIDELINE_ALIGNMENT: No current guideline (NCCN, ASCO as of early 2025) explicitly distinguishes ILC vs IDC for SLNB omission; however, the eligibility criteria in guidelines (e.g. postmenopausal, ≤2 cm, HR+ HER2–, negative ultrasound) were derived from trials largely comprised of IDC
ascopubs.org
. Implicitly, guidelines urge caution in unstudied subgroups: clinicians are advised to use SLNB omission criteria only if the patient closely matches trial populations, meaning limited data on ILC should temper omission decisions in lobular cases.
SUBGROUP_NOTES: Invasive lobular carcinoma tends to be ER+ and low-grade, which on the surface fits the “favorable” profile, but ILC’s diffuse growth can lead to larger pathological size and unpredictable nodal spread despite negative imaging. If an ILC meets all favorable criteria (small, low-grade, node-negative by exam/imaging), some may consider SLNB omission, but only with informed patient discussion. Pending dedicated data, many clinicians still perform SLNB in ILC to avoid missing occult positive nodes that could influence radiation or systemic therapy decisions.
CONFIDENCE_LEVEL: Moderate – The general principles of axillary management likely apply to ILC, but the limited subset of lobular patients in trials means there’s moderate uncertainty. We base recommendations on indirect evidence and expert opinion rather than lobular-specific trial data, so confidence is a step lower than for IDC cases.
WEB_SEARCH_USED: Yes – Investigated “SLNB omission in lobular carcinoma SOUND trial” and guideline commentary. Found that lobular histology made up ~10% of patients in SOUND/INSEMA
ascopubs.org
, with commentators noting no separate outcomes reported (hence limited data). A literature check (e.g. Hersh & King 2023 review) emphasized caution in ILC due to potential imaging underestimation, confirming the “disagree” stance absent robust data.
---END---

QUESTION_ID: 5
SELECTED_ANSWER: A
EVIDENCE_SUMMARY: In an ER+/HER2– patient who had neoadjuvant chemotherapy (NACT) and is found to have only isolated tumor cells (ITC) in 1 of 4 sentinel lymph nodes (SLNs) (ypN0[i+]), no further axillary treatment is needed beyond standard breast radiation. Isolated tumor cells (<0.2 mm clusters) are not considered true nodal metastases (classified as pN0)
ascopubs.org

and have not shown impact on survival; trials in the upfront surgery setting (e.g. ACOSOG Z0010/IBCSG 23-01) indicated that ITCs do not necessitate axillary dissection or nodal radiation. Furthermore, after NACT, the presence of only ITCs suggests an excellent nodal response – additional axillary treatment (ALND or nodal irradiation) would add morbidity with no proven benefit
pubmed.ncbi.nlm.nih.gov
. Whole-breast irradiation (which incidentally treats low axilla levels) and systemic endocrine therapy will address any microscopic residual disease.
KEY_REFERENCES: AJCC Staging 8th Edition – defines ITCs (≤0.2 mm) as pN0, not counting as positive nodes
ascopubs.org
; IBCSG 23-01 trial (2013) – showed no recurrence or survival disadvantage when ALND was omitted for minimal nodal disease (micrometastases/ITCs) with breast-conserving therapy; Meta-analysis (Zaborowski et al., EJSO 2025) – in post-NACT patients, omitting ALND in those with minimal residual nodal involvement did not worsen 5-year overall survival or axillary control
pubmed.ncbi.nlm.nih.gov
.
GUIDELINE_ALIGNMENT: NCCN and ASCO guidelines regard ITCs as node-negative for management purposes; completion ALND is not indicated for ITC-only SLNs. After NACT, guidelines (2023) still recommend ALND for confirmed nodal metastases, but ITCs are an exception often treated as pN0. In this scenario, the patient will receive whole-breast irradiation, and guidelines would consider that sufficient without further axillary-specific intervention, consistent with Z0011-era practices for minimal nodal burden.
SUBGROUP_NOTES: The patient's tumor biology (ER+/HER2–) further supports a conservative approach: she will receive endocrine therapy which helps eradicate microscopic disease. Her favorable response (only ITCs) implies no substantial nodal tumor remained. If she had a mastectomy (no breast radiation), management might differ (some would consider nodal irradiation for any nodal disease), but with lumpectomy and radiation, ITCs do not warrant escalation. Age and comorbidities can also be considered – here at 60 and presumably fit, standard practice still avoids unnecessary ALND for ITCs given lack of benefit.
CONFIDENCE_LEVEL: High – Strong evidence indicates no additional axillary therapy is needed for ITC findings. Long-term outcomes for ITC-only nodes treated without ALND are excellent, and multiple studies/guidelines concur that ITCs alone do not justify further axillary intervention
ascopubs.org
.
WEB_SEARCH_USED: Yes – Searched "post-NAC isolated tumor cells management" and "ITC breast cancer guidelines". Confirmed that ITCs are classified as pN0(i+) and do not count as positive nodes
ascopubs.org
. A 2025 systematic review/meta-analysis found no survival disadvantage omitting ALND in patients with minimal residual nodal disease after NACT
pubmed.ncbi.nlm.nih.gov

, reinforcing that no additional axillary treatment is needed for ITC in this context.
---END---

QUESTION_ID: 6
SELECTED_ANSWER: A
EVIDENCE_SUMMARY: For an ER+/HER2− patient post-NACT with a single sentinel node showing a micrometastasis (0.2–2 mm, ypN1mi) in 1 of 4 nodes, no completion ALND is recommended; whole-breast irradiation alone is sufficient axillary management. Evidence from trials in upfront surgery (IBCSG 23-01) demonstrated that patients with one or two micrometastatic SLNs who received breast-conserving therapy had no worse outcomes without ALND
nature.com
. Extrapolated to the post-NACT setting, a lone micrometastatic deposit after chemo indicates a good partial response and limited residual disease. Retrospective analyses and meta-data show no survival or recurrence penalty when ALND is omitted in cases of low-volume nodal disease after NACT
pubmed.ncbi.nlm.nih.gov
. Thus, with only 1 micro-positive node and planned breast radiation, further axillary surgery or dedicated nodal radiation is not necessary.
KEY_REFERENCES: IBCSG 23-01 (Galimberti et al., 2013) – randomized patients with ≤2 SLN micrometastases to ALND vs observation: no difference in 5-year DFS, supporting omission of ALND for micrometastases
nature.com
; ACOSOG Z0011 (Giuliano et al., 2011) – though in upfront setting, showed no benefit to ALND with 1–2 positive nodes (mostly macromets) when whole-breast RT given, implying even more so for micromets; Meta-analysis (2025) – including post-NACT studies, found omission of ALND in ypN1 (limited nodes) did not worsen axillary control or OS
pubmed.ncbi.nlm.nih.gov
.
GUIDELINE_ALIGNMENT: Contemporary guidelines allow skipping ALND for low-volume nodal disease. ASCO's 2025 update explicitly supports no ALND for 1–2 positive nodes in patients receiving breast RT
ascopost.com
(even in mastectomy cases with nodal RT, per SENOMAC data). While those guidelines addressed upfront surgery, the principle of avoiding ALND for minimal nodal involvement can extend post-NACT. As of Feb 2025, most guidelines would still formally call for ALND if any SLN is positive after NACT; however, expert practice has trended towards de-escalation for N1mi, especially if only one node and patient will get radiotherapy.
SUBGROUP_NOTES: This patient's luminal subtype will receive endocrine therapy, which helps control microscopic disease. The fact that only 1 of 4 nodes has a micromet suggests any additional nodal tumor burden is likely very low. If multiple nodes were positive or if the patient were not getting radiation (e.g. mastectomy scenario), recommendations might differ (possibly nodal irradiation). Patient preferences and comorbidities also matter:

avoiding ALND spares her the morbidity of lymphedema and shoulder stiffness, which is justified by the low anticipated benefit of ALND in this scenario.
CONFIDENCE_LEVEL: High – Based on robust trial data (IBCSG 23-01, Z0011) and emerging post-NACT evidence, we have high confidence that omitting ALND is safe when only a single micrometastatic node remains and radiation is planned
nature.com
pubmed.ncbi.nlm.nih.gov
. The consistency of findings in favor of no additional surgery for minimal nodal disease makes this a strong recommendation.
WEB_SEARCH_USED: Yes – Looked up "micrometastasis after neoadjuvant ALND needed?" and reviewed IBCSG 23-01 and de-escalation studies. Found that ALND omission for micromets is supported by trials
nature.com
. Also found 2025 meta-analysis indicating no detriment in survival or regional control without ALND in patients with limited nodal disease post-NACT
pubmed.ncbi.nlm.nih.gov
. This guided the recommendation of no further axillary therapy for 1 micrometastatic SLN.
---END---

QUESTION_ID: 7
SELECTED_ANSWER: C
EVIDENCE_SUMMARY: In an ER+/HER2– patient with residual micrometastatic disease in 2 of 4 SLNs after NACT, axillary lymph node dissection can be avoided, but adding regional nodal irradiation (RNI) is recommended for additional coverage. Having two positive sentinel nodes (even if micromet-sized) indicates a slightly higher axillary tumor burden; trials like ACOSOG Z0011 (upfront surgery) showed excellent control without ALND in patients with 1–2 positive nodes when whole-breast RT was given
nature.com
. Extrapolating post-NACT, omitting ALND is still reasonable, and the AMAROS trial suggests nodal radiation can substitute for surgery in controlling disease
nature.com
. Therefore, we would treat the axilla with radiation rather than surgery – whole-breast radiation with high tangents or comprehensive nodal irradiation – to address potential residual disease beyond the SLNs. This approach balances oncologic safety with lower morbidity, given endocrine therapy will also address systemic risk.
KEY_REFERENCES: ACOSOG Z0011 (2011) – no survival or recurrence difference with ALND vs no ALND in 1–2 node-positive lumpectomy patients, implying ALND can be skipped if RT is delivered
nature.com
; EORTC AMAROS (2014) – in upfront node-positive (sentinel) patients, axillary RT yielded equal regional control as ALND with less lymphedema, supporting RT in lieu of ALND for limited nodal disease; Meta-analysis (Zaborowski 2025) – in pooled post-NACT data, SLNB-only patients with limited nodes (including 2 nodes) had no worse 5-yr OS or axillary control than those who had ALND
pubmed.ncbi.nlm.nih.gov

.
GUIDELINE_ALIGNMENT: No explicit post-NACT guideline covers this exactly, but trends in guidelines favor less surgery for limited nodal involvement. NCCN (2023) advises considering nodal RT instead of ALND for 1–2 positive nodes in lumpectomy patients, based on Z0011/AMAROS. ASCO 2025 guidelines endorse omission of ALND even for mastectomy patients with 1–2 positive SLNs if they will receive nodal RT
ascopost.com
. Thus, offering nodal irradiation in lieu of ALND aligns with evolving guideline acceptance of radiation as adequate management for low-volume nodal disease.
SUBGROUP_NOTES: In a luminal cancer, two micrometastatic nodes still predict a good prognosis with endocrine therapy, but they do upstage her to clinical N1. Patient factors (age 60, presumably postmenopausal) and tumor factors (grade 2, good chemo response) favor avoiding ALND. We ensure regional therapy via radiation: typically, this would include supraclavicular ± axillary field radiation given two positive nodes, especially since nodal status is known. If this patient had contraindications to RT (e.g. prior radiation) or if surgical staging was inadequate (e.g. <3 SLNs examined), one might lean toward ALND. Here she had 4 SLNs retrieved, making false-negative risk low, so radiation is a sound choice.
CONFIDENCE_LEVEL: Moderate/High – Data strongly support not doing ALND, but recommending nodal irradiation is based on expert consensus and extrapolation (since trials didn't specifically randomize post-NACT patients with residual micromets). Given consistency of multiple trials (Z0011, AMAROS, IBCSG 23-01) and meta-analyses favoring non-inferiority of less invasive management
nature.com
pubmed.ncbi.nlm.nih.gov
, confidence is high that this approach is safe; it's moderate only in so far as post-NACT prospective data are still maturing.
WEB_SEARCH_USED: Yes – Searched "2 positive sentinel nodes after neoadjuvant" and reviewed Z0011 and AMAROS outcomes. Found that even with two positive nodes, ALND did not improve survival if radiation was given
nature.com
. Also consulted 2025 ASCO updates suggesting ALND omission for 1–2 nodes with planned RT
ascopost.com
. These searches confirmed that nodal irradiation is an appropriate substitute for ALND in this scenario.
---END---

QUESTION_ID: 8
SELECTED_ANSWER: A
EVIDENCE_SUMMARY: For a triple-negative breast cancer patient post-NACT with isolated tumor cells in 1 of 4 SLNs (ypN0[i+]), no further axillary treatment is indicated beyond the planned breast irradiation. ITCs alone are essentially considered node-negative (pN0)
ascopubs.org

, and their presence after chemotherapy suggests an almost complete nodal response. Given that she will receive whole-breast radiation (which covers level I axilla), additional axillary-specific intervention (ALND or nodal field RT) would confer little benefit and more harm. Studies have shown extremely low regional recurrence when only ITCs remain and no ALND is done, especially with triple-negative patients having received effective systemic therapy. Moreover, guidelines do not recommend ALND for ITCs in any subtype.
KEY_REFERENCES: AJCC 8th Ed. – classifies ITCs as not counting toward positive node count
ascopubs.org
; Chun et al., 2021 (Sci Rep) – retrospective data from NAC-treated patients showed no survival disadvantage when ALND was omitted in those with only minimal nodal disease (ITC/micromet) after chemo; Zaborowski et al., 2025 (meta-analysis) – omission of ALND in patients with post-NACT ITC/micromet was not associated with higher axillary recurrence or worse OS
pubmed.ncbi.nlm.nih.gov
.
GUIDELINE_ALIGNMENT: Standard guidelines treat ITC-only SLN findings as node-negative – hence no ALND. Post-NACT, NCCN would typically advise ALND for residual nodal disease; however, ITCs fall below the threshold of “disease” requiring action. Thus, this scenario aligns with general guidelines: proceed with adjuvant therapy (here, radiation, plus capecitabine typically for TNBC residual tumor) without extra axillary dissection or radiation. The existing evidence and guideline stance are consistent regardless of subtype when it comes to ITCs.
SUBGROUP_NOTES: Triple-negative biology doesn’t change the approach for ITCs because these tiny cell clusters are effectively handled by prior chemo and will be sterilized by radiation. The patient likely will also receive adjuvant capecitabine (per CREATE-X) for any residual TNBC, providing systemic coverage. If she had a mastectomy (no breast RT), one might more closely watch the axilla but still would not do ALND for ITC. Her relatively younger age (60) and triple-negative status mean we want to ensure adequate therapy, but in this case “adequate” is fulfilled by systemic therapy and standard radiation – additional axillary surgery would only increase morbidity with no proven benefit at this microscopic disease level.
CONFIDENCE_LEVEL: High – Strong consensus and data indicate that ITCs do not merit further axillary treatment. Triple-negative status does not override the principle that ITCs are biologically insignificant in terms of loco-regional control when appropriate systemic and radiation therapies are given
ascopubs.org
pubmed.ncbi.nlm.nih.gov
.
WEB_SEARCH_USED: Yes – Researched “isolated tumor cells after neoadjuvant triple negative”. Found that residual ITCs post-NACT are rare (~1–2% incidence) and carry minimal risk
ascopubs.org
. The 2025 meta-analysis of SLNB alone after NACT confirmed no higher recurrence when ALND was omitted for minimal nodal findings
pubmed.ncbi.nlm.nih.gov

. This supported the decision for no further axillary treatment in this TNBC case with ITCs.
---END---

QUESTION_ID: 9
SELECTED_ANSWER: C
EVIDENCE_SUMMARY: In a triple-negative patient with one SLN containing a micrometastasis after NACT (1 of 4 nodes, ypN1mi), completion ALND can be avoided, but regional nodal irradiation is advised. Limited retrospective evidence suggests that even TNBC patients with 1–2 residual positive nodes can safely forego ALND if nodal radiation is given
nature.com
. However, because triple-negative disease has no further “targeted” therapy beyond chemotherapy, ensuring local control via radiation is prudent. By delivering nodal irradiation (e.g., to axilla levels I–III and supraclavicular fossa) in addition to whole-breast RT, we effectively treat any potential remaining microscopic disease in the axilla, achieving outcomes comparable to ALND. A meta-analysis of post-NACT patients found no increase in axillary recurrence when ALND was omitted in those with low-volume nodal metastases, presumably because radiation was often utilized
pubmed.ncbi.nlm.nih.gov
. Thus, ALND is not necessary for a single micrometastatic SLN in TNBC, provided comprehensive radiation is given.
KEY_REFERENCES: Chun et al., 2021 (Scientific Reports) – in NAC-treated patients with 1–2 residual nodal metastases (various subtypes), no differences in DFS/OS were seen with SLNB alone vs ALND
nature.com
; Zaborowski et al., 2025 (EJSO meta-analysis) – among ~9,889 post-NACT patients, omitting ALND in those with limited nodal positivity (including micromets) did not worsen 5-year OS or regional control
pubmed.ncbi.nlm.nih.gov
; ACOSOG Z1071 (2013) – established feasibility of SLNB after NACT in initially node-positive patients; while it mandated ALND, its findings support reliance on SLNB for nodal status with multiple nodes removed.
GUIDELINE_ALIGNMENT: Formal guidelines (e.g., NCCN 2023) still recommend ALND for any residual nodal disease after NACT in TNBC, because prospective trials are pending. However, guideline committees recognize de-escalation trends: if only 1 node with micrometastatic disease remains, many tumor boards would proceed with nodal irradiation instead of ALND, consistent with emerging evidence. This approach aligns with the general principle from Z0011/AMAROS that nodal RT can control limited nodal disease. Until trials like Alliance A011202 report results, this is a cautious off-guideline approach informed by data.
SUBGROUP_NOTES: TNBC with residual nodal disease is a higher-risk scenario for recurrence, which is why we compensate by using nodal radiation. The patient will presumably also receive adjuvant capecitabine (which improves DFS in residual TNBC), providing systemic cover. If she were younger (<40) or had BRCA mutation, some might be even more aggressive locally, but evidence does not

indicate ALND improves outcomes. If multiple nodes had been positive or if SLNB mapping was suboptimal, ALND would be more strongly considered. But here, only one micromet in 4 nodes suggests low burden and reliable staging, supporting radiation-alone strategy.
CONFIDENCE_LEVEL: Moderate – While data suggest safety, triple-negative biology makes some clinicians uneasy about skipping surgery. Confidence is bolstered by consistent retrospective outcomes
nature.com
pubmed.ncbi.nlm.nih.gov
, but without prospective TNBC-specific trial results, this recommendation relies on extrapolation and multidisciplinary consensus (hence not “high” confidence, but moderate with evidence-based rationale).
WEB_SEARCH_USED: Yes – Searched “triple negative residual micrometastasis ALND vs radiation”. Found a 2025 meta-analysis confirming no OS or regional control benefit to ALND in patients with limited nodal metastases after NACT
pubmed.ncbi.nlm.nih.gov
. Also found institutional studies (e.g., from MD Anderson/Asan) supporting SLNB-only plus RT in ypN1 cases
nature.com
. These findings guided recommending nodal irradiation (for local control) without ALND in this TNBC scenario.
---END---

QUESTION_ID: 10
SELECTED_ANSWER: B
EVIDENCE_SUMMARY: For a triple-negative patient with micrometastatic disease in 2 of 4 SLNs after NACT, an axillary lymph node dissection is recommended. Two positive nodes (even micromet-sized) indicate a higher residual nodal burden, and in triple-negative disease – which has greater risk of locoregional recurrence – standard practice has been to proceed with ALND to ensure clearance of disease. Current guidelines still endorse completion ALND for any nodal metastases post-NACT in TNBC
sciencedirect.com
. While emerging studies suggest it might be possible to omit ALND with nodal radiation, this approach for multiple node involvement in TNBC lacks prospective validation. Given the more aggressive biology and the possibility of additional non-sentinel nodes harboring disease (higher with two positive SLNs), ALND provides definitive staging and local control.
KEY_REFERENCES: NCCN Guidelines (2024) – recommend ALND for patients with positive SLNs after neoadjuvant therapy, especially in cases like TNBC where nodal involvement remains
sciencedirect.com
; ACOSOG Z1071 (2013) – showed a ~12.6% false-negative rate of SLNB after NACT in initially node-positive patients (emphasizing caution if residual nodes are found); MD Anderson retrospective (2018) – reported that patients with multiple residual nodal metastases had improved regional control with ALND vs no ALND (though selection biases apply). ([Note: A 2025 meta-analysis suggests no OS detriment omitting ALND

pubmed.ncbi.nlm.nih.gov
, but patient selection and use of radiation are confounders])
GUIDELINE_ALIGNMENT: As of Feb 2025, guidelines (ASCO, NCCN) lean toward ALND for ypN+ with more than one node involved. The standard of care for TNBC with even limited nodal disease post-NACT is still surgical clearance of the axilla. Future guideline changes are expected pending trial results, but currently a patient with 2 positive SLNs (even micrometastases) would fall outside the validated “Z0011-like” criteria and be advised to undergo ALND.
SUBGROUP_NOTES: In triple-negative patients, residual nodal disease correlates with higher recurrence risk, and additional therapy (capecitabine) will be used to improve systemic outcomes. Locally, if ALND is done and reveals only those 2 micrometastatic nodes (and no others), some centers might omit regional nodal irradiation (since ALND provides local control), balancing toxicity. If patient factors (e.g., significant comorbidities or patient refusal) preclude ALND, one would then strongly consider comprehensive nodal irradiation as an alternative. But in a fit 60-year-old, ALND is the more assured path for local control in this scenario.
CONFIDENCE_LEVEL: Moderate – There is solid rationale and guideline support for ALND here, but growing retrospective evidence hints that ALND might be safely avoided with nodal RT. Until randomized trial data confirm that, the recommendation is made with moderate confidence favoring the traditional approach (erring on the side of caution for TNBC). The evidence is not as unequivocal as in luminal cases due to lack of direct trials, hence “moderate” confidence.
WEB_SEARCH_USED: Yes – Used queries like “ALND vs radiation residual nodes TNBC”. This revealed current guideline positions emphasizing ALND for post-NACT nodal disease
sciencedirect.com
. It also surfaced data that, although ALND might not improve survival
pubmed.ncbi.nlm.nih.gov
, in practice for multiple positive nodes many centers still perform ALND. These searches underscored that, for two positive nodes in TNBC, ALND remains the prevalent recommendation pending further evidence.
---END---

QUESTION_ID: 11
SELECTED_ANSWER: A
EVIDENCE_SUMMARY: In an HER2-positive patient post-NACT with only isolated tumor cells in 1 of 4 SLNs (ypN0[i+]), no further axillary treatment is indicated beyond breast radiation. ITCs are not considered macroscopic nodal metastases (classified as node-negative)
ascopubs.org
, and this minimal residual reflects an almost complete response in the axilla. With whole-breast irradiation (covering level I), any microscopic foci will be addressed, and the patient will continue HER2-targeted therapy (which contributes to eradicating residual disease systemically). There is no evidence that ALND or added nodal radiation improves outcomes when only ITCs are present – on the contrary, studies show negligible axillary recurrence in such cases

without ALND
pubmed.ncbi.nlm.nih.gov
. Thus, we treat her as if node-negative after NACT.
KEY_REFERENCES: AJCC staging – defines SLN ITCs as pN0 (no additional therapy needed for that finding)
ascopubs.org
; SOUND/INSEMA paradigm – even upfront, patients with minimal occult nodal involvement had excellent outcomes without axillary surgery
breastsurgeons.org
; Zaborowski et al. 2025 – systematic review indicating omission of ALND in patients with ITCs/micromets post-NACT did not raise axillary failure rates
pubmed.ncbi.nlm.nih.gov
.
GUIDELINE_ALIGNMENT: Guidelines concur that ITCs do not mandate ALND. Post-NACT, standard guidance would be ALND for “true” nodal metastases; however, ITCs are below that threshold. Thus, in practice, this aligns with guidelines treating her as ypN0. She will receive the indicated adjuvant therapies (radiation, completion of anti-HER2 regimen, possibly endocrine if ER+) without any axillary dissection or boost. This approach is consistent across subtypes – HER2+ status doesn’t change management of ITCs, as guidelines don’t carve out exceptions for subtype when the nodal burden is this low.
SUBGROUP_NOTES: HER2-positive tumors often achieve pCR in nodes; the presence of only ITCs suggests a near-pCR. She will likely be placed on adjuvant T-DM1 (per KATHERINE trial) for any residual disease in breast/nodes, which further lowers recurrence risk. This systemic therapy will address microscopic disease in lymph nodes as well. If she had a mastectomy (no breast RT), we might consider a low axilla field due to lack of surgical staging beyond SLNs, but with lumpectomy and tangential RT, additional nodal treatment is unnecessary. Patient factors (age 60, good candidate for continued HER2 therapy) reinforce focusing on systemic therapy rather than invasive axillary surgery for an ITC.
CONFIDENCE_LEVEL: High – Clear staging definitions and outcome data support no escalation for ITCs. The combination of an excellent treatment response, effective systemic therapy (T-DM1), and radiation makes it highly certain that ALND or nodal RT would not improve her prognosis
ascopubs.org
pubmed.ncbi.nlm.nih.gov
.
WEB_SEARCH_USED: Yes – Looked up “HER2 residual isolated tumor cells axilla”. Found no specific concerns unique to HER2+ in this setting; general sources treat ITC as insignificant metastasis. A review of post-NACT outcomes
pubmed.ncbi.nlm.nih.gov
reinforced that patients with minimal nodal involvement (including HER2+) did well without ALND. This confirmed that “None” (no extra axillary therapy) is appropriate.
---END---

QUESTION_ID: 12
SELECTED_ANSWER: C

EVIDENCE_SUMMARY: For an HER2-positive patient with 1 of 4 SLNs showing a micrometastasis after NACT, further axillary surgery can be avoided in favor of regional nodal irradiation. One small residual node (especially a micromet) is a limited disease burden; evidence (mostly from other subtypes and retrospective series) indicates no survival or control benefit from ALND in such cases if radiation is given
pubmed.ncbi.nlm.nih.gov
. Given HER2+ tumors' high chemo-sensitivity and the availability of effective post-neoadjuvant therapy (e.g., T-DM1, which cuts recurrence risk), the priority is to minimize surgery without compromising control. By delivering nodal irradiation (to axilla and supraclavicular nodes) along with whole-breast RT, we address potential residual disease in remaining nodes similarly to ALND. This approach is supported by trials like AMAROS (in upfront node-positive patients) showing that radiation achieves comparable axillary control to ALND for low-volume disease, and by emerging post-NACT data suggesting ALND omission is feasible with comprehensive RT
pubmed.ncbi.nlm.nih.gov
.
KEY_REFERENCES: AMAROS trial (EORTC 2014) – demonstrated that axillary RT is equivalent to ALND in controlling regional disease for patients with 1–2 positive SLNs, with less morbidity; Zaborowski et al., 2025 – meta-analysis found no worse 5-yr OS or axillary recurrence when ALND was omitted in patients with limited nodal positivity after NACT, many of whom received nodal irradiation
pubmed.ncbi.nlm.nih.gov
; KATHERINE trial (2018) – showed T-DM1 markedly reduces invasive recurrence in HER2+ patients with residual disease (improving systemic control, indirectly supporting less aggressive local surgery in these cases).
GUIDELINE_ALIGNMENT: As of early 2025, standard guidelines would still call for ALND in an HER2+ patient with any nodal metastasis post-NACT. However, they acknowledge that if ALND is forgone, radiation should not be de-escalated. In practice, many multidisciplinary teams now manage a single positive node (even in HER2+ cases) with nodal irradiation instead of ALND, aligning with evolving ASCO/ASTRO guidance that accepts radiation in place of ALND for limited node-positive disease
ascopost.com
. Thus, while not yet universally codified for post-NACT HER2+, this recommendation is consistent with guideline trajectories in similar contexts.
SUBGROUP_NOTES: HER2-positive patients benefit from tailored systemic therapy (here, likely T-DM1 for residual disease), which lowers distant and regional relapse risk. The decision to skip ALND is bolstered by her excellent response (only one micromet) and the knowledge that she'll have ongoing HER2 therapy. If she had multiple nodes positive or if the SLN mapping was suboptimal, we'd lean towards ALND. But with 4 SLNs retrieved and minimal disease, nodal RT provides a less morbid yet effective strategy. Patient preferences also matter – many women prefer to avoid ALND's lymphedema risk if medically sound to do so. In this case, we can

confidently omit ALND with the safeguard of radiation.
CONFIDENCE_LEVEL: Moderate – We base this on strong rationale and extrapolated evidence, but direct trial evidence in HER2+ post-NACT patients is pending. The consistency of results in similar scenarios (luminal and TNBC with limited nodes)
pubmed.ncbi.nlm.nih.gov
 gives moderate confidence that HER2+ will fare similarly, especially with potent systemic therapy, but until prospective data confirm, a degree of caution remains.
WEB_SEARCH_USED: Yes – Queried "HER2 positive one node after neoadjuvant ALND or radiation". Found general support for ALND omission with radiation in low-volume disease (ASCO Post 2025, SENOMAC, etc.)
ascopost.com
. Also reviewed the 2025 meta-analysis showing no axillary control difference without ALND in mixed cohorts
pubmed.ncbi.nlm.nih.gov
. These informed the recommendation to use nodal irradiation instead of ALND in this HER2+ patient with a single micrometastatic node.
---END---

QUESTION_ID: 13
SELECTED_ANSWER: B
EVIDENCE_SUMMARY: In an HER2-positive patient with micrometastatic disease in 2 of 4 SLNs after NACT, a completion ALND is recommended. Two positive nodes – even if small – raise concern for additional nonsentinel node involvement, and current practice for HER2+ cancers (which often had significant initial nodal disease pre-NACT) is to ensure full clearance of the axilla surgically. While radiation could potentially manage residual disease, there is not yet prospective evidence in post-NACT HER2+ patients to forgo ALND for multiple positive nodes. Moreover, guidelines up to 2025 still endorse ALND for patients with more than one positive sentinel node after chemo. By performing ALND, we gain definitive nodal status (which can guide radiation decisions, e.g., extent of nodal fields) and remove any remaining disease, after which she will also receive regional nodal irradiation only if indicated by final nodal burden. This aggressive local approach is justified by the higher pre-treatment nodal involvement often seen in HER2+ tumors and ensures optimal local control in the absence of trial data for omission.
KEY_REFERENCES: NCCN Guidelines (2023) – advise ALND for ypN+ disease, especially if more than one node involved (no trials yet support omission in this setting)
sciencedirect.com
; ACOSOG Z1071 (2013) – highlighted that if SLN is positive post-NACT, residual disease in non-sentinel nodes is not negligible, thus ALND was standard in that study; Alliance A011202 trial (ongoing as of 2025) – specifically addresses if nodal RT can replace ALND for patients with positive SLNs after NACT, but results pending (so until then, ALND remains the standard). ([Note: Post-hoc analyses like Zaborowski 2025 suggest no survival loss without ALND
pubmed.ncbi.nlm.nih.gov
, but this is hypothesis-generating, not practice-changing yet])
GUIDELINE_ALIGNMENT: According to ASCO and NCCN guidelines through

early 2025, patients with multiple positive SLNs after neoadjuvant therapy should undergo ALND. There is allowance to avoid ALND in 1–2 node-positive cases for upfront surgery under certain conditions, but these do not yet extend to the post-NACT setting for multiple node involvement. Thus, recommending ALND aligns with current guideline-driven standard of care for this scenario.
SUBGROUP_NOTES: HER2-positive patients will receive powerful systemic therapy (T-DM1) for residual disease, which dramatically lowers recurrence risk. However, local management is approached similarly to other subtypes until evidence suggests otherwise. If the patient had reasons to avoid surgery (frailty, patient refusal), one could consider nodal radiation as an alternative, acknowledging it’s off-protocol. But at age 60 and presumably healthy, she can tolerate ALND. Notably, if ALND finds only those two micrometastatic nodes and nothing further, her nodal stage remains low (ypN1mi) – radiation oncologists might then tailor nodal irradiation (some might omit full nodal fields given tiny deposits and ALND done). These nuances require multidisciplinary input, but the initial step is to proceed with ALND for completeness.
CONFIDENCE_LEVEL: Moderate – This is in line with standard practice and guidelines, so moderately high confidence in recommending ALND. However, the level of evidence is not high-quality (no RCT proving ALND improves survival here), and emerging data hint ALND might be safely skipped
pubmed.ncbi.nlm.nih.gov
. Because of these evolving insights but lack of definitive proof, the confidence is moderate, erring on the side of established practice for patient safety.
WEB_SEARCH_USED: Yes – Searched “HER2 multiple positive sentinel nodes after neoadjuvant” and reviewed guidelines. Found that guidelines still favor ALND in this circumstance
sciencedirect.com
. Also noted pending trials (Alliance) and a 2025 systematic review suggesting possible safety of SLNB alone
pubmed.ncbi.nlm.nih.gov
, but without published results, standard of care remains ALND. These informed the recommendation of ALND for two positive nodes in an HER2+ patient.
---END---

QUESTION_ID: 14
SELECTED_ANSWER: B
EVIDENCE_SUMMARY: For a patient with ER-negative (triple-negative) breast cancer who has one sentinel node with macrometastatic residual disease (~3 mm, ypN1) after NACT, the recommended additional axillary therapy is a completion axillary lymph node dissection. This scenario indicates a significant residual nodal tumor deposit, and historically the standard is to remove remaining axillary nodes surgically. Trials in the upfront surgery setting (like ACOSOG Z0011) that omitted ALND were limited to patients receiving breast RT and only 1–2 positive nodes, but they did not include patients after NACT; for post-neoadjuvant residual macrometastasis, guidelines still call for ALND to ensure regional control

sciencedirect.com
. Triple-negative disease, in particular, has higher relapse risks when nodal disease remains, so most multidisciplinary teams favor the definitive clearance that ALND provides. Although axillary radiation will be given to the breast (and likely regional nodes if ALND reveals more disease), ALND offers pathologic assurance of axillary status and potentially reduces nodal recurrence.
KEY_REFERENCES: NCCN Guidelines (2023–24) – recommend ALND for any node-positive (ypN+) patient after NACT, given lack of prospective data for omission
sciencedirect.com
; Alliance A011202 trial – addressing ALND vs axillary RT for patients with positive SLN after NACT (results not yet available as of Feb 2025); ACOSOG Z0011 – showed no ALND needed in some upfront cases, but specifically excluded patients who received neoadjuvant chemo, so not directly applicable here. Retrospective data (e.g., MD Anderson 2017) show low regional failure rates with ALND + RT in ypN1, supporting the aggressive approach (versus unknowns if ALND omitted).
GUIDELINE_ALIGNMENT: Current ASCO/ASTRO/NCCN guidelines uniformly advise ALND for residual macrometastatic nodal disease after NACT, especially in ER– (triple-negative) cancers where no further locoregional de-escalation criteria exist. This recommendation is firmly in line with guideline-based practice as of early 2025 – any ypN1 (≥2 mm) warrants ALND in a patient undergoing breast-conserving therapy (with subsequent radiation).
SUBGROUP_NOTES: Triple-negative patients with residual disease often receive adjuvant capecitabine to improve systemic outcomes. Locally, since she’s ER– (no endocrine therapy), maximizing local control is critical; ALND followed by whole-breast RT (and likely regional nodal RT depending on final node count) provides that. If ALND finds only the one node positive (and others negative), radiation oncologists may tailor the fields (some might treat supraclavicular nodes due to initial nodal involvement). Patient factors: at 60, presumably healthy, she can tolerate ALND; in a frailer patient, one might consider nodal RT instead, but that would be off-protocol and less common for a macromet in TNBC.
CONFIDENCE_LEVEL: High – This recommendation follows standard oncologic practice and guidelines, so we are highly confident it’s the safest approach. The evidence is based on consensus and indirect data (since no RCT specifically tested omission in this setting), but given triple-negative biology and current expert agreement, there is high confidence that ALND is appropriate.
WEB_SEARCH_USED: Yes – Consulted “post-NAC positive sentinel node triple negative” and guidelines. NCCN/ASCO materials confirmed ALND is indicated
sciencedirect.com
. No new data up to Feb 2025 suggest otherwise for TNBC. Though a meta-analysis (2025) implies no OS difference without ALND in combined cohorts
pubmed.ncbi.nlm.nih.gov
, its authors still call for prospective validation. Thus, the search reinforced ALND as the standard for this patient.
---END---

QUESTION_ID: 15
SELECTED_ANSWER: B
EVIDENCE_SUMMARY: In an HER2-positive patient with one sentinel lymph node containing macrometastatic residual disease (~3 mm) after NACT, completion ALND is recommended. Despite a good clinical response, the presence of a sizable nodal metastasis (ypN1) indicates not all disease was eradicated. Standard practice (as of Feb 2025) for any post-NACT nodal macrometastasis is to perform ALND, as no trial has yet shown it's safe to omit in this setting
sciencedirect.com
. Removing the remaining axillary nodes provides accurate staging (e.g., how many nodes involved) which can influence radiation decisions and ensures debulking of any additional tumor. The patient will already receive whole-breast irradiation, but if we rely on radiation alone for an unsampled axilla, there's uncertainty – ALND gives pathological clearance. Moreover, while HER2-targeted therapy (T-DM1) will greatly reduce recurrence risk systemically, local management still follows surgical principles. Until ongoing trials (like Alliance) prove equivalence of nodal RT, ALND remains the prudent approach for an HER2+ ypN1 scenario.
KEY_REFERENCES: NCCN Guidelines (2024) – endorse ALND for patients with positive SLNs after neoadjuvant therapy in HER2+ disease as well
sciencedirect.com
; ASCO Guideline Update (early 2025) – in early-stage setting allows skipping ALND for 1–2 nodes if nodal RT given post-mastectomy
ascopost.com
, but that does not specifically address post-NACT, so by default ALND is advised here; KATHERINE trial (2019) – demonstrated 50% reduction in recurrence with T-DM1 vs trastuzumab in HER2+ residual disease, underscoring that systemic therapy can handle microscopic disease, but local therapy (surgery + RT) still adhered to ALND in trial settings for node-positive patients.
GUIDELINE_ALIGNMENT: Performing ALND aligns with current guidelines for management of residual nodal disease. Neither NCCN nor ASCO (as of Feb 2025) supports SLNB alone when a macroscopic nodal metastasis remains after chemo. Guidelines would direct that after ALND, the patient receive regional nodal irradiation if she has multiple positive nodes, or at least supraclavicular RT given initial nodal involvement (even if only one node remains positive). Our recommendation fits within these established guidelines.
SUBGROUP_NOTES: HER2+ patients differ from TNBC in that they have a targeted therapy (T-DM1) post-NAC which significantly improves outcomes, possibly mitigating some risk of leaving nodal disease. However, decisions on omitting ALND aren't yet subtype-specific and are generally conservative. If this patient strongly wished to avoid ALND, one could consider treating the axilla with comprehensive RT instead, knowing that retrospective analyses have been encouraging
pubmed.ncbi.nlm.nih.gov
– but this would be off-guideline. In a healthy 60-year-old, the safer bet is ALND. The ALND will also confirm if only 1 node vs more are involved; if it's truly just 1, and small, her subsequent radiation may be tailored (some centers might not boost axilla if

ALND done and minimal disease). These nuances depend on final pathology and multidisciplinary discussion.
CONFIDENCE_LEVEL: High – There is broad agreement in the oncology community about performing ALND in this scenario as of 2025. Although we acknowledge evolving research, until level I data emerge, confidence remains high that ALND is the correct and standard recommendation for an HER2+ patient with residual macrometastatic nodal disease.
WEB_SEARCH_USED: Yes – Searched “HER2 residual node after NACT guidelines” and reviewed ASCO/NCCN recommendations. Found that guidelines uniformly favor ALND here
sciencedirect.com
. Also reviewed literature for de-escalation; while promising (SENOMAC, meta-analyses)
ascopost.com
pubmed.ncbi.nlm.nih.gov
, those do not yet change practice for post-NACT macromet disease. Therefore, searches confirmed that ALND remains the advised approach.
---END---
