# Supplementary material for: Performance of large language models in ambiguous treatment scenarios of early breast cancer
Source: Breast. 2026 Sep 23;90:104932. doi: 10.1016/j.breast.2026.104932 (PMC13641045; doi:10.1016/j.breast.2026.104932)
Supplement: Supplementary file 1 [file mmc1.zip › Suppl.Mat. - Anton Oseledchyk - Prompts, LLM Responses, R-Code/axillary_response_claude.pdf]

QUESTION_ID: 1
SELECTED_ANSWER: C
EVIDENCE_SUMMARY: The SOUND and INSEMA trials demonstrated that omitting SLNB in postmenopausal women with small (<2cm), grade 1-2, ER+/HER2- tumors with negative axillary ultrasound maintains excellent outcomes. ASCO 2024 guidelines support SLNB omission for tumors ≤2 cm in this specific population receiving breast-conserving therapy.
KEY_REFERENCES: SOUND trial (JAMA Oncol 2023), INSEMA trial (NEJM 2024), ASCO Guidelines Update 2024
GUIDELINE_ALIGNMENT: ASCO 2024 guideline update specifically recommends against routine SLNB in postmenopausal women ≥50 years with grade 1-2, ≤2 cm, HR+/HER2- tumors
SUBGROUP_NOTES: Must be postmenopausal, ≥50 years, grade 1-2, planned breast conservation with radiation
CONFIDENCE_LEVEL: High
WEB_SEARCH_USED: Yes
---END---

QUESTION_ID: 2
SELECTED_ANSWER: G
EVIDENCE_SUMMARY: HER2-positive disease requires accurate nodal staging for adjuvant therapy decisions including trastuzumab duration and potential neratinib. Current guidelines maintain SLNB for all HER2-positive tumors regardless of size given the aggressive biology and treatment implications.
KEY_REFERENCES: NCCN Guidelines 2025, ASCO Guidelines 2024, HER2-positive treatment protocols
GUIDELINE_ALIGNMENT: Current guidelines do not support SLNB omission in HER2-positive disease
SUBGROUP_NOTES: HER2-positive status changes treatment paradigm requiring accurate staging
CONFIDENCE_LEVEL: High
WEB_SEARCH_USED: Yes
---END---

QUESTION_ID: 3
SELECTED_ANSWER: G
EVIDENCE_SUMMARY: Triple-negative breast cancer has aggressive biology with higher nodal involvement rates and requires accurate staging for chemotherapy decisions. No current trials support SLNB omission in TNBC, and guidelines recommend SLNB for all TNBC regardless of size.
KEY_REFERENCES: SOUND trial (excluded TNBC), INSEMA trial (limited TNBC), NCCN Guidelines 2025
GUIDELINE_ALIGNMENT: All guidelines recommend SLNB for TNBC given aggressive biology and treatment implications
SUBGROUP_NOTES: TNBC requires accurate staging for systemic therapy decisions
CONFIDENCE_LEVEL: High
WEB_SEARCH_USED: Yes
---END---

QUESTION_ID: 4

SELECTED_ANSWER: B
EVIDENCE_SUMMARY: Limited data exists for SLNB omission in invasive lobular carcinoma (ILC) as most trials focused on invasive ductal carcinoma. ILC has different metastatic patterns and higher rates of discordant imaging, warranting caution in applying IDC criteria.
KEY_REFERENCES: SOUND trial (limited ILC), INSEMA trial (primarily IDC), specific ILC studies lacking
GUIDELINE_ALIGNMENT: No specific guidelines address SLNB omission in ILC
SUBGROUP_NOTES: ILC has different growth patterns and nodal involvement compared to IDC
CONFIDENCE_LEVEL: Moderate
WEB_SEARCH_USED: Yes
---END---

QUESTION_ID: 5
SELECTED_ANSWER: A
EVIDENCE_SUMMARY: Isolated tumor cells (ITCs) are considered node-negative disease (pN0i+) and do not require additional axillary treatment. Standard practice is observation with breast radiation alone, as ITCs have minimal impact on prognosis.
KEY_REFERENCES: AJCC 8th edition staging, ACOSOG Z0010, NCCN Guidelines 2025
GUIDELINE_ALIGNMENT: All guidelines classify ITCs as pN0i+ requiring no additional axillary treatment
SUBGROUP_NOTES: ITCs defined as clusters ≤0.2 mm
CONFIDENCE_LEVEL: High
WEB_SEARCH_USED: Yes
---END---

QUESTION_ID: 6
SELECTED_ANSWER: C
EVIDENCE_SUMMARY: Single micrometastatic disease (0.2-2mm) after neoadjuvant chemotherapy in ER+/HER2- disease warrants nodal irradiation based on AMAROS principles. The residual disease indicates incomplete response, and radiation provides equivalent control to ALND with less morbidity.
KEY_REFERENCES: AMAROS trial, Alliance A011202, NCCN Guidelines 2025
GUIDELINE_ALIGNMENT: Guidelines support nodal radiation for micrometastatic disease post-neoadjuvant therapy
SUBGROUP_NOTES: Post-neoadjuvant setting differs from upfront surgery scenarios
CONFIDENCE_LEVEL: Moderate
WEB_SEARCH_USED: Yes
---END---

QUESTION_ID: 7
SELECTED_ANSWER: C
EVIDENCE_SUMMARY: Two micrometastatic nodes after neoadjuvant chemotherapy indicates higher residual disease burden requiring nodal irradiation. This exceeds typical ACOSOG Z0011 criteria in the neoadjuvant setting, supporting radiation over ALND.
KEY_REFERENCES: AMAROS trial, Alliance A011202, expert consensus
GUIDELINE_ALIGNMENT: Guidelines support nodal radiation for multiple

positive nodes post-neoadjuvant therapy
SUBGROUP_NOTES: Higher burden (2 nodes) increases recurrence risk
CONFIDENCE_LEVEL: Moderate
WEB_SEARCH_USED: Yes
---END---

QUESTION_ID: 8
SELECTED_ANSWER: A
EVIDENCE_SUMMARY: ITCs in TNBC are still considered pN0i+ and do not require additional axillary treatment despite the aggressive subtype. Standard management remains observation with planned breast radiation.
KEY_REFERENCES: AJCC staging, ACOSOG Z0010, NCCN Guidelines 2025
GUIDELINE_ALIGNMENT: ITCs classified as node-negative regardless of tumor subtype
SUBGROUP_NOTES: TNBC biology doesn't change ITC management
CONFIDENCE_LEVEL: High
WEB_SEARCH_USED: Yes
---END---

QUESTION_ID: 9
SELECTED_ANSWER: C
EVIDENCE_SUMMARY: Micrometastatic disease in TNBC after neoadjuvant chemotherapy indicates incomplete response in aggressive disease, warranting nodal irradiation. The residual disease in TNBC carries higher risk than ER+ disease.
KEY_REFERENCES: CREATE-X implications, AMAROS principles, expert consensus
GUIDELINE_ALIGNMENT: Nodal radiation preferred for residual disease in TNBC
SUBGROUP_NOTES: TNBC with residual disease has poor prognosis requiring aggressive local control
CONFIDENCE_LEVEL: Moderate
WEB_SEARCH_USED: Yes
---END---

QUESTION_ID: 10
SELECTED_ANSWER: C
EVIDENCE_SUMMARY: Two micrometastatic nodes in TNBC after neoadjuvant therapy represents significant residual disease burden requiring nodal irradiation. The aggressive biology and multiple positive nodes support comprehensive radiation.
KEY_REFERENCES: AMAROS trial extension, Alliance A011202, expert consensus
GUIDELINE_ALIGNMENT: Multiple positive nodes post-NAC warrant radiation therapy
SUBGROUP_NOTES: High-risk scenario with TNBC and multiple positive nodes
CONFIDENCE_LEVEL: Moderate
WEB_SEARCH_USED: Yes
---END---

QUESTION_ID: 11
SELECTED_ANSWER: A

EVIDENCE_SUMMARY: ITCs in HER2-positive disease remain classified as pN0i+ requiring no additional axillary treatment. The excellent prognosis with HER2-targeted therapy and ITCs supports observation.
KEY_REFERENCES: AJCC staging, HER2-positive treatment guidelines, ACOSOG Z0010
GUIDELINE_ALIGNMENT: ITCs managed as node-negative regardless of HER2 status
SUBGROUP_NOTES: HER2-targeted therapy provides excellent systemic control
CONFIDENCE_LEVEL: High
WEB_SEARCH_USED: Yes
---END---

QUESTION_ID: 12
SELECTED_ANSWER: C
EVIDENCE_SUMMARY: Micrometastatic disease in HER2-positive cancer after neoadjuvant therapy (including anti-HER2 treatment) indicates incomplete response warranting nodal irradiation. Radiation provides local control equivalent to ALND with less morbidity.
KEY_REFERENCES: AMAROS trial, NSABP B-51/RTOG 1304, expert consensus
GUIDELINE_ALIGNMENT: Nodal radiation preferred for residual disease post-NAC
SUBGROUP_NOTES: Residual disease despite HER2-targeted therapy indicates resistance
CONFIDENCE_LEVEL: Moderate
WEB_SEARCH_USED: Yes
---END---

QUESTION_ID: 13
SELECTED_ANSWER: C
EVIDENCE_SUMMARY: Two micrometastatic nodes in HER2-positive disease after neoadjuvant therapy represents higher burden residual disease requiring nodal irradiation. Multiple positive nodes exceed typical observation criteria.
KEY_REFERENCES: AMAROS extension, NSABP B-51, expert consensus
GUIDELINE_ALIGNMENT: Multiple positive nodes warrant radiation therapy
SUBGROUP_NOTES: Higher nodal burden increases recurrence risk despite HER2-targeted therapy
CONFIDENCE_LEVEL: Moderate
WEB_SEARCH_USED: Yes
---END---

QUESTION_ID: 14
SELECTED_ANSWER: C
EVIDENCE_SUMMARY: Macrometastatic disease (>2mm) in ER-negative disease after neoadjuvant chemotherapy warrants nodal irradiation based on AMAROS principles and Alliance A011202 trial design. Radiation provides equivalent control to ALND with reduced morbidity.
KEY_REFERENCES: AMAROS trial, Alliance A011202, NCCN Guidelines 2025
GUIDELINE_ALIGNMENT: Guidelines support nodal radiation for macrometastatic disease post-NAC
SUBGROUP_NOTES: ER-negative disease requires optimal local control

given limited systemic options
CONFIDENCE_LEVEL: High
WEB_SEARCH_USED: Yes
---END---

QUESTION_ID: 15
SELECTED_ANSWER: C
EVIDENCE_SUMMARY: Macrometastatic disease in HER2-positive cancer after neoadjuvant therapy indicates incomplete pathologic response despite anti-HER2 treatment, warranting nodal irradiation. AMAROS principles support radiation over ALND for equivalent control with less morbidity.
KEY_REFERENCES: AMAROS trial, NSABP B-51/RTOG 1304, Alliance A011202
GUIDELINE_ALIGNMENT: Nodal radiation preferred for residual macrometastatic disease
SUBGROUP_NOTES: Residual disease despite optimal systemic therapy indicates need for comprehensive local control
CONFIDENCE_LEVEL: High
WEB_SEARCH_USED: Yes
---END---
