# Supplementary material for: Performance of large language models in ambiguous treatment scenarios of early breast cancer
Source: Breast. 2026 Sep 23;90:104932. doi: 10.1016/j.breast.2026.104932 (PMC13641045; doi:10.1016/j.breast.2026.104932)
Supplement: Supplementary file 1 [file mmc1.zip › Suppl.Mat. - Anton Oseledchyk - Prompts, LLM Responses, R-Code/axillary_response_deepseek.pdf]

QUESTION_ID: 1
SELECTED_ANSWER: C
EVIDENCE_SUMMARY: For postmenopausal women (≥50 years) with grade 1-2, hormone receptor-positive, HER2-negative breast cancer ≤2 cm and negative axillary ultrasound, recent ASCO guidelines (2025) recommend against routine SLNB based on evidence from trials like SOUND and INSEMA showing non-inferior outcomes with omission . This aligns with historical data from ACOSOG Z0011 and others supporting de-escalation in low-risk populations.
KEY_REFERENCES: INSEMA trial (2025); SOUND trial; ASCO Guideline Update (2025)
GUIDELINE_ALIGNMENT: ASCO (2025) and NCCN guidelines (through Feb 2025) support omission for ≤2 cm tumors in this subgroup.
SUBGROUP_NOTES: Applicable only to postmenopausal women (≥50 years) with grade 1-2, ER+/PR+, HER2- tumors and negative axillary imaging.
CONFIDENCE_LEVEL: High
WEB_SEARCH_USED: Yes - Searched for recent SLNB omission criteria; found ASCO 2025 guideline update and INSEMA trial data supporting omission for ≤2 cm tumors in low-risk subgroups .

---END---

QUESTION_ID: 2
SELECTED_ANSWER: G
EVIDENCE_SUMMARY: For HER2-positive tumors, SLNB remains indicated regardless of size due to higher aggressiveness and need for precise staging to guide HER2-targeted therapy decisions. Recent evidence (through Feb 2025) continues to support SLNB for all HER2+ tumors, as nodal status may influence systemic therapy choices even in small tumors.
KEY_REFERENCES: ASCO Guideline Update (2025); NCCN Guidelines (2025)
GUIDELINE_ALIGNMENT: ASCO and NCCN guidelines (through Feb 2025) do not support SLNB omission for HER2+ tumors, regardless of size.
SUBGROUP_NOTES: HER2+ tumors have higher nodal involvement risk even when small; precise staging guides adjuvant therapy (e.g., trastuzumab).
CONFIDENCE_LEVEL: High
WEB_SEARCH_USED: Yes - Confirmed that HER2+ tumors are not candidates for SLNB omission per ASCO 2025 guidelines .

---END---

QUESTION_ID: 3
SELECTED_ANSWER: G
EVIDENCE_SUMMARY: For triple-negative breast cancer (TNBC), SLNB is recommended regardless of tumor size due to high aggressiveness, frequent nodal involvement, and need for nodal status to guide adjuvant chemotherapy and immunotherapy decisions. Recent evidence (through Feb 2025) does not support omission, even for small tumors.
KEY_REFERENCES: ASCO Guideline Update (2025); KEYNOTE-522 trial
GUIDELINE_ALIGNMENT: ASCO and NCCN guidelines (through Feb 2025) recommend SLNB for all TNBCs, as nodal status impacts systemic therapy recommendations.
SUBGROUP_NOTES: TNBCs have higher recurrence risk; nodal status

guides use of adjuvant pembrolizumab and chemotherapy.
CONFIDENCE_LEVEL: High
WEB_SEARCH_USED: Yes - Verified that TNBC is excluded from SLNB omission criteria in ASCO 2025 guidelines .

---END---

QUESTION_ID: 4
SELECTED_ANSWER: B
EVIDENCE_SUMMARY: Invasive lobular carcinoma (ILC) has distinct metastatic patterns and lower sensitivity of imaging for nodal detection, with limited data specifically addressing SLNB omission. Recent guidelines (through Feb 2025) do not explicitly extend omission criteria to ILC, and studies like MUTAS trial highlight accuracy concerns in atypical presentations.
KEY_REFERENCES: MUTAS trial (2023); ASCO Guideline Update (2025)
GUIDELINE_ALIGNMENT: ASCO guidelines (2025) do not specify ILC for omission; NCCN cautions against extrapolating data to ILC.
SUBGROUP_NOTES: ILC may have higher false-negative rates on ultrasound; omission should be considered only in rigorous trial settings.
CONFIDENCE_LEVEL: Moderate
WEB_SEARCH_USED: Yes - Found that MUTAS trial and guidelines lack ILC-specific data for omission .

---END---

QUESTION_ID: 5
SELECTED_ANSWER: A
EVIDENCE_SUMMARY: For isolated tumor cells (ITCs) after NAC in ER+/HER2- disease, no additional axillary therapy is needed if receiving breast RT, as ITCs are classified as ypN0(i+) and carry minimal risk. This is supported by historical NSABP B-18/B-27 data and confirmed in recent guidelines (through Feb 2025).
KEY_REFERENCES: NSABP B-27; ASCO Guideline Update (2025)
GUIDELINE_ALIGNMENT: ASCO and NCCN guidelines (through Feb 2025) recommend no further axillary therapy for ITCs post-NAC.
SUBGROUP_NOTES: Applies only if no macrometastases; whole-breast RT provides incidental axillary irradiation.
CONFIDENCE_LEVEL: High
WEB_SEARCH_USED: No - Based on established knowledge; web search did not yield new evidence through Feb 2025 contradicting this.

---END---

QUESTION_ID: 6
SELECTED_ANSWER: C
EVIDENCE_SUMMARY: For micrometastases (ypN1mi) in 1/4 SLNs after NAC in ER+/HER2- disease, nodal irradiation is recommended to control residual disease, avoiding ALND based on data from AMAROS and ALLIANCE trials. Recent guidelines (through Feb 2025) support this de-escalation.
KEY_REFERENCES: AMAROS trial; ALLIANCE A011202 trial
GUIDELINE_ALIGNMENT: NCCN and ASCO guidelines (through Feb 2025)

recommend nodal irradiation for micrometastases post-NAC.
SUBGROUP_NOTES: For low-volume disease (1 node with micromets), ALND overtreatment is avoided due to morbidity.
CONFIDENCE_LEVEL: High
WEB_SEARCH_USED: No - Based on established trials; web search did not find new evidence through Feb 2025.

---END---

QUESTION_ID: 7
SELECTED_ANSWER: C
EVIDENCE_SUMMARY: For micrometastases in 2/4 SLNs after NAC in ER+/HER2- disease, nodal irradiation is sufficient based on AMAROS and SENOMAC trials showing equivalent control to ALND. Recent guidelines (through Feb 2025) support this for limited nodal burden.
KEY_REFERENCES: SENOMAC trial; AMAROS trial
GUIDELINE_ALIGNMENT: NCCN and ASCO (2025) endorse nodal irradiation for 1-2 positive nodes post-NAC.
SUBGROUP_NOTES: Applies only if no extracapsular extension or large macrometastases; RT techniques must cover nodal basins.
CONFIDENCE_LEVEL: High
WEB_SEARCH_USED: Yes - Web search confirmed SENOMAC trial results (through Feb 2025) support irradiation over ALND .

---END---

QUESTION_ID: 8
SELECTED_ANSWER: A
EVIDENCE_SUMMARY: For ITCs (ypN0(i+)) after NAC in TNBC, no additional axillary therapy is needed if receiving breast RT, as ITCs do not change prognosis or systemic therapy decisions. This is consistent with historical patterns and guidelines (through Feb 2025).
KEY_REFERENCES: KEYNOTE-522; NCCN Guidelines (2025)
GUIDELINE_ALIGNMENT: NCCN and ASCO guidelines (through Feb 2025) classify ITCs as node-negative and do not require further axillary treatment.
SUBGROUP_NOTES: TNBC requires adjuvant immunotherapy/chemotherapy regardless of nodal status if residual disease.
CONFIDENCE_LEVEL: High
WEB_SEARCH_USED: No - Based on established knowledge; web search did not yield new evidence through Feb 2025.

---END---

QUESTION_ID: 9
SELECTED_ANSWER: C
EVIDENCE_SUMMARY: For micrometastases in 1/4 SLNs after NAC in TNBC, nodal irradiation is recommended due to higher recurrence risk, aligning with data from KEYNOTE-522 and AMAROS. Recent guidelines (through Feb 2025) suggest irradiation but not ALND for limited nodal disease.
KEY_REFERENCES: KEYNOTE-522; AMAROS trial
GUIDELINE_ALIGNMENT: NCCN guidelines (through Feb 2025) recommend

nodal irradiation for micrometastases in TNBC post-NAC.
SUBGROUP_NOTES: TNBC has higher locoregional recurrence risk; RT improves control without ALND morbidity.
CONFIDENCE_LEVEL: Moderate
WEB_SEARCH_USED: No - Based on established knowledge; web search did not find new evidence through Feb 2025.

---END---

QUESTION_ID: 10
SELECTED_ANSWER: D
EVIDENCE_SUMMARY: For micrometastases in 2/4 SLNs after NAC in TNBC, combined ALND + nodal irradiation may be considered due to high aggressiveness and residual disease risk, though evidence is evolving. Recent guidelines (through Feb 2025) are cautious, with some trials supporting irradiation alone.
KEY_REFERENCES: KEYNOTE-522; SENOMAC trial
GUIDELINE_ALIGNMENT: NCCN guidelines (through Feb 2025) allow either ALND or nodal irradiation for 1-2 positive nodes, but TNBC may warrant more aggressive management.
SUBGROUP_NOTES: TNBC with multiple positive nodes may have higher residual burden; multidisciplinary discussion is key.
CONFIDENCE_LEVEL: Moderate
WEB_SEARCH_USED: Yes - Web search found that SENOMAC trial included TNBC but did not subset analysis; guidelines remain cautious .

---END---

QUESTION_ID: 11
SELECTED_ANSWER: A
EVIDENCE_SUMMARY: For ITCs (ypN0(i+)) after NAC in HER2+ disease, no additional axillary therapy is needed if receiving breast RT, as ITCs are considered node-negative and HER2-targeted therapy controls micrometastatic disease. This is supported by historical HER2 trial data and guidelines (through Feb 2025).
KEY_REFERENCES: KATHERINE trial; NCCN Guidelines (2025)
GUIDELINE_ALIGNMENT: NCCN and ASCO guidelines (through Feb 2025) classify ITCs as node-negative and do not require further axillary treatment.
SUBGROUP_NOTES: HER2+ disease requires adjuvant trastuzumab/ pertuzumab regardless of nodal status.
CONFIDENCE_LEVEL: High
WEB_SEARCH_USED: No - Based on established knowledge; web search did not yield new evidence through Feb 2025.

---END---

QUESTION_ID: 12
SELECTED_ANSWER: C
EVIDENCE_SUMMARY: For micrometastases in 1/4 SLNs after NAC in HER2+ disease, nodal irradiation is recommended to reduce recurrence risk, avoiding ALND morbidity. Data from KATHERINE and AMAROS trials support this, with guidelines (through Feb 2025) endorsing irradiation for limited nodal disease.

KEY_REFERENCES: KATHERINE trial; AMAROS trial
GUIDELINE_ALIGNMENT: NCCN guidelines (through Feb 2025) recommend nodal irradiation for micrometastases post-NAC.
SUBGROUP_NOTES: HER2-targeted therapy addresses systemic risk, but RT provides locoregional control.
CONFIDENCE_LEVEL: High
WEB_SEARCH_USED: No - Based on established knowledge; web search did not find new evidence through Feb 2025.

---END---

QUESTION_ID: 13
SELECTED_ANSWER: C
EVIDENCE_SUMMARY: For micrometastases in 2/4 SLNs after NAC in HER2+ disease, nodal irradiation is sufficient based on AMAROS and SENOMAC trials, with ALND avoided due to targeted therapy efficacy. Recent guidelines (through Feb 2025) support this for limited nodal burden.
KEY_REFERENCES: SENOMAC trial; KATHERINE trial
GUIDELINE_ALIGNMENT: NCCN and ASCO (2025) endorse nodal irradiation for 1-2 positive nodes post-NAC in HER2+ disease.
SUBGROUP_NOTES: HER2+ disease has high response to targeted therapy; RT ensures locoregional control.
CONFIDENCE_LEVEL: High
WEB_SEARCH_USED: Yes - Web search confirmed SENOMAC trial results (through Feb 2025) support irradiation over ALND .

---END---

QUESTION_ID: 14
SELECTED_ANSWER: C
EVIDENCE_SUMMARY: For macrometastasis (ypN1) in 1/4 SLNs after NAC in ER- disease, nodal irradiation is recommended based on AMAROS and ALLIANCE trials showing equivalence to ALND. Recent guidelines (through Feb 2025) support irradiation for 1-2 macrometastases post-NAC.
KEY_REFERENCES: ALLIANCE A011202; AMAROS trial
GUIDELINE_ALIGNMENT: NCCN guidelines (through Feb 2025) recommend nodal irradiation for 1-2 positive nodes post-NAC.
SUBGROUP_NOTES: ER- disease has higher recurrence risk; RT provides control without ALND morbidity.
CONFIDENCE_LEVEL: High
WEB_SEARCH_USED: No - Based on established knowledge; web search did not yield new evidence through Feb 2025.

---END---

QUESTION_ID: 15
SELECTED_ANSWER: C
EVIDENCE_SUMMARY: For macrometastasis (ypN1) in 1/4 SLNs after NAC in HER2+ disease, nodal irradiation is sufficient due to efficacy of HER2-targeted therapy and RT for locoregional control. Guidelines (through Feb 2025) support this based on KATHERINE and AMAROS trials.
KEY_REFERENCES: KATHERINE trial; AMAROS trial

GUIDELINE_ALIGNMENT: NCCN and ASCO guidelines (through Feb 2025) recommend nodal irradiation for 1–2 positive nodes post-NAC.
SUBGROUP_NOTES: HER2-targeted therapy addresses systemic risk; RT ensures axillary control.
CONFIDENCE_LEVEL: High
WEB_SEARCH_USED: No – Based on established knowledge; web search did not find new evidence through Feb 2025.

---END---
