# Supplementary material for: Performance of large language models in ambiguous treatment scenarios of early breast cancer
Source: Breast. 2026 Sep 23;90:104932. doi: 10.1016/j.breast.2026.104932 (PMC13641045; doi:10.1016/j.breast.2026.104932)
Supplement: Supplementary file 1 [file mmc1.zip › Suppl.Mat. - Anton Oseledchyk - Prompts, LLM Responses, R-Code/axillary_response_gemini.pdf]

QUESTION_ID: 1
SELECTED_ANSWER: A
EVIDENCE_SUMMARY: Standard of care for early-stage breast cancer is to perform sentinel lymph node biopsy (SLNB). However, for a very specific, low-risk patient population, omission of SLNB can be considered. Evidence and guideline updates through early 2025 support this for patients with small (T1a/T1b, ≤1.0 cm), clinically node-negative, hormone receptor-positive tumors, particularly in older patients. Omission is not recommended for larger tumors due to an increased risk of nodal involvement.
KEY_REFERENCES: NCCN Guidelines for Breast Cancer (versions through Feb 2025); ASCO Guidelines on Axillary Management in Early-Stage Breast Cancer (updates through Feb 2025); emerging data from trials such as MAGNIFICENT (abstract data from SABCS 2024).
GUIDELINE_ALIGNMENT: NCCN Breast Cancer Guidelines (v1.2024) and ASCO guidelines, which suggest considering omission of SLNB for patients aged >70 with cT1, cN0, ER-positive, HER2-negative breast cancer who are receiving endocrine therapy. The size criterion of ≤1.0 cm corresponds to T1a/T1b.
SUBGROUP_NOTES: Omission is highly selective and typically limited to older patients (>70 years) with very small, low-grade, cN0, ER-positive, HER2-negative tumors who will be treated with endocrine therapy. The patient's age (60) is borderline, but the size criteria remain paramount for any consideration of omission.
CONFIDENCE_LEVEL: High
WEB_SEARCH_USED: Yes - Searched for "NCCN guidelines omission of SLNB breast cancer," "ASCO guidelines sentinel lymph node biopsy ER+," and "MAGNIFICENT trial results SABCS 2024." Found that NCCN and ASCO support this de-escalation for T1a/T1b tumors in select older patients, and that this remains a topic of ongoing research.
---END---
QUESTION_ID: 2
SELECTED_ANSWER: G
EVIDENCE_SUMMARY: HER2-positive breast cancer is associated with a higher risk of axillary lymph node metastasis compared to ER+/HER2- disease, even at small tumor sizes. The standard of care is to perform sentinel lymph node biopsy (SLNB) regardless of tumor size to accurately stage the axilla. There is no established evidence or guideline recommendation through February 2025 to support omitting SLNB in this subtype.
KEY_REFERENCES: NCCN Breast Cancer Guidelines; ASCO guidelines on axillary management; multiple retrospective and prospective studies on HER2-positive breast cancer nodal positivity rates.
GUIDELINE_ALIGNMENT: NCCN Breast Cancer Guidelines (versions through Feb 2025) and ASCO guidelines universally recommend axillary staging via SLNB for cT1-T2, cN0 breast cancer, including the HER2-positive subtype.
SUBGROUP_NOTES: The HER2-positive subtype has a more aggressive biology and is treated with systemic targeted therapy, for which accurate nodal staging is crucial for determining prognosis and guiding further treatment decisions.
CONFIDENCE_LEVEL: High
WEB_SEARCH_USED: Yes - Searched for "omission of sentinel lymph node biopsy HER2 positive breast cancer." Confirmed that no guideline or

trial data up to February 2025 supports this practice.
---END---
QUESTION_ID: 3
SELECTED_ANSWER: G
EVIDENCE_SUMMARY: Triple-negative breast cancer (TNBC) is an aggressive subtype with a significant risk of nodal involvement, even with small primary tumors. Accurate axillary staging via sentinel lymph node biopsy (SLNB) is essential for guiding treatment decisions and prognosis. There is no evidence or guideline recommendation through February 2025 to support omitting SLNB in this patient population.
KEY_REFERENCES: NCCN Breast Cancer Guidelines; ASCO guidelines on axillary management; data on nodal positivity rates in TNBC.
GUIDELINE_ALIGNMENT: NCCN Breast Cancer Guidelines (versions through Feb 2025) and ASCO guidelines strongly recommend axillary staging via SLNB for cT1-T2, cN0 breast cancer, including the triple-negative subtype, to determine the extent of disease.
SUBGROUP_NOTES: TNBC has a more aggressive natural history compared to luminal subtypes. Omitting axillary staging would compromise treatment planning and risk management, making SLNB mandatory regardless of tumor size.
CONFIDENCE_LEVEL: High
WEB_SEARCH_USED: Yes - Searched for "omission of sentinel lymph node biopsy triple negative breast cancer." Confirmed that no evidence or guidelines up to February 2025 support omitting SLNB in TNBC.
---END---
QUESTION_ID: 4
SELECTED_ANSWER: B
EVIDENCE_SUMMARY: The criteria for omitting sentinel lymph node biopsy (SLNB) for invasive lobular carcinoma (ILC) should not be the same as for invasive ductal carcinoma (IDC). ILC is known for its diffuse, multifocal growth pattern and can be more difficult to detect on imaging, including axillary ultrasound. As a result, there is less confidence in the accuracy of clinical staging, and there is limited specific data on de-escalation of SLNB for ILC compared to the more common IDC subtype.
KEY_REFERENCES: Retrospective studies and case series on imaging and nodal metastatic patterns of ILC vs. IDC; NCCN and ASCO guidelines on axillary management, which do not distinguish the two but acknowledge the imaging challenges of ILC.
GUIDELINE_ALIGNMENT: While standard guidelines don't explicitly separate IDC and ILC for SLNB, their recommendation to proceed with SLNB is based on the highest standard of care, reflecting the lack of data to justify a change for ILC. This aligns with the principle of being more cautious when data is limited.
SUBGROUP_NOTES: The unique diffuse growth and imaging characteristics of ILC make clinical nodal assessment less reliable, thus warranting a more cautious approach to axillary staging.
CONFIDENCE_LEVEL: High
WEB_SEARCH_USED: Yes - Searched "sentinel lymph node biopsy ILC vs IDC," and "omission of SLNB invasive lobular carcinoma." Found that ILC is often excluded from or underrepresented in de-escalation trials, confirming the limited data and the need for caution.
---END---

QUESTION_ID: 5
SELECTED_ANSWER: A
EVIDENCE_SUMMARY: The patient has a clinically positive response to neoadjuvant chemotherapy (NAC), and the sentinel lymph node (SLN) biopsy shows isolated tumor cells (ITCs), which are considered a negative node (ypN0i+). The ACOSOG Z0011 trial established that in the setting of breast-conserving surgery and whole-breast radiation, an axillary lymph node dissection (ALND) is not needed for patients with up to two positive lymph nodes. ITCs are not considered to be a positive node for this recommendation. Therefore, no additional axillary therapy is needed.
KEY_REFERENCES: ACOSOG Z0011 trial (JAMA 2011, JCO 2017); NCCN Breast Cancer Guidelines (versions through Feb 2025).
GUIDELINE_ALIGNMENT: NCCN Breast Cancer Guidelines (versions through Feb 2025) consider ITCs (pN0i+) to be equivalent to pN0 for axillary management. The Z0011 trial demonstrated that ALND provides no additional benefit in this low-risk setting.
SUBGROUP_NOTES: This recommendation holds true for all subtypes (ER+, TN, HER2+) as the Z0011 trial included various subtypes.
CONFIDENCE_LEVEL: High
WEB_SEARCH_USED: Yes - Searched for "ACOSOG Z0011 isolated tumor cells," and "NCCN guidelines axillary management ITCs." The search confirmed that ITCs are categorized as ypN0i+ and generally do not warrant further axillary therapy.
---END---
QUESTION_ID: 6
SELECTED_ANSWER: C
EVIDENCE_SUMMARY: The patient has micrometastatic residual disease (ypN1mi, 1 of 4 SLN) after neoadjuvant chemotherapy (NAC) and is planned for radiation therapy (RT) to the breast. The Alliance A011202 trial showed that for patients with cN+ disease who convert to ypN0 or ypN1 (1-2 positive nodes) after NAC, axillary RT is non-inferior to ALND in terms of disease control. Therefore, the addition of nodal irradiation to breast RT is the recommended approach to cover the axillary nodal basin without the need for ALND.
KEY_REFERENCES: Alliance A011202 trial (JAMA Oncol 2023); NSABP B-51 trial (presented data supporting RT over ALND).
GUIDELINE_ALIGNMENT: NCCN Breast Cancer Guidelines (versions through Feb 2025) and ASCO guidelines, updated to reflect the Alliance and NSABP B-51 trial data, now recommend axillary nodal radiation as an alternative to ALND for patients who have a limited number of positive nodes after NAC.
SUBGROUP_NOTES: This recommendation is applicable across all subtypes and is specific to patients who are receiving breast RT.
CONFIDENCE_LEVEL: High
WEB_SEARCH_USED: Yes - Searched "Alliance A011202 trial results," "NSABP B-51 trial," and "NCCN guidelines axillary management post NAC." The search confirmed that the results of these trials were published and incorporated into guidelines before February 2025.
---END---
QUESTION_ID: 7
SELECTED_ANSWER: C
EVIDENCE_SUMMARY: The patient has micrometastatic residual disease

in 2 of 4 sentinel lymph nodes (ypN1mi). This scenario is well-addressed by the Alliance A011202 trial, which demonstrated that for patients who were cN+ and converted to ypN0/ypN1 after neoadjuvant chemotherapy, axillary RT provides a safe alternative to axillary lymph node dissection (ALND). The primary tumor subtype does not change this recommendation. Therefore, nodal irradiation is recommended in addition to breast RT.
KEY_REFERENCES: Alliance A011202 trial (JAMA Oncol 2023); NSABP B-51 trial.
GUIDELINE_ALIGNMENT: NCCN and ASCO guidelines (up to Feb 2025) incorporate data from trials like Alliance A011202, supporting axillary RT as an alternative to ALND for patients with a limited number of positive nodes after NAC.
SUBGROUP_NOTES: This recommendation applies to patients of all subtypes receiving breast-conserving surgery and breast RT.
CONFIDENCE_LEVEL: High
WEB_SEARCH_USED: Yes - Searched "Alliance A011202 micrometastasis," and "guidelines for axillary radiation post-NAC." The search confirmed that the trial and subsequent guideline updates support this approach.
---END---
QUESTION_ID: 8
SELECTED_ANSWER: A
EVIDENCE_SUMMARY: The patient has a clinically positive response to neoadjuvant chemotherapy (NAC), and the sentinel lymph node (SLN) biopsy shows isolated tumor cells (ITCs), which are considered a negative node (ypN0i+). Based on the ACOSOG Z0011 trial, which showed no benefit from ALND for patients with up to two positive nodes undergoing breast-conserving surgery and whole-breast radiation, ITCs do not warrant additional axillary therapy. This is consistent across all subtypes, including triple-negative breast cancer.
KEY_REFERENCES: ACOSOG Z0011 trial (JAMA 2011, JCO 2017); NCCN Breast Cancer Guidelines (versions through Feb 2025).
GUIDELINE_ALIGNMENT: NCCN guidelines (up to Feb 2025) and ASCO recommendations align with Z0011, treating ITCs as a negative node for purposes of axillary management.
SUBGROUP_NOTES: The biological subtype does not alter the management of ITCs in the axilla.
CONFIDENCE_LEVEL: High
WEB_SEARCH_USED: Yes - Confirmed the Z0011 findings regarding ITCs and their integration into major guidelines for breast cancer care up to February 2025.
---END---
QUESTION_ID: 9
SELECTED_ANSWER: C
EVIDENCE_SUMMARY: The patient has micrometastatic residual disease (ypN1mi, 1 of 4 SLN) after neoadjuvant chemotherapy (NAC). The Alliance A011202 trial demonstrated that axillary nodal irradiation is a safe and effective alternative to axillary lymph node dissection (ALND) for patients who convert from cN+ to ypN0/ypN1 after NAC. This holds true for all subtypes, including triple-negative breast cancer. Since the patient is receiving breast radiation therapy, nodal irradiation is the recommended additional

therapy.
KEY_REFERENCES: Alliance A011202 trial (JAMA Oncol 2023); NSABP B-51 trial.
GUIDELINE_ALIGNMENT: NCCN Breast Cancer Guidelines and ASCO guidelines (through Feb 2025) now support nodal RT as an alternative to ALND in this specific post-NAC scenario, based on the non-inferiority shown in Alliance A011202.
SUBGROUP_NOTES: The recommendation is independent of the tumor subtype for this post-NAC staging.
CONFIDENCE_LEVEL: High
WEB_SEARCH_USED: Yes - Confirmed that the Alliance A011202 trial results for this specific scenario and their inclusion in guidelines were well-established prior to March 2025.
---END---
QUESTION_ID: 10
SELECTED_ANSWER: C
EVIDENCE_SUMMARY: The patient has micrometastatic residual disease in 2 of 4 sentinel lymph nodes (ypN1mi) after neoadjuvant chemotherapy. The Alliance A011202 trial, a landmark study in this setting, demonstrated that for patients with limited residual nodal disease (1-2 positive nodes) after NAC, axillary radiation therapy (RT) is non-inferior to axillary lymph node dissection (ALND) in terms of survival outcomes. Therefore, nodal irradiation is the standard of care for additional axillary therapy.
KEY_REFERENCES: Alliance A011202 trial (JAMA Oncol 2023); NSABP B-51 trial.
GUIDELINE_ALIGNMENT: This approach is directly supported by NCCN and ASCO guidelines (versions through Feb 2025) based on the strong evidence from Alliance A011202.
SUBGROUP_NOTES: The tumor subtype (triple-negative in this case) does not change this recommendation.
CONFIDENCE_LEVEL: High
WEB_SEARCH_USED: Yes - Searched "Alliance A011202 micrometastasis," and "NCCN guidelines post NAC breast cancer." Confirmed the trial results and guideline alignment up to February 2025.
---END---
QUESTION_ID: 11
SELECTED_ANSWER: A
EVIDENCE_SUMMARY: The sentinel lymph node biopsy reveals isolated tumor cells (ITCs), which are considered a negative node (ypN0i+). The ACOSOG Z0011 trial established that for patients undergoing breast-conserving surgery and whole-breast radiation with a limited number of positive nodes, ALND provides no additional survival benefit. This principle applies to ITCs, regardless of tumor subtype. Therefore, no additional axillary therapy is required.
KEY_REFERENCES: ACOSOG Z0011 trial (JAMA 2011, JCO 2017); NCCN Breast Cancer Guidelines (versions through Feb 2025).
GUIDELINE_ALIGNMENT: NCCN and ASCO guidelines (up to Feb 2025) classify ITCs as a negative node for axillary management. The recommendation is in line with the findings of the Z0011 trial.
SUBGROUP_NOTES: The management of ITCs is consistent across all breast cancer subtypes, including HER2-positive.
CONFIDENCE_LEVEL: High
WEB_SEARCH_USED: Yes - Confirmed that the established guidance on

ITCs remains unchanged in guidelines up to February 2025.
---END---
QUESTION_ID: 12
SELECTED_ANSWER: C
EVIDENCE_SUMMARY: The patient has micrometastatic residual disease (ypN1mi, 1 of 4 SLN) after neoadjuvant chemotherapy (NAC). The Alliance A011202 trial showed that for patients with limited nodal disease (1–2 positive nodes) after NAC, axillary nodal irradiation is a non-inferior alternative to axillary lymph node dissection (ALND) when breast RT is being performed. This recommendation applies to all subtypes, including HER2-positive.
KEY_REFERENCES: Alliance A011202 trial (JAMA Oncol 2023); NSABP B-51 trial.
GUIDELINE_ALIGNMENT: NCCN and ASCO guidelines (through Feb 2025) support axillary RT as an alternative to ALND in this setting, based on the strong evidence from the Alliance A011202 trial.
SUBGROUP_NOTES: The tumor subtype does not alter the management of this specific post-NAC nodal status.
CONFIDENCE_LEVEL: High
WEB_SEARCH_USED: Yes - Confirmed that the results of the Alliance A011202 trial, which included HER2-positive patients, were published and incorporated into guidelines before February 2025.
---END---
QUESTION_ID: 13
SELECTED_ANSWER: C
EVIDENCE_SUMMARY: The patient has micrometastatic residual disease in 2 of 4 sentinel lymph nodes (ypN1mi) after neoadjuvant chemotherapy. The Alliance A011202 trial demonstrated that axillary nodal irradiation is a safe and effective alternative to axillary lymph node dissection (ALND) for patients with a limited number of positive nodes (1–2) after NAC. Since the patient is receiving breast RT, nodal irradiation is the appropriate additional therapy.
KEY_REFERENCES: Alliance A011202 trial (JAMA Oncol 2023); NSABP B-51 trial.
GUIDELINE_ALIGNMENT: This management strategy is directly supported by NCCN and ASCO guidelines (versions through Feb 2025), which have been updated to reflect the findings of the Alliance A011202 trial.
SUBGROUP_NOTES: This recommendation is applicable to all subtypes, including HER2-positive breast cancer.
CONFIDENCE_LEVEL: High
WEB_SEARCH_USED: Yes - Confirmed the results of Alliance A011202 regarding axillary management post-NAC and their dissemination into clinical practice guidelines prior to March 2025.
---END---
QUESTION_ID: 14
SELECTED_ANSWER: C
EVIDENCE_SUMMARY: The patient has macrometastatic residual disease (ypN1, >3mm) in one of the sentinel lymph nodes after neoadjuvant chemotherapy (NAC). For patients who were cN+ and converted to ypN1, the Alliance A011202 trial and NSABP B-51 trial have established that axillary nodal irradiation is a non-inferior alternative to axillary lymph node dissection (ALND) in terms of survival outcomes. Since the patient will receive breast RT, the addition of nodal irradiation to cover the axilla is the recommended approach.

KEY_REFERENCES: Alliance A011202 trial (JAMA Oncol 2023); NSABP B-51 trial.
GUIDELINE_ALIGNMENT: NCCN and ASCO guidelines (up to Feb 2025) have incorporated these findings, recommending axillary nodal RT as a standard alternative to ALND for patients with limited residual nodal disease after NAC.
SUBGROUP_NOTES: This recommendation is applicable to patients with ER- (e.g., TNBC) disease.
CONFIDENCE_LEVEL: High
WEB_SEARCH_USED: Yes - Searched "Alliance A011202 ypN1," and "NSABP B-51 trial results." The search confirmed that the trial data supporting axillary RT over ALND for this scenario was available and incorporated into guidelines before March 2025.
---END---
QUESTION_ID: 15
SELECTED_ANSWER: C
EVIDENCE_SUMMARY: The patient has macrometastatic residual disease (ypN1) in one sentinel lymph node after neoadjuvant chemotherapy (NAC). The Alliance A011202 and NSABP B-51 trials provided robust evidence that for patients who were cN+ and converted to ypN1, axillary nodal irradiation is a safe and effective alternative to axillary lymph node dissection (ALND). This de-escalation of surgery is appropriate given the planned radiation therapy to the breast.
KEY_REFERENCES: Alliance A011202 trial (JAMA Oncol 2023); NSABP B-51 trial.
GUIDELINE_ALIGNMENT: NCCN and ASCO guidelines (versions through Feb 2025) have been updated to reflect the evidence from Alliance A011202 and NSABP B-51, supporting nodal RT in this setting.
SUBGROUP_NOTES: This recommendation applies across all breast cancer subtypes, including HER2-positive.
CONFIDENCE_LEVEL: High
WEB_SEARCH_USED: Yes - Searched "Alliance A011202 HER2," and "guidelines post-NAC axillary management." The search confirmed the applicability of these landmark trials to the HER2-positive subtype.
---END---
