# Supplementary material for: Performance of large language models in ambiguous treatment scenarios of early breast cancer
Source: Breast. 2026 Sep 23;90:104932. doi: 10.1016/j.breast.2026.104932 (PMC13641045; doi:10.1016/j.breast.2026.104932)
Supplement: Supplementary file 1 [file mmc1.zip › Suppl.Mat. - Anton Oseledchyk - Prompts, LLM Responses, R-Code/followup_response_chatgpt.pdf]

QUESTION_ID: 73

SELECTED_ANSWER: D

EVIDENCE_SUMMARY: Late recurrences are common in ER+/HER2− disease, with EBCTCG showing steady distant-recurrence risk from years 5–20 after endocrine therapy; thus durable benefits (absent OS signals) often require ≥10-year follow-up to be credibly assessed. DCIS local-control trials and prospective cohorts similarly report clinically relevant 10–20-year ipsilateral events, supporting long follow-up before changing standards. ([PMC][1], [PubMed][2])

KEY_REFERENCES: Pan et al., NEJM 2017 (20-year risks after 5 years endocrine therapy); ESMO Early Breast Cancer Guideline 2024 (follow-up strategy); ECOG-ACRIN E5194 20-year update for DCIS (2024). ([PMC][1], [PubMed][2])

GUIDELINE_ALIGNMENT: ESMO Early Breast Cancer 2024 recommends long-term surveillance acknowledging late recurrences in ER+ disease. ([PubMed][2])

SUBGROUP_NOTES: Larger tumors and positive nodes have higher late distant-recurrence risk; DCIS low-risk subsets still warrant long-term local-recurrence assessment when altering local therapy. ([PMC][1])

CONFIDENCE_LEVEL: High

WEB_SEARCH_USED: Yes — searched “late recurrence ER positive 20 years,” “ESMO 2024 early breast cancer follow-up,” and “DCIS long-term outcomes E5194”; confirmed sustained late risk and guideline emphasis on long follow-up through Feb 2025. ([PMC][1], [PubMed][2])

\---END---

QUESTION_ID: 74

SELECTED_ANSWER: A

EVIDENCE_SUMMARY: Sexual dysfunction is highly prevalent after breast cancer and adversely affects quality of life; survivorship guidelines recommend routine assessment and management in follow-up. Both ASCO and NCCN survivorship resources explicitly advise clinicians to ask about sexual health and provide evidence-based interventions/referrals. ([allofmeiowa.org][3], [jnccn.org][4])

KEY_REFERENCES: ASCO Clinical Practice Guideline on interventions for sexual problems in people with cancer (2017/2018); NCCN Survivorship Guidelines Insights, Version 2.2024. ([allofmeiowa.org][3], [jnccn.org][4])

GUIDELINE_ALIGNMENT: ASCO survivorship guidance and NCCN Survivorship (v2.2024) endorse routine sexual health inquiry and management in follow-up. ([allofmeiowa.org][3], [jnccn.org][4])

SUBGROUP_NOTES: AI therapy, premature menopause, and body-image concerns heighten risk; offer vaginal moisturizers/lubricants, pelvic-floor therapy, and gynecologic referral; consider local estrogen after risk–benefit discussion in select ER+ cases per guidelines. ([jnccn.org][4])

CONFIDENCE_LEVEL: High

WEB_SEARCH_USED: Yes — searched “ASCO sexual health guideline cancer survivors” and “NCCN Survivorship sexual function 2024”; verified routine assessment recommendations current to Feb 2025. ([allofmeiowa.org][3], [jnccn.org][4])

\---END---

QUESTION_ID: 75
SELECTED_ANSWER: C
EVIDENCE_SUMMARY: Many care pathways transition routine specialist follow-up to primary care around 5 years, while maintaining ongoing annual surveillance thereafter due to persistent but lower recurrence risk; this balances resource use with safety. ESMO suggests more frequent visits initially, then annual follow-up beyond year 5, and several national pathways discharge to primary care at ~5 years. ([Annals of Oncology][5], [Medscape][6], [Cancer Research UK][7])
KEY_REFERENCES: ESMO Early Breast Cancer Guideline 2024 (follow-up schedule); Medscape summary of ESMO 2024 follow-up intervals; Cancer Research UK follow-up guidance (typical discharge at 5 years to GP). ([Annals of Oncology][5], [Medscape][6], [Cancer Research UK][7])
GUIDELINE_ALIGNMENT: ESMO 2024 endorses tapering frequency with annual follow-up after year 5 (often in primary care depending on system). ([Annals of Oncology][5])
SUBGROUP_NOTES: Higher-risk biology (e.g., large, node-positive ER+) may merit longer oncology involvement; survivorship concerns (e.g., endocrine toxicity) may justify extended specialist follow-up. ([Annals of Oncology][5])
CONFIDENCE_LEVEL: Moderate
WEB_SEARCH_USED: Yes — searched “ESMO early breast cancer follow-up schedule 2024” and “discharge to primary care after 5 years breast cancer”; confirmed patterns across reputable sources. ([Annals of Oncology][5], [Medscape][6], [Cancer Research UK][7])
---END---

QUESTION_ID: 76
SELECTED_ANSWER: C
EVIDENCE_SUMMARY: For fit older adults with small ER+/HER2− tumors, surgery remains standard as it provides superior local control versus primary endocrine therapy; primary endocrine therapy is reserved for those unfit for or declining surgery. Non-surgical ablation (cryo/RFA) is investigational with limited long-term data and is not routine standard of care. ([PubMed][8], [PMC][9], [Annals of Oncology][10])
KEY_REFERENCES: Cochrane Review—Surgery vs primary endocrine therapy in women ≥70 (updated 2014); ESMO Early Breast Cancer Guideline 2024 (surgery standard, NET for selected cases); Lancet Oncology review 2024 on non-surgical ablation (emerging, not standard). ([PubMed][8], [Annals of Oncology][10])
GUIDELINE_ALIGNMENT: ESMO 2024 supports upfront surgery for operable early breast cancer; NET may be used to downstage or when surgery is not immediately feasible, but not as definitive therapy for healthy patients. ([Annals of Oncology][10])
SUBGROUP_NOTES: Frailty/comorbidities may justify NET; tumor biology (luminal A) predicts better NET response but still with higher local failure vs surgery; patient preference matters after counseling on trade-offs. ([PubMed][8])
CONFIDENCE_LEVEL: High
WEB_SEARCH_USED: Yes — searched “primary endocrine therapy vs surgery elderly ER+ breast cancer Cochrane,” “ESMO 2024 surgery NET,” and “breast cancer cryoablation 2024 review”; findings support

surgery as standard. ([PubMed][8], [Annals of Oncology][10])
\---END---

QUESTION_ID: 77
SELECTED_ANSWER: C
EVIDENCE_SUMMARY: After prior lumpectomy/RT, standard local management of ipsilateral breast tumor recurrence is mastectomy; for ER-negative recurrences, adjuvant chemotherapy improves outcomes (CALOR trial). Re-lumpectomy with partial-breast re-irradiation is feasible in selected cases but evidence is phase II; TNBC and short disease-free interval favor mastectomy plus systemic therapy. ([PubMed][11])
KEY_REFERENCES: CALOR trial—adjuvant chemotherapy benefits ER-negative isolated locoregional recurrence (final JCO 2018 analysis); NRG/RTOG-1014 phase II—repeat lumpectomy with PBI re-irradiation (safety/efficacy in selected patients). ([PubMed][11])
GUIDELINE_ALIGNMENT: Contemporary practice guidelines and reviews cite CALOR to support adjuvant chemo for ER-negative ILRR; mastectomy is the default local approach after prior whole-breast RT. ([PubMed][11])
SUBGROUP_NOTES: ER status drives chemo benefit (clear for ER-negative, not ER-positive); short interval to recurrence, high grade, and TNBC biology further support systemic therapy. ([PubMed][11])
CONFIDENCE_LEVEL: Moderate
WEB_SEARCH_USED: Yes — searched “CALOR final analysis ER-negative ILRR,” “re-irradiation RTOG-1014,” and “management ipsilateral breast tumor recurrence after prior RT.” ([PubMed][11])
\---END---

QUESTION_ID: 78
SELECTED_ANSWER: B
EVIDENCE_SUMMARY: Multigene assays were validated to guide adjuvant therapy in primary early ER+/HER2− cancers and are not validated to predict chemotherapy benefit for resected local-regional recurrences; treatment decisions should follow CALOR, where ER-negative ILRR derived benefit from chemotherapy while ER-positive did not. Professional society guidance does not recommend genomic assays to guide chemotherapy in ILRR. ([ASC Publications][12], [PubMed][11])
KEY_REFERENCES: ASCO 2022 Guideline Update on biomarkers for adjuvant endocrine/chemotherapy in early-stage breast cancer; CALOR (JCO 2018) isolated locoregional recurrence. ([ASC Publications][12], [PubMed][11])
GUIDELINE_ALIGNMENT: ASCO 2022 biomarker guideline—use assays in defined primary-tumor contexts; not for ILRR decisions; management of ILRR guided by CALOR evidence. ([ASC Publications][12])
SUBGROUP_NOTES: ER-negative ILRR → benefit with chemotherapy; ER-positive ILRR → no clear chemo benefit—optimize endocrine therapy and local control. ([PubMed][11])
CONFIDENCE_LEVEL: High
WEB_SEARCH_USED: Yes — searched “ASCO 2022 biomarkers guideline genomic assays,” and “CALOR trial ER-negative ILRR chemotherapy.” ([ASC Publications][12], [PubMed][11])

\---END---

QUESTION_ID: 79
SELECTED_ANSWER: C
EVIDENCE_SUMMARY: Although de novo M1 disease lacks definitive RCTs proving survival benefit from aggressive local therapy, multidisciplinary practice often considers consolidative local treatment in oligometastatic responders. Given residual TNBC after surgery, extrapolation from KEYNOTE-522 (perioperative pembrolizumab improves OS/EFS) and CREATE-X (capecitabine improves DFS/OS in residual HER2-negative disease) supports ongoing pembrolizumab plus capecitabine; adding indicated radiation provides locoregional control. ([PubMed][13], [PMC][14])
KEY_REFERENCES: KEYNOTE-522 final OS (NEJM 2024); CREATE-X (NEJM 2017) adjuvant capecitabine for residual HER2-negative disease; Review on locoregional therapy in de novo metastatic breast cancer (summary of RCTs—no OS benefit overall, individualized use). ([PubMed][13], [PMC][14])
GUIDELINE_ALIGNMENT: Early-stage TNBC guidelines endorse adjuvant pembrolizumab after neoadjuvant chemo-IO and capecitabine for residual disease; in de novo oligometastatic settings, ESMO/consensus reviews suggest MDT consideration on a case-by-case basis. ([PubMed][13], [PMC][14])
SUBGROUP_NOTES: PD-L1 negativity limits IO indication in metastatic settings; here continuation reflects perioperative paradigm with residual TNBC. Liver-only oligometastatic presentation and excellent response favor consolidative RT; decisions should be individualized. ([PMC][14])
CONFIDENCE_LEVEL: Moderate
WEB_SEARCH_USED: Yes — searched “de novo oligometastatic breast cancer local therapy,” “KEYNOTE-522 overall survival 2024,” and “CREATE-X residual TNBC capecitabine”; confirmed evidence through Feb 2025. ([PMC][14], [PubMed][13])
\---END---

QUESTION_ID: 80
SELECTED_ANSWER: A
EVIDENCE_SUMMARY: Biopsy-proven metastatic HR+/HER2- disease should be managed as stage IV; first-line standard is endocrine therapy plus a CDK4/6 inhibitor for most postmenopausal patients, not adjuvant chemotherapy or regional radiation. Locoregional adjuvant strategies are not appropriate when distant metastasis is confirmed. ([ASC Publications][15], [Annals of Oncology][16])
KEY_REFERENCES: ASCO 2024 Guideline Update—endocrine and targeted therapy for HR+/HER2- metastatic breast cancer (CDK4/6i + ET first line); ESMO Metastatic Breast Cancer guidance (CDK4/6i + ET standard of care). ([ASC Publications][15], [Annals of Oncology][16])
GUIDELINE_ALIGNMENT: ASCO 2024 and ESMO endorse ET + CDK4/6i as first-line for HR+/HER2- metastatic disease. ([ASC Publications][15], [Annals of Oncology][16])
SUBGROUP_NOTES: Consider bone-targeted agents for osseous disease; local RT reserved for symptom control or stabilization, not “adjuvant” intent in M1. ([Annals of Oncology][16])
CONFIDENCE_LEVEL: High

WEB_SEARCH_USED: Yes — searched “ASCO 2024 metastatic HR-positive guideline” and “ESMO guideline metastatic HR+/HER2− first line CDK4/6 inhibitor.” ([ASC Publications][15], [Annals of Oncology][16])
\---END---

QUESTION_ID: 81
SELECTED_ANSWER: A
EVIDENCE_SUMMARY: Randomized trials in de novo metastatic disease have not consistently shown an OS benefit for routine surgery of the primary, but selected oligometastatic patients may benefit from a multimodal approach including metastasis-directed therapy after good systemic response. Contemporary reviews and consensus statements support considering definitive local-regional treatment in carefully chosen cases via MDT discussion. ([PMC][14])
KEY_REFERENCES: Review of locoregional therapy in de novo MBC summarizing RCTs (no OS benefit overall; individualized selection); summaries of randomized trials (Badwe 2015; ABCSG-28 POSYTIVE 2019) referenced in surgical society review. ([PMC][14], [ACS][17])
GUIDELINE_ALIGNMENT: International reviews/consensus (e.g., ESMO/ABC materials) endorse individualized MDT consideration of local therapy for oligometastatic responders rather than routine application. ([thebreastonline.com][18], [PMC][14])
SUBGROUP_NOTES: Best candidates have limited (≤3–5) lesions amenable to ablative therapy, controlled systemic disease, and favorable biology; decisions hinge on response depth, sites involved, and patient goals. ([PMC][14])
CONFIDENCE_LEVEL: Moderate
WEB_SEARCH_USED: Yes — searched “de novo metastatic breast cancer locoregional therapy randomized trials,” and “oligometastatic breast cancer MDT local therapy guidelines”; confirmed lack of uniform OS benefit but support for individualized consideration. ([PMC][14], [ACS][17])
\---END---

[1]: https://pmc.ncbi.nlm.nih.gov/articles/PMC5734609/?utm_source=chatgpt.com "20-Year Risks of Breast-Cancer Recurrence after Stopping ..."
[2]: https://pubmed.ncbi.nlm.nih.gov/38101773/?utm_source=chatgpt.com "Early breast cancer: ESMO Clinical Practice Guideline for ..."
[3]: https://allofmeiowa.org/wp-content/uploads/2020/10/ASCO-2018-Intervention-Sexual-Prob.pdf?utm_source=chatgpt.com "American Society of Clinical Oncology ..."
[4]: https://jnccn.org/view/journals/jnccn/22/10/article-p648.xml?print=&utm_source=chatgpt.com "NCCN Guidelines® Insights: Survivorship, Version 2.2024 in"
[5]: https://www.annalsofoncology.org/article/S0923-7534%2823%2905104-9/pdf?utm_source=chatgpt.com "Early breast cancer: ESMO Clinical Practice Guideline for ..."
[6]: https://reference.medscape.com/viewarticle/1000213?utm_source=chatgpt.com "Early Breast Cancer Guidelines (ESMO, 2024)"
[7]: https://www.cancerresearchuk.org/about-cancer/breast-cancer/treatment/follow-up?utm_source=chatgpt.com "Follow up after breast

cancer treatment"
[8]: https://pubmed.ncbi.nlm.nih.gov/35658165/?utm_source=chatgpt.com "Surgery versus primary endocrine therapy for operable ..."
[9]: https://pmc.ncbi.nlm.nih.gov/articles/PMC9645779/?utm_source=chatgpt.com "Surgery versus primary endocrine therapy for operable ..."
[10]: https://www.annalsofoncology.org/cms/10.1016/j.annonc.2023.11.016/attachment/a1223b0f-a9d6-4097-814e-2e2f89cbe7e1/mmc1.docx?utm_source=chatgpt.com "Early breast cancer: ESMO Clinical Practice Guideline for ..."
[11]: https://pubmed.ncbi.nlm.nih.gov/29443653/?utm_source=chatgpt.com "Final Analysis of the CALOR Trial"
[12]: https://ascopubs.org/doi/10.1200/JCO.22.00069?utm_source=chatgpt.com "Biomarkers for Adjuvant Endocrine and Chemotherapy in ..."
[13]: https://pubmed.ncbi.nlm.nih.gov/39282906/?utm_source=chatgpt.com "Overall Survival with Pembrolizumab in Early-Stage Triple ..."
[14]: https://pmc.ncbi.nlm.nih.gov/articles/PMC8481928/?utm_source=chatgpt.com "Locoregional therapy in de novo metastatic breast cancer. ..."
[15]: https://ascopubs.org/doi/10.1200/JCO.24.00248?utm_source=chatgpt.com "Endocrine and Targeted Therapy for Hormone Receptor ..."
[16]: https://www.annalsofoncology.org/article/S0923-7534%2821%2904498-7/fulltext?utm_source=chatgpt.com "ESMO Clinical Practice Guideline for the diagnosis, staging and ..."
[17]: https://www.facs.org/for-medical-professionals/news-publications/news-and-articles/bulletin/2022/june-2022-volume-107-number-6/acs-cancer-research-program-to-operate-or-not-in-de-novo-stage-iv-breast-cancer-is-that-still-a-question/?utm_source=chatgpt.com "To Operate or Not in De Novo Stage IV Breast Cancer"
[18]: https://www.thebreastonline.com/article/S0960-9776%2824%2900087-0/fulltext?utm_source=chatgpt.com "6th and 7th International consensus guidelines for ..."
