# Supplementary material for: Performance of large language models in ambiguous treatment scenarios of early breast cancer
Source: Breast. 2026 Sep 23;90:104932. doi: 10.1016/j.breast.2026.104932 (PMC13641045; doi:10.1016/j.breast.2026.104932)
Supplement: Supplementary file 1 [file mmc1.zip › Suppl.Mat. - Anton Oseledchyk - Prompts, LLM Responses, R-Code/followup_response_claude.pdf]

QUESTION_ID: 73
SELECTED_ANSWER: D
EVIDENCE_SUMMARY: ER-positive breast cancer has a prolonged risk of recurrence extending beyond 20 years, with approximately 1% annual risk persisting indefinitely according to EBCTCG data. MonarchE trial used 5-year follow-up data, but experts recommend at least 10 years follow-up before recommending new approaches without overall survival benefit, given the late recurrence pattern of ER+ disease.
KEY_REFERENCES: EBCTCG 20-year recurrence data (NEJM 2017), monarchE 5-year update (JCO 2024), ATAC/BIG 1-98 long-term follow-up
GUIDELINE_ALIGNMENT: FDA guidance recommends minimum 10-year follow-up for ER+ disease given late recurrence risk
SUBGROUP_NOTES: Higher risk patients may have earlier events, but substantial recurrences occur beyond 10 years
CONFIDENCE_LEVEL: High
WEB_SEARCH_USED: Yes
---END---

QUESTION_ID: 74
SELECTED_ANSWER: A
EVIDENCE_SUMMARY: Sexual health is a critical but often overlooked aspect of breast cancer survivorship, with studies showing it significantly impacts quality of life. NCCN and ASCO survivorship guidelines recommend routine assessment of sexual health, as many survivors experience dysfunction but rarely initiate discussions themselves.
KEY_REFERENCES: NCCN Survivorship Guidelines 2024, ASCO Breast Cancer Survivorship Guidelines, multiple QOL studies 2024
GUIDELINE_ALIGNMENT: NCCN Survivorship Guidelines specifically include sexual health assessment; ASCO recommends routine screening
SUBGROUP_NOTES: Particularly important in younger survivors and those on endocrine therapy
CONFIDENCE_LEVEL: High
WEB_SEARCH_USED: Yes
---END---

QUESTION_ID: 75
SELECTED_ANSWER: C
EVIDENCE_SUMMARY: Most guidelines recommend cancer specialist follow-up for 5 years, after which care can transition to primary care providers with appropriate survivorship planning. This balances the higher recurrence risk in the first 5 years with efficient resource utilization and patient convenience.
KEY_REFERENCES: ASCO/ACS Survivorship Guidelines, NCCN Guidelines 2024, survivorship care models
GUIDELINE_ALIGNMENT: ASCO/ACS recommend 5 years specialist follow-up; NCCN supports transition at 5 years with survivorship plan
SUBGROUP_NOTES: High-risk patients or those on active treatment may require longer specialist follow-up
CONFIDENCE_LEVEL: High
WEB_SEARCH_USED: Yes
---END---

QUESTION_ID: 76

SELECTED_ANSWER: B
EVIDENCE_SUMMARY: For elderly patients with small ER+ tumors seeking to avoid surgery, stereotactic body radiation therapy (SBRT/SABR) offers a non-invasive alternative with good local control. While surgery remains standard, SBRT is increasingly recognized as reasonable for patients unable or unwilling to undergo surgery, particularly in the elderly.
KEY_REFERENCES: ABLATIVE trial design, multiple SBRT studies in elderly, NCCN considerations for elderly
GUIDELINE_ALIGNMENT: Guidelines acknowledge SBRT as alternative when surgery contraindicated or refused
SUBGROUP_NOTES: Best suited for small (<2cm), ER+ tumors in elderly patients
CONFIDENCE_LEVEL: Moderate
WEB_SEARCH_USED: Yes
---END---

QUESTION_ID: 77
SELECTED_ANSWER: A
EVIDENCE_SUMMARY: Local recurrence after breast conservation and radiation requires mastectomy as salvage surgery standard of care. The similar histology and timing (3 years) suggest true recurrence rather than new primary, and additional chemotherapy is not indicated for a small local recurrence that can be surgically resected.
KEY_REFERENCES: NCCN Guidelines for local recurrence, salvage mastectomy outcomes data, CALOR trial
GUIDELINE_ALIGNMENT: NCCN and ASCO recommend mastectomy for ipsilateral breast tumor recurrence after BCT
SUBGROUP_NOTES: Re-irradiation remains investigational; systemic therapy reserved for unresectable disease
CONFIDENCE_LEVEL: High
WEB_SEARCH_USED: Yes
---END---

QUESTION_ID: 78
SELECTED_ANSWER: B
EVIDENCE_SUMMARY: Genomic risk signatures like Oncotype DX were validated in the adjuvant setting for primary breast cancer, not for recurrent disease. Patients with local recurrence on endocrine therapy have demonstrated endocrine resistance, making genomic scores less informative for chemotherapy benefit decisions in this context.
KEY_REFERENCES: TAILORx and RxPONDER inclusion criteria, expert consensus on recurrent disease, limited validation data
GUIDELINE_ALIGNMENT: No guidelines support genomic testing for recurrent disease; tests not validated in this setting
SUBGROUP_NOTES: Recurrence on ET indicates different biology than primary tumor testing would assess
CONFIDENCE_LEVEL: High
WEB_SEARCH_USED: Yes
---END---

QUESTION_ID: 79

SELECTED_ANSWER: B
EVIDENCE_SUMMARY: De novo stage IV TNBC with excellent response to systemic therapy and conversion to oligometastatic state may benefit from local consolidation. Continuing pembrolizumab with radiation to the primary and regional nodes follows the paradigm of treating all sites of disease in exceptional responders.
KEY_REFERENCES: SABR-COMET principles, oligometastatic breast cancer data, exceptional responder protocols
GUIDELINE_ALIGNMENT: Emerging consensus supports local therapy for exceptional responders with oligometastatic conversion
SUBGROUP_NOTES: Complete metabolic response at metastatic site with small residual primary supports aggressive local therapy
CONFIDENCE_LEVEL: Moderate
WEB_SEARCH_USED: Yes
---END---

QUESTION_ID: 80
SELECTED_ANSWER: A
EVIDENCE_SUMMARY: Confirmed metastatic disease (biopsy-proven spine metastasis) changes staging to de novo stage IV, requiring systemic therapy for metastatic disease. Standard first-line treatment is AI plus CDK4/6 inhibitor, without local therapy to the breast given the metastatic setting.
KEY_REFERENCES: PALOMA-2, MONALEESA trials, de novo stage IV management guidelines
GUIDELINE_ALIGNMENT: NCCN and ASCO recommend systemic therapy alone for de novo metastatic ER+ breast cancer
SUBGROUP_NOTES: Local therapy to primary considered only in exceptional responders or for palliation
CONFIDENCE_LEVEL: High
WEB_SEARCH_USED: Yes
---END---

QUESTION_ID: 81
SELECTED_ANSWER: A
EVIDENCE_SUMMARY: Emerging evidence from SABR-COMET and other trials supports local therapy for oligometastatic disease with good initial response, showing improved progression-free and potentially overall survival. The oligometastatic paradigm suggests curative potential when all disease sites can be ablated.
KEY_REFERENCES: SABR-COMET long-term results, NRG-BR002, multiple oligometastatic breast cancer studies
GUIDELINE_ALIGNMENT: Increasingly accepted though primarily within clinical trials; ESTRO-ASTRO consensus supports consideration
SUBGROUP_NOTES: Best candidates have limited sites (1-5), controlled primary, long disease-free interval
CONFIDENCE_LEVEL: Moderate
WEB_SEARCH_USED: Yes
---END---
