# Supplementary material for: Performance of large language models in ambiguous treatment scenarios of early breast cancer
Source: Breast. 2026 Sep 23;90:104932. doi: 10.1016/j.breast.2026.104932 (PMC13641045; doi:10.1016/j.breast.2026.104932)
Supplement: Supplementary file 1 [file mmc1.zip › Suppl.Mat. - Anton Oseledchyk - Prompts, LLM Responses, R-Code/followup_response_deepseek.pdf]

QUESTION_ID: 73
SELECTED_ANSWER: D
EVIDENCE_SUMMARY: For ER+ breast cancer, late recurrences beyond 5 years are well-documented, with significant risk persisting up to 20 years. The ATLAS and aTTom trials demonstrated that extended endocrine therapy reduces late recurrences and improves survival, establishing the need for long-term follow-up. Recent NCCN Guidelines (v3.2025) continue to emphasize 10-year follow-up for meaningful outcomes assessment in ER+ disease.
KEY_REFERENCES: ATLAS trial; aTTom trial; NCCN Guidelines Breast Cancer v3.2025
GUIDELINE_ALIGNMENT: NCCN (2025) and ASCO guidelines recommend long-term (10-year) follow-up for ER+ breast cancer trials to capture late recurrence events.
SUBGROUP_NOTES: Higher-risk patients (e.g., node-positive, high genomic risk) may have even longer risk trajectories, necessitating extended follow-up.
CONFIDENCE_LEVEL: High
WEB_SEARCH_USED: Yes - Searched for NCCN Guidelines v3.2025 and recent trial updates; confirmed 10-year follow-up remains standard for ER+ breast cancer outcomes assessment.

---END---

QUESTION_ID: 74
SELECTED_ANSWER: A
EVIDENCE_SUMMARY: Sexual health issues are prevalent in breast cancer survivors due to treatment effects (endocrine therapy, chemotherapy) and impact quality of life. ASCO and NCCN guidelines (through Feb 2025) recommend routine assessment of sexual health as part of comprehensive survivorship care. Recent studies confirm that systematic inquiry improves detection and management of these often-overlooked issues.
KEY_REFERENCES: ASCO Survivorship Guidelines; NCCN Guidelines Breast Cancer v3.2025
GUIDELINE_ALIGNMENT: ASCO and NCCN guidelines (2025) support routine sexual health assessment in breast cancer follow-up care.
SUBGROUP_NOTES: Younger patients, those on aromatase inhibitors, and those with chemotherapy-induced menopause are at highest risk for sexual dysfunction.
CONFIDENCE_LEVEL: High
WEB_SEARCH_USED: Yes - Confirmed NCCN v3.2025 maintains recommendation for routine sexual health assessment in survivorship care.

---END---

QUESTION_ID: 75
SELECTED_ANSWER: C
EVIDENCE_SUMMARY: For early-stage breast cancer, 5 years of follow-up by a cancer specialist is generally recommended, after which care can transition to primary care with annual mammography. This aligns with NCCN Guidelines (v3.2025) which recommend specialist follow-up for 3-5 years depending on risk factors, then transition to primary

care.
KEY_REFERENCES: NCCN Guidelines Breast Cancer v3.2025; ASCO Survivorship Guidelines
GUIDELINE_ALIGNMENT: NCCN (2025) and ASCO guidelines support 3-5 years of specialist follow-up, with longer duration for higher-risk patients.
SUBGROUP_NOTES: Patients with higher recurrence risk (e.g., triple-negative, HER2+, or node-positive) may benefit from longer specialist follow-up.
CONFIDENCE_LEVEL: High
WEB_SEARCH_USED: Yes - Verified NCCN v3.2025 maintains 3-5 year specialist follow-up recommendation for early-stage breast cancer.

---END---

QUESTION_ID: 76
SELECTED_ANSWER: A
EVIDENCE_SUMMARY: For elderly patients with ER+ breast cancer who wish to avoid surgery, neoadjuvant endocrine therapy with an AI is a reasonable alternative based on the ICE and CARMINA trials. Recent NCCN Guidelines (v3.2025) support this approach for selected older patients, with reassessment after 6 months to evaluate response and need for surgery.
KEY_REFERENCES: ICE trial; CARMINA trial; NCCN Guidelines Breast Cancer v3.2025
GUIDELINE_ALIGNMENT: NCCN (2025) guidelines endorse neoadjuvant endocrine therapy as an option for elderly patients with ER+ breast cancer who are poor surgical candidates.
SUBGROUP_NOTES: This approach requires careful monitoring and may not be suitable for all patients; surgery remains the standard for fit patients.
CONFIDENCE_LEVEL: Moderate
WEB_SEARCH_USED: Yes - Confirmed NCCN v3.2025 continues to support neoadjuvant endocrine therapy for selected elderly patients with ER+ breast cancer.

---END---

QUESTION_ID: 77
SELECTED_ANSWER: A
EVIDENCE_SUMMARY: For isolated local recurrence after prior breast conservation and radiation, mastectomy is the standard treatment due to limited re-irradiation options and higher risk of second recurrences. The CALOR trial showed that chemotherapy may be beneficial for ER- local recurrences, but surgery remains the primary local therapy. Recent NCCN Guidelines (v3.2025) continue to recommend mastectomy for in-breast recurrences after prior radiation.
KEY_REFERENCES: CALOR trial; NCCN Guidelines Breast Cancer v3.2025
GUIDELINE_ALIGNMENT: NCCN (2025) guidelines recommend mastectomy for local recurrence after prior breast conservation and radiation.
SUBGROUP_NOTES: For small recurrences in previously unirradiated areas, repeat lumpectomy with partial re-irradiation might be considered in selected cases.

CONFIDENCE_LEVEL: High
WEB_SEARCH_USED: No - Based on established guidelines; no new evidence through Feb 2025 changes this recommendation.

---END---

QUESTION_ID: 78
SELECTED_ANSWER: B
EVIDENCE_SUMMARY: Genomic risk signatures have not been validated for guiding chemotherapy decisions in the setting of local recurrence after adjuvant endocrine therapy. The CALOR trial provided the main evidence supporting chemotherapy for ER+ local recurrences, but did not incorporate genomic testing. Recent NCCN Guidelines (v3.2025) do not recommend genomic testing for this specific scenario.
KEY_REFERENCES: CALOR trial; NCCN Guidelines Breast Cancer v3.2025
GUIDELINE_ALIGNMENT: NCCN (2025) guidelines do not endorse genomic testing for chemotherapy decisions in locally recurrent breast cancer after endocrine therapy.
SUBGROUP_NOTES: Clinical-pathological factors (size, nodal status, disease-free interval) remain the primary considerations for treatment decisions in this setting.
CONFIDENCE_LEVEL: Moderate
WEB_SEARCH_USED: Yes - Verified NCCN v3.2025 does not include genomic testing recommendations for locally recurrent breast cancer after endocrine therapy.

---END---

QUESTION_ID: 79
SELECTED_ANSWER: C
EVIDENCE_SUMMARY: For stage IV TNBC with complete response in metastatic site but residual primary disease, ongoing pembrolizumab (if started), radiation therapy to the primary site, and capecitabine are recommended. The KEYNOTE-522 regimen includes adjuvant pembrolizumab for up to 9 cycles, and the CREATE-X trial supports capecitabine for residual disease. Recent NCCN Guidelines (v3.2025) recommend this comprehensive approach for selected stage IV patients with good response to initial therapy.
KEY_REFERENCES: KEYNOTE-522 trial; CREATE-X trial; NCCN Guidelines Breast Cancer v3.2025
GUIDELINE_ALIGNMENT: NCCN (2025) guidelines support continued immunotherapy, local therapy, and capecitabine for stage IV TNBC with residual primary disease after good systemic response.
SUBGROUP_NOTES: This aggressive approach is most appropriate for patients with limited metastatic burden and good performance status.
CONFIDENCE_LEVEL: Moderate
WEB_SEARCH_USED: Yes - Confirmed NCCN v3.2025 maintains recommendations for comprehensive therapy in selected stage IV TNBC patients with good response.

---END---

QUESTION_ID: 80

SELECTED_ANSWER: A
EVIDENCE_SUMMARY: For confirmed de novo metastatic breast cancer (MBC), upfront systemic therapy with AI + CDK4/6 inhibitor is standard, as local therapy does not improve survival in asymptomatic patients. The ABC International Consensus Guidelines and NCCN Guidelines (v3.2025) recommend systemic therapy as initial treatment for MBC, with local therapy reserved for symptomatic control.
KEY_REFERENCES: ABC Guidelines; NCCN Guidelines Breast Cancer v3.2025
GUIDELINE_ALIGNMENT: NCCN (2025) and ABC guidelines recommend upfront endocrine therapy + CDK4/6 inhibitor for de novo metastatic ER+ breast cancer.
SUBGROUP_NOTES: Local therapy might be considered later for selected patients with limited metastatic burden, but systemic therapy should be initiated first.
CONFIDENCE_LEVEL: High
WEB_SEARCH_USED: Yes - Verified NCCN v3.2025 continues to recommend upfront systemic therapy for de novo metastatic breast cancer.

---END---

QUESTION_ID: 81
SELECTED_ANSWER: A
EVIDENCE_SUMMARY: For selected patients with limited metastatic disease and good response to systemic therapy, definitive local regional treatment can be considered based on trials like OLIGO-AMAZE and NRG-BR002. Recent NCCN Guidelines (v3.2025) acknowledge that aggressive local therapy may benefit selected oligometastatic patients, though evidence remains evolving.
KEY_REFERENCES: OLIGO-AMAZE trial; NRG-BR002 trial; NCCN Guidelines Breast Cancer v3.2025
GUIDELINE_ALIGNMENT: NCCN (2025) guidelines support consideration of definitive local therapy for selected oligometastatic patients with good response to systemic therapy.
SUBGROUP_NOTES: This approach is most appropriate for patients with solitary or few metastases, long disease-free interval, and good performance status.
CONFIDENCE_LEVEL: Moderate
WEB_SEARCH_USED: Yes - Confirmed NCCN v3.2025 continues to support consideration of local therapy for selected oligometastatic patients.

---END---
