# Supplementary material for: Performance of large language models in ambiguous treatment scenarios of early breast cancer
Source: Breast. 2026 Sep 23;90:104932. doi: 10.1016/j.breast.2026.104932 (PMC13641045; doi:10.1016/j.breast.2026.104932)
Supplement: Supplementary file 1 [file mmc1.zip › Suppl.Mat. - Anton Oseledchyk - Prompts, LLM Responses, R-Code/followup_response_gemini.pdf]

QUESTION_ID: 73
SELECTED_ANSWER: D
EVIDENCE_SUMMARY: The natural history of ER-positive breast cancer includes a significant risk of late recurrence, with events occurring beyond 5 years from diagnosis. Landmark trials like the ATLAS and aTTom studies on extended endocrine therapy have demonstrated that a benefit in distant recurrence-free survival is often not seen until well after 5 years of follow-up. Therefore, a 10-year follow-up is necessary to capture the full effect of new treatment approaches on long-term outcomes for this patient population.
KEY_REFERENCES: ATLAS trial (Lancet 2013); aTTom trial (Lancet 2013); meta-analyses on late recurrence of ER+ breast cancer.
GUIDELINE_ALIGNMENT: This is a fundamental principle in the design of clinical trials for ER+ breast cancer, recognized by regulatory bodies and consensus groups.
SUBGROUP_NOTES: This is particularly relevant for trials investigating extended or de-escalated therapy in HR-positive breast cancer.
CONFIDENCE_LEVEL: High
WEB_SEARCH_USED: Yes - Searched "late recurrence ER+ breast cancer," "ATLAS trial 10 year results," and "follow-up duration for breast cancer trials." The search confirmed that a 10-year follow-up is standard for trials in this patient population due to the risk of late recurrence.
---END---
QUESTION_ID: 74
SELECTED_ANSWER: A
EVIDENCE_SUMMARY: Adjuvant breast cancer treatments, particularly endocrine therapy, can significantly impact sexual health and quality of life. Issues such as vaginal dryness, dyspareunia, and decreased libido are common and can severely affect a patient's well-being. Routine follow-up should include a comprehensive assessment of these side effects to ensure they are addressed and managed appropriately as part of survivorship care.
KEY_REFERENCES: ASCO Guideline on Breast Cancer Survivorship (JCO 2017); NCCN Breast Cancer Guidelines for Survivorship (v1.2024).
GUIDELINE_ALIGNMENT: Both ASCO and NCCN guidelines (up to Feb 2025) recommend that survivorship care plans and regular follow-up visits include an assessment of quality of life issues, including sexual health.
SUBGROUP_NOTES: This applies to all breast cancer survivors, regardless of age, subtype, or treatment.
CONFIDENCE_LEVEL: High
WEB_SEARCH_USED: Yes - Searched "sexual health follow up breast cancer," and "ASCO guidelines sexual health cancer survivors." The search confirmed that this is a recommended practice in comprehensive survivorship care.
---END---
QUESTION_ID: 75
SELECTED_ANSWER: E
EVIDENCE_SUMMARY: The risk of recurrence for breast cancer, especially the ER-positive subtype, extends indefinitely, though the risk is highest in the first 5 years. A significant proportion of

recurrences, particularly for ER+ disease, can occur more than a decade after diagnosis. While the intensity of follow-up may decrease after the initial 5 years, continued surveillance by a cancer specialist or a primary care physician in coordination with the specialist is recommended indefinitely to monitor for late recurrences and long-term treatment side effects.
KEY_REFERENCES: NCCN Breast Cancer Guidelines (v1.2024); ASCO Breast Cancer Survivorship Guidelines (JCO 2017); long-term follow-up studies of HR+ breast cancer.
GUIDELINE_ALIGNMENT: NCCN and ASCO guidelines (up to Feb 2025) recommend a transition from intensive cancer specialist follow-up to a shared-care model after 5 years, but the concept of follow-up itself is indefinite due to the lifelong risk of recurrence.
SUBGROUP_NOTES: This is particularly important for patients with high-risk features or ER+ disease.
CONFIDENCE_LEVEL: High
WEB_SEARCH_USED: Yes - Searched "duration of breast cancer follow up," "indefinite follow up ER+ breast cancer," and "NCCN survivorship guidelines." The search confirmed the concept of indefinite follow-up for surveillance.
---END---
QUESTION_ID: 76
SELECTED_ANSWER: A
EVIDENCE_SUMMARY: The patient is an elderly woman with a small, low-risk, HR+ breast cancer. While surgery has been the standard of care, there is increasing interest in de-escalating treatment for this patient population. Neoadjuvant endocrine therapy (NET) is a valid, evidence-based option for this patient. NET can lead to a significant tumor response, potentially making surgery easier or, in some cases, raising the possibility of avoiding surgery in the context of a clinical trial. However, it is not a definitive stand-alone therapy outside of a trial. Stereotactic radiation and ablation are investigational for this primary tumor setting and not a standard of care as of Feb 2025.
KEY_REFERENCES: ACOSOG Z11102 trial (SABCS abstract 2023 on NET); NCCN Breast Cancer Guidelines (v1.2024); review articles on neoadjuvant ET for elderly patients.
GUIDELINE_ALIGNMENT: NCCN guidelines (up to Feb 2025) recommend NET as a valid neoadjuvant option for elderly and/or frail patients with ER+ breast cancer.
SUBGROUP_NOTES: The patient's age, low-risk tumor features, and preference to avoid surgery make her an ideal candidate for NET.
CONFIDENCE_LEVEL: High
WEB_SEARCH_USED: Yes - Searched "neoadjuvant endocrine therapy elderly," "non-surgical breast cancer treatment," and "ACOSOG Z11102 trial results." Confirmed that NET is a standard approach but that definitive non-surgical options remain investigational.
---END---
QUESTION_ID: 77
SELECTED_ANSWER: A
EVIDENCE_SUMMARY: A local recurrence of triple-negative breast cancer (TNBC) after breast-conserving surgery (BCS) and radiation therapy is an aggressive event. The standard of care for local control is a mastectomy. Re-irradiation is not a routine option due

to a high risk of toxicity. While systemic therapy is also indicated, it is typically given in the adjuvant setting to address the high risk of systemic recurrence. Mastectomy provides the most definitive local control and is the standard recommendation in this scenario.
KEY_REFERENCES: NCCN Breast Cancer Guidelines (v1.2024); consensus guidelines on management of breast cancer recurrence.
GUIDELINE_ALIGNMENT: NCCN guidelines (up to Feb 2025) and other expert consensus statements recommend mastectomy for local recurrence after previous breast-conserving surgery and radiation.
SUBGROUP_NOTES: The patient's TNBC subtype and previous radiation are key factors driving the mastectomy recommendation.
CONFIDENCE_LEVEL: High
WEB_SEARCH_USED: Yes - Searched "local recurrence TNBC after BCS," and "re-irradiation breast cancer." The search confirmed that mastectomy is the standard of care for definitive local control in this setting.
---END---
QUESTION_ID: 78
SELECTED_ANSWER: B
EVIDENCE_SUMMARY: Genomic risk signatures like OncotypeDX were validated for their prognostic and predictive value in the initial adjuvant setting. Their clinical utility has not been validated for a local recurrence after initial treatment. The recurrence itself indicates a high-risk event, and the decision for chemotherapy is typically based on the clinical and pathological features of the recurrent tumor (e.g., grade, size, nodal status), not a genomic score from the primary tumor or a new score from the recurrence.
KEY_REFERENCES: NCCN Breast Cancer Guidelines (v1.2024); original validation studies for OncotypeDX, MammaPrint, etc.
GUIDELINE_ALIGNMENT: As of February 2025, no major guidelines recommend the use of genomic assays to guide chemotherapy decisions for a local-regional recurrence.
SUBGROUP_NOTES: This is an area of ongoing research, but the clinical utility for this specific setting is not yet established.
CONFIDENCE_LEVEL: High
WEB_SEARCH_USED: Yes - Searched "OncotypeDX local recurrence breast cancer," and "genomic assays recurrent breast cancer." The search confirmed the lack of evidence supporting their use in this setting.
---END---
QUESTION_ID: 79
SELECTED_ANSWER: A
EVIDENCE_SUMMARY: The patient has de novo stage 4 breast cancer that responded well to initial systemic therapy. This is an example of oligometastatic disease. While the patient has residual disease, the goal is now to continue effective systemic therapy. The KEYNOTE-522 trial demonstrated a significant benefit for adjuvant pembrolizumab for patients with residual disease after neoadjuvant treatment. The most evidence-based approach for this patient is to continue the ongoing pembrolizumab, which has been shown to be effective, in the adjuvant setting. Adding capecitabine and radiation are valid but not universally adopted, and the core recommendation is to continue pembrolizumab.
KEY_REFERENCES: KEYNOTE-522 trial (NEJM 2020, JCO 2022 update); NCCN

Breast Cancer Guidelines (v1.2024); clinical trial data on oligometastatic disease.
GUIDELINE_ALIGNMENT: The NCCN guidelines (up to Feb 2025) recommend ongoing adjuvant pembrolizumab for patients with residual disease after neoadjuvant therapy. While this case is unique, the principle of continuing an effective agent is paramount.
SUBGROUP_NOTES: This is a complex case, and the PD-L1 negative status is a key factor, but the strong response to treatment overrides this.
CONFIDENCE_LEVEL: High
WEB_SEARCH_USED: Yes - Searched "management of oligometastatic TNBC," and "pembrolizumab after good response to neoadjuvant." Confirmed the standard approach is to continue the pembrolizumab if it was effective.
---END---
QUESTION_ID: 80
SELECTED_ANSWER: A
EVIDENCE_SUMMARY: The biopsy-proven vertebral body lesion immediately upstages the patient to stage 4, metastatic breast cancer. The treatment goal shifts from curative to palliative. Adjuvant therapy and local-regional therapies to the breast and axilla are no longer indicated. For ER+/HER2- metastatic breast cancer, the standard of care is a combination of an aromatase inhibitor and a CDK4/6 inhibitor, as demonstrated by the MONALEESA, PALOMA, and NATALEE trials. The OncotypeDX score is irrelevant in this setting.
KEY_REFERENCES: MONALEESA-2, -3, -7 trials (various publications in NEJM); PALOMA-2, -3 trials (various publications in NEJM); NATALEE trial (ASCO 2023 abstract).
GUIDELINE_ALIGNMENT: NCCN and other international guidelines (up to Feb 2025) recommend AI + CDK4/6i as the first-line treatment for de novo metastatic, HR+ breast cancer.
SUBGROUP_NOTES: The diagnosis of metastatic disease supersedes all previous treatment plans.
CONFIDENCE_LEVEL: High
WEB_SEARCH_USED: Yes - Searched "CDK4/6 inhibitor metastatic breast cancer guidelines," "NCCN metastatic breast cancer." The search confirmed that this is the standard of care for de novo metastatic HR+ disease.
---END---
QUESTION_ID: 81
SELECTED_ANSWER: A
EVIDENCE_SUMMARY: For patients with very limited metastatic disease (oligometastatic disease), there is growing evidence to support the use of definitive local-regional treatment in addition to standard systemic therapy. The SABR-COMET trial and others have demonstrated that this approach can improve progression-free and potentially overall survival in highly selected patients. While not a standard of care for all metastatic patients, it is a strongly considered approach, especially when the initial response to systemic therapy is good.
KEY_REFERENCES: SABR-COMET trial (Lancet 2018); ongoing clinical trials and meta-analyses on oligometastatic breast cancer.
GUIDELINE_ALIGNMENT: The concept of treating oligometastatic disease

is recognized in NCCN guidelines and other expert consensus statements as a valid approach in a multidisciplinary setting, though it is often considered in the context of a clinical trial.
SUBGROUP_NOTES: This approach is only for highly selected patients with a limited number of metastatic sites and a good response to initial systemic therapy.
CONFIDENCE_LEVEL: High
WEB_SEARCH_USED: Yes - Searched "oligometastatic breast cancer definitive local therapy," and "SABR-COMET breast cancer." The search confirmed the growing evidence for this approach.
---END---
