# Supplementary material for: Performance of large language models in ambiguous treatment scenarios of early breast cancer
Source: Breast. 2026 Sep 23;90:104932. doi: 10.1016/j.breast.2026.104932 (PMC13641045; doi:10.1016/j.breast.2026.104932)
Supplement: Supplementary file 1 [file mmc1.zip › Suppl.Mat. - Anton Oseledchyk - Prompts, LLM Responses, R-Code/her2pos_response_chatgpt.pdf]

QUESTION_ID: 34
SELECTED_ANSWER: E
EVIDENCE_SUMMARY: Microinvasive (T1mi, ≤1 mm) HER2-positive breast cancer generally has excellent outcomes, and current literature/ guidelines do not show clear benefit for adjuvant chemotherapy/anti-HER2 therapy in this setting. While multiple foci of microinvasion can correlate with somewhat higher risk, studies have not demonstrated improved outcomes from paclitaxel/trastuzumab for T1mi, so systemic therapy is typically not recommended. ([PMC][1], [Frontiers][2])
KEY_REFERENCES: Johnson et al. “Treatment of small (T1mic, T1a/b) node-negative HER2+ BC” (review). ([PMC][3]); Song et al. “Multiple microinvasion foci in DCIS and outcomes” (Frontiers Oncol 2020). ([Frontiers][2]); Comparative summary noting guidelines generally do not recommend systemic therapy for MIBC. ([PMC][1])
GUIDELINE_ALIGNMENT: Consistent with ESMO Early Breast Cancer guideline (2024) principles for very small tumors (systemic therapy often unnecessary) and with common NCCN practice patterns for T1mi (no routine systemic therapy). ([PubMed][4], [nccn.org][5])
SUBGROUP_NOTES: Consider exceptions only in unusual, very high-risk biology (e.g., age ≤40 with extensive microinvasion) after multidisciplinary review; evidence for benefit remains lacking. ([PMC][1])
CONFIDENCE_LEVEL: Moderate
WEB_SEARCH_USED: Yes — searched “HER2 microinvasive guidelines”, “T1mi HER2 outcomes”, “multiple foci microinvasion”; found reviews and cohort data showing no proven benefit to adjuvant TH for T1mi and that multifocality increases risk but without therapy benefit. ([PMC][1], [Frontiers][2])
\---END---

QUESTION_ID: 35
SELECTED_ANSWER: C
EVIDENCE_SUMMARY: For a 2.4 cm, node-negative HER2-positive tumor after upfront surgery, multi-agent non-anthracycline chemotherapy with trastuzumab (TCbH/TCH) is a standard adjuvant option with efficacy comparable to anthracycline-containing regimens and less cardiotoxicity. Adding pertuzumab in the adjuvant setting provides benefit mainly for node-positive disease; node-negative cohorts do well without it. ([PMC][6], [PubMed][7])
KEY_REFERENCES: BCIRG-006 (establishing TCH as effective with lower cardiac risk). ([PMC][6]); APHINITY 8-year update (node-negative subgroup had no added benefit from pertuzumab). ([PubMed][7]); NCCN 2024 educational summary of adjuvant HER2-positive options. ([education.nccn.org][8])
GUIDELINE_ALIGNMENT: NCCN Breast Cancer 2024 and ESMO 2024 endorse adjuvant trastuzumab-based chemotherapy for T2N0 disease, with non-anthracycline regimens acceptable; pertuzumab reserved for higher-risk (especially node-positive). ([education.nccn.org][8], [PubMed][4])
SUBGROUP_NOTES: Consider cardiac comorbidities (favoring TCH) and patient preference; TH is typically reserved for smaller, stage I tumors (APT-like), not T2. ([The Lancet][9])
CONFIDENCE_LEVEL: High

WEB_SEARCH_USED: Yes – searched “BCIRG-006 TCH vs AC-TH,” “APHINITY node-negative subgroup,” and NCCN 2024 adjuvant HER2 guidance; results support TCbH without pertuzumab for N0. ([PMC][6], [PubMed][7], [education.nccn.org][8])
\---END---

QUESTION_ID: 36
SELECTED_ANSWER: C
EVIDENCE_SUMMARY: For residual invasive disease after neoadjuvant HER2-targeted chemotherapy, adjuvant T-DM1 significantly reduces recurrence risk versus trastuzumab (KATHERINE) and is standard regardless of biomarker shifts at surgery. Exploratory analyses show T-DM1 benefit is maintained across HER2 expression subgroups, including cases with lower HER2 in the residual tumor. ([education.nccn.org][8], [PMC][10])
KEY_REFERENCES: KATHERINE trial (NEJM 2019) – T-DM1 superior to trastuzumab for residual disease. ([education.nccn.org][8]); KATHERINE biomarker analysis (2023) – benefit largely independent of HER2 expression in residual disease. ([PMC][10])
GUIDELINE_ALIGNMENT: NCCN 2024 lists adjuvant T-DM1 for 14 cycles (Category 1) for residual disease after neoadjuvant therapy. ([education.nccn.org][8])
SUBGROUP_NOTES: Continue HER2-targeted therapy backbone with T-DM1 even if post-treatment HER2 retesting appears negative; manage toxicities (LFTs, thrombocytopenia, neuropathy). ([PMC][10])
CONFIDENCE_LEVEL: High
WEB_SEARCH_USED: Yes – searched “KATHERINE residual disease HER2 loss,” “NCCN 2024 T-DM1 adjuvant”; confirms T-DM1 recommendation regardless of HER2 retesting. ([PMC][10], [education.nccn.org][8])
\---END---

QUESTION_ID: 37
SELECTED_ANSWER: D
EVIDENCE_SUMMARY: For stage II (3 cm), ER-negative, HER2-positive disease, neoadjuvant multi-agent chemotherapy with dual HER2 blockade (TCbHP/TCHP) is preferred; anthracyclines add toxicity without improving outcomes when carboplatin and HP are used (TRAIN-2). Although HER2DX provides prognostic/pCR information, it is not yet guideline-endorsed to de-escalate neoadjuvant therapy in this setting. ([JAMA Network][11], [jnccn.org][12], [ESMO Open][13])
KEY_REFERENCES: TRAIN-2 randomized trial (anthracycline-free vs anthracycline-containing; similar EFS/OS, less cardiotoxicity). ([JAMA Network][11]); JNCCN review noting neoadjuvant preferred for ≥T2 or node-positive HER2+ disease. ([jnccn.org][12]); HER2DX analytical/clinical validation (not yet treatment-guiding). ([ESMO Open][13])
GUIDELINE_ALIGNMENT: ESMO 2024 endorses neoadjuvant chemo + dual HER2 blockade for stage II–III HER2+ disease; NCCN materials reflect TCHP as a common preferred regimen. ([PubMed][4], [education.nccn.org][8])
SUBGROUP_NOTES: HR-negative status and tumor size strengthen the case for full-intensity neoadjuvant TCbHP; consider THP in select lower-risk HR+ cases, but not ER-negative with 3 cm. ([JAMA Network][11])

CONFIDENCE_LEVEL: High
WEB_SEARCH_USED: Yes — searched “TRAIN-2 outcomes,” “neoadjuvant preferred ≥T2 HER2+,” and “HER2DX validation”; confirms TCbHP preference and non-directive status of HER2DX. ([JAMA Network][11], [jnccn.org][12], [ESMO Open][13])
\---END---

QUESTION_ID: 38
SELECTED_ANSWER: A
EVIDENCE_SUMMARY: In routine stage II/III HER2-positive breast cancer, an anthracycline-free neoadjuvant regimen with docetaxel/carboplatin plus trastuzumab/pertuzumab (TCbHP/TCHP) is preferred due to similar efficacy and lower cardiotoxicity compared with anthracycline-containing strategies. Randomized data (TRAIN-2) show no added benefit from anthracyclines when carboplatin and dual HER2 blockade are used. ([JAMA Network][11])
KEY_REFERENCES: TRAIN-2 randomized trial (JAMA Oncol 2021) — comparable EFS/OS without anthracyclines; toxicity favored anthracycline-free. ([JAMA Network][11]); Meta-analyses/reviews supporting anthracycline-free approaches with lower cardiac events. ([PMC][14])
GUIDELINE_ALIGNMENT: ESMO 2024 and NCCN educational materials: for stage II–III HER2+ EBC, neoadjuvant dual HER2 blockade + chemotherapy is standard, commonly using TCHP. ([PubMed][4], [education.nccn.org][8])
SUBGROUP_NOTES: Consider anthracyclines only in special high-risk scenarios or clinical trials; baseline cardiac risk further favors anthracycline-free regimens. ([PMC][14])
CONFIDENCE_LEVEL: High
WEB_SEARCH_USED: Yes — searched “TRAIN-2 anthracycline-free TCHP,” “ESMO 2024 early HER2 neoadjuvant,” “NCCN 2024 neoadjuvant HER2+”; all support TCbHP as preferred without adding AC. ([JAMA Network][11], [PubMed][4], [education.nccn.org][8])
\---END---

[1]: https://pmc.ncbi.nlm.nih.gov/articles/PMC11462587/?utm_source=chatgpt.com "A Comparative Study With Tis-T1 Breast Cancer - PMC"
[2]: https://www.frontiersin.org/journals/oncology/articles/10.3389/fonc.2020.607502/full?utm_source=chatgpt.com "Multiple Microinvasion Foci in Ductal Carcinoma In Situ Is ..."
[3]: https://pmc.ncbi.nlm.nih.gov/articles/PMC9156575/?utm_source=chatgpt.com "Treatment of small (T1mic, T1a, and T1b) node-negative ..."
[4]: https://pubmed.ncbi.nlm.nih.gov/38101773/?utm_source=chatgpt.com "Early breast cancer: ESMO Clinical Practice Guideline for ..."
[5]: https://www.nccn.org/guidelines/guidelines-detail?id=1419&utm_source=chatgpt.com "Breast Cancer - Guidelines Detail - NCCN"
[6]: https://pmc.ncbi.nlm.nih.gov/articles/PMC3446365/?utm_source=chatgpt.com "Adjuvant treatment of HER2-positive breast cancer"
[7]: https://pubmed.ncbi.nlm.nih.gov/39259927/?

utm_source=chatgpt.com "Adjuvant Pertuzumab and Trastuzumab in Early Human ..."
[8]: https://education.nccn.org/sites/default/files/course/2024-02/03_Robinson_NCCNbc24_v3.pdf?utm_source=chatgpt.com "[PDF] Neoadjuvant/Adjuvant Treatment for HER2-Positive Breast Cancer ..."
[9]: https://www.thelancet.com/journals/lanonc/article/PIIS1470-2045%2823%2900051-7/abstract?utm_source=chatgpt.com "Adjuvant paclitaxel and trastuzumab for node-negative ..."
[10]: https://pmc.ncbi.nlm.nih.gov/articles/PMC10102844/ " Biomarker Data from the Phase III KATHERINE Study of Adjuvant T-DM1 versus Trastuzumab for Residual Invasive Disease after Neoadjuvant Therapy for HER2-Positive Breast Cancer - PMC "
[11]: https://jamanetwork.com/journals/jamaoncology/fullarticle/2780055?utm_source=chatgpt.com "Three-Year Follow-up of Neoadjuvant Chemotherapy With ..."
[12]: https://jnccn.org/view/journals/jnccn/20/6/article-p723.xml?print=&result=1&rskey=ZnZPLT&utm_source=chatgpt.com "The Evolving Role of Neoadjuvant Therapy for Operable ..."
[13]: https://www.esmoopen.com/article/S2059-7029%2824%2900671-9/fulltext?utm_source=chatgpt.com "Analytical validation of HER2DX genomic test for early-stage HER2 ..."
[14]: https://pmc.ncbi.nlm.nih.gov/articles/PMC10061451/?utm_source=chatgpt.com "Neoadjuvant therapy with vs. without anthracyclines for ..."
