# Supplementary material for: Performance of large language models in ambiguous treatment scenarios of early breast cancer
Source: Breast. 2026 Sep 23;90:104932. doi: 10.1016/j.breast.2026.104932 (PMC13641045; doi:10.1016/j.breast.2026.104932)
Supplement: Supplementary file 1 [file mmc1.zip › Suppl.Mat. - Anton Oseledchyk - Prompts, LLM Responses, R-Code/her2pos_response_claude.pdf]

QUESTION_ID: 34
SELECTED_ANSWER: A
EVIDENCE_SUMMARY: Studies show that even small HER2-positive tumors including microinvasive disease benefit from adjuvant trastuzumab-based therapy, with significant improvements in disease-free survival. Adjuvant trastuzumab reduces recurrence risk even in microinvasive HER2-positive tumors, particularly in hormone receptor-negative cases which have the highest risk.
KEY_REFERENCES: APT trial (Tolaney 2015), CancerLinQ database analysis 2024, Italian multicenter retrospective study 2021
GUIDELINE_ALIGNMENT: NCCN 2024 recommends considering adjuvant therapy for HER2+ microinvasive disease (category 2B)
SUBGROUP_NOTES: ER-negative status increases recurrence risk; even 1-2 foci warrant consideration given HER2 3+ status
CONFIDENCE_LEVEL: Moderate
WEB_SEARCH_USED: Yes
---END---

QUESTION_ID: 35
SELECTED_ANSWER: A
EVIDENCE_SUMMARY: The APT trial established paclitaxel/trastuzumab (TH) as highly effective for node-negative HER2-positive tumors up to 3cm, with 98.7% 3-year DFS. Pertuzumab adds minimal benefit in low-risk node-negative disease and increases toxicity without improving outcomes in this setting.
KEY_REFERENCES: APT trial (NEJM 2015), APHINITY subgroup analysis, ATEMPT trial
GUIDELINE_ALIGNMENT: NCCN 2024 supports TH for T2N0 disease; pertuzumab not required for node-negative tumors
SUBGROUP_NOTES: ER-positive, node-negative, 2.4cm tumor represents low-risk disease ideal for de-escalation
CONFIDENCE_LEVEL: High
WEB_SEARCH_USED: Yes
---END---

QUESTION_ID: 36
SELECTED_ANSWER: C
EVIDENCE_SUMMARY: The KATHERINE trial demonstrated that T-DM1 should be given to all patients with residual disease after neoadjuvant therapy, even those with HER2-negative residual disease, as these patients still derived significant benefit. With 8-year follow-up, patients with HER2-negative residual disease had 95.2% 7-year IDFS with T-DM1 versus 60.3% with trastuzumab.
KEY_REFERENCES: KATHERINE trial primary analysis and 8-year follow-up, KATHERINE biomarker substudy 2022
GUIDELINE_ALIGNMENT: FDA and NCCN guidelines support T-DM1 for all residual disease regardless of HER2 retesting
SUBGROUP_NOTES: HER2 loss in residual disease occurs in 8% but doesn't negate T-DM1 benefit
CONFIDENCE_LEVEL: High
WEB_SEARCH_USED: Yes
---END---

QUESTION_ID: 37

SELECTED_ANSWER: D
EVIDENCE_SUMMARY: HER2DX with low risk but high pCR score suggests excellent response to neoadjuvant therapy, with studies showing 80-90% pCR rates with full TCbHP regimen in high pCR score patients. For ER-negative HER2-positive tumors with high pCR likelihood, neoadjuvant TCbHP maximizes pCR probability.
KEY_REFERENCES: HER2DX validation studies 2024, PHERGain correlative analysis, Spanish real-world HER2DX study
GUIDELINE_ALIGNMENT: SEOM-GEICAM-SOLTI 2023 guidelines recognize HER2DX for treatment selection
SUBGROUP_NOTES: ER-negative status and high pCR score favor intensive neoadjuvant approach for maximum benefit
CONFIDENCE_LEVEL: Moderate
WEB_SEARCH_USED: Yes
---END---

QUESTION_ID: 38
SELECTED_ANSWER: A
EVIDENCE_SUMMARY: TCbHP has become the preferred neoadjuvant regimen for HER2-positive breast cancer based on KRISTINE and TRAIN-2 trials, showing high pCR rates without need for anthracyclines. NCCN guidelines list TCbHP as the preferred regimen, though recent data suggest THP may be adequate for selected patients.
KEY_REFERENCES: KRISTINE trial, TRAIN-2 trial, TRYPHAENA safety run-in
GUIDELINE_ALIGNMENT: NCCN 2024, CSCO 2024, and ESMO guidelines support TCbHP as standard
SUBGROUP_NOTES: Adding anthracyclines after TCbHP provides no additional benefit and increases toxicity
CONFIDENCE_LEVEL: High
WEB_SEARCH_USED: Yes
---END---
