# Supplementary material for: Performance of large language models in ambiguous treatment scenarios of early breast cancer
Source: Breast. 2026 Sep 23;90:104932. doi: 10.1016/j.breast.2026.104932 (PMC13641045; doi:10.1016/j.breast.2026.104932)
Supplement: Supplementary file 1 [file mmc1.zip › Suppl.Mat. - Anton Oseledchyk - Prompts, LLM Responses, R-Code/her2pos_response_deepseek.pdf]

QUESTION_ID: 34
SELECTED_ANSWER: A
EVIDENCE_SUMMARY: For HER2-positive microinvasive breast cancer, adjuvant paclitaxel and trastuzumab (TH) is recommended even with 1-2 foci based on the APT trial, which included tumors up to 3 cm (including microinvasion) and showed excellent outcomes. NCCN Guidelines (v3.2025) recommend considering adjuvant systemic therapy for HER2+ microinvasion, and recent evidence through February 2025 continues to support this approach due to the aggressive nature of HER2-positive disease, even with minimal invasion.
KEY_REFERENCES: APT trial; NCCN Guidelines Breast Cancer v3.2025
GUIDELINE_ALIGNMENT: NCCN (2025) guidelines recommend adjuvant therapy for HER2+ microinvasive disease, with no specific focus threshold.
SUBGROUP_NOTES: ER-negative status increases the aggressiveness and reinforces the need for anti-HER2 therapy. Extensive DCIS alone does not mandate chemotherapy, but microinvasion changes the paradigm.
CONFIDENCE_LEVEL: High
WEB_SEARCH_USED: Yes - Searched for NCCN Guidelines v3.2025 and recent microinvasion recommendations; no new trials through Feb 2025 change this standard.

---END---

QUESTION_ID: 35
SELECTED_ANSWER: B
EVIDENCE_SUMMARY: For a node-negative, ER+/HER2+ tumor >2 cm, the standard adjuvant regimen is THP (paclitaxel, trastuzumab, pertuzumab) based on the APT and BERENICE trials, which showed excellent outcomes. NCCN Guidelines (v3.2025) continue to recommend dual HER2 blockade with chemotherapy for tumors >2 cm, even in node-negative disease, due to the higher risk associated with larger tumor size.
KEY_REFERENCES: APT trial; BERENICE trial; NCCN Guidelines Breast Cancer v3.2025
GUIDELINE_ALIGNMENT: NCCN (2025) and ASCO guidelines recommend THP for node-negative HER2+ tumors >2 cm.
SUBGROUP_NOTES: ER positivity may modulate aggressiveness, but tumor size >2 cm warrants dual HER2 blockade. Genomic assays like Oncotype DX may help in select cases, but dual blockade remains standard.
CONFIDENCE_LEVEL: High
WEB_SEARCH_USED: Yes - Confirmed NCCN v3.2025 maintains recommendation for dual HER2 blockade for tumors >2 cm.

---END---

QUESTION_ID: 36
SELECTED_ANSWER: B
EVIDENCE_SUMMARY: For residual disease after neoadjuvant TCHP with HER2 loss (now HER2-negative), T-DM1 is not indicated as it requires HER2 positivity. The KATHERINE trial excluded HER2-negative residuals. NCCN Guidelines (v3.2025) recommend considering alternative chemotherapy (e.g., adjuvant AC) in this scenario, as the tumor may have transformed to a HER2-negative phenotype with

different sensitivity.
KEY_REFERENCES: KATHERINE trial; NCCN Guidelines Breast Cancer v3.2025
GUIDELINE_ALIGNMENT: NCCN (2025) guidelines state T-DM1 is only for HER2-positive residual disease; for HER2-negative residuals, consider alternative chemotherapy.
SUBGROUP_NOTES: HER2 loss after neoadjuvant therapy may indicate a more resistant clone; AC chemotherapy could address this. ER negativity limits endocrine options.
CONFIDENCE_LEVEL: Moderate
WEB_SEARCH_USED: Yes - Searched for guidelines on HER2 loss post-neoadjuvant; NCCN v3.2025 addresses this scenario and does not recommend T-DM1.

---END---

QUESTION_ID: 37
SELECTED_ANSWER: C
EVIDENCE_SUMMARY: For a 3 cm ER-negative HER2+ tumor, neoadjuvant therapy is standard to assess response. While HER2DX may suggest high pCR probability, the tumor size (3 cm) and ER-negative status warrant a robust regimen like THP (paclitaxel/trastuzumab/pertuzumab). NCCN Guidelines (v3.2025) recommend neoadjuvant dual HER2 blockade with chemotherapy for tumors >2 cm, and HER2DX is not yet standard for de-escalation decisions.
KEY_REFERENCES: KRISTINE trial; NCCN Guidelines Breast Cancer v3.2025
GUIDELINE_ALIGNMENT: NCCN (2025) guidelines recommend neoadjuvant TCHP or THP for stage II-III HER2+ breast cancer.
SUBGROUP_NOTES: ER negativity increases aggressiveness and pCR likelihood, but de-escalation to single-agent taxane/trastuzumab is not yet standard for tumors >2 cm.
CONFIDENCE_LEVEL: High
WEB_SEARCH_USED: Yes - Searched for HER2DX utility; NCCN v3.2025 does not yet incorporate HER2DX for routine clinical decisions.

---END---

QUESTION_ID: 38
SELECTED_ANSWER: A
EVIDENCE_SUMMARY: The preferred regimen for stage II-III HER2+ breast cancer is TCbHP (docetaxel/carboplatin/trastuzumab/pertuzumab) based on the TRAIN-2 and KRISTINE trials, which showed high pCR rates and favorable toxicity compared to anthracycline-containing regimens. NCCN Guidelines (v3.2025) endorse TCbHP as a category 1 recommendation, avoiding anthracyclines to reduce cardiotoxicity and leukemia risk.
KEY_REFERENCES: TRAIN-2 trial; KRISTINE trial; NCCN Guidelines Breast Cancer v3.2025
GUIDELINE_ALIGNMENT: NCCN (2025) and ASCO guidelines recommend TCbHP as a preferred regimen for stage II-III HER2+ breast cancer.
SUBGROUP_NOTES: Anthracyclines may be considered in very high-risk cases (e.g., high nodal burden), but TCbHP is generally preferred.
CONFIDENCE_LEVEL: High

WEB_SEARCH_USED: Yes - Confirmed NCCN v3.2025 maintains TCbHP as a category 1 recommendation for neoadjuvant/adjuvant treatment of HER2+ breast cancer.

---END---
