# Supplementary material for: Performance of large language models in ambiguous treatment scenarios of early breast cancer
Source: Breast. 2026 Sep 23;90:104932. doi: 10.1016/j.breast.2026.104932 (PMC13641045; doi:10.1016/j.breast.2026.104932)
Supplement: Supplementary file 1 [file mmc1.zip › Suppl.Mat. - Anton Oseledchyk - Prompts, LLM Responses, R-Code/her2pos_response_gemini.pdf]

QUESTION_ID: 34
SELECTED_ANSWER: E
EVIDENCE_SUMMARY: The patient has microinvasive breast cancer (T1mi), which is defined as a tumor size of 1 mm or less. The risk of axillary lymph node metastasis and distant recurrence is exceedingly low for T1mi, regardless of HER2 status. As of February 2025, major clinical guidelines do not recommend adjuvant systemic therapy, including paclitaxel and trastuzumab, for T1mi disease due to the low absolute risk of recurrence and the potential for treatment-related toxicity.
KEY_REFERENCES: NCCN Breast Cancer Guidelines (v1.2024); ASCO/CAP guidelines on HER2 testing; retrospective studies on outcomes of T1mi breast cancer.
GUIDELINE_ALIGNMENT: NCCN guidelines (versions through Feb 2025) explicitly state that for T1mi disease, adjuvant systemic therapy is not required.
SUBGROUP_NOTES: The HER2-positive status does not alter the management of this very low-risk stage of disease.
CONFIDENCE_LEVEL: High
WEB_SEARCH_USED: Yes - Searched "adjuvant trastuzumab microinvasive breast cancer," and "NCCN guidelines T1mi." Confirmed that the standard of care is to omit systemic therapy for this stage.
---END---
QUESTION_ID: 35
SELECTED_ANSWER: B
EVIDENCE_SUMMARY: The patient has a high-risk, node-negative (T2N0) HER2-positive breast cancer. The APHINITY trial demonstrated a significant benefit for adding pertuzumab to a docetaxel and trastuzumab (TH) regimen in the adjuvant setting for patients with high-risk disease. While the benefit was most pronounced in node-positive patients, it was also observed in the high-risk T2, N0 subset. Therefore, the addition of pertuzumab (THP) is the most evidence-based and aggressive adjuvant approach for this patient.
KEY_REFERENCES: APHINITY trial (NEJM 2017); NCCN Breast Cancer Guidelines (v1.2024).
GUIDELINE_ALIGNMENT: NCCN guidelines (versions through Feb 2025) recommend adjuvant pertuzumab as an option for patients with high-risk HER2-positive breast cancer, including those with T2, N0 disease, based on the APHINITY trial results.
SUBGROUP_NOTES: The patient's T2 tumor size (2.4 cm) places her in a higher-risk category within the node-negative population, for whom the addition of pertuzumab is most likely to be beneficial.
CONFIDENCE_LEVEL: High
WEB_SEARCH_USED: Yes - Searched "APHINITY trial T2N0 subgroup," and "NCCN guidelines adjuvant pertuzumab." Confirmed that the APHINITY trial supports the use of THP for high-risk N0 patients.
---END---
QUESTION_ID: 36
SELECTED_ANSWER: C
EVIDENCE_SUMMARY: For a patient who received neoadjuvant therapy for HER2-positive breast cancer and has residual disease at the time of surgery, the standard of care is to switch to adjuvant trastuzumab emtansine (T-DM1). The KATHERINE trial demonstrated a significant improvement in invasive disease-free survival with adjuvant T-DM1

compared to trastuzumab in this specific patient population. The change in HER2 status on repeat testing does not change this recommendation, as the decision is based on the original HER2 status and the presence of residual disease after neoadjuvant therapy.
KEY_REFERENCES: KATHERINE trial (NEJM 2018); NCCN Breast Cancer Guidelines (v1.2024).
GUIDELINE_ALIGNMENT: NCCN and other international guidelines (through Feb 2025) have incorporated the KATHERINE trial findings, making adjuvant T-DM1 the preferred treatment for patients with residual disease after neoadjuvant HER2-directed therapy.
SUBGROUP_NOTES: This recommendation is specifically for patients with residual invasive disease after neoadjuvant therapy, regardless of the initial subtype or HER2 status on post-surgical pathology.
CONFIDENCE_LEVEL: High
WEB_SEARCH_USED: Yes - Searched "KATHERINE trial results," "adjuvant T-DM1 residual disease HER2." Confirmed that T-DM1 is the standard of care in this setting prior to the cutoff date.
---END---
QUESTION_ID: 37
SELECTED_ANSWER: E
EVIDENCE_SUMMARY: The HER2DX test is a prognostic genomic assay for HER2-positive breast cancer, but as of February 2025, it is not part of standard clinical practice guidelines (e.g., NCCN, ASCO) for making definitive treatment decisions, particularly for de-escalation of therapy in a high-risk patient. A 3 cm HER2-positive breast cancer (T2) is considered high-risk, and the standard of care is to recommend neoadjuvant chemotherapy with dual HER2 blockade to maximize the chance of a pathological complete response. The use of a novel genomic test to omit this established, effective therapy would not be recommended.
KEY_REFERENCES: NCCN Breast Cancer Guidelines (v1.2024); KEYNOTE-522, TRYPHAENA, and other landmark trials establishing neoadjuvant therapy for high-risk HER2-positive cancer.
GUIDELINE_ALIGNMENT: As of Feb 2025, major guidelines do not support using the HER2DX test to change the standard-of-care neoadjuvant approach for a T2, HER2-positive tumor.
SUBGROUP_NOTES: The tumor size is the key risk factor here, making neoadjuvant therapy the standard regardless of the HER2DX score.
CONFIDENCE_LEVEL: High
WEB_SEARCH_USED: Yes - Searched "HER2DX clinical use guidelines," "NCCN guidelines HER2DX." Confirmed that the test is not yet incorporated into standard treatment algorithms.
---END---
QUESTION_ID: 38
SELECTED_ANSWER: A
EVIDENCE_SUMMARY: For stage 2 or 3 HER2-positive breast cancer, neoadjuvant therapy is the standard of care to achieve a pathological complete response (pCR), which is associated with improved long-term outcomes. The most effective and widely adopted neoadjuvant regimen, based on trials like TRYPHAENA and APHINITY, is a combination of a taxane, carboplatin, trastuzumab, and pertuzumab (TCHP). This regimen offers the highest pCR rates among standard options.
KEY_REFERENCES: TRYPHAENA trial (Lancet Oncol 2013); APHINITY trial

(NEJM 2017); NCCN Breast Cancer Guidelines (v1.2024).
GUIDELINE_ALIGNMENT: NCCN guidelines (versions through Feb 2025) recommend a taxane, carboplatin, and dual HER2 blockade (TCHP) as a preferred neoadjuvant regimen for stage 2 and 3 HER2-positive breast cancer.
SUBGROUP_NOTES: This regimen is the standard of care for these stages and is suitable for most patients who can tolerate the combination therapy.
CONFIDENCE_LEVEL: High
WEB_SEARCH_USED: Yes - Searched "preferred neoadjuvant regimen HER2+," and "TCHP vs TCHP then AC." The search confirmed that TCHP is a standard, complete neoadjuvant regimen and not followed by AC.
---END---
