# Supplementary material for: Performance of large language models in ambiguous treatment scenarios of early breast cancer
Source: Breast. 2026 Sep 23;90:104932. doi: 10.1016/j.breast.2026.104932 (PMC13641045; doi:10.1016/j.breast.2026.104932)
Supplement: Supplementary file 1 [file mmc1.zip › Suppl.Mat. - Anton Oseledchyk - Prompts, LLM Responses, R-Code/hrpos_response_chatgpt.pdf]

QUESTION_ID: 39
SELECTED_ANSWER: B
EVIDENCE_SUMMARY: In ER+ early breast cancer, adjuvant chemotherapy is typically reserved for patients with a meaningful absolute benefit; many centers and decision tools (e.g., PREDICT) stratify <3% as low, 3–5% as intermediate, and >5% as high 10-year survival benefit—favoring chemotherapy when ≥5%. This aligns with contemporary guidance using multigene assays to estimate benefit and reserve chemotherapy for those with sufficient absolute gain. ([NICE][1], [breast.predict.cam][2], [ASC Publications][3])
KEY_REFERENCES: PREDICT tool documentation; ASCO 2022 Biomarkers guideline; NICE evidence review on tumor profiling/benefit thresholds (2024). ([breast.v3.predict.cam][4], [ASC Publications][3], [NICE][1])
GUIDELINE_ALIGNMENT: ASCO 2022 Biomarkers guideline supports using multigene assays to estimate chemotherapy benefit in node-negative/limited node-positive disease. ([ASC Publications][3])
SUBGROUP_NOTES: Patient values/comorbidity matter when absolute benefit is 3–5%; younger age or high clinical risk may lower the threshold. ([PMC][5])
CONFIDENCE_LEVEL: Moderate
WEB_SEARCH_USED: Yes — searched “PREDICT breast tool benefit threshold”, “NICE chemotherapy absolute benefit threshold”, “ASCO 2022 biomarkers”; found NICE categorization of <3%/3–5%/>5% and ASCO guidance confirming assay-guided selection (through Feb 2025).
\---END---

QUESTION_ID: 40
SELECTED_ANSWER: B
EVIDENCE_SUMMARY: Although many T1 (≤1 cm), node-negative ER+/HER2- tumors have low distant-recurrence risk, it is not uniformly negligible and genomic assays refine who benefits from chemotherapy. ASCO 2022 recommends Oncotype DX (and other assays) for node-negative ER+/HER2- cancers; NSABP B-21 primarily addressed local control with RT/TAM and does not obviate systemic risk assessment. ([ASC Publications][3], [MDPI][6])
KEY_REFERENCES: TAILORx (NEJM 2018/2019 exploratory analyses); ASCO 2022 Biomarkers guideline; NSABP B-21/related overviews of small-tumor local control. ([New England Journal of Medicine][7], [MDPI][6])
GUIDELINE_ALIGNMENT: ASCO 2022 Biomarkers endorses genomic testing in node-negative ER+/HER2- disease to guide chemo decisions; NCCN and ESMO similarly incorporate assays. ([ASC Publications][3])
SUBGROUP_NOTES: Younger patients and higher-grade/lymphovascular invasion may justify testing even for small tumors. ([New England Journal of Medicine][8])
CONFIDENCE_LEVEL: High
WEB_SEARCH_USED: Yes — searched “ASCO 2022 biomarkers early breast cancer,” “NSABP B-21 distant recurrence,” “TAILORx small tumors”; found ASCO guideline endorsing assays and B-21 focusing on local control (through Feb 2025).
\---END---

QUESTION_ID: 42

SELECTED_ANSWER: B
EVIDENCE_SUMMARY: For cT3N1, low-grade, strongly ER+/PR+ tumors (often luminal A, including lobular), neoadjuvant endocrine therapy (NET) is effective for downstaging and breast/axilla management, with ACOSOG Z1031 showing robust surgical conversion and biologic response to aromatase inhibitors. ESMO 2024 supports NET in postmenopausal, endocrine-sensitive tumors when chemotherapy benefit is expected to be low. ([PubMed][9])
KEY_REFERENCES: ACOSOG Z1031 (Ellis et al.); ESMO Early Breast Cancer Guideline 2024. ([PubMed][9])
GUIDELINE_ALIGNMENT: ESMO 2024 endorses NET for postmenopausal, endocrine-sensitive cT2–3 disease to improve operability. ([PubMed][10])
SUBGROUP_NOTES: Monitor early Ki-67 to confirm endocrine sensitivity; poor early decline may trigger switch to chemo. ([PubMed][11])
CONFIDENCE_LEVEL: High
WEB_SEARCH_USED: Yes — searched “ACOSOG Z1031,” “ESMO 2024 early breast neoadjuvant endocrine”; confirmed NET indications (through Feb 2025).
\---END---

QUESTION_ID: 43
SELECTED_ANSWER: B
EVIDENCE_SUMMARY: With a low genomic risk score in a postmenopausal woman with cT3N1, endocrine sensitivity is high; NET is favored to downstage, consistent with data from Z1031 and modern guidelines. Chemotherapy is generally deferred initially when genomic risk is low and clinical features suggest endocrine-responsive biology. ([PubMed][9])
KEY_REFERENCES: ACOSOG Z1031; ESMO 2024 Early Breast Cancer Guideline. ([PubMed][9])
GUIDELINE_ALIGNMENT: ESMO 2024 supports NET in postmenopausal, low-proliferative ER+ disease. ([PubMed][10])
SUBGROUP_NOTES: If inadequate response to NET (e.g., Ki-67 remains high), consider switching to chemotherapy. ([PubMed][11])
CONFIDENCE_LEVEL: Moderate
WEB_SEARCH_USED: Yes — “ESMO 2024 early breast endocrine therapy,” “ACOSOG Z1031” (updates through Feb 2025).
\---END---

QUESTION_ID: 44
SELECTED_ANSWER: B
EVIDENCE_SUMMARY: After NET with substantial response but residual disease including 3/11 positive nodes, a low genomic score suggests limited chemotherapy benefit in postmenopausal patients. RxPONDER shows postmenopausal women with 1–3 positive nodes and RS≤25 do not benefit from chemotherapy, supporting no adjuvant chemotherapy here. ([New England Journal of Medicine][12], [swog.org][13])
KEY_REFERENCES: RxPONDER (NEJM 2021); ASCO 2022 Biomarkers guideline. ([New England Journal of Medicine][12], [ASC Publications][3])
GUIDELINE_ALIGNMENT: ASCO 2022 recognizes lack of chemo benefit for postmenopausal N1 with RS≤25; endocrine therapy remains the

mainstay. ([ASC Publications][3])
SUBGROUP_NOTES: Consider optimal endocrine strategy and bone health; radiation per standard indications.
CONFIDENCE_LEVEL: Moderate
WEB_SEARCH_USED: Yes – “RxPONDER NEJM postmenopausal no benefit,” “ASCO 2022 biomarkers” (through Feb 2025).
\---END---

QUESTION_ID: 45
SELECTED_ANSWER: B
EVIDENCE_SUMMARY: For ER+ HER2- with one positive node, extended endocrine therapy beyond 5 years reduces late recurrences; trials/meta-analyses suggest 7–8 years is often optimal for intermediate risk, reserving 10 years for highest-risk groups. ASCO guidance and extended AI/tamoxifen trials support tailoring duration by nodal burden and risk. ([PMC][14], [New England Journal of Medicine][15])
KEY_REFERENCES: NSABP B-42 10-year update; MA.17R; ATLAS/aTTom (tamoxifen 10y vs 5y). ([PMC][16], [New England Journal of Medicine][15], [The Lancet][17])
GUIDELINE_ALIGNMENT: ASCO endocrine therapy guidance recommends extended therapy, typically 7–10 years, individualized by risk. ([ASC Publications][18])
SUBGROUP_NOTES: Balance benefits vs bone/cardiovascular toxicity; use AI in postmenopause when feasible. ([New England Journal of Medicine][15])
CONFIDENCE_LEVEL: High
WEB_SEARCH_USED: Yes – “NSABP B-42 10-year,” “MA.17R NEJM,” “ATLAS tamoxifen 10 years” (through Feb 2025).
\---END---

QUESTION_ID: 46
SELECTED_ANSWER: B
EVIDENCE_SUMMARY: Node-negative ER+/HER2- with high genomic risk (e.g., RS 28) has elevated late-recurrence risk; extended ET to \~7–8 years provides additional protection vs 5 years, with 10 years reserved for higher clinical risk. Trials (MA.17R, NSABP B-42) support benefit of extension, balanced against toxicity. ([New England Journal of Medicine][15], [PMC][16])
KEY_REFERENCES: MA.17R (NEJM 2016); NSABP B-42 10-year outcomes. ([New England Journal of Medicine][15], [PMC][16])
GUIDELINE_ALIGNMENT: ASCO recommends considering extended duration (7–10y) in higher-risk node-negative disease based on genomic risk. ([ASC Publications][18])
SUBGROUP_NOTES: Bone health monitoring is key; BCI can help decide on extension in select cases. ([ASC Publications][3])
CONFIDENCE_LEVEL: Moderate
WEB_SEARCH_USED: Yes – “extended endocrine therapy NSABP B-42,” “MA.17R” (through Feb 2025).
\---END---

QUESTION_ID: 47
SELECTED_ANSWER: C
EVIDENCE_SUMMARY: With 3 positive nodes, late-recurrence risk is high; extended endocrine therapy for a total of 10 years provides

maximal benefit in node-positive, high-risk patients. ATLAS/aTTom and AI extension trials support longer duration in such settings despite toxicity trade-offs. ([The Lancet][17], [PMC][16])
KEY_REFERENCES: ATLAS; NSABP B-42 10-year update; MA.17R. ([The Lancet][17], [PMC][16], [New England Journal of Medicine][15])
GUIDELINE_ALIGNMENT: ASCO guidance recommends up to 10 years for node-positive/high-risk patients. ([ASC Publications][18])
SUBGROUP_NOTES: Manage osteoporosis risk proactively with AI-based extension. ([New England Journal of Medicine][15])
CONFIDENCE_LEVEL: High
WEB_SEARCH_USED: Yes — “ATLAS,” “NSABP B-42 10-year,” “MA.17R” (through Feb 2025).
\---END---

QUESTION_ID: 48
SELECTED_ANSWER: B
EVIDENCE_SUMMARY: For postmenopausal T2N0 ER+/HER2-, RS 28 (“High 1”), chemotherapy is indicated; non-anthracycline TC×4–6 is effective and avoids anthracycline toxicity. US Oncology 9735 showed TC superiority over AC, and guidelines accept TC as a standard option in HR+ HER2- disease. ([PubMed][19])
KEY_REFERENCES: US Oncology 9735 (TC vs AC); EBCTCG meta-analysis on regimen effects (context). ([PubMed][19])
GUIDELINE_ALIGNMENT: NCCN/ESMO list both TC and anthracycline-taxane regimens as appropriate; regimen choice individualized. ([ESMO Open][20])
SUBGROUP_NOTES: Consider cardiac risk and patient preference when choosing to avoid anthracyclines.
CONFIDENCE_LEVEL: Moderate
WEB_SEARCH_USED: Yes — “US Oncology 9735 TC vs AC,” “anthracycline taxane EBCTCG” (through Feb 2025).
\---END---

QUESTION_ID: 49
SELECTED_ANSWER: A
EVIDENCE_SUMMARY: With RS 32 (“High 2”) and T2N0 ER+/HER2-, anthracycline-taxane (AC→T) is reasonable to maximize efficacy given higher recurrence risk; EBCTCG analyses show anthracycline-taxane regimens provide the greatest benefit at higher baseline risk. Non-anthracycline options remain acceptable for patients with contraindications. ([PubMed][21])
KEY_REFERENCES: EBCTCG chemotherapy meta-analysis; dose-intensity meta-analysis. ([PubMed][21], [The Lancet][22])
GUIDELINE_ALIGNMENT: NCCN/ESMO allow AC→T or TC; higher-risk profiles often receive anthracycline-taxane. ([ESMO Open][20])
SUBGROUP_NOTES: Cardiac comorbidities may favor TC despite high RS.
CONFIDENCE_LEVEL: Moderate
WEB_SEARCH_USED: Yes — “EBCTCG anthracycline taxane meta-analysis,” “dose-intensity meta-analysis” (through Feb 2025).
\---END---

QUESTION_ID: 50
SELECTED_ANSWER: A
EVIDENCE_SUMMARY: For 1.8 cm grade 3, node-negative ER+/HER2- with

RS 32, high biologic risk supports AC→T to maximize recurrence reduction, consistent with meta-analytic evidence for anthracycline-taxane benefit in higher-risk disease. ([PubMed][21])
KEY_REFERENCES: EBCTCG chemotherapy meta-analysis; US Oncology 9735 (context for non-anthracycline). ([PubMed][21])
GUIDELINE_ALIGNMENT: NCCN/ESMO permit AC→T for high-risk node-negative disease. ([ESMO Open][20])
SUBGROUP_NOTES: Consider TC if significant anthracycline contraindications.
CONFIDENCE_LEVEL: Moderate
WEB_SEARCH_USED: Yes — “EBCTCG early breast anthracycline taxane,” “US Oncology 9735” (through Feb 2025).
\---END---

QUESTION_ID: 51
SELECTED_ANSWER: A
EVIDENCE_SUMMARY: Postmenopausal T1cN1 (3/11 nodes) with RS 13 falls within RxPONDER’s postmenopausal cohort showing no chemo benefit for RS≤25 despite 1–3 positive nodes; endocrine therapy alone is appropriate. ([New England Journal of Medicine][12], [swog.org][13])
KEY_REFERENCES: RxPONDER (NEJM 2021). ([New England Journal of Medicine][12])
GUIDELINE_ALIGNMENT: ASCO 2022 Biomarkers and practice commonly omit chemo in this setting. ([ASC Publications][3])
SUBGROUP_NOTES: Radiation and optimal endocrine therapy per standard; consider bone protection with AI.
CONFIDENCE_LEVEL: High
WEB_SEARCH_USED: Yes — “RxPONDER postmenopausal RS ≤25 nodes” (through Feb 2025).
\---END---

QUESTION_ID: 52
SELECTED_ANSWER: C
EVIDENCE_SUMMARY: With ≥4 positive nodes (N2), data from RxPONDER do not apply; high nodal burden predicts substantial chemotherapy benefit in ER+ disease. Anthracycline-taxane regimens improve outcomes in such high-risk patients. ([PubMed][21])
KEY_REFERENCES: EBCTCG meta-analysis; general chemo benefit in node-positive disease. ([PubMed][21])
GUIDELINE_ALIGNMENT: NCCN/ESMO recommend chemotherapy for ≥4 nodes positive.
SUBGROUP_NOTES: Consider cardiac risk in anthracycline selection.
CONFIDENCE_LEVEL: High
WEB_SEARCH_USED: Yes — “EBCTCG meta-analysis high nodal burden” (through Feb 2025).
\---END---

QUESTION_ID: 53
SELECTED_ANSWER: A
EVIDENCE_SUMMARY: In a 42-year-old with node-negative ER+/HER2- and RS 12, TAILORx shows chemotherapy is not beneficial; endocrine therapy alone is appropriate. For low-risk premenopausal patients, tamoxifen alone is reasonable per SOFT/TEXT when clinical risk is

low. ([New England Journal of Medicine][7])
KEY_REFERENCES: TAILORx (NEJM 2018); SOFT/TEXT long-term updates. ([New England Journal of Medicine][7], [PubMed][23])
GUIDELINE_ALIGNMENT: ASCO 2022 Biomarkers endorses omitting chemo for RS<16; endocrine therapy individualized. ([ASC Publications][3])
SUBGROUP_NOTES: If higher clinical risk features existed, OFS±AI could be considered, but not required here. ([PubMed][23])
CONFIDENCE_LEVEL: High
WEB_SEARCH_USED: Yes — “TAILORx age effect,” “SOFT/TEXT update” (through Feb 2025).
\---END---

QUESTION_ID: 54
SELECTED_ANSWER: C
EVIDENCE_SUMMARY: For RS 18 at age 42 (node-negative), TAILORx suggests small chemo benefit in women ≤50 likely due to ovarian suppression; SOFT/TEXT demonstrate superior outcomes with OFS+AI over tamoxifen. Thus, OFS+AI is preferred over chemotherapy for many such patients. ([New England Journal of Medicine][8])
KEY_REFERENCES: TAILORx exploratory age analysis; SOFT/TEXT combined analysis. ([New England Journal of Medicine][8], [PubMed][23])
GUIDELINE_ALIGNMENT: ASCO 2022 Biomarkers notes chemo benefit in premenopausal may reflect OFS; OFS+AI is endorsed for higher-risk premenopausal ER+ disease. ([ASC Publications][3])
SUBGROUP_NOTES: Discuss fertility and OFS side effects.
CONFIDENCE_LEVEL: High
WEB_SEARCH_USED: Yes — “TAILORx age ≤50 RS 16–25”, “SOFT/TEXT DFS benefit” (through Feb 2025).
\---END---

QUESTION_ID: 55
SELECTED_ANSWER: D
EVIDENCE_SUMMARY: For RS 23 at age 42, TAILORx shows a clinically meaningful benefit from chemotherapy in women ≤50 with RS 21–25; therefore, chemo followed by endocrine therapy is recommended. OFS+AI alone may not fully replicate the benefit at this higher RS. ([New England Journal of Medicine][8])
KEY_REFERENCES: TAILORx (NEJM 2019 exploratory analysis). ([New England Journal of Medicine][8])
GUIDELINE_ALIGNMENT: ASCO 2022 Biomarkers acknowledges chemo benefit in premenopausal RS≤25 with node-negative disease at higher RS bands. ([ASC Publications][3])
SUBGROUP_NOTES: Add OFS+AI after chemo for maximal endocrine effect if tolerated. ([New England Journal of Medicine][24])
CONFIDENCE_LEVEL: High
WEB_SEARCH_USED: Yes — “TAILORx age effect RS 21–25” (through Feb 2025).
\---END---

QUESTION_ID: 56
SELECTED_ANSWER: D
EVIDENCE_SUMMARY: In premenopausal women with 1 positive node and RS 12, RxPONDER shows chemo benefit regardless of RS (0–25);

therefore, chemotherapy followed by endocrine therapy is indicated. ([New England Journal of Medicine][12])
KEY_REFERENCES: RxPONDER (NEJM 2021). ([New England Journal of Medicine][12])
GUIDELINE_ALIGNMENT: ASCO 2022 Biomarkers: premenopausal N1 derive benefit from chemo irrespective of RS≤25. ([ASC Publications][3])
SUBGROUP_NOTES: Consider OFS+AI as part of endocrine therapy after chemo. ([New England Journal of Medicine][24])
CONFIDENCE_LEVEL: High
WEB_SEARCH_USED: Yes — “RxPONDER premenopausal benefit” (through Feb 2025).
---END---

QUESTION_ID: 57
SELECTED_ANSWER: D
EVIDENCE_SUMMARY: Same rationale—premenopausal with one positive node and RS 18 benefits from chemotherapy per RxPONDER. Endocrine therapy (often OFS+AI) should follow. ([New England Journal of Medicine][12])
KEY_REFERENCES: RxPONDER (NEJM 2021). ([New England Journal of Medicine][12])
GUIDELINE_ALIGNMENT: ASCO 2022 Biomarkers. ([ASC Publications][3])
SUBGROUP_NOTES: Fertility counseling as appropriate.
CONFIDENCE_LEVEL: High
WEB_SEARCH_USED: Yes — “RxPONDER premenopausal 1–3 nodes RS≤25” (through Feb 2025).
---END---

QUESTION_ID: 58
SELECTED_ANSWER: D
EVIDENCE_SUMMARY: With RS 23 and one positive node, premenopausal women derive chemo benefit in RxPONDER across RS≤25; thus chemo followed by endocrine therapy is recommended. ([New England Journal of Medicine][12])
KEY_REFERENCES: RxPONDER (NEJM 2021). ([New England Journal of Medicine][12])
GUIDELINE_ALIGNMENT: ASCO 2022 Biomarkers. ([ASC Publications][3])
SUBGROUP_NOTES: Aim for OFS+AI after chemotherapy when feasible. ([New England Journal of Medicine][24])
CONFIDENCE_LEVEL: High
WEB_SEARCH_USED: Yes — “RxPONDER premenopausal chemo benefit” (through Feb 2025).
---END---

QUESTION_ID: 59
SELECTED_ANSWER: D
EVIDENCE_SUMMARY: With two positive nodes (premenopausal) and RS 12, RxPONDER indicates chemotherapy benefit regardless of RS; adjuvant CDK4/6 inhibitors require specific high-risk pathologic features not met here. Thus, chemotherapy then endocrine therapy (without CDK4/6 inhibitor) is appropriate. ([New England Journal of Medicine][12], [U.S. Food and Drug Administration][25])
KEY_REFERENCES: RxPONDER (NEJM 2021); FDA abemaciclib label expansion defining “high risk” criteria. ([New England Journal of

Medicine][12], [U.S. Food and Drug Administration][25])
GUIDELINE_ALIGNMENT: ASCO 2022 Biomarkers; FDA 2023 abemaciclib label (≥4 nodes or 1–3 nodes with grade 3 or ≥5 cm). ([U.S. Food and Drug Administration][25])
SUBGROUP_NOTES: Consider OFS+AI after chemo.
CONFIDENCE_LEVEL: High
WEB_SEARCH_USED: Yes — “RxPONDER,” “abemaciclib FDA label high-risk criteria” (through Feb 2025).
\---END---

QUESTION_ID: 60
SELECTED_ANSWER: D
EVIDENCE_SUMMARY: Two positive nodes with RS 18 (premenopausal) → chemo benefit per RxPONDER; no CDK4/6 inhibitor unless high-risk criteria are met, which they are not. ([New England Journal of Medicine][12], [U.S. Food and Drug Administration][25])
KEY_REFERENCES: RxPONDER; FDA abemaciclib label. ([New England Journal of Medicine][12], [U.S. Food and Drug Administration][25])
GUIDELINE_ALIGNMENT: ASCO 2022 Biomarkers; FDA labeling for abemaciclib. ([ASC Publications][3], [U.S. Food and Drug Administration][25])
SUBGROUP_NOTES: Endocrine therapy should include OFS.
CONFIDENCE_LEVEL: High
WEB_SEARCH_USED: Yes — as above (through Feb 2025).
\---END---

QUESTION_ID: 61
SELECTED_ANSWER: D
EVIDENCE_SUMMARY: One positive node (premenopausal) with RS 23 warrants chemotherapy per RxPONDER; CDK4/6 addition is not indicated absent high-risk pathologic features. ([New England Journal of Medicine][12], [U.S. Food and Drug Administration][25])
KEY_REFERENCES: RxPONDER; FDA abemaciclib label. ([New England Journal of Medicine][12], [U.S. Food and Drug Administration][25])
GUIDELINE_ALIGNMENT: ASCO 2022 Biomarkers; FDA 2023 abemaciclib criteria. ([ASC Publications][3], [U.S. Food and Drug Administration][25])
SUBGROUP_NOTES: Discuss fertility and OFS.
CONFIDENCE_LEVEL: High
WEB_SEARCH_USED: Yes — “RxPONDER,” “abemaciclib label” (through Feb 2025).
\---END---

QUESTION_ID: 62
SELECTED_ANSWER: D
EVIDENCE_SUMMARY: One positive node with Ki-67 30% (premenopausal) benefits from chemotherapy; however, adjuvant abemaciclib per FDA (2023) requires ≥4 nodes or 1–3 nodes with grade 3 or tumor ≥5 cm—Ki-67 alone is insufficient despite being ≥20%. Therefore, choose chemotherapy then endocrine therapy without CDK4/6 inhibitor. ([U.S. Food and Drug Administration][25])
KEY_REFERENCES: FDA 2023 abemaciclib label; IKWG/ASCO notes on limited utility of Ki-67 to direct adjuvant therapy. ([U.S. Food and Drug Administration][25], [Oxford Academic][26])

GUIDELINE_ALIGNMENT: FDA labeling; ASCO/IKWG caution against using Ki-67 alone for treatment selection. ([Oxford Academic][26])
SUBGROUP_NOTES: Consider OFS+AI following chemotherapy.
CONFIDENCE_LEVEL: High
WEB_SEARCH_USED: Yes – “abemaciclib label criteria,” “Ki-67 guidance” (through Feb 2025).
\---END---

QUESTION_ID: 63
SELECTED_ANSWER: B
EVIDENCE_SUMMARY: After neoadjuvant chemotherapy with pT1N0 at surgery (three negative SLNs), adjuvant abemaciclib is not indicated because monarchE enrollment/label require pathologic node-positive or other high-risk features (≥5 cm or grade 3). Continue optimal endocrine therapy alone. ([U.S. Food and Drug Administration][25], [PubMed][27])
KEY_REFERENCES: monarchE (JCO 2020) and subsequent updates; FDA 2023 label removing Ki-67 but retaining high-risk pathologic criteria. ([PubMed][27], [U.S. Food and Drug Administration][25])
GUIDELINE_ALIGNMENT: FDA label; expert guidance applies monarchE criteria to adjuvant selection. ([ASC Publications][28])
SUBGROUP_NOTES: If future pathology showed high-risk features, reconsider CDK4/6 inhibitor; not met here.
CONFIDENCE_LEVEL: High
WEB_SEARCH_USED: Yes – “monarchE inclusion,” “FDA abemaciclib label” (through Feb 2025).
\---END---

QUESTION_ID: 64
SELECTED_ANSWER: B
EVIDENCE_SUMMARY: With 1/3 SLNs positive but small residual tumor (1 cm) and grade 2, criteria for adjuvant abemaciclib are not met (requires ≥4 nodes or 1–3 nodes plus grade 3 or primary ≥5 cm). Thus, no adjuvant CDK4/6 inhibitor is recommended beyond endocrine therapy. ([U.S. Food and Drug Administration][25])
KEY_REFERENCES: FDA 2023 abemaciclib label; monarchE trial construct. ([U.S. Food and Drug Administration][25], [PubMed][27])
GUIDELINE_ALIGNMENT: FDA label; practice reflects monarchE criteria. ([ASC Publications][28])
SUBGROUP_NOTES: Consider radiotherapy and standard endocrine therapy optimization.
CONFIDENCE_LEVEL: High
WEB_SEARCH_USED: Yes – “abemaciclib label criteria” (through Feb 2025).
\---END---

QUESTION_ID: 65
SELECTED_ANSWER: A
EVIDENCE_SUMMARY: Postmenopausal node-negative ER+/HER2- with RS 25 is within TAILORx’s 11–25 range showing no chemo benefit in women >50; endocrine therapy alone (AI) is appropriate. ([New England Journal of Medicine][7])
KEY_REFERENCES: TAILORx (NEJM 2018). ([New England Journal of Medicine][7])

GUIDELINE_ALIGNMENT: ASCO 2022 Biomarkers endorses endocrine alone for RS ≤25 in women >50. ([ASC Publications][3])
SUBGROUP_NOTES: Discuss AI duration and side-effect management.
CONFIDENCE_LEVEL: High
WEB_SEARCH_USED: Yes — “TAILORx postmenopausal RS 25 no chemo” (through Feb 2025).
\---END---

QUESTION_ID: 66
SELECTED_ANSWER: B
EVIDENCE_SUMMARY: RS 26 exceeds the TAILORx intermediate range; chemotherapy plus endocrine therapy is recommended for node-negative ER+/HER2- disease with RS ≥26. ([New England Journal of Medicine][7])
KEY_REFERENCES: TAILORx paradigm and assay cutoffs; ASCO 2022 Biomarkers. ([New England Journal of Medicine][7], [ASC Publications][3])
GUIDELINE_ALIGNMENT: ASCO 2022 and NCCN practice endorse chemo for RS ≥26. ([ASC Publications][3])
SUBGROUP_NOTES: Regimen choice individualized (e.g., TC vs AC→T). ([PubMed][19])
CONFIDENCE_LEVEL: High
WEB_SEARCH_USED: Yes — “TAILORx thresholds,” “ASCO 2022 biomarkers” (through Feb 2025).
\---END---

QUESTION_ID: 67
SELECTED_ANSWER: A
EVIDENCE_SUMMARY: A Ki-67 of 26% alone should not dictate chemotherapy; ASCO/IKWG emphasize Ki-67’s limited standalone clinical utility for adjuvant chemo decisions. Without high-risk clinical/genomic features, an AI alone is appropriate for postmenopausal node-negative 2.1 cm, grade 2 tumors pending (or alongside) genomic testing. ([Oxford Academic][26], [ASC Publications][3])
KEY_REFERENCES: IKWG/ JNCI assessment of Ki-67; ASCO 2022 Biomarkers. ([Oxford Academic][26], [ASC Publications][3])
GUIDELINE_ALIGNMENT: ASCO/ESMO advise against using Ki-67 alone to guide chemotherapy in early ER+ disease. ([Annals of Oncology][29])
SUBGROUP_NOTES: Consider genomic assay to refine risk if uncertainty remains. ([ASC Publications][3])
CONFIDENCE_LEVEL: Moderate
WEB_SEARCH_USED: Yes — “Ki-67 guidance IKWG,” “ASCO 2022 biomarkers” (through Feb 2025).
\---END---

QUESTION_ID: 68
SELECTED_ANSWER: A
EVIDENCE_SUMMARY: Postmenopausal with 1 positive node and RS 18: RxPONDER shows no chemo benefit for RS≤25; endocrine therapy alone is appropriate. CDK4/6 therapy is reserved for high-risk pathologic features not present here. ([New England Journal of Medicine][12], [U.S. Food and Drug Administration][25])
KEY_REFERENCES: RxPONDER; FDA abemaciclib label criteria. ([New

England Journal of Medicine][12], [U.S. Food and Drug Administration][25])
GUIDELINE_ALIGNMENT: ASCO 2022 Biomarkers; FDA label. ([ASC Publications][3])
SUBGROUP_NOTES: Radiation per nodal involvement and endocrine optimization.
CONFIDENCE_LEVEL: High
WEB_SEARCH_USED: Yes – "RxPONDER postmenopausal N1 RS 18," "abemaciclib criteria" (through Feb 2025).
---END---

QUESTION_ID: 69
SELECTED_ANSWER: A
EVIDENCE_SUMMARY: With one positive node and Ki-67 18% (postmenopausal), Ki-67 alone should not drive chemotherapy; absent genomic high risk or high-risk pathologic criteria, endocrine therapy alone is appropriate. ([Oxford Academic][26])
KEY_REFERENCES: IKWG/ASCO guidance on Ki-67; ASCO 2022 Biomarkers. ([Oxford Academic][26], [ASC Publications][3])
GUIDELINE_ALIGNMENT: ASCO/ESMO recommend against basing chemo solely on Ki-67. ([Annals of Oncology][29])
SUBGROUP_NOTES: If genomic assay returns high risk, recommendations would change.
CONFIDENCE_LEVEL: Moderate
WEB_SEARCH_USED: Yes – "Ki-67 IKWG guidance," "ASCO biomarkers" (through Feb 2025).
---END---

QUESTION_ID: 70
SELECTED_ANSWER: A
EVIDENCE_SUMMARY: Postmenopausal with 2 positive nodes and RS 11: RxPONDER indicates no chemo benefit for RS≤25 in postmenopausal N1 disease; endocrine therapy alone is recommended. ([New England Journal of Medicine][12])
KEY_REFERENCES: RxPONDER (NEJM 2021). ([New England Journal of Medicine][12])
GUIDELINE_ALIGNMENT: ASCO 2022 Biomarkers. ([ASC Publications][3])
SUBGROUP_NOTES: Consider extended ET duration given nodal involvement. ([PMC][16])
CONFIDENCE_LEVEL: High
WEB_SEARCH_USED: Yes – "RxPONDER postmenopausal 1–3 nodes RS≤25" (through Feb 2025).
---END---

QUESTION_ID: 71
SELECTED_ANSWER: A
EVIDENCE_SUMMARY: With two positive nodes and Ki-67 11% (postmenopausal), absent high-risk pathologic/genomic features, endocrine therapy alone is appropriate; Ki-67 should not be used alone to recommend chemotherapy. ([Oxford Academic][26])
KEY_REFERENCES: IKWG/ASCO Ki-67 guidance; ASCO 2022 Biomarkers. ([Oxford Academic][26], [ASC Publications][3])
GUIDELINE_ALIGNMENT: ASCO/ESMO guidance. ([Annals of Oncology][29])
SUBGROUP_NOTES: Extended ET often recommended with nodal disease.

([PMC][16])
CONFIDENCE_LEVEL: Moderate
WEB_SEARCH_USED: Yes – “Ki-67 guidance,” “extended ET node-positive” (through Feb 2025).
\---END---

QUESTION_ID: 72
SELECTED_ANSWER: A
EVIDENCE_SUMMARY: Adjuvant CDK4/6 inhibitor use (abemaciclib; ribociclib in NATALEE) has been studied in adults inclusive of men, and management principles for early ER+ male breast cancer largely mirror those for women. Thus, selection criteria for adjuvant CDK4/6 therapy are applied similarly, subject to the labeled/eligible high-risk features. ([PubMed][30], [PMC][31], [ASC Publications][32])
KEY_REFERENCES: monarchE (JCO 2020) and updates; NATALEE (NEJM 2024); ASCO Male Breast Cancer Guideline (2020). ([PubMed][27], [ASC Publications][32])
GUIDELINE_ALIGNMENT: ASCO male breast cancer guidance notes treatment parallels; trial eligibility included men. ([ASC Publications][32], [PMC][31])
SUBGROUP_NOTES: In men, ensure endocrine backbone (AI requires GnRH agonist) and apply the same high-risk criteria (e.g., monarchE). ([PubMed][27])
CONFIDENCE_LEVEL: Moderate
WEB_SEARCH_USED: Yes – “monarchE inclusion men,” “NATALEE inclusion men,” “ASCO male breast guideline” (through Feb 2025).
\---END---

[1]: https://www.nice.org.uk/guidance/dg58/resources/dg34-tumour-profiling-tests-to-guide-adjuvant-chemotherapy-decisions-in-early-breast-cancer-evidence-review-pdf-17397331472581?utm_source=chatgpt.com "DG34 Tumour profiling tests to guide adjuvant ..."
[2]: https://breast.predict.cam/tool?utm_source=chatgpt.com "Tool - Predict Breast"
[3]: https://ascopubs.org/doi/10.1200/JCO.22.00069?utm_source=chatgpt.com "Biomarkers for Adjuvant Endocrine and Chemotherapy in ..."
[4]: https://breast.v3.predict.cam/about/overview/howpredictworks?utm_source=chatgpt.com "Overview - Predict Breast"
[5]: https://pmc.ncbi.nlm.nih.gov/articles/PMC10789872/?utm_source=chatgpt.com "An updated PREDICT breast cancer prognostic model ..."
[6]: https://www.mdpi.com/1718-7729/30/1/15?utm_source=chatgpt.com "Radiation in Early-Stage Breast Cancer: Moving beyond an ..."
[7]: https://www.nejm.org/doi/full/10.1056/NEJMoa1804710?utm_source=chatgpt.com "Adjuvant Chemotherapy Guided by a 21-Gene Expression ..."
[8]: https://www.nejm.org/doi/full/10.1056/NEJMoa1904819?utm_source=chatgpt.com "Clinical and Genomic Risk to Guide the Use of Adjuvant ..."
[9]: https://pubmed.ncbi.nlm.nih.gov/21555689/?utm_source=chatgpt.com "Randomized phase II neoadjuvant comparison between ..."

[10]: https://pubmed.ncbi.nlm.nih.gov/38101773/?utm_source=chatgpt.com "Early breast cancer: ESMO Clinical Practice Guideline for ..."
[11]: https://pubmed.ncbi.nlm.nih.gov/36653664/?utm_source=chatgpt.com "Data from ACOSOG Z1031 (Alliance), a Randomized ..."
[12]: https://www.nejm.org/doi/full/10.1056/NEJMoa2108873?utm_source=chatgpt.com "21-Gene Assay to Inform Chemotherapy Benefit in Node- ..."
[13]: https://www.swog.org/news-events/news/2021/12/03/rxponder-results-published-nejm?utm_source=chatgpt.com "RxPONDER Results Published in NEJM"
[14]: https://pmc.ncbi.nlm.nih.gov/articles/PMC10453164/?utm_source=chatgpt.com "Extended Adjuvant Endocrine Therapy in Early Breast ..."
[15]: https://www.nejm.org/doi/full/10.1056/NEJMoa1604700?utm_source=chatgpt.com "Extending Aromatase-Inhibitor Adjuvant Therapy to 10 Years"
[16]: https://pmc.ncbi.nlm.nih.gov/articles/PMC10637036/?utm_source=chatgpt.com "Ten-year update: NRG Oncology/National Surgical ..."
[17]: https://www.thelancet.com/journals/lancet/article/PIIS0140-6736%2812%2961963-1/fulltext?utm_source=chatgpt.com "Long-term effects of continuing adjuvant tamoxifen to 10 ..."
[18]: https://ascopubs.org/doi/10.1200/JCO.18.01160?utm_source=chatgpt.com "Adjuvant Endocrine Therapy for Women With Hormone ..."
[19]: https://pubmed.ncbi.nlm.nih.gov/19204201/?utm_source=chatgpt.com "7-Year Follow-Up of US Oncology Research Trial 9735"
[20]: https://www.esmoopen.com/article/S2059-7029%2822%2900079-5/pdf?utm_source=chatgpt.com "Anthracyclines in the treatment of patients with early breast ..."
[21]: https://pubmed.ncbi.nlm.nih.gov/22152853/?utm_source=chatgpt.com "meta-analyses of long-term outcome among 100000 ..."
[22]: https://www.thelancet.com/journals/lancet/article/PIIS0140-6736%2818%2933137-4/fulltext?utm_source=chatgpt.com "a patient-level meta-analysis of 37 298 women with early ..."
[23]: https://pubmed.ncbi.nlm.nih.gov/36521078/?utm_source=chatgpt.com "Long-Term Follow-Up of the Combined TEXT and SOFT ..."
[24]: https://www.nejm.org/doi/full/10.1056/NEJMoa1803164?utm_source=chatgpt.com "Tailoring Adjuvant Endocrine Therapy for Premenopausal ..."
[25]: https://www.fda.gov/drugs/resources-information-approved-drugs/fda-expands-early-breast-cancer-indication-abemaciclib-endocrine-therapy?utm_source=chatgpt.com "FDA expands early breast cancer indication for ..."
[26]: https://academic.oup.com/jnci/article/113/7/808/6053794?utm_source=chatgpt.com "Assessment of Ki67 in Breast Cancer: Updated ..."
[27]: https://pubmed.ncbi.nlm.nih.gov/32954927/?utm_source=chatgpt.com "Abemaciclib Combined With Endocrine Therapy

for the ..."
[28]: https://ascopubs.org/doi/pdf/10.1200/JCO.24.00886?utm_source=chatgpt.com "Optimal Adjuvant Chemotherapy and Targeted Therapy for ..."
[29]: https://www.annalsofoncology.org/article/S0923-7534%2823%2905104-9/pdf?utm_source=chatgpt.com "Early breast cancer: ESMO Clinical Practice Guideline for ..."
[30]: https://pubmed.ncbi.nlm.nih.gov/38507751/?utm_source=chatgpt.com "Ribociclib plus Endocrine Therapy in Early Breast Cancer"
[31]: https://pmc.ncbi.nlm.nih.gov/articles/PMC10233570/?utm_source=chatgpt.com "a Phase III trial of adjuvant ribociclib + endocrine therapy ..."
[32]: https://ascopubs.org/doi/10.1200/JCO.19.03120?utm_source=chatgpt.com "Management of Male Breast Cancer: ASCO Guideline"
