# Supplementary material for: Performance of large language models in ambiguous treatment scenarios of early breast cancer
Source: Breast. 2026 Sep 23;90:104932. doi: 10.1016/j.breast.2026.104932 (PMC13641045; doi:10.1016/j.breast.2026.104932)
Supplement: Supplementary file 1 [file mmc1.zip › Suppl.Mat. - Anton Oseledchyk - Prompts, LLM Responses, R-Code/hrpos_response_claude.pdf]

QUESTION_ID: 39
SELECTED_ANSWER: C
EVIDENCE_SUMMARY: The observed 37% reduction in risk of recurrence and 34% reduction in mortality risk at 10 years, associated with a 15.4% absolute benefit in disease-free state and 10.1% in survival, reaffirm the role of adjuvant chemohormonal therapy. Generally, a 3% or greater absolute benefit is considered clinically meaningful for recommending adjuvant chemotherapy in ER-positive breast cancer.
KEY_REFERENCES: EBCTCG meta-analyses, TAILORx trial, RxPONDER trial
GUIDELINE_ALIGNMENT: ASCO, NCCN guidelines support 3% threshold for clinical benefit
SUBGROUP_NOTES: Lower thresholds may be considered for younger patients or those with high-risk features
CONFIDENCE_LEVEL: High
WEB_SEARCH_USED: Yes
---END---

QUESTION_ID: 40
SELECTED_ANSWER: B
EVIDENCE_SUMMARY: While tumors ≤1 cm generally have favorable prognosis, The 21-gene breast cancer assay (OncotypeDX) yields a Recurrence Score result that predicts the 10-yr risk of distant recurrence (DR) and the likelihood of chemotherapy (CT) benefit in patients (pts) with ER+ EBC. Some small ER-positive tumors can have high recurrence scores indicating higher risk and potential chemotherapy benefit.
KEY_REFERENCES: NSABP B-21, TAILORx subanalyses of small tumors
GUIDELINE_ALIGNMENT: NCCN and ASCO recommend genomic testing even for small node-negative ER+ tumors
SUBGROUP_NOTES: Particularly important for grade 2-3 tumors, younger patients, or those with other high-risk features
CONFIDENCE_LEVEL: High
WEB_SEARCH_USED: Yes
---END---

QUESTION_ID: 42
SELECTED_ANSWER: B
EVIDENCE_SUMMARY: neoadjuvant endocrine therapy (NET) facilitates down-staging of the tumor and increased rates of breast-conserving surgery in ER-positive breast cancer. For clinical T3N1 grade 1 strongly ER/PR positive lobular histology, neoadjuvant endocrine therapy is appropriate as these features predict good endocrine sensitivity.
KEY_REFERENCES: ASCO Neoadjuvant Guidelines 2021, multiple NET trials
GUIDELINE_ALIGNMENT: ASCO and NCCN support NET for strongly ER+ tumors requiring downstaging
SUBGROUP_NOTES: Lobular histology and grade 1 features predict excellent endocrine response
CONFIDENCE_LEVEL: High
WEB_SEARCH_USED: Yes
---END---

QUESTION_ID: 43

SELECTED_ANSWER: B
EVIDENCE_SUMMARY: With confirmed low-risk genomic score in strongly ER/PR positive grade 1 tumor, neoadjuvant endocrine therapy alone is appropriate. NET should not only be used to allow surgery or to improve breast-conserving surgery rates in patients not eligible for NCT, and low genomic score confirms minimal chemotherapy benefit.
KEY_REFERENCES: ASCO Neoadjuvant Guidelines, NET trials with genomic scoring
GUIDELINE_ALIGNMENT: ASCO supports NET alone for low genomic risk ER+ tumors
SUBGROUP_NOTES: CDK4/6 inhibitors not established in neoadjuvant setting outside trials
CONFIDENCE_LEVEL: High
WEB_SEARCH_USED: Yes
---END---

QUESTION_ID: 44
SELECTED_ANSWER: A
EVIDENCE_SUMMARY: Despite low genomic score, residual disease in breast and 3 of 11 nodes after neoadjuvant endocrine therapy indicates endocrine resistance and higher risk. Adjuvant chemotherapy should be considered given the significant residual disease burden indicating inadequate response to endocrine therapy alone.
KEY_REFERENCES: CREATE-X concept, neoadjuvant therapy response-guided trials
GUIDELINE_ALIGNMENT: NCCN recommends considering chemotherapy for significant residual disease after NET
SUBGROUP_NOTES: Residual nodal disease particularly warrants additional systemic therapy
CONFIDENCE_LEVEL: Moderate
WEB_SEARCH_USED: Yes
---END---

QUESTION_ID: 45
SELECTED_ANSWER: B
EVIDENCE_SUMMARY: Women with node-positive breast cancer should be offered extended AI-based therapy for up to a total of 10 years of adjuvant endocrine treatment. For 1 positive node with 1.8 cm grade 2 tumor, 7-8 years represents appropriate duration balancing benefit and toxicity.
KEY_REFERENCES: ASCO Extended Endocrine Therapy Guidelines, MA.17, NSABP B-42
GUIDELINE_ALIGNMENT: ASCO recommends 7-10 years for node-positive disease
SUBGROUP_NOTES: Single positive node allows consideration of shorter duration within extended therapy
CONFIDENCE_LEVEL: High
WEB_SEARCH_USED: Yes
---END---

QUESTION_ID: 46
SELECTED_ANSWER: B
EVIDENCE_SUMMARY: In TAILORx, women aged 50 or younger with node-

negative disease and a recurrence score between 16 and 25 had a decrease in distant recurrence with adjuvant chemotherapy added to endocrine therapy. High recurrence score (28) indicates higher risk warranting extended endocrine therapy of 7-8 years.
KEY_REFERENCES: TAILORx, ASCO Extended Therapy Guidelines
GUIDELINE_ALIGNMENT: ASCO supports extended therapy for high-risk node-negative disease
SUBGROUP_NOTES: Duration may depend on age and tolerance
CONFIDENCE_LEVEL: High
WEB_SEARCH_USED: Yes
---END---

QUESTION_ID: 47
SELECTED_ANSWER: C
EVIDENCE_SUMMARY: Extended therapy in the setting of higher-risk node-positive breast cancers provides a substantial reduction in risk of recurrence that, in the opinion of the Expert Panel, warrants the ongoing AEs and risks of treatment. With 3 of 8 positive nodes, 10 years of endocrine therapy is recommended.
KEY_REFERENCES: ASCO Extended Therapy Guidelines, MA.17, EBCTCG meta-analyses
GUIDELINE_ALIGNMENT: ASCO strongly recommends 10 years for multiple positive nodes
SUBGROUP_NOTES: Higher nodal burden clearly benefits from maximum duration
CONFIDENCE_LEVEL: High
WEB_SEARCH_USED: Yes
---END---

QUESTION_ID: 48
SELECTED_ANSWER: B
EVIDENCE_SUMMARY: TC was considered a standard regimen for adjuvant therapy for node-negative or low-risk node-positive breast cancer. For postmenopausal women with T2N0 and OncotypeDX RS 28, TC provides adequate efficacy with less toxicity than AC/T.
KEY_REFERENCES: US Oncology 9735, ABC trials, network meta-analyses
GUIDELINE_ALIGNMENT: NCCN includes TC as option for node-negative disease
SUBGROUP_NOTES: Postmenopausal status and absence of nodal involvement support TC
CONFIDENCE_LEVEL: High
WEB_SEARCH_USED: Yes
---END---

QUESTION_ID: 49
SELECTED_ANSWER: A
EVIDENCE_SUMMARY: With OncotypeDX RS 32 (high risk), Sequential AC-T yielded longer OS than AC, CMF, or no adjuvant chemotherapy. The higher recurrence score warrants more intensive anthracycline-taxane combination for optimal outcomes.
KEY_REFERENCES: TAILORx high-risk subgroup, EBCTCG meta-analyses
GUIDELINE_ALIGNMENT: NCCN recommends AC/T for high recurrence scores
SUBGROUP_NOTES: RS ≥31 traditionally indicates high risk benefiting from anthracyclines

CONFIDENCE_LEVEL: High
WEB_SEARCH_USED: Yes
---END---

QUESTION_ID: 50
SELECTED_ANSWER: A
EVIDENCE_SUMMARY: Grade 3 tumor with OncotypeDX RS 32 represents high-risk disease where Sequential AC-T is likely to be the most effective adjuvant therapy regimen for early-stage breast cancer. The combination of high grade and high RS strongly supports anthracycline-based therapy.
KEY_REFERENCES: TAILORx, EBCTCG meta-analyses, network meta-analyses
GUIDELINE_ALIGNMENT: NCCN and ASCO recommend AC/T for high-risk features
SUBGROUP_NOTES: Grade 3 with high RS particularly benefits from intensive chemotherapy
CONFIDENCE_LEVEL: High
WEB_SEARCH_USED: Yes
---END---

QUESTION_ID: 51
SELECTED_ANSWER: C
EVIDENCE_SUMMARY: Among premenopausal women with one to three positive lymph nodes and a recurrence score of 25 or lower, those who received chemoendocrine therapy had longer invasive disease-free survival. With 3 positive nodes, AC/T chemotherapy provides optimal benefit despite low RS.
KEY_REFERENCES: RxPONDER trial, SWOG S8814
GUIDELINE_ALIGNMENT: NCCN recommends chemotherapy for multiple positive nodes
SUBGROUP_NOTES: Nodal burden of 3 nodes warrants chemotherapy regardless of RS
CONFIDENCE_LEVEL: High
WEB_SEARCH_USED: Yes
---END---

QUESTION_ID: 52
SELECTED_ANSWER: C
EVIDENCE_SUMMARY: With 4 of 11 positive nodes (N2 disease), the chemotherapy benefit was dependent on the recurrence-score value but nodal burden this high warrants AC/T chemotherapy for optimal disease control regardless of low RS.
KEY_REFERENCES: RxPONDER, SWOG S8814
GUIDELINE_ALIGNMENT: NCCN strongly recommends chemotherapy for N2 disease
SUBGROUP_NOTES: Four or more positive nodes represents high-risk disease requiring intensive therapy
CONFIDENCE_LEVEL: High
WEB_SEARCH_USED: Yes
---END---

QUESTION_ID: 53
SELECTED_ANSWER: B
EVIDENCE_SUMMARY: For premenopausal women with low-risk disease

(T1cN0, grade 2, RS 12), OFS + tamoxifen provides superior disease control compared to tamoxifen alone while avoiding chemotherapy toxicity given the low recurrence score.
KEY_REFERENCES: SOFT/TEXT trials, TAILORx
GUIDELINE_ALIGNMENT: ASCO and ESMO support OFS for higher-risk premenopausal patients
SUBGROUP_NOTES: Age 42 with intermediate-risk features benefits from OFS
CONFIDENCE_LEVEL: High
WEB_SEARCH_USED: Yes
---END---

QUESTION_ID: 54
SELECTED_ANSWER: B
EVIDENCE_SUMMARY: women aged 50 or younger with node-negative disease and a recurrence score between 16 and 25 had a decrease in distant recurrence with adjuvant chemotherapy. With RS 18 in a 42-year-old, OFS + tamoxifen provides good disease control.
KEY_REFERENCES: TAILORx age-based analyses, SOFT/TEXT
GUIDELINE_ALIGNMENT: ASCO supports OFS for premenopausal intermediate-risk disease
SUBGROUP_NOTES: RS 18 in age 42 suggests benefit from endocrine intensification without chemotherapy
CONFIDENCE_LEVEL: High
WEB_SEARCH_USED: Yes
---END---

QUESTION_ID: 55
SELECTED_ANSWER: D
EVIDENCE_SUMMARY: women aged 50 or younger with node-negative disease and a recurrence score between 16 and 25 had a decrease in distant recurrence with adjuvant chemotherapy added to endocrine therapy. RS 23 in a 42-year-old warrants chemotherapy followed by endocrine therapy.
KEY_REFERENCES: TAILORx trial
GUIDELINE_ALIGNMENT: NCCN recommends chemotherapy for RS 21-25 in women ≤50
SUBGROUP_NOTES: Age ≤50 with RS >20 clearly benefits from chemotherapy
CONFIDENCE_LEVEL: High
WEB_SEARCH_USED: Yes
---END---

QUESTION_ID: 56
SELECTED_ANSWER: C
EVIDENCE_SUMMARY: premenopausal women with one to three positive lymph nodes and a recurrence score of 25 or lower, those who received chemoendocrine therapy had longer invasive disease-free survival. With one node and RS 12, OFS + AI provides optimal endocrine therapy.
KEY_REFERENCES: RxPONDER, SOFT/TEXT trials
GUIDELINE_ALIGNMENT: ASCO supports OFS + AI for node-positive premenopausal patients
SUBGROUP_NOTES: Single node with low RS allows endocrine therapy

optimization without chemotherapy
CONFIDENCE_LEVEL: High
WEB_SEARCH_USED: Yes
---END---

QUESTION_ID: 57
SELECTED_ANSWER: D
EVIDENCE_SUMMARY: premenopausal women with one to three positive lymph nodes and a recurrence score of 25 or lower, those who received chemoendocrine therapy had longer invasive disease-free survival. Node-positive disease in premenopausal women benefits from chemotherapy regardless of RS.
KEY_REFERENCES: RxPONDER trial
GUIDELINE_ALIGNMENT: NCCN recommends chemotherapy for premenopausal node-positive disease
SUBGROUP_NOTES: Premenopausal status with nodal involvement indicates chemotherapy benefit
CONFIDENCE_LEVEL: High
WEB_SEARCH_USED: Yes
---END---

QUESTION_ID: 58
SELECTED_ANSWER: D
EVIDENCE_SUMMARY: premenopausal women with one to three positive lymph nodes and a recurrence score of 25 or lower, those who received chemoendocrine therapy had longer invasive disease-free survival. RS 23 with node-positive disease in premenopausal patient clearly warrants chemotherapy.
KEY_REFERENCES: RxPONDER trial
GUIDELINE_ALIGNMENT: NCCN strongly recommends chemotherapy for this profile
SUBGROUP_NOTES: Combination of nodal disease and RS approaching 25 indicates clear chemotherapy benefit
CONFIDENCE_LEVEL: High
WEB_SEARCH_USED: Yes
---END---

QUESTION_ID: 59
SELECTED_ANSWER: D
EVIDENCE_SUMMARY: With two positive nodes in a premenopausal patient, premenopausal women with one to three positive lymph nodes and a recurrence score of 25 or lower, those who received chemoendocrine therapy had longer invasive disease-free survival. Chemotherapy followed by endocrine therapy is indicated despite low RS.
KEY_REFERENCES: RxPONDER trial, monarchE eligibility criteria
GUIDELINE_ALIGNMENT: NCCN recommends chemotherapy for premenopausal patients with multiple nodes
SUBGROUP_NOTES: Two nodes insufficient for CDK4/6 inhibitor criteria in most trials
CONFIDENCE_LEVEL: High
WEB_SEARCH_USED: Yes
---END---

QUESTION_ID: 60
SELECTED_ANSWER: D
EVIDENCE_SUMMARY: Premenopausal woman with two positive nodes benefits from chemotherapy based on RxPONDER regardless of RS. CDK4/6 inhibitors require higher risk features (≥4 nodes or other high-risk criteria per monarchE).
KEY_REFERENCES: RxPONDER, monarchE trial criteria
GUIDELINE_ALIGNMENT: NCCN recommends chemotherapy; CDK4/6 inhibitors for higher risk only
SUBGROUP_NOTES: Two nodes alone insufficient for adjuvant CDK4/6 inhibitor indication
CONFIDENCE_LEVEL: High
WEB_SEARCH_USED: Yes
---END---

QUESTION_ID: 61
SELECTED_ANSWER: D
EVIDENCE_SUMMARY: Premenopausal patient with one positive node and RS 23 requires chemotherapy based on RxPONDER trial showing benefit regardless of RS in premenopausal node-positive disease, followed by endocrine therapy.
KEY_REFERENCES: RxPONDER trial
GUIDELINE_ALIGNMENT: NCCN recommends chemotherapy for premenopausal node-positive disease
SUBGROUP_NOTES: Single node insufficient for CDK4/6 inhibitor consideration
CONFIDENCE_LEVEL: High
WEB_SEARCH_USED: Yes
---END---

QUESTION_ID: 62
SELECTED_ANSWER: D
EVIDENCE_SUMMARY: Premenopausal woman with node-positive disease and Ki67 30% (high proliferation) warrants chemotherapy followed by endocrine therapy. High Ki67 indicates aggressive biology requiring chemotherapy.
KEY_REFERENCES: St. Gallen consensus (through 2023), RxPONDER
GUIDELINE_ALIGNMENT: International guidelines recommend chemotherapy for high Ki67 with nodal disease
SUBGROUP_NOTES: Ki67 ≥30% considered high risk requiring chemotherapy
CONFIDENCE_LEVEL: High
WEB_SEARCH_USED: Yes
---END---

QUESTION_ID: 63
SELECTED_ANSWER: B
EVIDENCE_SUMMARY: With complete nodal response (3 negative sentinel nodes) after neoadjuvant chemotherapy for cT2N1 disease, the patient achieved good response. monarchE clinical trial included patients with stage II and III hormone receptor-positive, HER2-negative disease at especially high risk which this patient no longer meets.
KEY_REFERENCES: monarchE trial criteria
GUIDELINE_ALIGNMENT: ASCO CDK4/6 inhibitor guidelines require high-

risk residual disease
SUBGROUP_NOTES: Nodal pCR indicates good prognosis not requiring CDK4/6 inhibitor
CONFIDENCE_LEVEL: High
WEB_SEARCH_USED: Yes
---END---

QUESTION_ID: 64
SELECTED_ANSWER: A
EVIDENCE_SUMMARY: With residual nodal disease (1 of 3 positive) after neoadjuvant chemotherapy, patient has high-risk features. monarchE eligibility required at least one of grade 3 or Ki-67 index ≥20%, or tumor size ≥5cm with residual node-positive disease warranting CDK4/6 inhibitor.
KEY_REFERENCES: monarchE trial
GUIDELINE_ALIGNMENT: ASCO recommends CDK4/6 inhibitors for residual node-positive disease after neoadjuvant chemotherapy
SUBGROUP_NOTES: Residual nodal disease indicates high risk benefiting from CDK4/6 inhibitor
CONFIDENCE_LEVEL: High
WEB_SEARCH_USED: Yes
---END---

QUESTION_ID: 65
SELECTED_ANSWER: B
EVIDENCE_SUMMARY: women aged 50 or younger with node-negative disease and a recurrence score between 16 and 25 had a decrease in distant recurrence with adjuvant chemotherapy. Postmenopausal women with RS 25 benefit from chemotherapy followed by AI based on TAILORx.
KEY_REFERENCES: TAILORx trial
GUIDELINE_ALIGNMENT: NCCN recommends chemotherapy for RS ≥26 regardless of age
SUBGROUP_NOTES: RS 25 is borderline but generally warrants chemotherapy in node-negative disease
CONFIDENCE_LEVEL: High
WEB_SEARCH_USED: Yes
---END---

QUESTION_ID: 66
SELECTED_ANSWER: B
EVIDENCE_SUMMARY: OncotypeDX RS 26 indicates high risk where chemotherapy benefit is established. TAILORx showed clear benefit for RS >25, warranting chemotherapy followed by AI in postmenopausal women.
KEY_REFERENCES: TAILORx trial
GUIDELINE_ALIGNMENT: NCCN strongly recommends chemotherapy for RS ≥26
SUBGROUP_NOTES: Clear indication for chemotherapy based on genomic risk
CONFIDENCE_LEVEL: High
WEB_SEARCH_USED: Yes
---END---

QUESTION_ID: 67
SELECTED_ANSWER: B
EVIDENCE_SUMMARY: Ki67 of 26% indicates intermediate-high proliferation in node-negative disease. Given tumor size 2.1 cm and grade 2, chemotherapy followed by AI is appropriate for this higher-risk profile.
KEY_REFERENCES: International consensus guidelines, St. Gallen (through 2023)
GUIDELINE_ALIGNMENT: Guidelines support chemotherapy for Ki67 >20-30% with other risk factors
SUBGROUP_NOTES: Ki67 alone less predictive than genomic assays but 26% suggests higher risk
CONFIDENCE_LEVEL: Moderate
WEB_SEARCH_USED: Yes
---END---

QUESTION_ID: 68
SELECTED_ANSWER: A
EVIDENCE_SUMMARY: postmenopausal women showed no significant difference in invasive disease-free survival with the addition of chemotherapy for RS ≤25. With RS 18 and single positive node in postmenopausal woman, AI alone is appropriate.
KEY_REFERENCES: RxPONDER trial
GUIDELINE_ALIGNMENT: NCCN supports omitting chemotherapy for postmenopausal RS ≤25 with 1-3 nodes
SUBGROUP_NOTES: Postmenopausal status key factor allowing chemotherapy omission
CONFIDENCE_LEVEL: High
WEB_SEARCH_USED: Yes
---END---

QUESTION_ID: 69
SELECTED_ANSWER: A
EVIDENCE_SUMMARY: Postmenopausal patient with single positive node and Ki67 18% (intermediate) can be treated with AI alone. RxPONDER showed no chemotherapy benefit for postmenopausal women with limited nodal disease.
KEY_REFERENCES: RxPONDER trial
GUIDELINE_ALIGNMENT: NCCN supports AI alone for postmenopausal limited nodal disease with favorable features
SUBGROUP_NOTES: Ki67 <20% with single node represents lower risk
CONFIDENCE_LEVEL: High
WEB_SEARCH_USED: Yes
---END---

QUESTION_ID: 70
SELECTED_ANSWER: C
EVIDENCE_SUMMARY: With 2 of 4 positive nodes and low RS 11, postmenopausal patient still qualifies for AI + CDK4/6 inhibitor. NATALEE included a much broader set of patients including those with 2-3 positive nodes regardless of RS.
KEY_REFERENCES: RxPONDER, NATALEE trial criteria
GUIDELINE_ALIGNMENT: ASCO allows CDK4/6 inhibitors for 2+ nodes meeting trial criteria

SUBGROUP_NOTES: Multiple nodes represent higher risk despite low RS
CONFIDENCE_LEVEL: Moderate
WEB_SEARCH_USED: Yes
---END---

QUESTION_ID: 71
SELECTED_ANSWER: C
EVIDENCE_SUMMARY: Two positive nodes with Ki67 11% (low) in postmenopausal patient warrants consideration of CDK4/6 inhibitor. NATALEE correlate to an intermediate/high-risk population including those with multiple positive nodes.
KEY_REFERENCES: NATALEE trial criteria
GUIDELINE_ALIGNMENT: ASCO CDK4/6 guidelines include patients with 2+ nodes
SUBGROUP_NOTES: Low Ki67 but multiple nodes justify intensified therapy
CONFIDENCE_LEVEL: Moderate
WEB_SEARCH_USED: Yes
---END---

QUESTION_ID: 72
SELECTED_ANSWER: B
EVIDENCE_SUMMARY: Limited data exists for CDK4/6 inhibitors in men with breast cancer as they were excluded from pivotal trials. NATALEE trial evaluated the addition of 3 years of ribociclib to standard endocrine therapy versus endocrine therapy alone in women and men but male enrollment was minimal, making evidence insufficient for routine use.
KEY_REFERENCES: monarchE and NATALEE trials (limited male patients)
GUIDELINE_ALIGNMENT: No specific guidelines endorse routine CDK4/6 use in males
SUBGROUP_NOTES: Case-by-case consideration may be appropriate for high-risk male patients
CONFIDENCE_LEVEL: High
WEB_SEARCH_USED: Yes
---END---
