# Supplementary material for: Performance of large language models in ambiguous treatment scenarios of early breast cancer
Source: Breast. 2026 Sep 23;90:104932. doi: 10.1016/j.breast.2026.104932 (PMC13641045; doi:10.1016/j.breast.2026.104932)
Supplement: Supplementary file 1 [file mmc1.zip › Suppl.Mat. - Anton Oseledchyk - Prompts, LLM Responses, R-Code/hrpos_response_deepseek.pdf]

QUESTION_ID: 39
SELECTED_ANSWER: C
EVIDENCE_SUMMARY: For ER+ breast cancer, chemotherapy is generally recommended when the absolute improvement in distant recurrence-free survival is at least 3%, based on TAILORx and RxPONDER trials which established this threshold for intermediate-risk patients. Recent NCCN Guidelines (v3.2025) continue to support this 3% threshold as the point where chemotherapy benefits outweigh risks for most patients.
KEY_REFERENCES: TAILORx trial; RxPONDER trial; NCCN Guidelines Breast Cancer v3.2025
GUIDELINE_ALIGNMENT: NCCN (2025) and ASCO guidelines endorse using a 3% or greater absolute improvement in distant recurrence to guide chemotherapy decisions in ER+ breast cancer.
SUBGROUP_NOTES: Younger patients may derive greater absolute benefit from chemotherapy, potentially lowering the threshold for consideration in premenopausal women.
CONFIDENCE_LEVEL: High
WEB_SEARCH_USED: Yes - Confirmed NCCN v3.2025 maintains the 3% threshold for chemotherapy benefit in ER+ breast cancer.

---END---

QUESTION_ID: 40
SELECTED_ANSWER: B
EVIDENCE_SUMMARY: Genomic risk assays remain valuable even for small, node-negative ER+ tumors, as they can identify patients with higher recurrence risk who might benefit from chemotherapy. NSABP B-21 showed low but non-zero distant recurrence risk, and subsequent trials (TAILORx) demonstrated that genomic testing can refine risk stratification beyond clinicopathologic factors alone.
KEY_REFERENCES: NSABP B-21; TAILORx trial; NCCN Guidelines Breast Cancer v3.2025
GUIDELINE_ALIGNMENT: NCCN (2025) guidelines recommend considering genomic testing for T1b tumors and larger, regardless of perceived low clinical risk.
SUBGROUP_NOTES: Very low-risk tumors (e.g., <0.5 cm) may not require testing, but 1 cm tumors should be evaluated based on individual risk factors.
CONFIDENCE_LEVEL: High
WEB_SEARCH_USED: No - Based on established trial data; no new evidence through Feb 2025 changes this recommendation.

---END---

QUESTION_ID: 42
SELECTED_ANSWER: B
EVIDENCE_SUMMARY: For this strongly ER+/PR+, grade 1 T3N1 tumor, neoadjuvant endocrine therapy is preferred over chemotherapy due to the high likelihood of endocrine sensitivity and lower chemotherapy benefit in low-grade luminal tumors. Recent NCCN Guidelines (v3.2025) support neoadjuvant endocrine therapy for strongly hormone receptor-positive, low-grade tumors regardless of size.
KEY_REFERENCES: ALTERNATE trial; NCCN Guidelines Breast Cancer

v3.2025
GUIDELINE_ALIGNMENT: NCCN (2025) and ASCO guidelines recommend neoadjuvant endocrine therapy for strongly ER+ tumors, particularly in postmenopausal women.
SUBGROUP_NOTES: Lobular histology may respond slower to endocrine therapy but is generally chemotherapy-resistant. Genomic testing could be considered but should not delay treatment initiation.
CONFIDENCE_LEVEL: High
WEB_SEARCH_USED: Yes - Verified NCCN v3.2025 continues to recommend neoadjuvant endocrine therapy for strongly ER+ tumors.

---END---

QUESTION_ID: 43
SELECTED_ANSWER: B
EVIDENCE_SUMMARY: With the addition of a low-risk genomic score, neoadjuvant endocrine therapy is strongly favored over chemotherapy for this strongly ER+/PR+, grade 1 T3N1 tumor. The low genomic risk confirms the low biological aggressiveness and minimal expected benefit from chemotherapy.
KEY_REFERENCES: ALTERNATE trial; TAILORx trial; NCCN Guidelines Breast Cancer v3.2025
GUIDELINE_ALIGNMENT: NCCN (2025) guidelines support neoadjuvant endocrine therapy for strongly ER+ tumors with low genomic risk.
SUBGROUP_NOTES: The combination of clinical, pathological, and genomic low risk makes chemotherapy very unlikely to provide meaningful benefit.
CONFIDENCE_LEVEL: High
WEB_SEARCH_USED: No - Based on established knowledge; no new evidence through Feb 2025 changes this approach.

---END---

QUESTION_ID: 44
SELECTED_ANSWER: A
EVIDENCE_SUMMARY: Despite the initial low-risk features, the presence of residual disease in both breast and multiple lymph nodes (3/11) after neoadjuvant endocrine therapy indicates endocrine resistance and higher recurrence risk. Adjuvant chemotherapy is recommended based on the CREATE-X trial principle, though specific data for endocrine therapy resistance is less robust.
KEY_REFERENCES: CREATE-X trial; NCCN Guidelines Breast Cancer v3.2025
GUIDELINE_ALIGNMENT: NCCN (2025) guidelines recommend considering chemotherapy for patients with residual disease after neoadjuvant therapy, though evidence is stronger for chemotherapy resistance than endocrine resistance.
SUBGROUP_NOTES: The extent of nodal involvement (3/11) significantly increases recurrence risk, warranting chemotherapy despite initial low-risk features.
CONFIDENCE_LEVEL: Moderate
WEB_SEARCH_USED: No - Based on established principles; no new evidence through Feb 2025 specifically addresses this scenario.

---END---

QUESTION_ID: 45
SELECTED_ANSWER: A
EVIDENCE_SUMMARY: For node-positive ER+ breast cancer (1/4 nodes), the standard duration of endocrine therapy is 5 years, with extension to 10 years considered based on individual risk assessment. Recent NCCN Guidelines (v3.2025) recommend 5 years as the minimum, with extended therapy considered for higher-risk patients.
KEY_REFERENCES: ATLAS trial; aTTom trial; NCCN Guidelines Breast Cancer v3.2025
GUIDELINE_ALIGNMENT: NCCN (2025) and ASCO guidelines recommend 5-10 years of endocrine therapy for node-positive breast cancer, with duration individualized based on risk factors.
SUBGROUP_NOTES: With only 1 positive node and moderate tumor size, 5 years may be sufficient, but longer duration could be considered if other risk factors are present.
CONFIDENCE_LEVEL: High
WEB_SEARCH_USED: Yes - Confirmed NCCN v3.2025 maintains recommendation for 5-10 years of endocrine therapy in node-positive disease.

---END---

QUESTION_ID: 46
SELECTED_ANSWER: B
EVIDENCE_SUMMARY: For node-negative ER+ breast cancer with high genomic risk (Oncotype DX 28), 7-8 years of endocrine therapy is often recommended to balance recurrence reduction with side effects. While 5 years is minimum, extended therapy provides additional risk reduction as shown in MA.17R and DATA trials.
KEY_REFERENCES: MA.17R trial; DATA trial; NCCN Guidelines Breast Cancer v3.2025
GUIDELINE_ALIGNMENT: NCCN (2025) guidelines recommend considering extended endocrine therapy for 7-10 years in patients with higher recurrence risk.
SUBGROUP_NOTES: High genomic risk score justifies extended therapy duration despite node-negative status.
CONFIDENCE_LEVEL: High
WEB_SEARCH_USED: No - Based on established trials; no new evidence through Feb 2025 changes this recommendation.

---END---

QUESTION_ID: 47
SELECTED_ANSWER: C
EVIDENCE_SUMMARY: For ER+ breast cancer with 3/8 positive nodes, 10 years of endocrine therapy is recommended due to the high risk of late recurrence. The ATLAS and aTTom trials demonstrated significant reduction in late recurrences and breast cancer mortality with 10 years of tamoxifen.
KEY_REFERENCES: ATLAS trial; aTTom trial; NCCN Guidelines Breast Cancer v3.2025

GUIDELINE_ALIGNMENT: NCCN (2025) and ASCO guidelines recommend 10 years of endocrine therapy for node-positive breast cancer with ≥4 positive nodes or other high-risk features.
SUBGROUP_NOTES: The significant nodal burden (3/8 nodes) places this patient at high risk for late recurrence, warranting extended therapy.
CONFIDENCE_LEVEL: High
WEB_SEARCH_USED: Yes - Verified NCCN v3.2025 continues to recommend extended endocrine therapy for high-risk node-positive patients.

---END---

QUESTION_ID: 48
SELECTED_ANSWER: B
EVIDENCE_SUMMARY: For a postmenopausal woman with T2N0 ER+ breast cancer and Oncotype DX score of 28, TC chemotherapy (docetaxel/ cyclophosphamide) is recommended based on TAILORx which showed benefit for scores >25. TC is preferred over AC/T for its favorable toxicity profile and equivalent efficacy in lower-risk patients.
KEY_REFERENCES: TAILORx trial; WSG-AGO EC-Doc trial; NCCN Guidelines Breast Cancer v3.2025
GUIDELINE_ALIGNMENT: NCCN (2025) guidelines recommend chemotherapy for Oncotype DX scores >25 in postmenopausal women, with TC as a preferred regimen.
SUBGROUP_NOTES: The moderately high recurrence score (28) indicates significant chemotherapy benefit despite node-negative status.
CONFIDENCE_LEVEL: High
WEB_SEARCH_USED: No - Based on established trials; no new evidence through Feb 2025 changes this recommendation.

---END---

QUESTION_ID: 49
SELECTED_ANSWER: A
EVIDENCE_SUMMARY: For a postmenopausal woman with T2N0 ER+ breast cancer and Oncotype DX score of 32, AC/T chemotherapy is recommended due to the high recurrence risk. While TC is often used for lower-risk cases, the very high score (32) may warrant the more robust anthracycline-based regimen, especially given the T2 size.
KEY_REFERENCES: TAILORx trial; NCCN Guidelines Breast Cancer v3.2025
GUIDELINE_ALIGNMENT: NCCN (2025) guidelines recommend chemotherapy for Oncotype DX scores >25, with regimen choice based on risk level.
SUBGROUP_NOTES: The very high score (32) and T2 size suggest substantial recurrence risk, favoring a more intensive regimen.
CONFIDENCE_LEVEL: Moderate
WEB_SEARCH_USED: No - Based on established knowledge; no new evidence through Feb 2025 specifically addresses regimen choice for very high scores.

---END---

QUESTION_ID: 50
SELECTED_ANSWER: A
EVIDENCE_SUMMARY: For this T1cN0 grade 3 tumor with Oncotype DX

score of 32, AC/T chemotherapy is recommended due to the combination of high genomic risk and adverse pathological features (grade 3). The high score indicates chemotherapy benefit, and the grade 3 histology suggests potential responsiveness to anthracyclines.
KEY_REFERENCES: TAILORx trial; NCCN Guidelines Breast Cancer v3.2025
GUIDELINE_ALIGNMENT: NCCN (2025) guidelines recommend chemotherapy for Oncotype DX scores >25, with anthracycline-based regimens considered for higher-risk features.
SUBGROUP_NOTES: The grade 3 histology adds to the concern despite the small tumor size, favoring a more robust chemotherapy regimen.
CONFIDENCE_LEVEL: High
WEB_SEARCH_USED: No - Based on established knowledge; no new evidence through Feb 2025 changes this approach.

---END---

QUESTION_ID: 51
SELECTED_ANSWER: A
EVIDENCE_SUMMARY: For this node-positive (3/11) ER+ breast cancer with low Oncotype DX score of 13, no chemotherapy is recommended based on RxPONDER trial results which showed minimal benefit from chemotherapy in postmenopausal women with scores ≤25. Recent NCCN Guidelines (v3.2025) continue to support chemotherapy omission in this scenario.
KEY_REFERENCES: RxPONDER trial; NCCN Guidelines Breast Cancer v3.2025
GUIDELINE_ALIGNMENT: NCCN (2025) and ASCO guidelines recommend against chemotherapy for postmenopausal women with 1-3 positive nodes and Oncotype DX scores ≤25.
SUBGROUP_NOTES: The low score (13) indicates minimal chemotherapy benefit despite nodal involvement.
CONFIDENCE_LEVEL: High
WEB_SEARCH_USED: Yes - Confirmed NCCN v3.2025 maintains recommendation against chemotherapy for scores ≤25 in postmenopausal women with 1-3 positive nodes.

---END---

QUESTION_ID: 52
SELECTED_ANSWER: C
EVIDENCE_SUMMARY: For this N2 disease (4/11 nodes) with Oncotype DX score of 13, AC/T chemotherapy is recommended despite the low score. The RxPONDER trial included only 1-3 positive nodes, and patients with ≥4 nodes were not represented, so chemotherapy is generally recommended based on the high nodal burden regardless of genomic score.
KEY_REFERENCES: RxPONDER trial; NCCN Guidelines Breast Cancer v3.2025
GUIDELINE_ALIGNMENT: NCCN (2025) guidelines recommend chemotherapy for patients with ≥4 positive nodes regardless of genomic score.
SUBGROUP_NOTES: The extensive nodal involvement (N2) outweighs the low genomic score in determining recurrence risk and chemotherapy benefit.
CONFIDENCE_LEVEL: High

WEB_SEARCH_USED: Yes - Verified NCCN v3.2025 continues to recommend chemotherapy for ≥4 positive nodes regardless of genomic score.

---END---

QUESTION_ID: 53
SELECTED_ANSWER: A
EVIDENCE_SUMMARY: For this premenopausal woman with node-negative ER+ breast cancer and low Oncotype DX score of 12, tamoxifen alone is sufficient based on TAILORx results which showed minimal chemotherapy benefit for scores <26. Recent NCCN Guidelines (v3.2025) support endocrine therapy alone in this scenario.
KEY_REFERENCES: TAILORx trial; SOFT trial; NCCN Guidelines Breast Cancer v3.2025
GUIDELINE_ALIGNMENT: NCCN (2025) guidelines recommend endocrine therapy alone for premenopausal women with node-negative disease and Oncotype DX scores <26.
SUBGROUP_NOTES: The low score (12) indicates excellent prognosis with endocrine therapy alone.
CONFIDENCE_LEVEL: High
WEB_SEARCH_USED: No - Based on established trials; no new evidence through Feb 2025 changes this recommendation.

---END---

QUESTION_ID: 54
SELECTED_ANSWER: A
EVIDENCE_SUMMARY: For this premenopausal woman with node-negative ER+ breast cancer and Oncotype DX score of 18, tamoxifen alone is recommended. The score is below the threshold (26) where chemotherapy benefit was demonstrated in TAILORx for premenopausal women.
KEY_REFERENCES: TAILORx trial; NCCN Guidelines Breast Cancer v3.2025
GUIDELINE_ALIGNMENT: NCCN (2025) guidelines recommend endocrine therapy alone for node-negative disease with Oncotype DX scores <26.
SUBGROUP_NOTES: The intermediate score (18) still falls below the chemotherapy benefit threshold for node-negative disease.
CONFIDENCE_LEVEL: High
WEB_SEARCH_USED: No - Based on established trials; no new evidence through Feb 2025 changes this recommendation.

---END---

QUESTION_ID: 55
SELECTED_ANSWER: D
EVIDENCE_SUMMARY: For this premenopausal woman with node-negative ER+ breast cancer and Oncotype DX score of 23, chemotherapy followed by endocrine therapy is recommended. TAILORx showed that premenopausal women with scores ≥16 may derive some chemotherapy benefit, and scores ≥26 show clear benefit.
KEY_REFERENCES: TAILORx trial; NCCN Guidelines Breast Cancer v3.2025
GUIDELINE_ALIGNMENT: NCCN (2025) guidelines recommend considering chemotherapy for premenopausal women with node-negative disease and Oncotype DX scores 16-25, and strongly recommend for scores ≥26.

SUBGROUP_NOTES: The score of 23 is in the intermediate range where chemotherapy benefit is uncertain but often recommended for premenopausal women.
CONFIDENCE_LEVEL: Moderate
WEB_SEARCH_USED: No - Based on established trials; no new evidence through Feb 2025 changes this recommendation.

---END---

QUESTION_ID: 56
SELECTED_ANSWER: B
EVIDENCE_SUMMARY: For this premenopausal woman with 1 positive node and low Oncotype DX score of 12, OFS + tamoxifen is recommended. The RxPONDER trial showed minimal chemotherapy benefit for scores ≤25 in premenopausal women with 1-3 positive nodes, but OFS improves outcomes compared to tamoxifen alone as shown in SOFT/TEXT trials.
KEY_REFERENCES: RxPONDER trial; SOFT/TEXT trials; NCCN Guidelines Breast Cancer v3.2025
GUIDELINE_ALIGNMENT: NCCN (2025) guidelines recommend OFS + endocrine therapy for premenopausal women with higher-risk features, and against chemotherapy for scores ≤25 with 1-3 positive nodes.
SUBGROUP_NOTES: The low score (12) indicates minimal chemotherapy benefit, but nodal involvement justifies intensification of endocrine therapy with OFS.
CONFIDENCE_LEVEL: High
WEB_SEARCH_USED: Yes - Confirmed NCCN v3.2025 maintains recommendation against chemotherapy for scores ≤25 in premenopausal women with 1-3 positive nodes.

---END---

QUESTION_ID: 57
SELECTED_ANSWER: D
EVIDENCE_SUMMARY: For this premenopausal woman with 1 positive node and Oncotype DX score of 18, chemotherapy followed by endocrine therapy is recommended. The RxPONDER trial showed that premenopausal women with 1-3 positive nodes and intermediate scores may derive chemotherapy benefit, though the threshold is not precisely defined.
KEY_REFERENCES: RxPONDER trial; SOFT/TEXT trials; NCCN Guidelines Breast Cancer v3.2025
GUIDELINE_ALIGNMENT: NCCN (2025) guidelines recommend considering chemotherapy for premenopausal women with 1-3 positive nodes and intermediate Oncotype DX scores (16-25).
SUBGROUP_NOTES: The intermediate score (18) combined with nodal involvement suggests potential chemotherapy benefit in premenopausal women.
CONFIDENCE_LEVEL: Moderate
WEB_SEARCH_USED: No - Based on established trials; no new evidence through Feb 2025 changes this recommendation.

---END---

QUESTION_ID: 58
SELECTED_ANSWER: D

EVIDENCE_SUMMARY: For this premenopausal woman with 1 positive node and Oncotype DX score of 23, chemotherapy followed by endocrine therapy is strongly recommended. The RxPONDER trial suggested chemotherapy benefit for premenopausal women with scores above 16-20 range, and scores ≥25 show clear benefit.
KEY_REFERENCES: RxPONDER trial; NCCN Guidelines Breast Cancer v3.2025
GUIDELINE_ALIGNMENT: NCCN (2025) guidelines recommend chemotherapy for premenopausal women with 1-3 positive nodes and Oncotype DX scores ≥26, and strongly consider for scores 16-25.
SUBGROUP_NOTES: The score of 23 is close to the threshold where clear benefit was demonstrated, warranting chemotherapy.
CONFIDENCE_LEVEL: High
WEB_SEARCH_USED: No - Based on established trials; no new evidence through Feb 2025 changes this recommendation.

---END---

QUESTION_ID: 59
SELECTED_ANSWER: B
EVIDENCE_SUMMARY: For this premenopausal woman with 2 positive nodes and low Oncotype DX score of 12, OFS + endocrine therapy is recommended without chemotherapy. The RxPONDER trial included patients with 1-3 positive nodes and showed minimal chemotherapy benefit for scores ≤25 in premenopausal women.
KEY_REFERENCES: RxPONDER trial; SOFT/TEXT trials; NCCN Guidelines Breast Cancer v3.2025
GUIDELINE_ALIGNMENT: NCCN (2025) guidelines recommend against chemotherapy for premenopausal women with 1-3 positive nodes and Oncotype DX scores ≤25.
SUBGROUP_NOTES: The low score (12) indicates minimal chemotherapy benefit despite two positive nodes.
CONFIDENCE_LEVEL: High
WEB_SEARCH_USED: Yes - Verified NCCN v3.2025 continues to recommend against chemotherapy for scores ≤25 in premenopausal women with 1-3 positive nodes.

---END---

QUESTION_ID: 60
SELECTED_ANSWER: D
EVIDENCE_SUMMARY: For this premenopausal woman with 2 positive nodes and Oncotype DX score of 18, chemotherapy followed by endocrine therapy is recommended. The RxPONDER trial showed that premenopausal women with 1-3 positive nodes and intermediate scores may derive chemotherapy benefit.
KEY_REFERENCES: RxPONDER trial; NCCN Guidelines Breast Cancer v3.2025
GUIDELINE_ALIGNMENT: NCCN (2025) guidelines recommend considering chemotherapy for premenopausal women with 1-3 positive nodes and Oncotype DX scores 16-25.
SUBGROUP_NOTES: The intermediate score (18) combined with two positive nodes suggests chemotherapy benefit.
CONFIDENCE_LEVEL: Moderate

WEB_SEARCH_USED: No - Based on established trials; no new evidence through Feb 2025 changes this recommendation.

---END---

QUESTION_ID: 61
SELECTED_ANSWER: D
EVIDENCE_SUMMARY: For this premenopausal woman with 1 positive node and Oncotype DX score of 23, chemotherapy followed by endocrine therapy is recommended. The score is in the range where RxPONDER showed chemotherapy benefit for premenopausal women with 1-3 positive nodes.
KEY_REFERENCES: RxPONDER trial; NCCN Guidelines Breast Cancer v3.2025
GUIDELINE_ALIGNMENT: NCCN (2025) guidelines recommend chemotherapy for premenopausal women with 1-3 positive nodes and Oncotype DX scores ≥26, and strongly consider for scores 16-25.
SUBGROUP_NOTES: The score of 23 is close to the threshold where clear benefit was demonstrated, warranting chemotherapy despite only one positive node.
CONFIDENCE_LEVEL: High
WEB_SEARCH_USED: No - Based on established trials; no new evidence through Feb 2025 changes this recommendation.

---END---

QUESTION_ID: 62
SELECTED_ANSWER: D
EVIDENCE_SUMMARY: For this premenopausal woman with 1 positive node and high Ki67 (30%), chemotherapy followed by endocrine therapy is recommended. High Ki67 suggests proliferative disease and potential chemotherapy responsiveness, regardless of genomic score.
KEY_REFERENCES: IBCSG trials; NCCN Guidelines Breast Cancer v3.2025
GUIDELINE_ALIGNMENT: NCCN (2025) guidelines consider high Ki67 as a factor favoring chemotherapy in addition to genomic testing results.
SUBGROUP_NOTES: The high proliferation rate (Ki67 30%) indicates aggressive biology and likely chemotherapy benefit despite the small tumor size.
CONFIDENCE_LEVEL: High
WEB_SEARCH_USED: No - Based on established knowledge; no new evidence through Feb 2025 changes this approach.

---END---

QUESTION_ID: 63
SELECTED_ANSWER: B
EVIDENCE_SUMMARY: For this patient with residual breast tumor but node-negative status after neoadjuvant chemotherapy, adjuvant CDK4/6 inhibitor is not recommended. The monarchE trial eligibility required ≥4 positive nodes or 1-3 nodes with additional high-risk features, which are not present here.
KEY_REFERENCES: monarchE trial; NCCN Guidelines Breast Cancer v3.2025
GUIDELINE_ALIGNMENT: NCCN (2025) guidelines recommend CDK4/6

inhibitors only for high-risk HR+ breast cancer with ≥4 positive nodes or 1-3 nodes with additional risk factors.
SUBGROUP_NOTES: The residual breast disease alone does not meet the high-risk criteria for CDK4/6 inhibitor use.
CONFIDENCE_LEVEL: High
WEB_SEARCH_USED: Yes - Confirmed NCCN v3.2025 maintains strict criteria for adjuvant CDK4/6 inhibitor use.

---END---

QUESTION_ID: 64
SELECTED_ANSWER: A
EVIDENCE_SUMMARY: For this patient with residual breast tumor and 1 positive node after neoadjuvant chemotherapy, adjuvant CDK4/6 inhibitor is recommended. The monarchE trial included patients with 1-3 positive nodes plus additional risk factors (such as residual disease after neoadjuvant chemotherapy), showing improved outcomes with abemaciclib.
KEY_REFERENCES: monarchE trial; NCCN Guidelines Breast Cancer v3.2025
GUIDELINE_ALIGNMENT: NCCN (2025) guidelines recommend CDK4/6 inhibitors for patients with 1-3 positive nodes and additional high-risk features such as residual disease after neoadjuvant chemotherapy.
SUBGROUP_NOTES: The combination of residual breast disease and positive node meets high-risk criteria for CDK4/6 inhibitor use.
CONFIDENCE_LEVEL: High
WEB_SEARCH_USED: Yes - Verified NCCN v3.2025 continues to recommend CDK4/6 inhibitors for this high-risk scenario.

---END---

QUESTION_ID: 65
SELECTED_ANSWER: B
EVIDENCE_SUMMARY: For this postmenopausal woman with node-negative ER+ breast cancer and Oncotype DX score of 25, chemotherapy followed by AI is recommended. TAILORx showed chemotherapy benefit for scores >25 in postmenopausal women, and the score of 25 is at the threshold where benefit may be present.
KEY_REFERENCES: TAILORx trial; NCCN Guidelines Breast Cancer v3.2025
GUIDELINE_ALIGNMENT: NCCN (2025) guidelines recommend considering chemotherapy for postmenopausal women with node-negative disease and Oncotype DX scores 21-25, and strongly recommend for scores >25.
SUBGROUP_NOTES: The score of 25 is at the threshold where chemotherapy benefit becomes more certain.
CONFIDENCE_LEVEL: Moderate
WEB_SEARCH_USED: No - Based on established trials; no new evidence through Feb 2025 changes this recommendation.

---END---

QUESTION_ID: 66
SELECTED_ANSWER: B
EVIDENCE_SUMMARY: For this postmenopausal woman with node-negative

ER+ breast cancer and Oncotype DX score of 26, chemotherapy followed by AI is recommended. TAILORx showed clear chemotherapy benefit for scores >25 in postmenopausal women.
KEY_REFERENCES: TAILORx trial; NCCN Guidelines Breast Cancer v3.2025
GUIDELINE_ALIGNMENT: NCCN (2025) guidelines recommend chemotherapy for postmenopausal women with node-negative disease and Oncotype DX scores >25.
SUBGROUP_NOTES: The score of 26 is above the threshold where clear chemotherapy benefit was demonstrated.
CONFIDENCE_LEVEL: High
WEB_SEARCH_USED: No - Based on established trials; no new evidence through Feb 2025 changes this recommendation.

---END---

QUESTION_ID: 67
SELECTED_ANSWER: A
EVIDENCE_SUMMARY: For this postmenopausal woman with node-negative ER+ breast cancer and Ki67 of 26%, AI alone is recommended. While high Ki67 suggests proliferation, treatment decisions should primarily be based on genomic testing when available, as Ki67 has significant variability in measurement.
KEY_REFERENCES: TAILORx trial; NCCN Guidelines Breast Cancer v3.2025
GUIDELINE_ALIGNMENT: NCCN (2025) guidelines emphasize genomic testing over Ki67 for chemotherapy decisions in node-negative ER+ breast cancer.
SUBGROUP_NOTES: Without a genomic score, the high Ki67 might suggest chemotherapy consideration, but genomic testing would provide more precise guidance.
CONFIDENCE_LEVEL: Moderate
WEB_SEARCH_USED: No - Based on established knowledge; no new evidence through Feb 2025 changes this approach.

---END---

QUESTION_ID: 68
SELECTED_ANSWER: A
EVIDENCE_SUMMARY: For this postmenopausal woman with 1 positive node and Oncotype DX score of 18, AI alone is recommended. The RxPONDER trial showed minimal chemotherapy benefit for scores ≤25 in postmenopausal women with 1-3 positive nodes.
KEY_REFERENCES: RxPONDER trial; NCCN Guidelines Breast Cancer v3.2025
GUIDELINE_ALIGNMENT: NCCN (2025) guidelines recommend against chemotherapy for postmenopausal women with 1-3 positive nodes and Oncotype DX scores ≤25.
SUBGROUP_NOTES: The low score (18) indicates minimal chemotherapy benefit despite nodal involvement.
CONFIDENCE_LEVEL: High
WEB_SEARCH_USED: Yes - Confirmed NCCN v3.2025 maintains recommendation against chemotherapy for scores ≤25 in postmenopausal women with 1-3 positive nodes.

---END---

QUESTION_ID: 69
SELECTED_ANSWER: A
EVIDENCE_SUMMARY: For this postmenopausal woman with 1 positive node and Ki67 of 18%, AI alone is recommended. Without a high-risk genomic score, chemotherapy benefit is likely minimal based on RxPONDER principles extrapolated to Ki67.
KEY_REFERENCES: RxPONDER trial; NCCN Guidelines Breast Cancer v3.2025
GUIDELINE_ALIGNMENT: NCCN (2025) guidelines recommend genomic testing to guide chemotherapy decisions in node-positive ER+ breast cancer, with Ki67 being less definitive.
SUBGROUP_NOTES: The moderate Ki67 (18%) alone does not justify chemotherapy in the absence of other high-risk features.
CONFIDENCE_LEVEL: Moderate
WEB_SEARCH_USED: No - Based on established principles; no new evidence through Feb 2025 changes this approach.

---END---

QUESTION_ID: 70
SELECTED_ANSWER: C
EVIDENCE_SUMMARY: For this postmenopausal woman with 2 positive nodes and very low Oncotype DX score of 11, AI + CDK4/6 inhibitor is recommended. The monarchE trial showed benefit with abemaciclib in high-risk HR+ breast cancer, which includes patients with ≥4 positive nodes or 1-3 nodes with additional risk factors, but not for very low genomic risk.
KEY_REFERENCES: monarchE trial; NCCN Guidelines Breast Cancer v3.2025
GUIDELINE_ALIGNMENT: NCCN (2025) guidelines recommend CDK4/6 inhibitors only for high-risk HR+ breast cancer, which is not clearly met with a very low genomic score.
SUBGROUP_NOTES: The very low score (11) suggests excellent prognosis with endocrine therapy alone, and CDK4/6 inhibitors may not be necessary.
CONFIDENCE_LEVEL: Low
WEB_SEARCH_USED: Yes - Verified NCCN v3.2025 maintains strict criteria for CDK4/6 inhibitor use, which may not be met with a very low genomic score.

---END---

QUESTION_ID: 71
SELECTED_ANSWER: A
EVIDENCE_SUMMARY: For this postmenopausal woman with 2 positive nodes and low Ki67 of 11%, AI alone is recommended. The low proliferation rate suggests indolent disease, and without other high-risk features, CDK4/6 inhibitors are not indicated.
KEY_REFERENCES: monarchE trial; NCCN Guidelines Breast Cancer v3.2025
GUIDELINE_ALIGNMENT: NCCN (2025) guidelines recommend CDK4/6 inhibitors only for high-risk HR+ breast cancer, which is not clearly met with low Ki67.

SUBGROUP_NOTES: The low proliferation rate (Ki67 11%) suggests indolent disease with good prognosis on endocrine therapy alone.
CONFIDENCE_LEVEL: Moderate
WEB_SEARCH_USED: No - Based on established knowledge; no new evidence through Feb 2025 changes this approach.

---END---

QUESTION_ID: 72
SELECTED_ANSWER: B
EVIDENCE_SUMMARY: Men with ER+ breast cancer should not be offered CDK4/6 inhibitor treatment based on the same criteria as women, as they were excluded from the major trials (monarchE, NATALEE) establishing these indications. Treatment decisions for men should be based on extrapolation from women's data but with caution due to lack of specific evidence.
KEY_REFERENCES: monarchE trial; NCCN Guidelines Breast Cancer v3.2025
GUIDELINE_ALIGNMENT: NCCN (2025) guidelines note that evidence for CDK4/6 inhibitors in men is limited, and decisions should be made on a case-by-case basis.
SUBGROUP_NOTES: Male breast cancer is rare, and specific trial data are lacking, necessitating careful individualized decision-making.
CONFIDENCE_LEVEL: Moderate
WEB_SEARCH_USED: Yes - Confirmed NCCN v3.2025 continues to note the limited evidence for CDK4/6 inhibitors in male breast cancer.

---END---
