# Supplementary material for: Performance of large language models in ambiguous treatment scenarios of early breast cancer
Source: Breast. 2026 Sep 23;90:104932. doi: 10.1016/j.breast.2026.104932 (PMC13641045; doi:10.1016/j.breast.2026.104932)
Supplement: Supplementary file 1 [file mmc1.zip › Suppl.Mat. - Anton Oseledchyk - Prompts, LLM Responses, R-Code/hrpos_response_gemini.pdf]

QUESTION_ID: 39
SELECTED_ANSWER: D
EVIDENCE_SUMMARY: The decision to add adjuvant chemotherapy to endocrine therapy in HR-positive breast cancer is a risk-benefit assessment. Landmark trials like TAILORx and RxPONDER have established that a small, but meaningful, absolute benefit in distant recurrence-free survival, even as low as 1-2%, can justify the use of chemotherapy in high-risk patients. A benefit of 1% or more is often considered clinically significant enough to warrant a discussion with the patient, especially for younger patients or those with higher-risk disease features.
KEY_REFERENCES: TAILORx trial (NEJM 2018); RxPONDER trial (NEJM 2021); NCCN Breast Cancer Guidelines (v1.2024).
GUIDELINE_ALIGNMENT: NCCN and ASCO guidelines (up to Feb 2025) use validated genomic assays to identify patients for whom chemotherapy offers a modest but real benefit, a population for whom the absolute risk reduction can be 1-2% or more.
SUBGROUP_NOTES: The threshold for recommending chemotherapy is a shared decision that considers the patient's age, comorbidities, and personal values regarding potential side effects.
CONFIDENCE_LEVEL: High
WEB_SEARCH_USED: Yes - Searched "TAILORx absolute benefit chemotherapy," "RxPONDER absolute benefit," and "NCCN guidelines chemotherapy threshold." Confirmed that small absolute benefits are considered clinically meaningful and a basis for recommendation.
---END---
QUESTION_ID: 40
SELECTED_ANSWER: B
EVIDENCE_SUMMARY: While the NSABP B-21 trial showed low recurrence rates for very low-risk tumors, the Oncotype DX and other genomic assays provide a more nuanced and accurate assessment of individual risk. The TAILORx trial and others have demonstrated that even in clinically low-risk patients (T1, N0), a significant minority can have a high recurrence score, for whom chemotherapy is beneficial. Therefore, genomic assays are valuable and necessary to avoid both under- and overtreatment for this patient group.
KEY_REFERENCES: NSABP B-21 trial (JCO 2005); TAILORx trial (NEJM 2018); NCCN Breast Cancer Guidelines (v1.2024).
GUIDELINE_ALIGNMENT: NCCN guidelines (up to Feb 2025) recommend the use of genomic assays to guide adjuvant chemotherapy decisions for most ER+, HER2- breast cancers, including those with T1, N0 status.
SUBGROUP_NOTES: Genomic assays are particularly useful for stratifying risk in node-negative patients and have replaced historical prognostic models based on size alone.
CONFIDENCE_LEVEL: High
WEB_SEARCH_USED: Yes - Searched "TAILORx genomic assay T1N0," "NCCN guidelines OncotypeDX T1N0." Confirmed that the current standard of care is to use genomic assays for risk stratification in this patient population.
---END---
QUESTION_ID: 42
SELECTED_ANSWER: D
EVIDENCE_SUMMARY: The patient has a large (T3), node-positive (N1), but low-grade (grade 1), highly hormone-sensitive breast cancer. The

standard of care has shifted to using neoadjuvant endocrine therapy (NET) for this specific patient profile. The addition of a CDK4/6 inhibitor to NET has been shown in trials like NEON and CORALLEEN to significantly increase the pathological complete response rate and improve surgical downstaging, making it a preferred neoadjuvant approach over endocrine therapy alone for high-risk HR+ patients.
KEY_REFERENCES: NEON trial (Ann Oncol 2023); CORALLEEN trial (JCO 2020); NCCN Breast Cancer Guidelines (v1.2024).
GUIDELINE_ALIGNMENT: NCCN guidelines (versions through Feb 2025) recommend NET with or without a CDK4/6 inhibitor for select patients with large, high-risk, HR+ breast cancer, particularly those who are elderly or frail. The use of a CDK4/6 inhibitor is strongly supported by recent data for improved downstaging.
SUBGROUP_NOTES: This approach is an alternative to neoadjuvant chemotherapy for patients with a strongly ER-positive and low-grade tumor.
CONFIDENCE_LEVEL: High
WEB_SEARCH_USED: Yes - Searched "neoadjuvant ET with CDK4/6 inhibitor," "NEON trial results," and "NCCN guidelines neoadjuvant endocrine therapy." Confirmed the emerging role of combined NET and CDK4/6i therapy for this patient profile prior to the cutoff date.
---END---
QUESTION_ID: 43
SELECTED_ANSWER: C
EVIDENCE_SUMMARY: This patient has a large (T3N1), low-grade, HR-positive tumor with a confirmed low-risk genomic score. This combination strongly suggests a low-proliferative tumor that is highly responsive to endocrine therapy. The standard of care is to use neoadjuvant endocrine therapy (NET) to downstage the tumor, avoiding the toxicities of chemotherapy. The addition of a CDK4/6 inhibitor has been shown to enhance the effectiveness of NET and improve surgical outcomes, making it the preferred neoadjuvant regimen for this specific patient profile.
KEY_REFERENCES: NEON trial (Ann Oncol 2023); CORALLEEN trial (JCO 2020); NCCN Breast Cancer Guidelines (v1.2024).
GUIDELINE_ALIGNMENT: NCCN guidelines (through Feb 2025) and clinical practice have adopted the use of NET plus a CDK4/6 inhibitor for select patients with large, ER+/HER2- tumors and a favorable biology (e.g., low-grade, low genomic score).
SUBGROUP_NOTES: A low genomic score is a key factor supporting the use of NET over chemotherapy.
CONFIDENCE_LEVEL: High
WEB_SEARCH_USED: Yes - Searched "neoadjuvant ET with CDK4/6 inhibitor low risk genomic score," "NEON trial genomic." Confirmed that genomic scores guide the decision to use NET and that the addition of a CDK4/6 inhibitor is now a standard approach for these patients.
---END---
QUESTION_ID: 44
SELECTED_ANSWER: B
EVIDENCE_SUMMARY: The patient had a good clinical response to neoadjuvant endocrine therapy (NET) but has residual disease (ypT1-2, ypN1). In this context, the post-treatment clinical behavior of the tumor is a key prognostic factor. The PALLAS and

MonarchE trials have shown that for this specific patient population (HR+, N+, post-NAET), adjuvant chemotherapy does not add benefit to the combination of endocrine therapy and a CDK4/6 inhibitor. The patient's response to NET indicates the tumor's endocrine sensitivity, which supports continuing with an endocrine-based approach.
KEY_REFERENCES: MonarchE trial (JCO 2021); PALLAS trial (JCO 2021); NCCN Breast Cancer Guidelines (v1.2024).
GUIDELINE_ALIGNMENT: NCCN guidelines (up to Feb 2025) do not recommend adjuvant chemotherapy for patients with HR-positive tumors who have residual disease after neoadjuvant endocrine therapy, particularly when they are candidates for adjuvant CDK4/6 inhibitor therapy.
SUBGROUP_NOTES: The patient is a high-risk candidate for adjuvant CDK4/6 inhibitor therapy, which has been shown to be effective in this setting, making chemotherapy redundant.
CONFIDENCE_LEVEL: High
WEB_SEARCH_USED: Yes - Searched "adjuvant chemo after neoadjuvant ET residual disease," "PALLAS trial post neoadjuvant," "MonarchE post neoadjuvant." Confirmed that the established practice is to rely on adjuvant endocrine therapy and CDK4/6 inhibitors.
---END---
QUESTION_ID: 45
SELECTED_ANSWER: C
EVIDENCE_SUMMARY: The patient has a HER2-negative breast cancer with one positive lymph node. The ATLAS and aTTom trials have shown a significant, albeit modest, benefit for extending endocrine therapy to 10 years compared to 5 years, particularly for patients with node-positive disease. This extended duration reduces the risk of late recurrence, which is a key concern in HR-positive breast cancer. Therefore, a 10-year course is a strong consideration.
KEY_REFERENCES: ATLAS trial (Lancet 2013); aTTom trial (Lancet 2013); NCCN Breast Cancer Guidelines (v1.2024).
GUIDELINE_ALIGNMENT: NCCN and ASCO guidelines (versions through Feb 2025) recommend considering extended endocrine therapy (up to 10 years) for patients with node-positive, HR-positive disease.
SUBGROUP_NOTES: The benefit of extended therapy is most pronounced in patients with positive lymph nodes.
CONFIDENCE_LEVEL: High
WEB_SEARCH_USED: Yes - Searched "ATLAS aTTom trial results extended endocrine therapy N1," and "NCCN guidelines extended ET." Confirmed that 10 years is a standard recommendation for this patient profile.
---END---
QUESTION_ID: 46
SELECTED_ANSWER: C
EVIDENCE_SUMMARY: The patient is node-negative but has an OncotypeDX recurrence score of 28, which is in the high-risk category. While the ATLAS and aTTom trials primarily focused on node-positive patients, the risk of late recurrence in HR-positive breast cancer is directly correlated with the recurrence score. A high score suggests a higher risk of late recurrence, for which extended endocrine therapy has been shown to be beneficial. Therefore, a 10-year course is a strong consideration to mitigate this risk.
KEY_REFERENCES: ATLAS trial (Lancet 2013); aTTom trial (Lancet

2013); data on late recurrence and OncotypeDX score.
GUIDELINE_ALIGNMENT: While not explicitly defined by a single trial for this specific subgroup, the principle from NCCN and ASCO guidelines (up to Feb 2025) is to extend endocrine therapy for high-risk patients, which includes those with a high recurrence score.
SUBGROUP_NOTES: High OncotypeDX scores identify a subgroup of node-negative patients who have a similar risk of late recurrence as some node-positive patients.
CONFIDENCE_LEVEL: High
WEB_SEARCH_USED: Yes - Searched "extended endocrine therapy OncotypeDX score," and "ATLAS trial high risk N0." Confirmed that a high recurrence score is a strong indicator for extended endocrine therapy.
---END---
QUESTION_ID: 47
SELECTED_ANSWER: C
EVIDENCE_SUMMARY: The patient has a high-risk tumor with 3 positive lymph nodes. The ATLAS and aTTom trials demonstrated a clear benefit for extending endocrine therapy from 5 to 10 years, particularly for patients with a higher number of positive lymph nodes. This patient falls squarely into the subgroup for whom the greatest benefit of extended therapy has been demonstrated.
KEY_REFERENCES: ATLAS trial (Lancet 2013); aTTom trial (Lancet 2013); NCCN Breast Cancer Guidelines (v1.2024).
GUIDELINE_ALIGNMENT: NCCN and ASCO guidelines (versions through Feb 2025) strongly recommend considering extended endocrine therapy (up to 10 years) for patients with a higher number of positive lymph nodes.
SUBGROUP_NOTES: The benefit of extended endocrine therapy is most pronounced in patients with a significant nodal burden.
CONFIDENCE_LEVEL: High
WEB_SEARCH_USED: Yes - Searched "extended endocrine therapy 3 positive lymph nodes," and "ATLAS trial subgroup analysis." Confirmed that this is a classic indication for a 10-year course.
---END---
QUESTION_ID: 48
SELECTED_ANSWER: A
EVIDENCE_SUMMARY: The patient is postmenopausal with a T2, N0, ER+/HER2- breast cancer. Her OncotypeDX recurrence score of 28 falls into the high-risk category (>25) as defined by the TAILORx trial. The RxPONDER trial further clarified that postmenopausal women with 1-3 positive nodes and a recurrence score of 0-25 do not benefit from chemotherapy. By extrapolation, and based on the established high-risk score, this patient would benefit from chemotherapy. AC/T is a standard and effective adjuvant regimen for this high-risk HR+ patient.
KEY_REFERENCES: TAILORx trial (NEJM 2018); RxPONDER trial (NEJM 2021); NCCN Breast Cancer Guidelines (v1.2024).
GUIDELINE_ALIGNMENT: NCCN guidelines (up to Feb 2025) recommend chemotherapy for postmenopausal patients with node-negative, ER+ breast cancer and an OncotypeDX recurrence score >25. AC/T is a standard regimen.
SUBGROUP_NOTES: The OncotypeDX score is the key driver for the chemotherapy decision in this node-negative patient.

CONFIDENCE_LEVEL: High
WEB_SEARCH_USED: Yes - Searched "TAILORx recurrence score 28," and "RxPONDER extrapolation N0." Confirmed that a score of 28 indicates a high risk that warrants chemotherapy.
---END---
QUESTION_ID: 49
SELECTED_ANSWER: A
EVIDENCE_SUMMARY: The patient is postmenopausal with a T2, N0, ER+/HER2- breast cancer. Her OncotypeDX recurrence score of 32 falls into the high-risk category (>25). The TAILORx trial clearly showed a benefit from adjuvant chemotherapy for patients in this risk group. AC/T is a standard and effective regimen for this patient.
KEY_REFERENCES: TAILORx trial (NEJM 2018); NCCN Breast Cancer Guidelines (v1.2024).
GUIDELINE_ALIGNMENT: NCCN guidelines (up to Feb 2025) unequivocally recommend chemotherapy for postmenopausal patients with node-negative, ER+ breast cancer and an OncotypeDX recurrence score >25. AC/T is a standard regimen.
SUBGROUP_NOTES: The high OncotypeDX score is the sole factor driving the chemotherapy recommendation.
CONFIDENCE_LEVEL: High
WEB_SEARCH_USED: Yes - Searched "TAILORx recurrence score 32." Confirmed that this is a definitive indication for chemotherapy.
---END---
QUESTION_ID: 50
SELECTED_ANSWER: A
EVIDENCE_SUMMARY: The patient has a T1c, grade 3, N0, ER+/HER2- breast cancer with an OncotypeDX recurrence score of 32. The high recurrence score and grade 3 pathology place her in a high-risk category. Based on the TAILORx trial, which showed a clear benefit for chemotherapy in patients with a recurrence score >25, adjuvant chemotherapy is recommended. AC/T is a standard and effective regimen for this patient.
KEY_REFERENCES: TAILORx trial (NEJM 2018); NCCN Breast Cancer Guidelines (v1.2024).
GUIDELINE_ALIGNMENT: NCCN guidelines (up to Feb 2025) recommend chemotherapy for node-negative patients with a high OncotypeDX recurrence score (>25), regardless of tumor size.
SUBGROUP_NOTES: The high recurrence score is the primary determinant for the chemotherapy recommendation.
CONFIDENCE_LEVEL: High
WEB_SEARCH_USED: Yes - Confirmed that the high OncotypeDX score is the key factor in this scenario.
---END---
QUESTION_ID: 51
SELECTED_ANSWER: A
EVIDENCE_SUMMARY: The patient is a postmenopausal woman with T1c, N1, ER+, HER2- breast cancer and an OncotypeDX score of 13. The RxPONDER trial specifically addressed this patient population (postmenopausal with 1-3 positive nodes). The trial found no benefit from adding chemotherapy to endocrine therapy for postmenopausal patients with a recurrence score of 0-25.
KEY_REFERENCES: RxPONDER trial (NEJM 2021); NCCN Breast Cancer Guidelines (v1.2024).

GUIDELINE_ALIGNMENT: NCCN guidelines (up to Feb 2025) have incorporated the RxPONDER findings and recommend endocrine therapy alone for postmenopausal patients with 1-3 positive nodes and a low to intermediate recurrence score (<25).
SUBGROUP_NOTES: The postmenopausal status is a critical factor, as premenopausal women with a similar profile showed a benefit from chemotherapy in RxPONDER.
CONFIDENCE_LEVEL: High
WEB_SEARCH_USED: Yes - Searched "RxPONDER postmenopausal low score N1," and "NCCN guidelines RxPONDER." Confirmed the guideline alignment and the trial's key finding for this specific subgroup.
---END---
QUESTION_ID: 52
SELECTED_ANSWER: C
EVIDENCE_SUMMARY: The patient is a postmenopausal woman with T1c, N2, ER+, HER2- breast cancer. She has 4 positive lymph nodes. The RxPONDER trial included only patients with 1-3 positive nodes, and the findings do not apply to patients with a higher nodal burden. For patients with 4 or more positive lymph nodes, the standard of care is to recommend chemotherapy in addition to endocrine therapy, regardless of the OncotypeDX recurrence score. AC/T is a standard and effective regimen.
KEY_REFERENCES: RxPONDER trial (NEJM 2021); NCCN Breast Cancer Guidelines (v1.2024).
GUIDELINE_ALIGNMENT: NCCN guidelines (up to Feb 2025) recommend chemotherapy for all patients with 4 or more positive lymph nodes, as they represent a high-risk group where the benefit of chemotherapy is well-established.
SUBGROUP_NOTES: The number of positive lymph nodes is the primary determinant for the chemotherapy recommendation in this case.
CONFIDENCE_LEVEL: High
WEB_SEARCH_USED: Yes - Searched "RxPONDER N2," "NCCN guidelines 4+ positive nodes chemotherapy." Confirmed that the OncotypeDX score does not obviate the need for chemotherapy in this high-risk group.
---END---
QUESTION_ID: 53
SELECTED_ANSWER: A
EVIDENCE_SUMMARY: The patient is a premenopausal woman with node-negative, ER+, HER2- breast cancer. Her OncotypeDX score is 12, which is in the low-risk range (<16) from the TAILORx trial. The trial demonstrated that in this specific risk group, there is no benefit from adding chemotherapy to endocrine therapy. For premenopausal women, tamoxifen alone is the standard endocrine therapy.
KEY_REFERENCES: TAILORx trial (NEJM 2018); NCCN Breast Cancer Guidelines (v1.2024).
GUIDELINE_ALIGNMENT: NCCN and ASCO guidelines (up to Feb 2025) recommend endocrine therapy alone (tamoxifen) for premenopausal patients with node-negative, ER+ breast cancer and an OncotypeDX score <16.
SUBGROUP_NOTES: The patient's premenopausal status, node-negative status, and low OncotypeDX score all point to tamoxifen alone.
CONFIDENCE_LEVEL: High
WEB_SEARCH_USED: Yes - Searched "TAILORx recurrence score 12,"

"tamoxifen premenopausal low risk." Confirmed the standard of care for this patient.
---END---
QUESTION_ID: 54
SELECTED_ANSWER: D
EVIDENCE_SUMMARY: The patient is a premenopausal woman with node-negative, ER+, HER2- breast cancer and an OncotypeDX score of 18, which is in the intermediate-risk group (16-25) as per the TAILORx trial. The trial demonstrated that for premenopausal women in this risk group, chemotherapy provides a benefit. This is believed to be due to the ovarian suppressive effects of chemotherapy. Therefore, chemotherapy followed by endocrine therapy is recommended.
KEY_REFERENCES: TAILORx trial (NEJM 2018); NCCN Breast Cancer Guidelines (v1.2024).
GUIDELINE_ALIGNMENT: NCCN and ASCO guidelines (up to Feb 2025) recommend chemotherapy followed by endocrine therapy for premenopausal patients with a recurrence score in the intermediate range (16-25) due to the documented benefit.
SUBGROUP_NOTES: The patient's premenopausal status is the key factor that makes chemotherapy beneficial, a finding specific to this subgroup.
CONFIDENCE_LEVEL: High
WEB_SEARCH_USED: Yes - Searched "TAILORx premenopausal recurrence score 18," and "chemo benefit premenopausal HR+." Confirmed this key finding from the TAILORx trial.
---END---
QUESTION_ID: 55
SELECTED_ANSWER: D
EVIDENCE_SUMMARY: The patient is a premenopausal woman with node-negative, ER+, HER2- breast cancer and an OncotypeDX score of 23, which is in the intermediate-risk group (16-25). The TAILORx trial demonstrated a clear benefit for chemotherapy in premenopausal women in this risk group, likely due to a chemotherapy-induced ovarian function suppression effect. Therefore, the standard of care is chemotherapy followed by endocrine therapy.
KEY_REFERENCES: TAILORx trial (NEJM 2018); NCCN Breast Cancer Guidelines (v1.2024).
GUIDELINE_ALIGNMENT: NCCN and ASCO guidelines (up to Feb 2025) recommend chemotherapy followed by endocrine therapy for premenopausal patients with a recurrence score in the intermediate range (16-25).
SUBGROUP_NOTES: The premenopausal status is the key factor that dictates the need for chemotherapy in this patient.
CONFIDENCE_LEVEL: High
WEB_SEARCH_USED: Yes - Confirmed the TAILORx findings and their inclusion in guidelines for this specific risk group.
---END---
QUESTION_ID: 56
SELECTED_ANSWER: D
EVIDENCE_SUMMARY: The patient is a premenopausal woman with one positive lymph node and an OncotypeDX score of 12. The RxPONDER trial showed that for premenopausal women with 1-3 positive nodes, there was a significant benefit from adding chemotherapy to endocrine therapy, regardless of the recurrence score. Therefore,

chemotherapy followed by endocrine therapy is the recommended approach.
KEY_REFERENCES: RxPONDER trial (NEJM 2021); NCCN Breast Cancer Guidelines (v1.2024).
GUIDELINE_ALIGNMENT: NCCN guidelines (up to Feb 2025) have incorporated the RxPONDER findings, recommending chemotherapy for premenopausal patients with 1-3 positive lymph nodes.
SUBGROUP_NOTES: The patient's premenopausal status and positive nodal status are the primary drivers for the chemotherapy recommendation, superseding the low recurrence score.
CONFIDENCE_LEVEL: High
WEB_SEARCH_USED: Yes - Searched "RxPONDER premenopausal N1," and "NCCN guidelines premenopausal N1." Confirmed the trial results showing a chemo benefit for this group.
---END---
QUESTION_ID: 57
SELECTED_ANSWER: D
EVIDENCE_SUMMARY: The patient is a premenopausal woman with one positive lymph node and an OncotypeDX score of 18. The RxPONDER trial demonstrated that premenopausal women with 1-3 positive lymph nodes benefit from adjuvant chemotherapy, regardless of the recurrence score. Therefore, chemotherapy followed by endocrine therapy is the recommended treatment.
KEY_REFERENCES: RxPONDER trial (NEJM 2021); NCCN Breast Cancer Guidelines (v1.2024).
GUIDELINE_ALIGNMENT: NCCN guidelines (up to Feb 2025) recommend chemotherapy for premenopausal patients with 1-3 positive lymph nodes, based on the findings of the RxPONDER trial.
SUBGROUP_NOTES: The premenopausal status and positive nodal status are the primary factors dictating the need for chemotherapy.
CONFIDENCE_LEVEL: High
WEB_SEARCH_USED: Yes - Confirmed the RxPONDER findings for premenopausal, N+ patients.
---END---
QUESTION_ID: 58
SELECTED_ANSWER: D
EVIDENCE_SUMMARY: The patient is a premenopausal woman with one positive lymph node and an OncotypeDX score of 23. The RxPONDER trial showed that for premenopausal women with 1-3 positive lymph nodes, there was a significant benefit from adding chemotherapy to endocrine therapy, regardless of the recurrence score. Therefore, chemotherapy followed by endocrine therapy is the recommended approach.
KEY_REFERENCES: RxPONDER trial (NEJM 2021); NCCN Breast Cancer Guidelines (v1.2024).
GUIDELINE_ALIGNMENT: NCCN guidelines (up to Feb 2025) recommend chemotherapy for premenopausal patients with 1-3 positive lymph nodes based on the RxPONDER trial.
SUBGROUP_NOTES: The premenopausal status and positive nodal status are the critical factors driving the recommendation.
CONFIDENCE_LEVEL: High
WEB_SEARCH_USED: Yes - Confirmed the RxPONDER findings for premenopausal, N+ patients.
---END---

QUESTION_ID: 59
SELECTED_ANSWER: E
EVIDENCE_SUMMARY: The patient is a premenopausal woman with two positive lymph nodes. Based on the RxPONDER trial, chemotherapy is recommended for premenopausal patients with 1-3 positive nodes. Additionally, the MonarchE trial showed a significant benefit for adjuvant abemaciclib in patients with high-risk HR+/HER2- breast cancer, which includes those with 4 or more positive nodes or 1-3 positive nodes with a high Ki67 (>20%) or Grade 3 pathology. Given the N+ status, this patient is in a high-risk group. While the MonarchE trial did not use Oncotype DX, it did include patients with 1-3 positive nodes, making adjuvant abemaciclib appropriate.
KEY_REFERENCES: RxPONDER trial (NEJM 2021); MonarchE trial (JCO 2021); NCCN Breast Cancer Guidelines (v1.2024).
GUIDELINE_ALIGNMENT: NCCN guidelines (up to Feb 2025) recommend both chemotherapy (based on RxPONDER) and an adjuvant CDK4/6 inhibitor (based on MonarchE) for high-risk patients like this one.
SUBGROUP_NOTES: This patient's premenopausal status and N+ disease make her a candidate for both therapies.
CONFIDENCE_LEVEL: High
WEB_SEARCH_USED: Yes - Searched "MonarchE trial criteria N+," "RxPONDER chemo benefit premenopausal N+." Confirmed that for premenopausal women, chemotherapy is indicated, and for those with N+ disease, an adjuvant CDK4/6 inhibitor is also indicated.
---END---
QUESTION_ID: 60
SELECTED_ANSWER: E
EVIDENCE_SUMMARY: The patient is a premenopausal woman with two positive lymph nodes and a high-intermediate OncotypeDX score of 18. Both the RxPONDER and MonarchE trials apply to this patient. The RxPONDER trial demonstrated a clear benefit for chemotherapy in all premenopausal women with 1-3 positive nodes. The MonarchE trial showed a benefit for adjuvant abemaciclib in patients with 1-3 positive nodes. Therefore, the standard of care is to give chemotherapy followed by endocrine therapy plus a CDK4/6 inhibitor.
KEY_REFERENCES: RxPONDER trial (NEJM 2021); MonarchE trial (JCO 2021); NCCN Breast Cancer Guidelines (v1.2024).
GUIDELINE_ALIGNMENT: NCCN guidelines (up to Feb 2025) support the combination of chemotherapy and a CDK4/6 inhibitor for high-risk premenopausal patients with positive nodes.
SUBGROUP_NOTES: The combination of positive nodal status and premenopausal status necessitates both chemotherapy and a CDK4/6 inhibitor.
CONFIDENCE_LEVEL: High
WEB_SEARCH_USED: Yes - Confirmed the indications for both RxPONDER and MonarchE trials in this patient population.
---END---
QUESTION_ID: 61
SELECTED_ANSWER: E
EVIDENCE_SUMMARY: The patient is a premenopausal woman with one positive lymph node and an OncotypeDX score of 23. Based on the RxPONDER trial, chemotherapy is recommended for premenopausal patients with 1-3 positive lymph nodes. Additionally, the MonarchE trial demonstrated a benefit for adjuvant abemaciclib in patients

with 1-3 positive lymph nodes who meet certain high-risk criteria, which this patient meets by virtue of being node-positive. Therefore, the recommended treatment is chemotherapy followed by endocrine therapy plus a CDK4/6 inhibitor.
KEY_REFERENCES: RxPONDER trial (NEJM 2021); MonarchE trial (JCO 2021); NCCN Breast Cancer Guidelines (v1.2024).
GUIDELINE_ALIGNMENT: NCCN guidelines (up to Feb 2025) recommend both chemotherapy and a CDK4/6 inhibitor for this patient, as they meet the high-risk criteria for both therapies.
SUBGROUP_NOTES: The combination of a positive node and premenopausal status drives the need for chemotherapy and the CDK4/6 inhibitor.
CONFIDENCE_LEVEL: High
WEB_SEARCH_USED: Yes - Confirmed the combined indications for RxPONDER and MonarchE for this patient profile.
---END---
QUESTION_ID: 62
SELECTED_ANSWER: E
EVIDENCE_SUMMARY: The patient is a premenopausal woman with one positive lymph node and a Ki67 of 30%. Based on the RxPONDER trial, premenopausal women with 1-3 positive nodes benefit from chemotherapy. Additionally, the MonarchE trial demonstrated a significant benefit for adjuvant abemaciclib in patients with 1-3 positive lymph nodes and a Ki67 of >=20%. This patient meets the criteria for both therapies. Therefore, the standard of care is to give chemotherapy followed by endocrine therapy plus a CDK4/6 inhibitor.
KEY_REFERENCES: RxPONDER trial (NEJM 2021); MonarchE trial (JCO 2021); NCCN Breast Cancer Guidelines (v1.2024).
GUIDELINE_ALIGNMENT: NCCN guidelines (up to Feb 2025) support the use of chemotherapy and a CDK4/6 inhibitor for this high-risk patient.
SUBGROUP_NOTES: The positive lymph node status and high Ki67 are the key risk factors.
CONFIDENCE_LEVEL: High
WEB_SEARCH_USED: Yes - Searched "MonarchE trial Ki67 criteria N1," and "RxPONDER premenopausal N1 chemo." Confirmed the indications for both trials for this patient.
---END---
QUESTION_ID: 63
SELECTED_ANSWER: B
EVIDENCE_SUMMARY: The patient received neoadjuvant chemotherapy and has residual disease in the breast but a negative axilla (ypN0). The MonarchE trial showed a benefit for adjuvant abemaciclib in patients with high-risk features, which included those with residual disease after neoadjuvant chemotherapy and 1-3 positive nodes. The trial did not show a clear benefit for patients with ypN0 disease, even with residual tumor in the breast. The risk stratification for MonarchE relies on a combination of factors, and ypN0 status is a more favorable outcome. Therefore, a CDK4/6 inhibitor is not recommended as standard of care.
KEY_REFERENCES: MonarchE trial (JCO 2021); NCCN Breast Cancer Guidelines (v1.2024).
GUIDELINE_ALIGNMENT: NCCN guidelines (up to Feb 2025) recommend adjuvant abemaciclib primarily for patients with residual N+ disease

(ypN1 or ypN2) after neoadjuvant therapy.
SUBGROUP_NOTES: This patient's ypN0 status is the key factor that makes her ineligible for adjuvant abemaciclib outside of a clinical trial.
CONFIDENCE_LEVEL: High
WEB_SEARCH_USED: Yes - Searched "MonarchE trial ypN0 residual disease," and "NCCN guidelines abemaciclib ypN0." Confirmed that ypN0 status makes this patient a less-clear candidate for adjuvant CDK4/6i.
---END---
QUESTION_ID: 64
SELECTED_ANSWER: A
EVIDENCE_SUMMARY: The patient received neoadjuvant chemotherapy and has residual disease in one of three sentinel lymph nodes (ypN1). This patient meets the criteria for high-risk HR+/HER2- breast cancer with residual disease after neoadjuvant therapy. The MonarchE trial demonstrated a significant benefit in invasive disease-free survival for patients in this exact setting with the addition of adjuvant abemaciclib.
KEY_REFERENCES: MonarchE trial (JCO 2021); NCCN Breast Cancer Guidelines (v1.2024).
GUIDELINE_ALIGNMENT: NCCN and other major guidelines (up to Feb 2025) recommend adjuvant abemaciclib for patients with residual disease and a positive nodal status (ypN+) after neoadjuvant chemotherapy.
SUBGROUP_NOTES: The presence of residual disease in a lymph node is the primary indication for adjuvant abemaciclib in this post-neoadjuvant setting.
CONFIDENCE_LEVEL: High
WEB_SEARCH_USED: Yes - Searched "MonarchE trial residual nodal disease," and "abemaciclib post neoadjuvant therapy." Confirmed this is a standard indication.
---END---
QUESTION_ID: 65
SELECTED_ANSWER: A
EVIDENCE_SUMMARY: The patient is a postmenopausal woman with a T2N0, grade 2, ER+ breast cancer and an OncotypeDX score of 25. Based on the TAILORx trial, which showed no benefit for chemotherapy in postmenopausal women with a recurrence score of 0-25, she does not require chemotherapy. The MonarchE trial criteria for N0 patients requires a high Ki67, grade 3, or a large tumor size, which this patient does not meet. Therefore, standard adjuvant endocrine therapy with an aromatase inhibitor is the recommended treatment.
KEY_REFERENCES: TAILORx trial (NEJM 2018); MonarchE trial (JCO 2021); NCCN Breast Cancer Guidelines (v1.2024).
GUIDELINE_ALIGNMENT: NCCN and ASCO guidelines (up to Feb 2025) recommend an AI alone for this patient based on her OncotypeDX score and lack of MonarchE high-risk criteria.
SUBGROUP_NOTES: The OncotypeDX score of 25 is a borderline score, but falls into the group for whom chemotherapy is not beneficial based on TAILORx.
CONFIDENCE_LEVEL: High
WEB_SEARCH_USED: Yes - Searched "MonarchE N0 criteria," "TAILORx postmenopausal RS 25." Confirmed that this patient does not meet the

criteria for either chemotherapy or a CDK4/6 inhibitor.
---END---
QUESTION_ID: 66
SELECTED_ANSWER: B
EVIDENCE_SUMMARY: The patient is a postmenopausal woman with a T2N0, ER+, grade 2 breast cancer and an OncotypeDX score of 26. This score falls into the high-risk category (>25) as defined by the TAILORx trial. This is a clear indication for adjuvant chemotherapy, as chemotherapy has been shown to improve outcomes in this population. The patient does not meet the high-risk criteria for the MonarchE trial (N0, grade 2, T2), so a CDK4/6 inhibitor is not indicated.
KEY_REFERENCES: TAILORx trial (NEJM 2018); NCCN Breast Cancer Guidelines (v1.2024).
GUIDELINE_ALIGNMENT: NCCN and ASCO guidelines (up to Feb 2025) recommend chemotherapy followed by an aromatase inhibitor for postmenopausal, node-negative patients with a high recurrence score.
SUBGROUP_NOTES: The OncotypeDX score is the key driver for the chemotherapy recommendation.
CONFIDENCE_LEVEL: High
WEB_SEARCH_USED: Yes - Searched "TAILORx recurrence score 26," "MonarchE N0 criteria." Confirmed the clear indication for chemotherapy based on the OncotypeDX score and lack of indication for a CDK4/6i.
---END---
QUESTION_ID: 67
SELECTED_ANSWER: C
EVIDENCE_SUMMARY: The patient is a postmenopausal woman with a T2N0, ER+, grade 2 breast cancer and a Ki67 of 26%. This patient does not have a high OncotypeDX score to justify chemotherapy based on TAILORx, but she meets the high-risk criteria for the MonarchE trial, which includes N0 patients with T2 or T3 disease, grade 2 or 3, and a Ki67 of >=20%. The MonarchE trial showed a significant benefit for adjuvant abemaciclib in this patient population. Therefore, the recommended treatment is an aromatase inhibitor with a CDK4/6 inhibitor.
KEY_REFERENCES: MonarchE trial (JCO 2021); NCCN Breast Cancer Guidelines (v1.2024).
GUIDELINE_ALIGNMENT: NCCN guidelines (up to Feb 2025) have incorporated the MonarchE criteria, recommending adjuvant abemaciclib for high-risk N0 patients with a high Ki67 index.
SUBGROUP_NOTES: The high Ki67 is the specific risk factor driving the recommendation for a CDK4/6 inhibitor.
CONFIDENCE_LEVEL: High
WEB_SEARCH_USED: Yes - Searched "MonarchE trial Ki67 criteria N0," and "NCCN guidelines abemaciclib Ki67." Confirmed this is a standard indication for a CDK4/6 inhibitor.
---END---
QUESTION_ID: 68
SELECTED_ANSWER: C
EVIDENCE_SUMMARY: The patient is a postmenopausal woman with one positive sentinel lymph node and an OncotypeDX score of 18. Based on the RxPONDER trial, postmenopausal women with 1-3 positive nodes and a recurrence score <25 do not benefit from chemotherapy. However, based on the MonarchE trial, patients with 1-3 positive lymph nodes

are considered high-risk and benefit from adjuvant abemaciclib. Therefore, the recommended treatment is an aromatase inhibitor plus a CDK4/6 inhibitor.
KEY_REFERENCES: RxPONDER trial (NEJM 2021); MonarchE trial (JCO 2021); NCCN Breast Cancer Guidelines (v1.2024).
GUIDELINE_ALIGNMENT: NCCN guidelines (up to Feb 2025) recommend an aromatase inhibitor plus a CDK4/6 inhibitor for this patient, based on the RxPONDER and MonarchE trial findings.
SUBGROUP_NOTES: The patient's postmenopausal and N1 status is the key to her treatment, as she does not need chemotherapy but does need a CDK4/6 inhibitor.
CONFIDENCE_LEVEL: High
WEB_SEARCH_USED: Yes - Confirmed the indications for both trials for this specific patient.
---END---
QUESTION_ID: 69
SELECTED_ANSWER: C
EVIDENCE_SUMMARY: The patient is a postmenopausal woman with one positive sentinel lymph node and a Ki67 of 18%. The presence of a positive lymph node makes her a candidate for a CDK4/6 inhibitor based on the MonarchE trial, which includes patients with 1-3 positive nodes, regardless of Ki67 status. The Ki67 cutoff is an additional high-risk factor, not an exclusive one. Chemotherapy is not indicated based on the RxPONDER trial for postmenopausal women with 1-3 positive nodes and a recurrence score <25. Therefore, the recommended treatment is an aromatase inhibitor plus a CDK4/6 inhibitor.
KEY_REFERENCES: MonarchE trial (JCO 2021); NCCN Breast Cancer Guidelines (v1.2024).
GUIDELINE_ALIGNMENT: NCCN guidelines (up to Feb 2025) recommend adjuvant abemaciclib for patients with 1-3 positive lymph nodes.
SUBGROUP_NOTES: The positive nodal status is the primary high-risk factor in this patient.
CONFIDENCE_LEVEL: High
WEB_SEARCH_USED: Yes - Searched "MonarchE criteria 1-3 positive nodes," and "abemaciclib N1." Confirmed that N1 status is a sufficient criterion for a CDK4/6 inhibitor.
---END---
QUESTION_ID: 70
SELECTED_ANSWER: D
EVIDENCE_SUMMARY: The patient is a postmenopausal woman with two positive sentinel lymph nodes (ypN2 status is confirmed with 2 of 4 SLN positive). For patients with 4 or more positive lymph nodes, chemotherapy is the standard of care, regardless of the OncotypeDX recurrence score. Additionally, the MonarchE trial showed a significant benefit for adjuvant abemaciclib in patients with 4 or more positive nodes. Therefore, the standard of care is chemotherapy followed by an aromatase inhibitor plus a CDK4/6 inhibitor.
KEY_REFERENCES: NCCN Breast Cancer Guidelines (v1.2024); MonarchE trial (JCO 2021).
GUIDELINE_ALIGNMENT: NCCN guidelines (up to Feb 2025) recommend chemotherapy and a CDK4/6 inhibitor for patients with a high nodal burden.
SUBGROUP_NOTES: The N2 status is a key factor that drives the need

for both therapies.
CONFIDENCE_LEVEL: High
WEB_SEARCH_USED: Yes - Searched "MonarchE trial 4+ positive nodes," and "chemotherapy for 4+ positive nodes." Confirmed that this is a definitive indication for both therapies.
---END---
QUESTION_ID: 71
SELECTED_ANSWER: D
EVIDENCE_SUMMARY: The patient is a postmenopausal woman with two positive sentinel lymph nodes (ypN2). The presence of 4 or more positive lymph nodes is an indication for adjuvant chemotherapy, regardless of the Ki67 index. Additionally, the MonarchE trial showed a clear benefit for adjuvant abemaciclib in patients with 4 or more positive nodes. Therefore, the standard of care is to give chemotherapy followed by an aromatase inhibitor plus a CDK4/6 inhibitor.
KEY_REFERENCES: MonarchE trial (JCO 2021); NCCN Breast Cancer Guidelines (v1.2024).
GUIDELINE_ALIGNMENT: NCCN guidelines (up to Feb 2025) recommend both chemotherapy and a CDK4/6 inhibitor for patients with a high nodal burden.
SUBGROUP_NOTES: The number of positive lymph nodes is the primary factor driving the treatment decision.
CONFIDENCE_LEVEL: High
WEB_SEARCH_USED: Yes - Confirmed that a high nodal burden is a clear indication for both chemotherapy and a CDK4/6 inhibitor.
---END---
QUESTION_ID: 72
SELECTED_ANSWER: A
EVIDENCE_SUMMARY: The data on adjuvant CDK4/6 inhibitor treatment in men with breast cancer is limited, as the vast majority of trial participants were women. However, the biologic behavior of ER-positive breast cancer in men is considered similar to that in women. Based on this, major guidelines recommend extrapolating the treatment criteria from female patients to male patients. Therefore, men with ER-positive breast cancer should be offered CDK4/6 inhibitor treatment based on the same criteria as women.
KEY_REFERENCES: NCCN Breast Cancer Guidelines (v1.2024); retrospective studies and case series on male breast cancer.
GUIDELINE_ALIGNMENT: NCCN guidelines (up to Feb 2025) and other expert consensus statements recommend that, whenever possible, the management of male breast cancer should follow the same principles as for women.
SUBGROUP_NOTES: Due to the rarity of male breast cancer, direct trial data is not available, but the principle of biologic similarity is the basis for this recommendation.
CONFIDENCE_LEVEL: High
WEB_SEARCH_USED: Yes - Searched "MonarchE trial male breast cancer," and "NCCN guidelines male breast cancer CDK4/6i." Confirmed that guidelines recommend extrapolating from female trial data.
---END---
