# Supplementary material for: Performance of large language models in ambiguous treatment scenarios of early breast cancer
Source: Breast. 2026 Sep 23;90:104932. doi: 10.1016/j.breast.2026.104932 (PMC13641045; doi:10.1016/j.breast.2026.104932)
Supplement: Supplementary file 1 [file mmc1.zip › Suppl.Mat. - Anton Oseledchyk - Prompts, LLM Responses, R-Code/Prompt_Axillary_Management_questions.pdf]

You are an expert physician analyzing breast cancer clinical questions. Please use BOTH your comprehensive medical knowledge AND web search to provide the best evidence-based answers.

CRITICAL TIME RESTRICTION:
- Only use evidence published up to FEBRUARY 28, 2025
- EXCLUDE the St. Gallen 2025 International Consensus Conference
- Any evidence or guidelines after February 2025 should NOT be used

APPROACH FOR EACH QUESTION:
1. FIRST: Use your extensive static knowledge of:
   - Established clinical trials (ACOSOG Z0011, AMAROS, TAILORx, RxPONDER, KEYNOTE-522, etc.)
   - Standard guidelines and their historical recommendations
   - Fundamental oncology principles and standard of care
   - Well-established evidence through your knowledge cutoff

2. THEN: Use WEB SEARCH to:
   - Find recent updates ONLY through February 2025
   - Verify guideline versions (up to Feb 2025)
   - Check for trial results published before March 2025
   - Find consensus statements (excluding St. Gallen 2025)
   - Confirm if established recommendations have changed

3. SYNTHESIZE: Combine both sources for the most complete answer:
   - Base your reasoning on established evidence
   - Update with findings from web search (through Feb 2025 only)
   - Note if web search confirms or modifies established knowledge

IMPORTANT:
- Use BOTH static knowledge AND web search for comprehensive analysis
- STRICTLY EXCLUDE: St. Gallen 2025 conference and any evidence after Feb 28, 2025
- Your static knowledge provides the foundation
- Web search provides updates and verification (through Feb 2025)
- Cite both established studies and recent updates
- Be explicit about what comes from your knowledge vs. web search

For EACH question below, provide your answer in EXACTLY this format:

QUESTION_ID: [the question ID]
SELECTED_ANSWER: [single letter A-G only]
EVIDENCE_SUMMARY: [2-3 sentences combining established evidence and recent updates through Feb 2025]
KEY_REFERENCES: [List 2-3 most important studies - both landmark trials and recent updates through Feb 2025]
GUIDELINE_ALIGNMENT: [Which guidelines support this - versions through Feb 2025 only]
SUBGROUP_NOTES: [Any relevant patient factors that might modify recommendation]
CONFIDENCE_LEVEL: [High/Moderate/Low based on evidence quality and consistency]
WEB_SEARCH_USED: [Yes - list what you searched for and what updates

through Feb 2025 you found]
---END---

Now analyze ALL 15 questions from Axillary_Management:

=== QUESTION ID: 1 ===

QUESTION: A 60 year old woman has been diagnosed with early stage breast cancer. A core biopsy shows grade 2 invasive ductal carcinoma. Axillary exam and axillary ultrasound are negative. Based on clinical exam and imaging, up to what size tumor would you be comfortable ornitting sentinel lymph node surgery? - Tumor is strongly ER+, PR+, HER2 negative

OPTIONS:
A: 1.0 cm or less
B: 1.5 cm
C: 2.0 cm
D: 2.5 cm
E: 3.0 cm
F: Any cT1 or cT2 lesion
G: Perform SLN surgery regardless of size

=== QUESTION ID: 2 ===

QUESTION: A 60 year old woman has been diagnosed with early stage breast cancer. A core biopsy shows grade 2 invasive ductal carcinoma. Axillary exam and axillary ultrasound are negative. Based on clinical exam and imaging, up to what size tumor would you be comfortable ornitting sentinel lymph node surgery? - Tumor is strongly ER+, PR+, HER2 positive

OPTIONS:
A: 1.0 cm or less
B: 1.5 cm
C: 2.0 cm
D: 2.5 cm
E: 3.0 cm
F: Any cT1 or cT2 lesion
G: Perform SLN surgery regardless of size

=== QUESTION ID: 3 ===

QUESTION: A 60 year old woman has been diagnosed with early stage breast cancer. A core biopsy shows grade 2 invasive ductal carcinoma. Axillary exam and axillary ultrasound are negative. Based on clinical exam and imaging, up to what size tumor would you be comfortable ornitting sentinel lymph node surgery? - Tumor is triple-negative

OPTIONS:

A: 1.0 cm or less
B: 1.5 cm
C: 2.0 cm
D: 2.5 cm
E: 3.0 cm
F: Any cT1 or cT2 lesion
G: Perform SLN surgery regardless of size

=== QUESTION ID: 4 ===

QUESTION: The same criteria for omission of SNB should apply to invasive ductal carcinomas and to invasive lobular carcinomas

OPTIONS:
A: Agree
B: Disagree as we have limited data on ILC

=== QUESTION ID: 5 ===

QUESTION: A patient with breast cancer has received neoadjuvant chemotherapy with clinical response, and at the time of sentinel lymph node surgery. She will receive radiation therapy to the breast. What additional axillary therapy would you recommend? - ER positive, HER2 negative - ITC, 1 of 4 SLN

OPTIONS:
A: None
B: ALND
C: Nodal irradiation
D: ALND + nodal irradiation

=== QUESTION ID: 6 ===

QUESTION: A patient with breast cancer has received neoadjuvant chemotherapy with clinical response, and at the time of sentinel lymph node surgery. She will receive radiation therapy to the breast. What additional axillary therapy would you recommend? - ER positive, HER2 negative - Micrometastatic residual disease, 1 of 4 SLN

OPTIONS:
A: None
B: ALND
C: Nodal irradiation
D: ALND + nodal irradiation

=== QUESTION ID: 7 ===

QUESTION: A patient with breast cancer has received neoadjuvant chemotherapy with clinical response, and at the time of sentinel

lymph node surgery. She will receive radiation therapy to the breast. What additional axillary therapy would you recommend? - ER positive, HER2 negative - Micrometastatic residual disease, 2 of 4 SLN

OPTIONS:
A: None
B: ALND
C: Nodal irradiation
D: ALND + nodal irradiation

=== QUESTION ID: 8 ===

QUESTION: A patient with breast cancer has received neoadjuvant chemotherapy with clinical response, and at the time of sentinel lymph node surgery. She will receive radiation therapy to the breast. What additional axillary therapy would you recommend? - Triple negative - ITC, 1 of 4 SLN

OPTIONS:
A: None
B: ALND
C: Nodal irradiation
D: ALND + nodal irradiation

=== QUESTION ID: 9 ===

QUESTION: A patient with breast cancer has received neoadjuvant chemotherapy with clinical response, and at the time of sentinel lymph node surgery. She will receive radiation therapy to the breast. What additional axillary therapy would you recommend? - Triple negative - Micrometastatic residual disease, 1 of 4 SLN

OPTIONS:
A: None
B: ALND
C: Nodal irradiation
D: ALND + nodal irradiation

=== QUESTION ID: 10 ===

QUESTION: A patient with breast cancer has received neoadjuvant chemotherapy with clinical response, and at the time of sentinel lymph node surgery. She will receive radiation therapy to the breast. What additional axillary therapy would you recommend? - Triple negative - Micrometastatic residual disease, 2 of 4 SLN

OPTIONS:
A: None
B: ALND
C: Nodal irradiation

D: ALND + nodal irradiation

=== QUESTION ID: 11 ===

QUESTION: A patient with breast cancer has received neoadjuvant chemotherapy with clinical response, and at the time of sentinel lymph node surgery. She will receive radiation therapy to the breast. What additional axillary therapy would you recommend? - HER2 positive - ITC, 1 of 4 SLN

OPTIONS:
A: None
B: ALND
C: Nodal irradiation
D: ALND + nodal irradiation

=== QUESTION ID: 12 ===

QUESTION: A patient with breast cancer has received neoadjuvant chemotherapy with clinical response, and at the time of sentinel lymph node surgery. She will receive radiation therapy to the breast. What additional axillary therapy would you recommend? - HER2 positive - Micrometastatic residual disease, 1 of 4 SLN

OPTIONS:
A: None
B: ALND
C: Nodal irradiation
D: ALND + nodal irradiation

=== QUESTION ID: 13 ===

QUESTION: A patient with breast cancer has received neoadjuvant chemotherapy with clinical response, and at the time of sentinel lymph node surgery. She will receive radiation therapy to the breast. What additional axillary therapy would you recommend? - HER2 positive - Micrometastatic residual disease, 2 of 4 SLN

OPTIONS:
A: None
B: ALND
C: Nodal irradiation
D: ALND + nodal irradiation

=== QUESTION ID: 14 ===

QUESTION: A patient with ER- breast cancer has received neoadjuvant chemotherapy with clinical response, and at the time of sentinel lymph node surgery had macrometastatic residual disease in one of the sentinel lymph nodes. She will receive radiation therapy to the

breast. What additional axillary therapy would you recommend for ypN1 disease {3 mm) in 1 of 4 sentinel lymph nodes?

OPTIONS:
A: None
B: ALND
C: Nodal irradiation
D: ALND + nodal irradiation

=== QUESTION ID: 15 ===

QUESTION: A patient with HER 2 positive breast cancer has received neoadjuvant chemotherapy with clinical response, and at the time of sentinel lymph node surgery had residua l macrometastatic disease in one of the sentinel lymph nodes. She will receive radiation therapy to the breast. What additional axillary therapy would you recommend for ypN1 disease (3 mm) in 1 of 4 sentinel lymph nodes?

OPTIONS:
A: None
B: ALND
C: Nodal irradiation
D: ALND + nodal irradiation

CRITICAL REMINDERS:
1. START with your comprehensive static medical knowledge
2. ENHANCE with web search for updates ONLY through February 28, 2025
3. EXCLUDE St. Gallen 2025 conference findings
4. SYNTHESIZE both sources for the most complete, current answer (up to Feb 2025)
5. Format each response exactly as specified above with ---END--- after each question
6. Answer ALL questions above in order
