# Supplementary material for: Performance of large language models in ambiguous treatment scenarios of early breast cancer
Source: Breast. 2026 Sep 23;90:104932. doi: 10.1016/j.breast.2026.104932 (PMC13641045; doi:10.1016/j.breast.2026.104932)
Supplement: Supplementary file 1 [file mmc1.zip › Suppl.Mat. - Anton Oseledchyk - Prompts, LLM Responses, R-Code/Prompt_FollowUp_Survival_questions.pdf]

You are an expert physician analyzing breast cancer clinical questions. Please use BOTH your comprehensive medical knowledge AND web search to provide the best evidence-based answers.

CRITICAL TIME RESTRICTION:
- Only use evidence published up to FEBRUARY 28, 2025
- EXCLUDE the St. Gallen 2025 International Consensus Conference
- Any evidence or guidelines after February 2025 should NOT be used

APPROACH FOR EACH QUESTION:
1. FIRST: Use your extensive static knowledge of:
   - Established clinical trials (ACOSOG Z0011, AMAROS, TAILORx, RxPONDER, KEYNOTE-522, etc.)
   - Standard guidelines and their historical recommendations
   - Fundamental oncology principles and standard of care
   - Well-established evidence through your knowledge cutoff

2. THEN: Use WEB SEARCH to:
   - Find recent updates ONLY through February 2025
   - Verify guideline versions (up to Feb 2025)
   - Check for trial results published before March 2025
   - Find consensus statements (excluding St. Gallen 2025)
   - Confirm if established recommendations have changed

3. SYNTHESIZE: Combine both sources for the most complete answer:
   - Base your reasoning on established evidence
   - Update with findings from web search (through Feb 2025 only)
   - Note if web search confirms or modifies established knowledge

IMPORTANT:
- Use BOTH static knowledge AND web search for comprehensive analysis
- STRICTLY EXCLUDE: St. Gallen 2025 conference and any evidence after Feb 28, 2025
- Your static knowledge provides the foundation
- Web search provides updates and verification (through Feb 2025)
- Cite both established studies and recent updates
- Be explicit about what comes from your knowledge vs. web search

For EACH question below, provide your answer in EXACTLY this format:

QUESTION_ID: [the question ID]
SELECTED_ANSWER: [single letter A-G only]
EVIDENCE_SUMMARY: [2-3 sentences combining established evidence and recent updates through Feb 2025]
KEY_REFERENCES: [List 2-3 most important studies - both landmark trials and recent updates through Feb 2025]
GUIDELINE_ALIGNMENT: [Which guidelines support this - versions through Feb 2025 only]
SUBGROUP_NOTES: [Any relevant patient factors that might modify recommendation]
CONFIDENCE_LEVEL: [High/Moderate/Low based on evidence quality and consistency]
WEB_SEARCH_USED: [Yes - list what you searched for and what updates

through Feb 2025 you found]
---END---

Now analyze ALL 9 questions from FollowUp_Survival:

=== QUESTION ID: 73 ===

QUESTION: Given the natural history of ER positive, HER2 negative early stage breast cancer and ductal carcinoma in situ, the appropriate duration of follow-up on clinical therapy trials for women with ER+ tumors before recommending new treatment approaches is, in the absence of overall survival benefit, how long?

OPTIONS:
A: 2 years
B: 3 years
C: 5 years
D: 10 years

=== QUESTION ID: 74 ===

QUESTION: Breast cancer patients should routinely be asked about sexual health in regular follow ups

OPTIONS:
A: Agree
B: Disagree

=== QUESTION ID: 75 ===

QUESTION: The appropriate duration of follow-up by a cancer specialist for women with early stage breast cancer is

OPTIONS:
A: 1 year
B: 3 years
C: 5 years
D: 10 years
E: Indefinite

=== QUESTION ID: 76 ===

QUESTION: A 76-year-old woman with 1 cm breast cancer, grade 2, ER positive, Prpositive, and HER2 negative. Her axilla is negative on ultrasound, and she will not require sentinel lymph node surgery. She would prefer to avoid surgery if reasonable for long term cancer outcomes. You would recommend:

OPTIONS:
A: Neoadjuvant endocrine therapy with an AI and reassessment

surgical options in 6 months
B: Stereotactic radiation therapy
C: Upfront surgery
D: Cryo- or radiofrequency ablation therapy to the breast tumor

=== QUESTION ID: 77 ===

QUESTION: A patient with breast conserving surgery, adjuvant AC/T chemotherapy, and radiation therapy for T1cN0 triple negative breast cancer. Three years later she has a local recurrence in the breast with similar histology, measuring ~1.5 cm. You would recommend:

OPTIONS:
A: Mastectomy
B: Lumpectomy with re-irradiation
C: Mastectomy followed by adjuvant TCb chemotherapy
D: Neoadjuvant TCb chemotherapy
E: Neoadjuvant TCb chemotherapy and pembrolizumab

=== QUESTION ID: 78 ===

QUESTION: A patient with history of stage 2, ER positive, PR positive, HER2 negative breast cancer treated with breast conserving surgery, radiation therapy, and adjuvant AI. Six years after initial treatment, local recurrence in the breast. ER+, PR low, HER2-, grade 2. Do you believe genomic risk signature testing is informative for the benefits of chemotherapy in women who have had local-regional tumor recurrence on adjuvant endocrine therapy?

OPTIONS:
A: Yes
B: No

=== QUESTION ID: 79 ===

QUESTION: A 53-year-old woman diagnosed with T3Nx triple negative breast cancer. Staging scans identify isolated liver lesion, which is biopsied and shown to be metastatic cancer. Testing of the breast primary is PD-L1 negative. She receives induction TCb/AC chemotherapy with concurrent pembrolizumab, and has substantial clinical response. Repeat imaging shows resolution of the liver lesion. Mastectomy and sentinel lymph node surgery showing residual 1.3 cm grade 3 TNBC in breast. You would recommend:

OPTIONS:
A: Ongoing pembrolizumab
B: Ongoing pembrolizumab and radiation therapy
C: Ongoing pembrolizumab, radiation therapy, and capecitabine
D: Observation

=== QUESTION ID: 80 ===

QUESTION: A 63 year old woman has been diagnosed with ER positive, PR positive, HER2 negative breast cancer. She undergoes breast conserving surgery and sentinel lymph node surgery. She has 2.8 cm of grade 2 cancer in the breast, with 3 of 3 axillary lymph nodes positive for metastatic breast cancer. An OncotypeDX recurrence score is sent, and is 28. Staging scans show two areas suspicious for metastatic disease, one in the lumbar spine, and another in a rib. The vertebral body lesion is biopsied, and positive for metastatic carcinoma. You would recommend the following:

OPTIONS:
A: Initiate of AI and CDK4/6i for metastatic breast cancer
B: Adjuvant chemotherapy followed by AI and CDK4/6i
C: Adjuvant chemotherapy followed by radiation therapy to the breast and regional nodes as indicated, followed by AI and CDK4/6i

=== QUESTION ID: 81 ===

QUESTION: In general for a patient with very limited metastatic disease and highly effective treatment options and/or good initial response to therapy one should strongly consider definitive local regional treatment.

OPTIONS:
A: Agree
B: Disagree

CRITICAL REMINDERS:
1. START with your comprehensive static medical knowledge
2. ENHANCE with web search for updates ONLY through February 28, 2025
3. EXCLUDE St. Gallen 2025 conference findings
4. SYNTHESIZE both sources for the most complete, current answer (up to Feb 2025)
5. Format each response exactly as specified above with ---END--- after each question
6. Answer ALL questions above in order
