# Supplementary material for: Performance of large language models in ambiguous treatment scenarios of early breast cancer
Source: Breast. 2026 Sep 23;90:104932. doi: 10.1016/j.breast.2026.104932 (PMC13641045; doi:10.1016/j.breast.2026.104932)
Supplement: Supplementary file 1 [file mmc1.zip › Suppl.Mat. - Anton Oseledchyk - Prompts, LLM Responses, R-Code/Prompt_Radiation_Therapy_questions.pdf]

You are an expert physician analyzing breast cancer clinical questions. Please use BOTH your comprehensive medical knowledge AND web search to provide the best evidence-based answers.

CRITICAL TIME RESTRICTION:
- Only use evidence published up to FEBRUARY 28, 2025
- EXCLUDE the St. Gallen 2025 International Consensus Conference
- Any evidence or guidelines after February 2025 should NOT be used

APPROACH FOR EACH QUESTION:
1. FIRST: Use your extensive static knowledge of:
   - Established clinical trials (ACOSOG Z0011, AMAROS, TAILORx, RxPONDER, KEYNOTE-522, etc.)
   - Standard guidelines and their historical recommendations
   - Fundamental oncology principles and standard of care
   - Well-established evidence through your knowledge cutoff

2. THEN: Use WEB SEARCH to:
   - Find recent updates ONLY through February 2025
   - Verify guideline versions (up to Feb 2025)
   - Check for trial results published before March 2025
   - Find consensus statements (excluding St. Gallen 2025)
   - Confirm if established recommendations have changed

3. SYNTHESIZE: Combine both sources for the most complete answer:
   - Base your reasoning on established evidence
   - Update with findings from web search (through Feb 2025 only)
   - Note if web search confirms or modifies established knowledge

IMPORTANT:
- Use BOTH static knowledge AND web search for comprehensive analysis
- STRICTLY EXCLUDE: St. Gallen 2025 conference and any evidence after Feb 28, 2025
- Your static knowledge provides the foundation
- Web search provides updates and verification (through Feb 2025)
- Cite both established studies and recent updates
- Be explicit about what comes from your knowledge vs. web search

For EACH question below, provide your answer in EXACTLY this format:

QUESTION_ID: [the question ID]
SELECTED_ANSWER: [single letter A-G only]
EVIDENCE_SUMMARY: [2-3 sentences combining established evidence and recent updates through Feb 2025]
KEY_REFERENCES: [List 2-3 most important studies - both landmark trials and recent updates through Feb 2025]
GUIDELINE_ALIGNMENT: [Which guidelines support this - versions through Feb 2025 only]
SUBGROUP_NOTES: [Any relevant patient factors that might modify recommendation]
CONFIDENCE_LEVEL: [High/Moderate/Low based on evidence quality and consistency]
WEB_SEARCH_USED: [Yes - list what you searched for and what updates

through Feb 2025 you found]
---END---

Now analyze ALL 9 questions from Radiation_Therapy:

=== QUESTION ID: 16 ===

QUESTION: A 66 year old woman has undergone mastectomy for a grade 2, ER positive, HER2 negative breast cancer. The tumor measures 1.6 cm, and 1 of 3 sentinel lymph nodes are positive. In addition to systemic therapy, what radiation therapy would you recommend?

OPTIONS:
A: No radiation Therapy
B: Chest wall irradiation only
C: Chest wall and regional nodal irradiation

=== QUESTION ID: 17 ===

QUESTION: A 41 year old warnen has undergone mastectomy for a grade 2, ER positive, HER2 negative breast cancer. The tumor measures 1.6 cm, and 1 of 3 sentinel lymph nodes are positive and the genomic score is low to intermediate risk. In addition to systemic therapy, what radiation therapy would you recommend?

OPTIONS:
A: No radiation Therapy
B: Chest wall irradiation only
C: Chest wall and regional nodal irradiation

=== QUESTION ID: 18 ===

QUESTION: You are treating a patient who has had neoadjuvant chemotherapy, mastectomy, im plant reconstruction, and axillary lymph node dissection for stage 3 breast cancer, stage T3N2, with metastatic involvement of 8 of 11 axillary lym,ph nodes. She will receive post-mastectomy radiation therapy including regiona l nodal irradiation. You would recommend which treatment schedule?

OPTIONS:
A: Conventional fractionation with 25 fractions
B: Moderate hypofractionation with 15-16 fraction

=== QUESTION ID: 19 ===

QUESTION: A 52 year old postmenopausal woman has undergone BCS for a stage 1 brea·st cancer. The tumor is T1 c, with negative sentinel lymph nodes, a1nd was grade 1, ER positive> 95°/4, PR positive> 95%, and HER2 negative. She will receive adju;vant endocrine therapy. What radiation therapy schedule would you recommend?

OPTIONS:
A: No radiation Therapy
B: Conventional fractionation with 25 fractions
C: Moderate hypofractionation with 15-16 fraction
D: Ultra-hypofractionation whole breast irradiation (i .. e. 5 days of consecutive treatment, a la FAST-FORWARD)
E: Partial breast irradiation (5-lSFx/IORT/Brachytherapy)

=== QUESTION ID: 20 ===

QUESTION: A vigorous 69 year old postmenopausal woman has had BCS for a T1 c, node-negative tumor that, was grade 2, strongly ER and PR positive, and HER2 negative. Her mother lived to age 86, and she expects to do the same. She will receive adjuvant endocrine therapy. What radiation therapy schedule would you recommend?

OPTIONS:
A: No radiation Therapy
B: Conventional fractionation with 25 fractions
C: Moderate hypofractionation with 15-16 fraction
D: Ultra-hypofractionation whole breast irradiation (i .. e. 5 days of consecutive treatment, a la FAST-FORWARD)
E: Partial breast irradiation (5-lSFx/IORT/Brachytherapy)

=== QUESTION ID: 21 ===

QUESTION: For a postmenopausat woman in her 60s who has a Tl c cancer with low risk features, and a negative ax1llary ultrasound. You would recommend which of the following?

OPTIONS:
A: whole breast irradiation
B: Partial breast irradiation
C: Sentinel lymph node biopsy, then partial breast irradiation, if negative

=== QUESTION ID: 22 ===

QUESTION: A 70 year old woman has undergone breast conserving surgery for a 1.3 cm, grade 1-2 of 3, and strongly ER positive, PR positive breast cancer, and HER2 0 by IHC. You would recommend:

OPTIONS:
A: No further therapy
B: Radiation therapy
C: Endocrine therapy
D: Radiation therapy and endocrine therapy

=== QUESTION ID: 23 ===

QUESTION: In a patient with a clinically negative axilla, the size threshold for initiating neoadjuvant TCb/AC/pembrolizumab in newly diagnosed TNBC is:

OPTIONS:
A: 1.0 cm
B: 1.5 cm
C: 2.0 cm
D: 2.5 cm
E: 3.0 cm
F: greater than 3.0 cm

=== QUESTION ID: 24 ===

QUESTION: A healthy young woman presented with a small breast nodule, and was diagnosed with high-grade, triple negative breast cancer. On MRI and US imaging, the tumor measures 1.7489753cm in size, and the axilla is clinically negative. Would you recommend neoadjuvant chemotherapy in combination with checkpoint inhibitor immunotherapy?

OPTIONS:
A: Yes
B: No

CRITICAL REMINDERS:
1. START with your comprehensive static medical knowledge
2. ENHANCE with web search for updates ONLY through February 28, 2025
3. EXCLUDE St. Gallen 2025 conference findings
4. SYNTHESIZE both sources for the most complete, current answer (up to Feb 2025)
5. Format each response exactly as specified above with ---END--- after each question
6. Answer ALL questions above in order
