# Supplementary material for: Performance of large language models in ambiguous treatment scenarios of early breast cancer
Source: Breast. 2026 Sep 23;90:104932. doi: 10.1016/j.breast.2026.104932 (PMC13641045; doi:10.1016/j.breast.2026.104932)
Supplement: Supplementary file 1 [file mmc1.zip › Suppl.Mat. - Anton Oseledchyk - Prompts, LLM Responses, R-Code/Prompt_Systemic_Her2pos_questions.pdf]

You are an expert physician analyzing breast cancer clinical questions. Please use BOTH your comprehensive medical knowledge AND web search to provide the best evidence-based answers.

CRITICAL TIME RESTRICTION:
- Only use evidence published up to FEBRUARY 28, 2025
- EXCLUDE the St. Gallen 2025 International Consensus Conference
- Any evidence or guidelines after February 2025 should NOT be used

APPROACH FOR EACH QUESTION:
1. FIRST: Use your extensive static knowledge of:
   - Established clinical trials (ACOSOG Z0011, AMAROS, TAILORx, RxPONDER, KEYNOTE-522, etc.)
   - Standard guidelines and their historical recommendations
   - Fundamental oncology principles and standard of care
   - Well-established evidence through your knowledge cutoff

2. THEN: Use WEB SEARCH to:
   - Find recent updates ONLY through February 2025
   - Verify guideline versions (up to Feb 2025)
   - Check for trial results published before March 2025
   - Find consensus statements (excluding St. Gallen 2025)
   - Confirm if established recommendations have changed

3. SYNTHESIZE: Combine both sources for the most complete answer:
   - Base your reasoning on established evidence
   - Update with findings from web search (through Feb 2025 only)
   - Note if web search confirms or modifies established knowledge

IMPORTANT:
- Use BOTH static knowledge AND web search for comprehensive analysis
- STRICTLY EXCLUDE: St. Gallen 2025 conference and any evidence after Feb 28, 2025
- Your static knowledge provides the foundation
- Web search provides updates and verification (through Feb 2025)
- Cite both established studies and recent updates
- Be explicit about what comes from your knowledge vs. web search

For EACH question below, provide your answer in EXACTLY this format:

QUESTION_ID: [the question ID]
SELECTED_ANSWER: [single letter A-G only]
EVIDENCE_SUMMARY: [2-3 sentences combining established evidence and recent updates through Feb 2025]
KEY_REFERENCES: [List 2-3 most important studies - both landmark trials and recent updates through Feb 2025]
GUIDELINE_ALIGNMENT: [Which guidelines support this - versions through Feb 2025 only]
SUBGROUP_NOTES: [Any relevant patient factors that might modify recommendation]
CONFIDENCE_LEVEL: [High/Moderate/Low based on evidence quality and consistency]
WEB_SEARCH_USED: [Yes - list what you searched for and what updates

through Feb 2025 you found]
---END---

Now analyze ALL 5 questions from Systemic_Her2pos:

=== QUESTION ID: 34 ===

QUESTION: A patient has undergone mastectomy for extensive high-grade DCIS. At the time of surgery she is found to have foci of microinvasive breast cancer that is ER negative and HER2 3+ by IHC. What number of foci of microinvasive cancer would prompt you to recommend adjuvant paclitaxel/trastuzumab therapy?

OPTIONS:
A: 1-2
B: 3-5
C: 5-9
D: 10 or more
E: I would not recommend paclitaxel/trastuzumab for microinvasion

=== QUESTION ID: 35 ===

QUESTION: A patient has a newly diagnosed breast cancer that is ER positive and HER2 positive. She undergoes surgery, with final pathology showing a 2.4 cm tumor, with negative lymph nodes. As adjuvant chemotherapy including HER2-blockade, you would recommend:

OPTIONS:
A: TH
B: TH + Pertuzumab
C: TCbH
D: TCbH + Pertuzumab

=== QUESTION ID: 36 ===

QUESTION: A patient received neoadjuvant TCHP for stage 2, node-positive, ER negative, HER2 positive (3+) breast cancer. At surgery, she is found to have residual tumor in the breast and lymph nodes. Repeat HER2 testing indicates that the tumor is HER2 2+ by IHC, and negative by FISH (HER2/neu copy number, 3.9, ratio 1.9). As adjuvant treatment, you would recommend:

OPTIONS:
A: No further systemic therapy
B: Adjuvant AC or EC chemotherapy
C: Trastuzumab emtansine
D: Trastuzumab deruxtecan

=== QUESTION ID: 37 ===

QUESTION: A patient has a 3cm estrogen receptor negative HER2+ breast cancer. The HER2DX test shows low risk and high pCR score. You would recommend:

OPTIONS:
A: Surgery, then adjuvant paclitaxel/trastuzumab
B: Neoadjuvant paclitaxel/trastuzumab
C: Neoadjuvant THP
D: Neoadjuvant TCbHP
E: Would not have used HER2DX

=== QUESTION ID: 38 ===

QUESTION: In routine treatment of stage 2 or 3, HER2 positive breast cancer, the preferred regimen is:

OPTIONS:
A: TCbHP
B: TCbHP and then AC
C: TCbHP and then AC with HP

CRITICAL REMINDERS:
1. START with your comprehensive static medical knowledge
2. ENHANCE with web search for updates ONLY through February 28, 2025
3. EXCLUDE St. Gallen 2025 conference findings
4. SYNTHESIZE both sources for the most complete, current answer (up to Feb 2025)
5. Format each response exactly as specified above with ---END--- after each question
6. Answer ALL questions above in order
