# Supplementary material for: Performance of large language models in ambiguous treatment scenarios of early breast cancer
Source: Breast. 2026 Sep 23;90:104932. doi: 10.1016/j.breast.2026.104932 (PMC13641045; doi:10.1016/j.breast.2026.104932)
Supplement: Supplementary file 1 [file mmc1.zip › Suppl.Mat. - Anton Oseledchyk - Prompts, LLM Responses, R-Code/Prompt_Systemic_HRpos_questions.pdf]

You are an expert physician analyzing breast cancer clinical questions. Please use BOTH your comprehensive medical knowledge AND web search to provide the best evidence-based answers.

CRITICAL TIME RESTRICTION:
- Only use evidence published up to FEBRUARY 28, 2025
- EXCLUDE the St. Gallen 2025 International Consensus Conference
- Any evidence or guidelines after February 2025 should NOT be used

APPROACH FOR EACH QUESTION:
1. FIRST: Use your extensive static knowledge of:
   - Established clinical trials (ACOSOG Z0011, AMAROS, TAILORx, RxPONDER, KEYNOTE-522, etc.)
   - Standard guidelines and their historical recommendations
   - Fundamental oncology principles and standard of care
   - Well-established evidence through your knowledge cutoff

2. THEN: Use WEB SEARCH to:
   - Find recent updates ONLY through February 2025
   - Verify guideline versions (up to Feb 2025)
   - Check for trial results published before March 2025
   - Find consensus statements (excluding St. Gallen 2025)
   - Confirm if established recommendations have changed

3. SYNTHESIZE: Combine both sources for the most complete answer:
   - Base your reasoning on established evidence
   - Update with findings from web search (through Feb 2025 only)
   - Note if web search confirms or modifies established knowledge

IMPORTANT:
- Use BOTH static knowledge AND web search for comprehensive analysis
- STRICTLY EXCLUDE: St. Gallen 2025 conference and any evidence after Feb 28, 2025
- Your static knowledge provides the foundation
- Web search provides updates and verification (through Feb 2025)
- Cite both established studies and recent updates
- Be explicit about what comes from your knowledge vs. web search

For EACH question below, provide your answer in EXACTLY this format:

QUESTION_ID: [the question ID]
SELECTED_ANSWER: [single letter A-G only]
EVIDENCE_SUMMARY: [2-3 sentences combining established evidence and recent updates through Feb 2025]
KEY_REFERENCES: [List 2-3 most important studies - both landmark trials and recent updates through Feb 2025]
GUIDELINE_ALIGNMENT: [Which guidelines support this - versions through Feb 2025 only]
SUBGROUP_NOTES: [Any relevant patient factors that might modify recommendation]
CONFIDENCE_LEVEL: [High/Moderate/Low based on evidence quality and consistency]
WEB_SEARCH_USED: [Yes - list what you searched for and what updates

through Feb 2025 you found]
---END---

Now analyze ALL 33 questions from Systemic_HRpos:

=== QUESTION ID: 39 ===

QUESTION: In discussing adjuvant chemotherapy in ER positive breast cancer, you would in general recommend chemotherapy if the likely benefit on distant-recurrence free survival is:

OPTIONS:
A: 10% or more
B: 5% or more
C: 3% or more
D: 1% or more

=== QUESTION ID: 40 ===

QUESTION: Based on historical outcomes of studies such as NSABP B-21 for tumors 1 cm or smaller, node-negative, ER positive, and HER2 negative, the risk of distant metastatic recurrence with endocrine therapy is sufficiently low that genomic risk assays are unnecessary.

OPTIONS:
A: True
B: False

=== QUESTION ID: 42 ===

QUESTION: A 63 year old woman presents with clinical T3N1 breast cancer that is grade 1, has ductal and lobular histology, and is strongly ER positive and PR positive. As her next treatment, you would recommend:

OPTIONS:
A: Neoadjuvant chemotherapy
B: Neoadjuvant endocrine therapy
C: Order genomic signature
D: Neoadjuvant endocrine therapy and CDK4/6 inhibitor
E: Proceed to mastectomy as systemic treatment is unlikely to affect surgical options

=== QUESTION ID: 43 ===

QUESTION: A 63 year old woman presents with clinical T3N1 breast cancer that is grade 1, has ductal and lobular histology, and is strongly ER positive and PR positive with a low risk genomic score. As her next treatment, you would recommend:

OPTIONS:
A: Neoadjuvant chemotherapy
B: Neoadjuvant endocrine therapy
C: Neoadjuvant endocrine therapy and CDK4/6 inhibitor
D: Proceed to mastectomy as systemic treatment is unlikely to affect surgical options

=== QUESTION ID: 44 ===

QUESTION: A 63 year old woman presents with clinical T3N1 breast cancer that is grade 1, has ductal and lobular histology, and is strongly ER positive and PR positive with a low risk genomic score. She receives 6 months of neoadjuvant endocrine treatment with substantial clinical response. Following surgery she is found to have residual tumor in the breast cancer and in 3 of 11 axillary lymph nodes. In addition to optimal endocrine therapy, would you now recommend adjuvant chemotherapy?

OPTIONS:
A: Yes
B: No

=== QUESTION ID: 45 ===

QUESTION: The recommended duration of endocrine therapy for a patient with a HER2 negative breast cancer of 1.8 cm, grade 2 is: for ER positive, with 1 of 4 sentinel lymph nodes positive.

OPTIONS:
A: 5 years
B: 7 to 8 years
C: 10 years

=== QUESTION ID: 46 ===

QUESTION: The recommended duration of endocrine therapy for a patient with a HER2 negative breast cancer of 1.8 cm, grade 2 is: for node-negative ER positive, with an OncotypeDX recurrence score of 28 (or MammaPrint 'high' score)

OPTIONS:
A: 5 years
B: 7 to 8 years
C: 10 years

=== QUESTION ID: 47 ===

QUESTION: The recommended duration of endocrine therapy for a patient with a HER2 negative breast cancer of 1.8 cm, grade 2 is:

for ER positive, with 3 of 8 axillary lymph nodes positive

OPTIONS:
A: 5 years
B: 7 to 8 years
C: 10 years

=== QUESTION ID: 48 ===

QUESTION: A Postmenopausal woman with T2N0 ER+, PR+, HER2- breast cancer, 2.4 cm, grade 2, OncotypeDX recurrence score of 28 (or MammaPrint 'High 1') result. As adjuvant chemotherapy, you would recommend:

OPTIONS:
A: AC/T
B: TC

=== QUESTION ID: 49 ===

QUESTION: A postmenopausal woman with T2N0 ER positive, PR positive, HER2 negative breast cancer, 2.4 cm, grade 2, OncotypeDX recurrence score of 32 (or MammaPrint High 2) result. As adjuvant chemotherapy, you would recommend:

OPTIONS:
A: AC/T
B: TC

=== QUESTION ID: 50 ===

QUESTION: A Postmenopausal woman with T2 N0 ER positive, PR positive, HER2 negative breast cancer, 1.8 cm, grade 3, OncotypeDX recurrence score of 32 (or MammaPrint High 2) result. As adjuvant chemotherapy, you would recommend:

OPTIONS:
A: AC/T
B: TC

=== QUESTION ID: 51 ===

QUESTION: In a 60-year-old postmenopausal woman with T1cN1 ER+, PR+, HER2- breast cancer affecting 3 of 11 lymph nodes, OncotypeDX score of 13. In addition to adjuvant endocrine treatments, you would recommend:

OPTIONS:
A: No chemotherapy
B: TC chemotherapy

C: AC/T chemotherapy

=== QUESTION ID: 52 ===

QUESTION: In 60-year-old postmenopausal woman with T1cN2 ER+, PR+, HER2- breast cancer affecting 4 of 11 lymph nodes, OncotypeDX score of 13. In addition to adjuvant endocrine treatments, you would recommend:

OPTIONS:
A: No chemotherapy
B: TC chemotherapy
C: AC/T chemotherapy

=== QUESTION ID: 53 ===

QUESTION: A premenopausal 42 year old woman has been diagnosed with an ER positive, HER2 negative 1.4 cm,grade 2, node-negative breast cancer. As adjuvant therapy, you would recommend: if OncotypeDX score is 12

OPTIONS:
A: Tamoxifen
B: OFS + tamoxifen
C: OFS + AI
D: Chemotherapy followed by endocrine therapy

=== QUESTION ID: 54 ===

QUESTION: A premenopausal 42 year old woman has been diagnosed with an ER positive, HER2 negative 1.4 cm,grade 2, node-negative breast cancer. As adjuvant therapy, you would recommend: if OncotypeDX score is 18

OPTIONS:
A: Tamoxifen
B: OFS + tamoxifen
C: OFS + AI
D: Chemotherapy followed by endocrine therapy

=== QUESTION ID: 55 ===

QUESTION: A premenopausal 42 year old woman has been diagnosed with an ER positive, HER2 negative 1.4 cm,grade 2, node-negative breast cancer. As adjuvant therapy, you would recommend: if OncotypeDX score is 23

OPTIONS:
A: Tamoxifen
B: OFS + tamoxifen

C: OFS + AI
D: Chemotherapy followed by endocrine therapy

=== QUESTION ID: 56 ===

QUESTION: A premenopausal 42 year old woman has been diagnosed with an ER positive, HER2 negative 1.4 cm, grade 2, with one positive axillary lymph node. As adjuvant therapy, you would recommend: if OncotypeDX score is 12

OPTIONS:
A: Tamoxifen
B: OFS + tamoxifen
C: OFS + AI
D: Chemotherapy followed by endocrine therapy

=== QUESTION ID: 57 ===

QUESTION: A premenopausal 42 year old woman has been diagnosed with an ER positive, HER2 negative 1.4 cm, grade 2, with one positive axillary lymph node. As adjuvant therapy, you would recommend: if OncotypeDX score is 18

OPTIONS:
A: Tamoxifen
B: OFS + tamoxifen
C: OFS + AI
D: Chemotherapy followed by endocrine therapy

=== QUESTION ID: 58 ===

QUESTION: A premenopausal 42 year old woman has been diagnosed with an ER positive, HER2 negative 1.4 cm, grade 2, with one positive axillary lymph node. As adjuvant therapy, you would recommend: if OncotypeDX score is 23

OPTIONS:
A: Tamoxifen
B: OFS + tamoxifen
C: OFS + AI
D: Chemotherapy followed by endocrine therapy

=== QUESTION ID: 59 ===

QUESTION: A premenopausal 42-year-old woman has been diagnosed with a 1.4 cm, grade 2 tumor. As adjuvant treatment, you would recommend: in case of two positive axillary lymph nodes, OncotypeDX score is 12

OPTIONS:
A: Tamoxifen

B: OFS + ET
C: OFS + ET + CDK46 inhibitor
D: Chemotherapy followed by endocrine therapy
E: Chemotherapy followed by ET plus a CDK46 inhibitor

=== QUESTION ID: 60 ===

QUESTION: A premenopausal 42-year-old woman has been diagnosed with a 1.4 cm, grade 2 tumor. As adjuvant treatment, you would recommend: in case of two positive axillary lymph nodes, OncotypeDX score is 18

OPTIONS:
A: Tamoxifen
B: OFS + ET
C: OFS + ET + CDK46 inhibitor
D: Chemotherapy followed by endocrine therapy
E: Chemotherapy followed by ET plus a CDK46 inhibitor

=== QUESTION ID: 61 ===

QUESTION: A premenopausal 42-year-old woman has been diagnosed with a 1.4 cm, grade 2 tumor. As adjuvant treatment, you would recommend: in case of one positive axillary lymph node, OncotypeDX score is 23

OPTIONS:
A: Tamoxifen
B: OFS + ET
C: OFS + ET + CDK46 inhibitor
D: Chemotherapy followed by endocrine therapy
E: Chemotherapy followed by ET plus a CDK46 inhibitor

=== QUESTION ID: 62 ===

QUESTION: A premenopausal 42-year-old woman has been diagnosed with a 1.4 cm, grade 2 tumor. As adjuvant treatment, you would recommend: in case of one positive axillary lymph node, Ki67 is 30%

OPTIONS:
A: Tamoxifen
B: OFS + ET
C: OFS + ET + CDK46 inhibitor
D: Chemotherapy followed by endocrine therapy
E: Chemotherapy followed by ET plus a CDK46 inhibitor

=== QUESTION ID: 63 ===

QUESTION: A 53-year-old woman received neoadjuvant chemotherapy for grade 2 breast cancer with T2N1 clinical presentation. She had a major clinical response. At surgery, she had a residual 1.0 cm tumor in breast. In addition to adjuvant endocrine therapy, would you

recommend adjuvant CDK4/6 inhibitor therapy? In case of 3 negative sentinel lymph nodes

OPTIONS:
A: Yes
B: No

=== QUESTION ID: 64 ===

QUESTION: A 53-year-old woman received neoadjuvant chemotherapy for grade 2 breast cancer with T2N1 clinical presentation. She had a major clinical response. At surgery, she had a residual 1.0 cm tumor in breast. In addition to adjuvant endocrine therapy, would you recommend adjuvant CDK4/6 inhibitor therapy? In case of 1 of 3 sentinel lymph nodes positive

OPTIONS:
A: Yes
B: No

=== QUESTION ID: 65 ===

QUESTION: A 57-year-old postmenopausal woman has been diagnosed with 2.1 cm, node-negative, grade 2, ER positive breast cancer, OncotypeDX score of 25. As adjuvant treatment, you would recommend?

OPTIONS:
A: AI
B: Chemotherapy then AI
C: AI + CDK4/6i
D: Chemotherapy then AI + CDK4i

=== QUESTION ID: 66 ===

QUESTION: A postmenopausal patient has been diagnosed with a 2.1 cm, ER positive, grade 2 breast cancer. What adjuvant therapy would you recommend? In case of node negative, OncotypeDX score of 26

OPTIONS:
A: AI
B: Chemotherapy then AI
C: AI + CDK4/6i
D: Chemotherapy then AI + CDK4/6i

=== QUESTION ID: 67 ===

QUESTION: A postmenopausal patient has been diagnosed with a 2.1 cm, ER positive, grade 2 breast cancer. What adjuvant therapy would you recommend? In case of node negative, Ki67 of 26%

OPTIONS:
A: AI
B: Chemotherapy then AI
C: AI + CDK4/6i
D: Chemotherapy then AI + CDK4/6i

=== QUESTION ID: 68 ===

QUESTION: A postmenopausal patient has been diagnosed with a 2.1 cm, ER positive, grade 2 breast cancer. What adjuvant therapy would you recommend? In case of 1 of 3 SLN positive, OncotypeDX score of 18

OPTIONS:
A: AI
B: Chemotherapy then AI
C: AI + CDK4/6i
D: Chemotherapy then AI + CDK4/6i

=== QUESTION ID: 69 ===

QUESTION: AdjuvanA postmenopausal patient has been diagnosed with a 2.1 cm, ER positive, grade 2 breast cancer. What adjuvant therapy would you recommend? In case of 1 of 3 SLN positive, Ki67 of 18%

OPTIONS:
A: AI
B: Chemotherapy then AI
C: AI + CDK4/6i
D: Chemotherapy then AI + CDK4/6i

=== QUESTION ID: 70 ===

QUESTION: A postmenopausal patient has been diagnosed with a 2.1 cm, ER positive, grade 2 breast cancer. What adjuvant therapy would you recommend? In case of 2 of 4 SLN positive, OncotypeDX score of 11

OPTIONS:
A: AI
B: Chemotherapy then AI
C: AI + CDK4/6i
D: Chemotherapy then AI + CDK4/6i

=== QUESTION ID: 71 ===

QUESTION: A postmenopausal patient has been diagnosed with a 2.1 cm, ER positive, grade 2 breast cancer. What adjuvant therapy would you recommend? In case of 2 of 4 SLN positive, Ki67 of 11%

OPTIONS:
A: AI

B: Chemotherapy then AI
C: AI + CDK4/6i
D: Chemotherapy then AI + CDK4/6i

=== QUESTION ID: 72 ===

QUESTION: Men with ER+ breast cancer should be offered CDK4/6 inhibitor treatment based on the same criteria as women with early stage breast cancer.

OPTIONS:
A: True
B: False

CRITICAL REMINDERS:
1. START with your comprehensive static medical knowledge
2. ENHANCE with web search for updates ONLY through February 28, 2025
3. EXCLUDE St. Gallen 2025 conference findings
4. SYNTHESIZE both sources for the most complete, current answer (up to Feb 2025)
5. Format each response exactly as specified above with ---END--- after each question
6. Answer ALL questions above in order
