# Supplementary material for: Performance of large language models in ambiguous treatment scenarios of early breast cancer
Source: Breast. 2026 Sep 23;90:104932. doi: 10.1016/j.breast.2026.104932 (PMC13641045; doi:10.1016/j.breast.2026.104932)
Supplement: Supplementary file 1 [file mmc1.zip › Suppl.Mat. - Anton Oseledchyk - Prompts, LLM Responses, R-Code/Prompt_Systemic_TNBC_questions.pdf]

You are an expert physician analyzing breast cancer clinical questions. Please use BOTH your comprehensive medical knowledge AND web search to provide the best evidence-based answers.

CRITICAL TIME RESTRICTION:
- Only use evidence published up to FEBRUARY 28, 2025
- EXCLUDE the St. Gallen 2025 International Consensus Conference
- Any evidence or guidelines after February 2025 should NOT be used

APPROACH FOR EACH QUESTION:
1. FIRST: Use your extensive static knowledge of:
   - Established clinical trials (ACOSOG Z0011, AMAROS, TAILORx, RxPONDER, KEYNOTE-522, etc.)
   - Standard guidelines and their historical recommendations
   - Fundamental oncology principles and standard of care
   - Well-established evidence through your knowledge cutoff

2. THEN: Use WEB SEARCH to:
   - Find recent updates ONLY through February 2025
   - Verify guideline versions (up to Feb 2025)
   - Check for trial results published before March 2025
   - Find consensus statements (excluding St. Gallen 2025)
   - Confirm if established recommendations have changed

3. SYNTHESIZE: Combine both sources for the most complete answer:
   - Base your reasoning on established evidence
   - Update with findings from web search (through Feb 2025 only)
   - Note if web search confirms or modifies established knowledge

IMPORTANT:
- Use BOTH static knowledge AND web search for comprehensive analysis
- STRICTLY EXCLUDE: St. Gallen 2025 conference and any evidence after Feb 28, 2025
- Your static knowledge provides the foundation
- Web search provides updates and verification (through Feb 2025)
- Cite both established studies and recent updates
- Be explicit about what comes from your knowledge vs. web search

For EACH question below, provide your answer in EXACTLY this format:

QUESTION_ID: [the question ID]
SELECTED_ANSWER: [single letter A-G only]
EVIDENCE_SUMMARY: [2-3 sentences combining established evidence and recent updates through Feb 2025]
KEY_REFERENCES: [List 2-3 most important studies - both landmark trials and recent updates through Feb 2025]
GUIDELINE_ALIGNMENT: [Which guidelines support this - versions through Feb 2025 only]
SUBGROUP_NOTES: [Any relevant patient factors that might modify recommendation]
CONFIDENCE_LEVEL: [High/Moderate/Low based on evidence quality and consistency]
WEB_SEARCH_USED: [Yes - list what you searched for and what updates

through Feb 2025 you found]
---END---

Now analyze ALL 10 questions from Systemic_TNBC:

=== QUESTION ID: 25 ===

QUESTION: A patient has undergone initial surgery for what was thought to be a stage 1 TNBC. At the time of surgery, she was found to have a 1.1 cm, grade 3 TNBC. What would you recommend as adjuvant therapy? - Metastatic cancer in 1 of 3 axillary lymph nodes

OPTIONS:
A: AC/T
B: TCb/AC
C: TCb/AC/pembrolizumab
D: TC
E: AC/T/avelumab

=== QUESTION ID: 26 ===

QUESTION: A patient has undergone initial surgery for what was thought to be a stage 1 TNBC. At the time of surgery, she was found to have a 1.1 cm, grade 3 TNBC. What would you recommend as adjuvant therapy? - Metastatic cancer in 3 of 5 axillary lymph nodes

OPTIONS:
A: AC/T
B: TCb/AC
C: TCb/AC/pembrolizumab
D: TC
E: AC/T/avelumab

=== QUESTION ID: 27 ===

QUESTION: You are planning to treat a healthy patient with stage 2, node-positive TNBC using the TCb/AC/pembrolizumab regimen. For the chemotherapy portion, you would recommend which of the following:

OPTIONS:
A: Weekly paclitaxel with every 3 week carboplatin, then every 3 week AC
B: Weekly paclitaxel with weekly carboplatin, then every 3 week AC
C: Weekly paclitaxel with every 3 week carboplatin, then every 2 week AC
D: Weekly paclitaxel with weekly carboplatin, then every 2 week AC

=== QUESTION ID: 28 ===

QUESTION: You are treating a patient with stage 2 TNBC. She received

neoadjuvant TCb/AC/durvalumab (a la GeparNuevo), and was found at surgery to have a pathological complete response. In routine practice, you would recommend which of the following:

OPTIONS:
A: Ongoing adjuvant checkpoint inhibition with durvalumab
B: Ongoing adjuvant checkpoint inhibition with pembrolizumab
C: No adjuvant checkpoint inhibitor

=== QUESTION ID: 29 ===

QUESTION: You are treating a patient with stage 2 TNBC. She received neoadjuvant TCb/AC/pembrolizumab (ala KN522) with a good clinical response, but was found at surgery to have residual disease constituting less than 1 cm tumor in the breast with negative lymph nodes. You would recommend which of the following in adjuvant therapy?

OPTIONS:
A: Ongoing pembrolizumab
B: Ongoing pembrolizumab and capecitabine
C: Capecitabine alone

=== QUESTION ID: 30 ===

QUESTION: You are caring for a patient with stage 2, node-positive TNBC, who has medical contraindications to checkpoint inhibitor therapy. As preferred chemotherapy options, you would recommend which of the following?

OPTIONS:
A: AC/T
B: TCb/AC

=== QUESTION ID: 31 ===

QUESTION: You are caring for a 29 year old woman who presents with a 2.5 cm, triple negative breast cancer and a clinically negative axilla on exam and imaging. She is concerned about future fertility. You recommend consulting with a fertility specialist for possible procedures to preserve fertility options, and initiation of GnRH agonist treatment during chemotherapy. In addition, you would advise the following:

OPTIONS:
A: Immunotherapy will not adversely impact her future fertility
B: The impact of immunotherapy on future fertility is not known but you would favor treatment with pembrolizumab
C: The impact of immunotherapy on future fertility is not known and you would not favor treatment with pembrolizumab given her...

=== QUESTION ID: 32 ===

QUESTION: You are treating a patient with stage 2, node-positive TNBC. She additionally has a deleterious BRCA1 or 2 pathogenic variant. She received neoadjuvant TCb/AC/pembrolizumab (ala KN522), and had residual tumor in the lymph nodes. In the adjuvant setting, you would recommend:

OPTIONS:
A: Adjuvant pembrolizumab
B: Adjuvant pembrolizumab and olaparib
C: Adjuvant pembrolizumab, and capecitabine, then olaparib
D: Adjuvant olaparib without pembrolizumab

=== QUESTION ID: 33 ===

QUESTION: A patient has received neoadjuvant TCb/AC for clinical stage 1 TNBC, and achieved a complete pathological response. However, a tumor-specific genomic assay for minimal residual disease drawn after surgery with a blood based, tumor specific genomic era assay is 'positive.' Staging scans are negative. You would recommend:

OPTIONS:
A: Initiate pembrolizumab
B: Initiate pembrolizumab and capecitabine
C: Capecitabine
D: No further therapy

=== QUESTION ID: 41 ===

QUESTION: A 50 year old woman is discussing treatment options for a 3-4 cm, clinically node-negative breast cancer with grade 3 features. The tumor is ER low-positive (5%), PR < 1%, and HER2 negative by IHC. Repeat ER testing confirms the result. As neoadjuvant therapy, you would recommend:

OPTIONS:
A: AC/T
B: TC
C: Pembrolizumab with concurrent taxane then AC chemotherapy

CRITICAL REMINDERS:
1. START with your comprehensive static medical knowledge
2. ENHANCE with web search for updates ONLY through February 28, 2025
3. EXCLUDE St. Gallen 2025 conference findings
4. SYNTHESIZE both sources for the most complete, current answer (up to Feb 2025)

5. Format each response exactly as specified above with ---END---
after each question
6. Answer ALL questions above in order
