# Supplementary material for: Performance of large language models in ambiguous treatment scenarios of early breast cancer
Source: Breast. 2026 Sep 23;90:104932. doi: 10.1016/j.breast.2026.104932 (PMC13641045; doi:10.1016/j.breast.2026.104932)
Supplement: Supplementary file 1 [file mmc1.zip › Suppl.Mat. - Anton Oseledchyk - Prompts, LLM Responses, R-Code/radiation_response_chatgpt.pdf]

QUESTION_ID: 16
SELECTED_ANSWER: C
EVIDENCE_SUMMARY: For pT1–2 disease with 1–3 positive nodes after mastectomy, meta-analysis shows PMRT reduces locoregional recurrence and breast-cancer mortality; contemporary practice includes chest wall plus regional nodes (supraclavicular ± internal mammary) rather than chest wall alone. When nodal disease is identified only on SLN, completion ALND can be omitted if nodal irradiation is delivered, with excellent control (AMAROS) and supported by newer SENOMAC data that included mastectomy patients. ([PubMed][1], [The Lancet][2], [PMC][3], [New England Journal of Medicine][4])
KEY_REFERENCES: EBCTCG PMRT meta-analysis (Lancet 2014); AMAROS trial (EORTC 10981-22023); SENOMAC trial (NEJM 2024). ([PubMed][1], [PMC][3], [New England Journal of Medicine][4])
GUIDELINE_ALIGNMENT: NCCN Breast Cancer 2024 (PMRT strongly considered for 1–3 positive nodes; RNI fields specified); ASCO/ASTRO/SSO PMRT focused update (2016–2017) supports PMRT in 1–3 nodes. ([nursingcenter.com][5], [ASC Publications][6])
SUBGROUP_NOTES: Internal mammary coverage considered for medial/central tumors; patient comorbidity and reconstruction plans may influence technique but not the indication for RNI. ([nursingcenter.com][5])
CONFIDENCE_LEVEL: High
WEB_SEARCH_USED: Yes — searched "EBCTCG PMRT 1–3 nodes," "NCCN 2024 PMRT," "AMAROS axillary radiotherapy," "SENOMAC mastectomy omission ALND"; updates through Feb 2025 confirm ongoing recommendation for PMRT+RNI in N1 disease.
\---END---

QUESTION_ID: 17
SELECTED_ANSWER: C
EVIDENCE_SUMMARY: Node-positive (1 of 3 SLNs) after mastectomy warrants PMRT to chest wall and regional nodes to reduce recurrence and mortality; genomic score does not replace nodal status for PMRT decisions. Evidence supports omission of ALND when RNI is delivered, including for mastectomy cohorts. ([PubMed][1], [The Lancet][2], [PMC][3])
KEY_REFERENCES: EBCTCG PMRT meta-analysis 2014; AMAROS trial; SENOMAC 2024. ([PubMed][1], [PMC][3], [New England Journal of Medicine][4])
GUIDELINE_ALIGNMENT: NCCN 2024; ASCO/ASTRO/SSO PMRT focused update (2016–2017). ([nursingcenter.com][5], [ASC Publications][6])
SUBGROUP_NOTES: Younger age (<45–50) may increase baseline LRR risk, further reinforcing PMRT benefit; genomic assays guide systemic therapy but are not used to omit PMRT in node-positive disease. ([nursingcenter.com][5])
CONFIDENCE_LEVEL: High
WEB_SEARCH_USED: Yes — as above; no changes through Feb 2025 that would remove PMRT for N1 after mastectomy.
\---END---

QUESTION_ID: 18
SELECTED_ANSWER: B
EVIDENCE_SUMMARY: Moderate hypofractionation (e.g., \~40–42.5 Gy in

15–16 fractions) is endorsed for PMRT/RNI, including with reconstruction, showing comparable tumor control and similar complication rates to conventional 25 fractions in randomized and prospective studies. Contemporary guidance documents and randomized data (e.g., FABREC) support hypofractionated PMRT in reconstructed patients. ([PMC][7], [ASCO Post][8], [JAMA Network][9])
KEY_REFERENCES: ASTRO/ASCO WBI hypofractionation guideline framework (2018) applied broadly; FABREC randomized trial (JAMA Oncol 2024 news/coverage); prospective hypofractionated PMRT series (Khan 2017). ([practicalradonc.org][10], [ASCO Post][8], [PMC][11])
GUIDELINE_ALIGNMENT: ASTRO guidance favors hypofractionation where feasible; PMRT public-comment draft (2024) and professional summaries endorse moderate hypofractionation across indications, including with reconstruction. ([astro.org][12])
SUBGROUP_NOTES: Consider chest wall bolus/boost and implant status when planning; immediate implant reconstruction does not preclude moderate hypofractionation. ([ASCO Post][8])
CONFIDENCE_LEVEL: Moderate
WEB_SEARCH_USED: Yes — searched “hypofractionated PMRT reconstruction randomized,” “ASTRO PMRT guideline 2024,” “Khan 11-fraction PMRT”; updates to Feb 2025 support moderate hypofractionation.
\---END---

QUESTION_ID: 19
SELECTED_ANSWER: E
EVIDENCE_SUMMARY: For low-risk stage I (T1cN0), HR-positive disease after lumpectomy with endocrine therapy, partial-breast irradiation (PBI) provides equivalent local control to WBI in appropriately selected patients and reduces treatment volume/toxicity; eligibility includes age ≥50, pT1 (≤2–3 cm), node-negative, ER+. Ultra-hypofractionated WBI (FAST-Forward 26 Gy/5 fx) is also effective, but PBI is preferred here given very favorable features. ([PubMed][13], [PMC][14])
KEY_REFERENCES: ASTRO PBI Clinical Practice Guideline (Practical Radiation Oncology 2024); FAST-Forward RCT (Lancet 2020; HTA 2023). ([PubMed][13], [The Lancet][15])
GUIDELINE_ALIGNMENT: ASTRO 2024 PBI guideline (strong recommendations for suitable patients); NCCN recognizes PBI for selected node-negative early cancers. ([PubMed][13], [nursingcenter.com][5])
SUBGROUP_NOTES: Requires negative margins and pathologic node-negative status (SLNB) before PBI; consider tumor bed location and cosmesis. ([PubMed][13])
CONFIDENCE_LEVEL: High
WEB_SEARCH_USED: Yes — searched “ASTRO 2023/2024 partial breast guideline,” “FAST-Forward 5 fractions”; updates through Feb 2025 reaffirm PBI indications.
\---END---

QUESTION_ID: 20
SELECTED_ANSWER: E
EVIDENCE_SUMMARY: In healthy 69-year-old with T1cN0, HR+/HER2-disease and long life expectancy, adjuvant irradiation is generally

advised; PBI offers oncologic equivalence to WBI for suitable patients with reduced toxicity and greater convenience. Although omission of RT is reasonable in older low-risk cohorts (CALGB 9343, PRIME II), RT (preferably PBI) minimizes ipsilateral recurrence for patients expecting decades of survival. ([PubMed][13], [New England Journal of Medicine][16])

KEY_REFERENCES: ASTRO PBI guideline 2024; CALGB 9343 long-term; PRIME II (NEJM 2023). ([PubMed][13], [New England Journal of Medicine][16])

GUIDELINE_ALIGNMENT: ASTRO 2024 PBI; NCCN recognizes omission of RT in select ≥70 HR+ cases on ET but favors RT to reduce local failure; PBI is a recommended option in suitable patients. ([nursingcenter.com][5], [PubMed][13])

SUBGROUP_NOTES: If significant comorbidities or limited life expectancy, RT could be omitted on ET alone; otherwise, PBI balances control and toxicity. ([PubMed][17], [New England Journal of Medicine][16])

CONFIDENCE_LEVEL: High

WEB_SEARCH_USED: Yes — searched “CALGB 9343 long term,” “PRIME II NEJM 2023,” “ASTRO 2024 PBI”; updates support PBI and clarify omission trade-offs.

\---END---

QUESTION_ID: 21

SELECTED_ANSWER: C

EVIDENCE_SUMMARY: PBI is typically recommended for pathologically node-negative disease; if SLNB is omitted, nodal status is unknown and PBI (which does not treat nodes) may be inappropriate. Trials like SOUND/INSEMA support SLNB omission with negative axillary ultrasound, but PBI guidance still specifies node-negative selection—so performing SLNB and, if pN0, using PBI is most guideline-concordant. ([PubMed][13], [JAMA Network][18])

KEY_REFERENCES: ASTRO PBI guideline 2024 (eligibility emphasizes node-negative selection); SOUND trial (JAMA Oncol 2023) showing non-inferiority of SLNB omission with negative axillary US. ([PubMed][13], [JAMA Network][18])

GUIDELINE_ALIGNMENT: ASTRO 2024 PBI; NCCN allows consideration of SLNB omission in some settings but PBI candidacy assumes node-negative pathology. ([PubMed][13], [nursingcenter.com][5])

SUBGROUP_NOTES: If WBI were planned, omitting SLNB could be reasonable in select low-risk patients per SOUND; for PBI, obtain nodal pathology first. ([JAMA Network][18])

CONFIDENCE_LEVEL: Moderate

WEB_SEARCH_USED: Yes — searched “ASTRO partial breast guideline node-negative,” “SOUND trial SLNB omission”; no Feb 2025 updates changing the need for node-negative status for PBI.

\---END---

QUESTION_ID: 22

SELECTED_ANSWER: D

EVIDENCE_SUMMARY: In women ≥70 with small, node-negative, ER+ tumors, omitting RT while giving endocrine therapy increases local recurrence without affecting survival (CALGB 9343; PRIME II). Given excellent health and standard practice, combining endocrine therapy

with breast irradiation provides the best local control; omission can be individualized but is not the default if longevity is expected. ([PubMed][17], [New England Journal of Medicine][16])
KEY_REFERENCES: CALGB 9343 long-term (JCO 2013); PRIME II 10-year (NEJM 2023). ([PubMed][17], [New England Journal of Medicine][16])
GUIDELINE_ALIGNMENT: NCCN 2024 acknowledges option to omit RT in ≥70 on ET but endorses RT to reduce local failure; ASTRO WBI/PBI guidance supports RT choices for early HR+ disease. ([nursingcenter.com][5])
SUBGROUP_NOTES: Frailty, short life expectancy, or RT contraindications favor ET alone; otherwise, RT+ET maximizes local control. ([PubMed][17])
CONFIDENCE_LEVEL: Moderate
WEB_SEARCH_USED: Yes — searched “CALGB 9343,” “PRIME II NEJM 2023,” “NCCN 2024 omission older women”; updates through Feb 2025 consistent.
\---END---

QUESTION_ID: 23
SELECTED_ANSWER: C
EVIDENCE_SUMMARY: KEYNOTE-522 established neoadjuvant chemo-immunotherapy for stage II–III TNBC: eligibility included cT1c with node-positive disease or cT2–T4 regardless of nodal status—thus for a clinically node-negative axilla, size threshold is ≥2.0 cm (T2). NCCN reflects pembrolizumab-based neoadjuvant therapy for stage II–III TNBC. ([OncLive][19], [New England Journal of Medicine][20], [Keytruda HCP][21])
KEY_REFERENCES: KEYNOTE-522 (NEJM 2020; subsequent analyses); NCCN educational slide set 2024 (TNBC management). ([New England Journal of Medicine][20], [PMC][22], [education.nccn.org][23])
GUIDELINE_ALIGNMENT: NCCN 2024 lists pembrolizumab-containing neoadjuvant regimens for stage II–III TNBC. ([Keytruda HCP][21])
SUBGROUP_NOTES: For cT1cN0 (1.0–2.0 cm), standard is surgery first then adjuvant chemo; neoadjuvant pembro-chemo reserved for stage II–III or node-positive T1c. ([New England Journal of Medicine][20])
CONFIDENCE_LEVEL: High
WEB_SEARCH_USED: Yes — searched “KEYNOTE-522 inclusion criteria,” “NCCN 2024 TNBC pembrolizumab”; updates through Feb 2025 confirm stage II–III criterion.
\---END---

QUESTION_ID: 24
SELECTED_ANSWER: B
EVIDENCE_SUMMARY: For a 1.75 cm (T1c) cN0 TNBC, neoadjuvant pembrolizumab-based therapy is not indicated per KEYNOTE-522 (restricted to stage II–III unless T1c with node-positive disease). Standard care is upfront surgery with adjuvant chemotherapy for tumors ≥1 cm; neoadjuvant chemo-IO would be reserved if nodal disease were present or tumor ≥2 cm. ([OncLive][19], [New England Journal of Medicine][20])
KEY_REFERENCES: KEYNOTE-522 (NEJM 2020; inclusion criteria summarized by experts); NCCN 2024 TNBC management slides. ([OncLive][19], [New England Journal of Medicine][20], [education.nccn.org][23])

GUIDELINE_ALIGNMENT: NCCN 2024 (pembrolizumab Category 1 preferred for stage II–III TNBC; not for stage I cN0). ([Keytruda HCP][21])
SUBGROUP_NOTES: If suspicious nodes appear on imaging or biopsy confirms nodal involvement, neoadjuvant chemo-IO becomes appropriate; germline BRCA may alter adjuvant choices (e.g., olaparib). ([education.nccn.org][23])
CONFIDENCE_LEVEL: High
WEB_SEARCH_USED: Yes — searched “KEYNOTE-522 inclusion,” “NCCN 2024 TNBC pembrolizumab”; updates through Feb 2025 unchanged.
\---END---

[1]: https://pubmed.ncbi.nlm.nih.gov/24656685/?utm_source=chatgpt.com "Effect of radiotherapy after mastectomy and axillary surgery ..."
[2]: https://www.thelancet.com/journals/lancet/article/PIIS0140-6736%2814%2962239-X/fulltext?utm_source=chatgpt.com "Postmastectomy radiotherapy in patients with breast cancer"
[3]: https://pmc.ncbi.nlm.nih.gov/articles/PMC4291166/?utm_source=chatgpt.com "Radiotherapy or surgery of the axilla after a positive ..."
[4]: https://www.nejm.org/doi/abs/10.1056/NEJMoa2313487?utm_source=chatgpt.com "Omitting Axillary Dissection in Breast Cancer with Sentinel- ..."
[5]: https://www.nursingcenter.com/getattachment/Clinical-Resources/Guideline-Summaries/Breast-Cancer/Breast-Cancer-Guidelines-2024-Update.pdf.aspx?utm_source=chatgpt.com "National Comprehensive Cancer Network (NCCN) Clinical ..."
[6]: https://ascopubs.org/doi/10.1200/JCO.2016.69.1188?utm_source=chatgpt.com "Postmastectomy Radiotherapy: An American Society of ..."
[7]: https://pmc.ncbi.nlm.nih.gov/articles/PMC7809517/?utm_source=chatgpt.com "Hypofractionated Postmastectomy Radiation Therapy - PMC"
[8]: https://ascopost.com/issues/november-25-2023/hypofractionation-shows-comparable-results-to-conventional-postmastectomy-radiation-therapy-with-added-benefits/?utm_source=chatgpt.com "Hypofractionation Shows Comparable Results to ..."
[9]: https://jamanetwork.com/journals/jamaoncology/fullarticle/2822041?utm_source=chatgpt.com "Hypofractionated vs Conventionally Fractionated ..."
[10]: https://www.practicalradonc.org/cms/10.1016/j.prro.2018.01.012/attachment/c299a424-455c-47b4-b36b-58b04ffc824f/mmc1.pdf?utm_source=chatgpt.com "Radiation Therapy for the Whole Breast"
[11]: https://pmc.ncbi.nlm.nih.gov/articles/PMC5476174/?utm_source=chatgpt.com "First Results From a Prospective Phase II Trial - PMC"
[12]: https://www.astro.org/provider-resources/guidelines/astro-s-guideline-on-post-mastectomy-radiotherapy?utm_source=chatgpt.com "ASTRO guideline - post-mastectomy radiation"
[13]: https://pubmed.ncbi.nlm.nih.gov/37977261/?utm_source=chatgpt.com "Partial Breast Irradiation for Patients With Early-Stage ..."
[14]: https://pmc.ncbi.nlm.nih.gov/articles/PMC11017153/?

utm_source=chatgpt.com "One versus three weeks hypofractionated whole breast ..."
[15]: https://www.thelancet.com/journals/lancet/article/PIIS0140-6736%2820%2930932-6/fulltext?utm_source=chatgpt.com "Hypofractionated breast radiotherapy for 1 week versus 3 ..."
[16]: https://www.nejm.org/doi/full/10.1056/NEJMoa2207586?utm_source=chatgpt.com "Breast-Conserving Surgery with or without Irradiation in ..."
[17]: https://pubmed.ncbi.nlm.nih.gov/23690420/?utm_source=chatgpt.com "long-term follow-up of CALGB 9343"
[18]: https://jamanetwork.com/journals/jamaoncology/fullarticle/2809872?utm_source=chatgpt.com "Sentinel Lymph Node Biopsy vs No Axillary Surgery in ..."
[19]: https://www.onclive.com/view/keynote-522-neoadjuvant-and-adjuvant-pembrolizumab-in-tnbc "KEYNOTE-522: Neoadjuvant and Adjuvant Pembrolizumab in TNBC"
[20]: https://www.nejm.org/doi/full/10.1056/NEJMoa1910549?utm_source=chatgpt.com "Pembrolizumab for Early Triple-Negative Breast Cancer"
[21]: https://www.keytrudahcp.com/efficacy/early-stage-triple-negative-breast-cancer/?utm_source=chatgpt.com "KEYNOTE-522 - Clinical Trial Results | HCP"
[22]: https://pmc.ncbi.nlm.nih.gov/articles/PMC10652156/?utm_source=chatgpt.com "Pembrolizumab Plus Chemotherapy Followed by ..."
[23]: https://education.nccn.org/sites/default/files/course/2024-02/05_Santa-Maria_NCCNbc24_v1.pdf?utm_source=chatgpt.com "Updates to the Management of Triple Negative Breast ..."
