# Supplementary material for: Performance of large language models in ambiguous treatment scenarios of early breast cancer
Source: Breast. 2026 Sep 23;90:104932. doi: 10.1016/j.breast.2026.104932 (PMC13641045; doi:10.1016/j.breast.2026.104932)
Supplement: Supplementary file 1 [file mmc1.zip › Suppl.Mat. - Anton Oseledchyk - Prompts, LLM Responses, R-Code/radiation_response_claude.pdf]

QUESTION_ID: 16
SELECTED_ANSWER: C
EVIDENCE_SUMMARY: Current guidelines recommend post-mastectomy radiation therapy (PMRT) including regional nodal irradiation for patients with 1-3 positive nodes, though the decision should be individualized based on risk factors. Factors such as age, tumor grade, size, and number of positive nodes influence the recommendation, with most experts recommending comprehensive treatment for patients with positive nodes after mastectomy.
KEY_REFERENCES: ASCO/ASTRO/SSO Focused Guideline Update 2016, EBCTCG meta-analysis 2014, START trials
GUIDELINE_ALIGNMENT: NCCN 2024, ASCO/ASTRO/SSO guidelines support PMRT with RNI for N1 disease
SUBGROUP_NOTES: Age 66 with 1.6cm tumor is intermediate risk; single positive node of 3 examined suggests consideration for comprehensive treatment
CONFIDENCE_LEVEL: Moderate
WEB_SEARCH_USED: Yes
---END---

QUESTION_ID: 17
SELECTED_ANSWER: A
EVIDENCE_SUMMARY: For younger patients (age 41) with T1-2 N1 disease and low-intermediate genomic risk, the benefit of PMRT is controversial, with some experts suggesting omission may be reasonable in selected low-risk patients. The low-intermediate genomic score suggests favorable biology that may reduce the absolute benefit of radiation therapy.
KEY_REFERENCES: MA.20 trial subgroup analysis, RxPONDER biomarker analysis, ASCO/ASTRO guidelines
GUIDELINE_ALIGNMENT: NCCN allows consideration of PMRT omission in selected low-risk N1 patients with favorable features
SUBGROUP_NOTES: Young age (41) typically favors PMRT, but low-intermediate genomic score and single micrometastasis support potential omission
CONFIDENCE_LEVEL: Low
WEB_SEARCH_USED: Yes
---END---

QUESTION_ID: 18
SELECTED_ANSWER: B
EVIDENCE_SUMMARY: Multiple randomized trials including START-B and the Canadian trial have demonstrated that moderate hypofractionation (15-16 fractions) is non-inferior to conventional fractionation (25 fractions) for local control and toxicity. The RT CHARM trial recently demonstrated non-inferiority of hypofractionation even in patients with reconstruction after mastectomy.
KEY_REFERENCES: START-B trial 10-year results, RT CHARM trial 2024, Canadian hypofractionation trial
GUIDELINE_ALIGNMENT: ASTRO, NCCN, and UK guidelines support hypofractionation as standard for PMRT through Feb 2025
SUBGROUP_NOTES: High nodal burden (8/11 positive) and reconstruction do not contraindicate hypofractionation based on recent evidence
CONFIDENCE_LEVEL: High

WEB_SEARCH_USED: Yes
---END---

QUESTION_ID: 19
SELECTED_ANSWER: D
EVIDENCE_SUMMARY: The FAST-Forward trial demonstrated that ultra-hypofractionation (26 Gy in 5 fractions) is non-inferior to standard fractionation for local control with acceptable toxicity at 5 years. For low-risk, strongly ER/PR positive, node-negative disease, ultra-hypofractionation offers excellent convenience without compromising outcomes.
KEY_REFERENCES: FAST-Forward 5-year results, UK START trials, ASTRO hypofractionation guidelines
GUIDELINE_ALIGNMENT: UK and international guidelines support ultra-hypofractionation for suitable patients
SUBGROUP_NOTES: Grade 1, strongly hormone receptor positive, node-negative represents ideal candidate for ultra-hypofractionation
CONFIDENCE_LEVEL: High
WEB_SEARCH_USED: Yes
---END---

QUESTION_ID: 20
SELECTED_ANSWER: C
EVIDENCE_SUMMARY: For patients requiring whole breast irradiation, moderate hypofractionation (15-16 fractions) has been established as the standard of care with equivalent efficacy and improved convenience compared to conventional fractionation. While partial breast irradiation could be considered, whole breast treatment with moderate hypofractionation remains the most evidence-based approach.
KEY_REFERENCES: START-B trial, Canadian trial, ASTRO consensus guidelines
GUIDELINE_ALIGNMENT: NCCN and ASTRO guidelines prefer moderate hypofractionation as standard
SUBGROUP_NOTES: T1c grade 2 disease in a vigorous 69-year-old favors comprehensive treatment with convenient schedule
CONFIDENCE_LEVEL: High
WEB_SEARCH_USED: Yes
---END---

QUESTION_ID: 21
SELECTED_ANSWER: C
EVIDENCE_SUMMARY: Current ASTRO 2024 guidelines strongly recommend partial breast irradiation for suitable candidates with T1c, low-risk features. However, sentinel lymph node biopsy remains standard to confirm node-negative status before proceeding with PBI in patients with clinically negative nodes.
KEY_REFERENCES: ASTRO PBI Guidelines 2024, NSABP B-39/RTOG 0413, GEC-ESTRO trial
GUIDELINE_ALIGNMENT: ASTRO 2024 guidelines support PBI for eligible patients after pathologic nodal assessment
SUBGROUP_NOTES: Postmenopausal women in 60s with T1c low-risk cancer are ideal PBI candidates after confirming N0 status
CONFIDENCE_LEVEL: High
WEB_SEARCH_USED: Yes

---END---

QUESTION_ID: 22
SELECTED_ANSWER: D
EVIDENCE_SUMMARY: While CALGB 9343 and PRIME II trials showed radiation can be omitted in selected elderly patients, standard care remains radiation therapy plus endocrine therapy for optimal local control. At 10 years, the absolute difference in local recurrence is 8.6 percentage points favoring radiation therapy.
KEY_REFERENCES: CALGB 9343 long-term results, PRIME II 10-year update, NCCN guidelines
GUIDELINE_ALIGNMENT: Guidelines allow RT omission discussion but standard remains combined modality therapy
SUBGROUP_NOTES: Age 70 with grade 1-2, strongly ER/PR positive allows consideration of RT omission with informed consent
CONFIDENCE_LEVEL: High
WEB_SEARCH_USED: Yes
---END---

QUESTION_ID: 23
SELECTED_ANSWER: C
EVIDENCE_SUMMARY: KEYNOTE-522 enrolled patients with T1c N1-2 or T2-4 N0-2 TNBC, establishing the 2cm threshold as the standard size cutoff for initiating neoadjuvant therapy with pembrolizumab. Clinical practice generally reserves neoadjuvant pembrolizumab for stage II-III disease, with most experts using a 2cm size threshold.
KEY_REFERENCES: KEYNOTE-522 trial, FDA approval criteria, ASCO guidelines 2022
GUIDELINE_ALIGNMENT: FDA approval and NCCN guidelines support neoadjuvant therapy for T2 (>2cm) or node-positive TNBC
SUBGROUP_NOTES: Clinically node-negative status does not change size threshold of 2cm for neoadjuvant therapy eligibility
CONFIDENCE_LEVEL: High
WEB_SEARCH_USED: Yes
---END---

QUESTION_ID: 24
SELECTED_ANSWER: B
EVIDENCE_SUMMARY: KEYNOTE-522 included tumors >2cm or T1c with node-positive disease; a 1.75cm tumor with negative nodes does not meet standard criteria for neoadjuvant pembrolizumab. Most experts reserve neoadjuvant immunotherapy for stage II-III disease, not using it for tumors under 2cm unless node-positive.
KEY_REFERENCES: KEYNOTE-522 eligibility criteria, FDA label for pembrolizumab, NCCN guidelines
GUIDELINE_ALIGNMENT: Current guidelines do not support neoadjuvant immunotherapy for T1c N0 TNBC
SUBGROUP_NOTES: Despite high-grade features, size <2cm with negative nodes indicates upfront surgery followed by adjuvant therapy
CONFIDENCE_LEVEL: High
WEB_SEARCH_USED: Yes
---END---
