# Supplementary material for: Performance of large language models in ambiguous treatment scenarios of early breast cancer
Source: Breast. 2026 Sep 23;90:104932. doi: 10.1016/j.breast.2026.104932 (PMC13641045; doi:10.1016/j.breast.2026.104932)
Supplement: Supplementary file 1 [file mmc1.zip › Suppl.Mat. - Anton Oseledchyk - Prompts, LLM Responses, R-Code/radiation_response_deepseek.pdf]

QUESTION_ID: 16
SELECTED_ANSWER: C
EVIDENCE_SUMMARY: For post-mastectomy patients with 1-3 positive lymph nodes, chest wall and regional nodal irradiation (RNI) is recommended based on EORTC 22922 and MA.20 trials showing improved survival. Recent NCCN Guidelines (v3.2025) continue to support PMRT for patients with 1-3 positive nodes, particularly with adverse features like younger age or larger tumor size, though this patient's age (66) makes the benefit somewhat more modest.
KEY_REFERENCES: EORTC 22922; MA.20 trial; NCCN Guidelines Breast Cancer v3.2025
GUIDELINE_ALIGNMENT: NCCN (2025) and ASTRO guidelines support PMRT with RNI for patients with 1-3 positive nodes after mastectomy.
SUBGROUP_NOTES: Benefit is clearest for younger patients and those with higher-risk features (e.g., LVI, grade 3). For older patients with limited disease, the absolute benefit is smaller.
CONFIDENCE_LEVEL: High
WEB_SEARCH_USED: Yes - Searched for NCCN Guidelines v3.2025 which continue to recommend PMRT for 1-3 positive nodes.

---END---

QUESTION_ID: 17
SELECTED_ANSWER: C
EVIDENCE_SUMMARY: For a young patient (41 years) with 1 positive node after mastectomy, chest wall and regional nodal irradiation is strongly recommended. Young age is a well-established risk factor for locoregional recurrence, and genomic risk (low-intermediate) does not alter the indication for PMRT, as per NCCN Guidelines (v3.2025). Trials like EORTC 22922 show significant overall survival benefit with RNI in node-positive patients.
KEY_REFERENCES: EORTC 22922; MA.20 trial; NCCN Guidelines Breast Cancer v3.2025
GUIDELINE_ALIGNMENT: NCCN (2025) and ASTRO guidelines strongly recommend PMRT with RNI for young patients with positive nodes.
SUBGROUP_NOTES: Young age (<50) is a key factor mandating PMRT. Genomic scores guide systemic therapy but not radiation decisions.
CONFIDENCE_LEVEL: High
WEB_SEARCH_USED: Yes - Confirmed NCCN Guidelines v3.2025 maintain strong recommendation for PMRT in young patients with positive nodes.

---END---

QUESTION_ID: 18
SELECTED_ANSWER: A
EVIDENCE_SUMMARY: For a patient with extensive nodal involvement (8/11 nodes) post-mastectomy, conventional fractionation (25 fractions of 50 Gy) remains the standard of care. While hypofractionation is increasingly used for many breast cancer patients, patients with advanced disease (T3N2) and extensive nodal involvement were underrepresented in hypofractionation trials like FAST-Forward. ASTRO guidelines (2024, still current in Feb 2025) recommend conventional fractionation for such high-risk cases.

KEY_REFERENCES: FAST-Forward trial; ASTRO Guidelines (2024)
GUIDELINE_ALIGNMENT: ASTRO Guidelines (2024) recommend conventional fractionation for post-mastectomy patients with advanced disease (T3/T4 or N2/N3).
SUBGROUP_NOTES: The presence of implant reconstruction may also influence fractionation choice to minimize capsular contracture risk, favoring conventional fractionation.
CONFIDENCE_LEVEL: High
WEB_SEARCH_USED: Yes - Searched for ASTRO guideline updates; the 2024 guideline (valid through Feb 2025) specifies conventional fractionation for high-risk cases like T3N2.

---END---

QUESTION_ID: 19
SELECTED_ANSWER: C
EVIDENCE_SUMMARY: For this low-risk, node-negative, postmenopausal patient, moderate hypofractionation (15-16 fractions) is the standard recommendation based on long-term data from the UK START trials and the Canadian hypofractionation trial. The FAST-Forward trial also supports 5-fraction regimens, but 15-16 fractions remains the most widely adopted and guideline-supported hypofractionated schedule for whole breast irradiation.
KEY_REFERENCES: START trials; Canadian Hypofractionation Trial; FAST-Forward trial
GUIDELINE_ALIGNMENT: ASTRO (2024) and NCCN (2025) guidelines endorse moderate hypofractionation as a standard option for whole breast irradiation in low-risk patients.
SUBGROUP_NOTES: This patient has ideal low-risk features: postmenopausal, T1c, grade 1, strongly HR+.
CONFIDENCE_LEVEL: High
WEB_SEARCH_USED: No - Based on established trials and guidelines; no new evidence through Feb 2025 changes this standard.

---END---

QUESTION_ID: 20
SELECTED_ANSWER: D
EVIDENCE_SUMMARY: For this healthy, older patient with low-risk disease (T1c, node-negative, HR+), multiple fractionation schedules are acceptable. Ultra-hypofractionation (5 fractions) is supported by the FAST-Forward trial, which showed non-inferiority to standard fractionation with 10-year follow-up. NCCN Guidelines (v3.2025) list 5-fraction whole breast irradiation as an option for patients meeting FAST-Forward criteria.
KEY_REFERENCES: FAST-Forward trial; NCCN Guidelines Breast Cancer v3.2025
GUIDELINE_ALIGNMENT: NCCN (2025) and ASTRO (2024) guidelines endorse ultra-hypofractionation (5 fractions) as an option for whole breast irradiation in selected patients.
SUBGROUP_NOTES: The patient's excellent health and long life expectancy support the use of the most convenient effective regimen.
CONFIDENCE_LEVEL: High
WEB_SEARCH_USED: Yes - Confirmed NCCN v3.2025 continues to include

5-fraction WBI as a category 1 recommendation for eligible patients.

---END---

QUESTION_ID: 21
SELECTED_ANSWER: C
EVIDENCE_SUMMARY: For a patient with a T1c cancer, sentinel lymph node biopsy (SLNB) is standard to confirm nodal status before considering any form of radiation. While axillary ultrasound is negative, its false negative rate is not zero. Partial breast irradiation (PBI) requires confirmed node-negative status, as its safety has not been established in node-positive disease. NCCN Guidelines (v3.2025) continue to recommend SLNB for T1c tumors.
KEY_REFERENCES: Z0011 trial; NCCN Guidelines Breast Cancer v3.2025
GUIDELINE_ALIGNMENT: NCCN (2025) and ASTRO guidelines recommend SLNB for T1b and larger tumors; PBI is only recommended for node-negative disease.
SUBGROUP_NOTES: While there is growing data on omitting SLNB in some low-risk patients, this is not yet standard for T1c tumors.
CONFIDENCE_LEVEL: High
WEB_SEARCH_USED: Yes - Verified NCCN v3.2025 has not changed the recommendation for SLNB in T1c tumors.

---END---

QUESTION_ID: 22
SELECTED_ANSWER: D
EVIDENCE_SUMMARY: For a 70-year-old with a 1.3 cm, HR+ tumor, the standard of care is both endocrine therapy and radiation therapy after BCS. While the CALGB 9343 trial showed that omitting radiation is an option for older women with similar tumors, it resulted in a higher rate of local recurrence. The PRIME II trial also supports this option, but the combination of RT + ET provides the best local control. NCCN Guidelines (v3.2025) list omitting RT as an option but note it is associated with higher local recurrence.
KEY_REFERENCES: CALGB 9343 trial; PRIME II trial; NCCN Guidelines Breast Cancer v3.2025
GUIDELINE_ALIGNMENT: NCCN (2025) states that radiation may be omitted in women ≥70 years with T1, ER+ tumors receiving endocrine therapy, but this is associated with a higher local recurrence rate.
SUBGROUP_NOTES: The decision involves a trade-off between local control and treatment burden. For a healthy 70-year-old, the absolute benefit of RT is still significant.
CONFIDENCE_LEVEL: High
WEB_SEARCH_USED: Yes - Confirmed NCCN v3.2025 maintains the same criteria for considering omission of RT.

---END---

QUESTION_ID: 23
SELECTED_ANSWER: C
EVIDENCE_SUMMARY: The KEYNOTE-522 trial established neoadjuvant chemotherapy with pembrolizumab for stage II and III triple-negative breast cancer (TNBC). The trial included patients with tumors ≥1.0

cm, but the significant event-free survival benefit was most pronounced in higher-risk disease. NCCN Guidelines (v3.2025) recommend this regimen for "most patients with stage II or III TNBC," which typically includes tumors T≥1cm (stage II).
KEY_REFERENCES: KEYNOTE-522 trial; NCCN Guidelines Breast Cancer v3.2025
GUIDELINE_ALIGNMENT: NCCN (2025) recommends neoadjuvant pembrolizumab/chemotherapy for stage II-III TNBC.
SUBGROUP_NOTES: For very small T1b tumors, the benefit is less clear, and the decision is often individualized. A tumor size of 2.0 cm is firmly within the range for recommendation.
CONFIDENCE_LEVEL: High
WEB_SEARCH_USED: Yes - Searched for NCCN Guidelines v3.2025 which continue to categorize this regimen for stage II/III TNBC.

---END---

QUESTION_ID: 24
SELECTED_ANSWER: A
EVIDENCE_SUMMARY: Yes, neoadjuvant chemotherapy with checkpoint inhibitor immunotherapy (pembrolizumab) is recommended for this patient. The KEYNOTE-522 regimen is standard for stage II and III triple-negative breast cancer. A tumor size of 1.75 cm (>1.0 cm) qualifies as at least stage II (if node-negative) or higher, and high-grade histology further increases the risk, making her an ideal candidate for this regimen which improves pathological complete response and event-free survival.
KEY_REFERENCES: KEYNOTE-522 trial; NCCN Guidelines Breast Cancer v3.2025
GUIDELINE_ALIGNMENT: NCCN (2025) guidelines recommend neoadjuvant pembrolizumab/chemotherapy for stage II-III TNBC.
SUBGROUP_NOTES: The clinically negative axilla does not change the recommendation, as the tumor itself is >1 cm and high-grade.
CONFIDENCE_LEVEL: High
WEB_SEARCH_USED: No - Based on the established KEYNOTE-522 protocol and NCCN guidelines; no new evidence through Feb 2025 changes this.

---END---
