# Supplementary material for: Performance of large language models in ambiguous treatment scenarios of early breast cancer
Source: Breast. 2026 Sep 23;90:104932. doi: 10.1016/j.breast.2026.104932 (PMC13641045; doi:10.1016/j.breast.2026.104932)
Supplement: Supplementary file 1 [file mmc1.zip › Suppl.Mat. - Anton Oseledchyk - Prompts, LLM Responses, R-Code/radiation_response_gemini.pdf]

QUESTION_ID: 16
SELECTED_ANSWER: C
EVIDENCE_SUMMARY: For a patient with a T1 tumor and one positive sentinel lymph node after mastectomy, the standard of care is post-mastectomy radiation therapy (PMRT). Landmark trials like the EORTC 22922 and MA.20 have demonstrated a clear benefit for adding regional nodal irradiation (RNI) to chest wall irradiation in patients with 1-3 positive lymph nodes, showing improved locoregional control and survival. This patient's N1 status is a strong indication for RNI.
KEY_REFERENCES: EORTC 22922 trial (NEJM 1999); MA.20 trial (NEJM 2014); NCCN Guidelines for Breast Cancer (v1.2024).
GUIDELINE_ALIGNMENT: The NCCN Breast Cancer Guidelines (versions through Feb 2025) recommend PMRT including the chest wall and regional nodal basins for patients with 1-3 positive lymph nodes, particularly after mastectomy.
SUBGROUP_NOTES: While the tumor is small, the presence of a positive node is the primary driver for a recommendation of RNI.
CONFIDENCE_LEVEL: High
WEB_SEARCH_USED: Yes - Searched "EORTC 22922 trial results," "MA.20 trial chest wall and RNI," and "NCCN guidelines PMRT 1-3 nodes." Found that these trials' results and the resulting guideline recommendations were well-established and adopted prior to February 2025.
---END---
QUESTION_ID: 17
SELECTED_ANSWER: C
EVIDENCE_SUMMARY: The patient's N1 status (1 positive node) after mastectomy is the primary determinant for the radiation therapy recommendation. As established by the EORTC 22922 and MA.20 trials, chest wall irradiation combined with regional nodal irradiation (RNI) is standard for patients with 1-3 positive nodes to improve locoregional control and survival. The low to intermediate genomic score, while relevant for systemic therapy decisions (chemotherapy), is not a recognized factor for de-escalating radiation therapy, which is based on anatomic risk.
KEY_REFERENCES: EORTC 22922 trial (NEJM 1999); MA.20 trial (NEJM 2014); NCCN Guidelines for Breast Cancer (v1.2024).
GUIDELINE_ALIGNMENT: NCCN and other major guidelines (through Feb 2025) base radiation recommendations on surgical and pathologic factors (e.g., nodal status, tumor size), not on genomic scores. The N1 status dictates the need for chest wall and RNI.
SUBGROUP_NOTES: The patient's young age further supports an aggressive approach to locoregional control.
CONFIDENCE_LEVEL: High
WEB_SEARCH_USED: Yes - Searched "genomic scores radiation therapy breast cancer," "NCCN guidelines genomic assay RT." Confirmed that up to Feb 2025, genomic assays were not used to guide radiation decisions.
---END---
QUESTION_ID: 18
SELECTED_ANSWER: B
EVIDENCE_SUMMARY: The patient's stage 3 breast cancer with extensive nodal involvement (8/11 positive) is a clear indication for post-

mastectomy radiation therapy (PMRT) with regional nodal irradiation (RNI). The standard of care has shifted from conventional fractionation (25 fractions) to moderate hypofractionation (15-16 fractions) for both whole breast and post-mastectomy radiation. Landmark trials like the UK FAST and Canadian trials have demonstrated that moderate hypofractionation is non-inferior in terms of local control and has similar or better cosmetic outcomes and toxicity profiles.
KEY_REFERENCES: UK FAST trials (Lancet Oncol 2011); Canadian Hypofractionation trial (NEJM 2002); NCCN Guidelines for Breast Cancer (v1.2024).
GUIDELINE_ALIGNMENT: ASTRO and NCCN guidelines (up to Feb 2025) strongly endorse moderate hypofractionation for both whole breast and post-mastectomy radiation, including when regional nodal basins are irradiated.
SUBGROUP_NOTES: This recommendation is applicable even in the setting of implant reconstruction.
CONFIDENCE_LEVEL: High
WEB_SEARCH_USED: Yes - Searched "hypofractionated PMRT NCCN," "UK FAST trial PMRT," and "Canadian hypofractionation trial." The search confirmed the widespread adoption of moderate hypofractionation for PMRT prior to March 2025.
---END---
QUESTION_ID: 19
SELECTED_ANSWER: C
EVIDENCE_SUMMARY: This patient has a low-risk, stage 1 breast cancer treated with breast-conserving surgery (BCS). The standard of care is whole breast irradiation. The START A and B trials, as well as the Canadian trial, established that moderate hypofractionation (15-16 fractions) is non-inferior to conventional fractionation (25 fractions) in terms of local control and offers superior convenience and reduced cost. While ultra-hypofractionation (5 days) is an option based on trials like FAST-Forward, moderate hypofractionation is the most widely adopted and established hypofractionated schedule for this patient profile as of early 2025.
KEY_REFERENCES: START A and B trials (Lancet Oncol 2008); Canadian Hypofractionation trial (NEJM 2002); NCCN Guidelines for Breast Cancer (v1.2024).
GUIDELINE_ALIGNMENT: NCCN and ASTRO guidelines (up to Feb 2025) recommend moderate hypofractionation as the preferred treatment schedule for most patients receiving whole breast irradiation.
SUBGROUP_NOTES: The patient's low-risk tumor and node-negative status make her an ideal candidate for hypofractionation.
CONFIDENCE_LEVEL: High
WEB_SEARCH_USED: Yes - Searched "NCCN guidelines hypofractionation breast cancer," and "START trials." Confirmed the widespread adoption of moderate hypofractionation as the standard of care before the cutoff date.
---END---
QUESTION_ID: 20
SELECTED_ANSWER: D
EVIDENCE_SUMMARY: This patient is a vigorous 69-year-old with a low-risk, node-negative, T1c, ER-positive tumor. She is an excellent candidate for breast-conserving surgery followed by radiation

therapy. Given her age, vigor, and long-life expectancy, she is an ideal candidate for de-escalation of treatment burden while maintaining efficacy. The FAST-Forward trial demonstrated that a 5-fraction, ultra-hypofractionated regimen is non-inferior to the standard 15-fraction regimen in terms of local control and has similar toxicity, making it a highly convenient and effective option for this low-risk group.
KEY_REFERENCES: FAST-Forward trial (Lancet 2020); ASTRO Consensus Guidelines on Hypofractionated Whole Breast Irradiation (2018).
GUIDELINE_ALIGNMENT: ASTRO and NCCN guidelines (up to Feb 2025) have incorporated ultra-hypofractionation as a valid and recommended option for carefully selected, low-risk patients like this one.
SUBGROUP_NOTES: The patient's stated life expectancy makes de-escalation of the RT schedule based on convenience and quality of life a priority, while maintaining efficacy.
CONFIDENCE_LEVEL: High
WEB_SEARCH_USED: Yes - Searched "FAST-Forward trial results," "ASTRO guidelines ultra-hypofractionation." Confirmed the publication of the trial and its incorporation into major guidelines before the specified cutoff date.
---END---
QUESTION_ID: 21
SELECTED_ANSWER: C
EVIDENCE_SUMMARY: The patient has a clinically negative axilla, but the tumor size is T1c (>1.0 cm). The standard of care for a tumor of this size, even with low-risk features, is to perform axillary staging via a sentinel lymph node biopsy (SLNB) to confirm node-negative status. If the SLN is negative, the patient is a perfect candidate for partial breast irradiation (PBI), given her age and low-risk tumor. PBI targets only the tumor bed, offering a shorter and more convenient course of treatment than whole breast irradiation.
KEY_REFERENCES: ASTRO Consensus Statement on Partial Breast Irradiation (2017); NCCN Breast Cancer Guidelines (v1.2024).
GUIDELINE_ALIGNMENT: NCCN and ASTRO guidelines (up to Feb 2025) recommend SLNB for this patient profile and support PBI for low-risk, node-negative patients who meet specific criteria (age >50, T1, N0).
SUBGROUP_NOTES: PBI is a suitable treatment for carefully selected patients, and this patient fits the established criteria.
CONFIDENCE_LEVEL: High
WEB_SEARCH_USED: Yes - Searched "ASTRO PBI guidelines," "NCCN guidelines sentinel node biopsy T1c." Confirmed the sequential steps of staging and then de-escalated therapy for this specific patient profile.
---END---
QUESTION_ID: 22
SELECTED_ANSWER: D
EVIDENCE_SUMMARY: This patient has a T1c, ER-positive tumor post-breast conserving surgery. The standard of care for an ER-positive tumor is adjuvant endocrine therapy to reduce the risk of systemic recurrence. While trials like CALGB 9343 have shown that omitting radiation therapy does not impact survival in older patients, it significantly increases the risk of local recurrence. Given the

patient's age (70) and tumor size, both endocrine therapy and radiation therapy are standard options. The most comprehensive recommendation for achieving both systemic and local control is to provide both.
KEY_REFERENCES: CALGB 9343 trial (JCO 2004, 2010); NCCN Guidelines for Breast Cancer (v1.2024).
GUIDELINE_ALIGNMENT: NCCN and ASTRO guidelines (up to Feb 2025) present both options (endocrine therapy alone or with RT) for this specific patient population, acknowledging that the primary goal of RT is local control. The standard, most comprehensive recommendation is to provide both to maximize control.
SUBGROUP_NOTES: Omitting RT is a valid choice discussed with the patient, but recommending both therapies is a standard and safe approach for comprehensive management.
CONFIDENCE_LEVEL: High
WEB_SEARCH_USED: Yes - Searched "CALGB 9343 trial outcomes," and "NCCN guidelines RT omission elderly." Found that while omission of RT is an option, it is not the only recommended choice and the standard of care includes both.
---END---
QUESTION_ID: 23
SELECTED_ANSWER: A
EVIDENCE_SUMMARY: The KEYNOTE-522 trial, which evaluated the addition of pembrolizumab to neoadjuvant chemotherapy for triple-negative breast cancer (TNBC), enrolled patients with a tumor size of at least T1c. A T1c tumor is defined as being greater than 1.0 cm and up to 2.0 cm. Therefore, a tumor size of >1.0 cm with a clinically negative axilla is the established threshold for initiating this treatment regimen.
KEY_REFERENCES: KEYNOTE-522 trial (NEJM 2020); NCCN Breast Cancer Guidelines (v1.2024).
GUIDELINE_ALIGNMENT: NCCN guidelines (up to Feb 2025) specifically reference the KEYNOTE-522 trial and recommend neoadjuvant pembrolizumab + chemotherapy for T1c, N0 or T2-4, N0-2 TNBC.
SUBGROUP_NOTES: This recommendation applies to patients with clinically negative axillary lymph nodes and is now a standard of care for this patient group.
CONFIDENCE_LEVEL: High
WEB_SEARCH_USED: Yes - Searched "KEYNOTE-522 inclusion criteria," and "NCCN guidelines TNBC pembrolizumab." Confirmed the T1c (>1.0 cm) threshold for inclusion in the trial and subsequent guideline recommendations.
---END---
QUESTION_ID: 24
SELECTED_ANSWER: A
EVIDENCE_SUMMARY: The patient has a high-grade, triple-negative breast cancer with a clinically negative axilla, and a tumor size of 1.74 cm. This tumor size falls within the T1c category (>1.0 cm). The KEYNOTE-522 trial demonstrated a significant benefit in both pathologic complete response (pCR) and event-free survival with the addition of pembrolizumab to neoadjuvant chemotherapy in patients with stage 2 or 3 TNBC. This included patients with T1c, N0 disease, making her an ideal candidate for this regimen.
KEY_REFERENCES: KEYNOTE-522 trial (NEJM 2020); NCCN Breast Cancer

Guidelines (v1.2024).
GUIDELINE_ALIGNMENT: NCCN and other major guidelines (up to Feb 2025) recommend neoadjuvant pembrolizumab + chemotherapy for patients with T1c, N0 or higher stage TNBC.
SUBGROUP_NOTES: The young age and aggressive biology of TNBC support a neoadjuvant approach to assess response to therapy and improve outcomes.
CONFIDENCE_LEVEL: High
WEB_SEARCH_USED: Yes - Searched "KEYNOTE-522 TNBC pCR," and "NCCN guidelines neoadjuvant pembrolizumab." Confirmed that the trial results and guideline recommendations support using this regimen for this patient profile.
---END---
