# Supplementary material for: Performance of large language models in ambiguous treatment scenarios of early breast cancer
Source: Breast. 2026 Sep 23;90:104932. doi: 10.1016/j.breast.2026.104932 (PMC13641045; doi:10.1016/j.breast.2026.104932)
Supplement: Supplementary file 1 [file mmc1.zip › Suppl.Mat. - Anton Oseledchyk - Prompts, LLM Responses, R-Code/tnbc_response_chatgpt.pdf]

QUESTION_ID: 25
SELECTED_ANSWER: A
EVIDENCE_SUMMARY: After upfront surgery revealing pT1cN1 TNBC, the standard adjuvant approach is anthracycline/taxane chemotherapy (e.g., dose-dense AC→T). Neoadjuvant pembrolizumab-based chemoimmunotherapy from KEYNOTE-522 cannot be “back-filled” post-op, and adjuvant checkpoint blockade without prior neoadjuvant use is not established. Guidelines through 2024 support adjuvant AC→T (or TC if anthracycline-ineligible) for node-positive TNBC. ([New England Journal of Medicine][1], [nursingcenter.com][2], [PMC][3])
KEY_REFERENCES: KEYNOTE-522 (Schmid et al., NEJM 2022/2024) — establishes benefit for neoadjuvant pembro + chemo, not de-novo adjuvant use; NCCN Breast Cancer 2024 regimen summary for adjuvant chemo (AC→T/TC). ([New England Journal of Medicine][1], [nursingcenter.com][2])
GUIDELINE_ALIGNMENT: NCCN Breast Cancer (2024) and ESMO Early Breast Cancer (2024) endorse adjuvant anthracycline/taxane chemotherapy for node-positive HER2-negative/TNBC after upfront surgery. ([nursingcenter.com][2], [PubMed][4])
SUBGROUP_NOTES: Consider non-anthracycline TC for cardiac risk; germline BRCA may influence future PARP inhibitor use only in high-risk settings after (neo)adjuvant chemo. ([Nature][5])
CONFIDENCE_LEVEL: High
WEB_SEARCH_USED: Yes — searched “NCCN 2024 TNBC adjuvant chemotherapy,” “KEYNOTE-522 adjuvant component,” and “ESMO 2024 early breast cancer”; found that adjuvant IO is not recommended de-novo and AC→T remains standard (through Feb 2025).
\---END---

QUESTION_ID: 26
SELECTED_ANSWER: A
EVIDENCE_SUMMARY: With 3/5 positive nodes after upfront surgery, TNBC remains an indication for adjuvant anthracycline/taxane chemotherapy; checkpoint inhibitor therapy in the adjuvant-only setting is not standard. This aligns with NCCN/ESMO through 2024–early 2025 which list AC→T (or TC if needed) as preferred adjuvant regimens for node-positive HER2-negative disease. ([nursingcenter.com][2], [PubMed][4])
KEY_REFERENCES: NCCN Breast Cancer 2024 adjuvant chemotherapy regimens; ESMO Early Breast Cancer 2024 guideline. ([nursingcenter.com][2], [PubMed][4])
GUIDELINE_ALIGNMENT: NCCN (2024) and ESMO (2024) recommend adjuvant anthracycline/taxane chemo for node-positive TNBC after upfront surgery. ([nursingcenter.com][2], [PubMed][4])
SUBGROUP_NOTES: Consider dose-dense scheduling when feasible; tailor for comorbidities (e.g., cardiac).
CONFIDENCE_LEVEL: High
WEB_SEARCH_USED: Yes — verified NCCN 2024 adjuvant regimens and ESMO 2024 guidance; no new evidence supporting de-novo adjuvant IO through Feb 2025. ([nursingcenter.com][2], [PubMed][4])
\---END---

QUESTION_ID: 27
SELECTED_ANSWER: B

EVIDENCE_SUMMARY: The regimen most faithful to KEYNOTE-522 uses weekly paclitaxel with weekly carboplatin followed by q3-week AC, alongside pembrolizumab throughout; both weekly and q3-week carboplatin were permitted, with weekly commonly used. KEYNOTE-522 showed improved pCR, EFS, and OS with this peri-operative approach. ([PMC][6], [New England Journal of Medicine][7])
KEY_REFERENCES: KEYNOTE-522 original NEJM report (2020), EFS update (2022), and OS analysis (2024) — dosing allowed weekly carboplatin with weekly paclitaxel then q3-week AC. ([New England Journal of Medicine][7])
GUIDELINE_ALIGNMENT: ESMO 2024 and ASCO/NCCN educational updates endorse neoadjuvant pembro + chemo per KEYNOTE-522, then adjuvant pembro to complete 1 year. ([Annals of Oncology][8], [education.nccn.org][9])
SUBGROUP_NOTES: Dose-dense AC (q2-week) is used in practice, but KEYNOTE-522 used q3-week AC; consider patient fitness and growth-factor support if intensifying. ([PMC][10])
CONFIDENCE_LEVEL: High
WEB_SEARCH_USED: Yes — checked KEYNOTE-522 dosing details and outcomes (NEJM 2020/2022/2024) and educational summaries. ([PMC][6], [New England Journal of Medicine][7])
\---END---

QUESTION_ID: 28
SELECTED_ANSWER: C
EVIDENCE_SUMMARY: In GeparNuevo, durvalumab was given neoadjuvantly only and improved long-term outcomes despite a modest pCR increase; there was no adjuvant durvalumab component. Switching to or adding adjuvant checkpoint inhibition after neoadjuvant durvalumab is not supported by current guidelines; adjuvant pembrolizumab is specifically tied to patients who started pembrolizumab pre-op. ([PubMed][11], [Annals of Oncology][12], [PMC][13])
KEY_REFERENCES: GeparNuevo (Loibl et al., Ann Oncol 2022); ESMO Early Breast Cancer 2024 guideline (peri-operative pembro standard for stage II–III TNBC). ([Annals of Oncology][12], [PubMed][4])
GUIDELINE_ALIGNMENT: ESMO 2024 and NCCN education materials restrict adjuvant IO to continuation after neoadjuvant pembrolizumab. ([Annals of Oncology][8], [education.nccn.org][14])
SUBGROUP_NOTES: If pCR is achieved after neoadjuvant IO, no adjuvant IO is recommended outside trials; proceed with standard RT/systemic therapy per pathology. ([Annals of Oncology][8])
CONFIDENCE_LEVEL: High
WEB_SEARCH_USED: Yes — searched “GeparNuevo durvalumab adjuvant,” and “ESMO 2024 early TNBC immunotherapy guidance.” ([PubMed][11], [Annals of Oncology][12])
\---END---

QUESTION_ID: 29
SELECTED_ANSWER: B
EVIDENCE_SUMMARY: After KN522-style neoadjuvant therapy with residual TNBC, adjuvant pembrolizumab should be continued to complete 1 year, and capecitabine is supported by CREATE-X for residual HER2-negative disease. NCCN educational materials note both options and acknowledge limited data on sequencing/combining; many

clinicians continue pembrolizumab and add capecitabine for higher-risk residual disease. ([New England Journal of Medicine][1], [education.nccn.org][14])
KEY_REFERENCES: KEYNOTE-522 EFS/OS (NEJM 2022/2024); CREATE-X (NEJM 2017); NCCN 2023–2024 slide set stating pembro/capecitabine/olaparib options for post-neoadjuvant residual disease (no definitive sequencing data). ([New England Journal of Medicine][1], [education.nccn.org][14])
GUIDELINE_ALIGNMENT: NCCN (2023–2024 education) and ESMO 2024 endorse adjuvant pembrolizumab continuation after neoadjuvant pembro; capecitabine recommended for residual TNBC. ([education.nccn.org][14], [PubMed][4])
SUBGROUP_NOTES: Consider capecitabine benefit greatest in substantial residual burden; tailor for toxicity, dihydropyrimidine dehydrogenase status, and patient preference.
CONFIDENCE_LEVEL: Moderate
WEB_SEARCH_USED: Yes – reviewed NEJM KN522 updates, CREATE-X, and NCCN education statements on combining/sequencing capecitabine with pembrolizumab (through Feb 2025). ([New England Journal of Medicine][15], [education.nccn.org][14])
\---END---

QUESTION_ID: 30
SELECTED_ANSWER: B
EVIDENCE_SUMMARY: For stage II, node-positive TNBC when checkpoint inhibitors are contraindicated, adding carboplatin to taxane/anthracycline neoadjuvant chemotherapy improves pCR and has shown EFS benefit (BrighTNess), making TCb→AC a preferred option. Guidelines and reviews support platinum-containing neoadjuvant regimens in high-risk TNBC. ([PubMed][16], [MDPI][17], [Annals of Oncology][8])
KEY_REFERENCES: BrighTNess final analyses (Ann Oncol 2021/2022); CALGB 40603 and GeparSixto (pCR improvement with platinum); ESMO Early Breast Cancer 2024. ([PubMed][16], [MDPI][17])
GUIDELINE_ALIGNMENT: ESMO 2024 (and NCCN education) endorse platinum-containing neoadjuvant chemo for stage II–III TNBC, particularly without IO. ([Annals of Oncology][8], [education.nccn.org][14])
SUBGROUP_NOTES: Monitor hematologic toxicity; consider BRCA status (may influence subsequent PARP inhibitor eligibility if high-risk). ([Nature][5])
CONFIDENCE_LEVEL: High
WEB_SEARCH_USED: Yes – searched “BrighTNess EFS,” “platinum in neoadjuvant TNBC,” and “ESMO 2024 early TNBC recommendations.” ([PubMed][16], [MDPI][17])
\---END---

QUESTION_ID: 31
SELECTED_ANSWER: B
EVIDENCE_SUMMARY: For a 2.5 cm cN0 stage II TNBC, neoadjuvant pembrolizumab + chemotherapy per KEYNOTE-522 improves EFS and OS and remains recommended despite limited data on fertility effects of ICIs. Oncofertility best practices (consultation, cryopreservation, and GnRH agonist during chemo) are advised, and literature to date

indicates the fertility impact of ICIs is uncertain (mixed preclinical data, scarce human data); thus proceed with standard-of-care pembrolizumab while optimizing fertility preservation. ([New England Journal of Medicine][1], [ASC Publications][18], [ScienceDirect][19], [PMC][20])
KEY_REFERENCES: KEYNOTE-522 EFS/OS (NEJM 2022/2024); ASCO/oncology literature on fertility preservation and GnRH agonists; 2024 reviews on ICI fertility risk (uncertain). ([New England Journal of Medicine][1], [ASC Publications][18], [ScienceDirect][19])
GUIDELINE_ALIGNMENT: ESMO 2024 endorses peri-operative pembrolizumab for stage II–III TNBC; oncofertility principles per ASCO/consensus reviews. ([PubMed][4], [ASC Publications][18])
SUBGROUP_NOTES: Delay pregnancy attempts until completion of systemic therapy and appropriate washout; monitor for endocrine irAEs that could impact ovarian function. ([ScienceDirect][19])
CONFIDENCE_LEVEL: Moderate
WEB_SEARCH_USED: Yes — verified KN522 benefits, fertility-preservation guidance, and evidence on ICI fertility effects (uncertain) through Feb 2025. ([New England Journal of Medicine][1], [ASC Publications][18], [ScienceDirect][19])
\---END---

QUESTION_ID: 32
SELECTED_ANSWER: B
EVIDENCE_SUMMARY: In gBRCA-mutated TNBC with residual nodal disease after neoadjuvant pembro-chemo, continue adjuvant pembrolizumab to complete 1 year (per KN522) and give 1 year of adjuvant olaparib (per OlympiA), as both independently improve outcomes; NCCN education materials acknowledge no definitive sequencing/combination data, allowing sequential or concurrent use. This strategy maximizes proven benefits in high-risk, HER2-negative gBRCA carriers. ([New England Journal of Medicine][1], [OncLive][21], [education.nccn.org][14])
KEY_REFERENCES: OlympiA (NEJM 2021; Lancet Oncol 2022 update) demonstrating DFS/OS benefit of adjuvant olaparib; KEYNOTE-522 (NEJM 2022/2024) for peri-operative pembrolizumab; NCCN education slide (2023/2024) noting feasibility of combining/sequencing pembro/capecitabine/olaparib. ([OncLive][21], [New England Journal of Medicine][1], [education.nccn.org][14])
GUIDELINE_ALIGNMENT: ESMO 2024 endorses adjuvant olaparib for high-risk gBRCA HER2-negative EBC; continuation of pembrolizumab per KN522 pathway. ([PubMed][4])
SUBGROUP_NOTES: Overlapping toxicities (anemia/fatigue) warrant monitoring; prioritize olaparib if forced to choose due to OlympiA's OS benefit, but many complete both when tolerable. ([OncLive][21])
CONFIDENCE_LEVEL: High
WEB_SEARCH_USED: Yes — searched "OlympiA adjuvant olaparib," "KN522 adjuvant pembrolizumab continuation," and "NCCN sequence/combination statements." ([OncLive][21], [New England Journal of Medicine][1], [education.nccn.org][14])
\---END---

QUESTION_ID: 33
SELECTED_ANSWER: D

EVIDENCE_SUMMARY: After pCR to neoadjuvant chemotherapy, a positive tumor-informed MRD assay (ctDNA) is prognostic but not yet actionable in early breast cancer; ctDNA-guided escalation has not shown clear clinical utility. In cTRAK-TN, most ctDNA-positive patients already had occult metastases at detection and pembrolizumab did not consistently clear ctDNA; guidelines do not recommend starting adjuvant therapy solely for MRD positivity outside a trial. ([PubMed][22], [Enlighten Publications][23], [Nature][24])
KEY_REFERENCES: cTRAK-TN (Ann Oncol 2023); Reviews on ctDNA/MRD in early breast cancer (2024) highlighting investigational status. ([PubMed][22], [Nature][24])
GUIDELINE_ALIGNMENT: No NCCN/ESMO recommendation (through Feb 2025) to treat based on MRD alone; observation or clinical trial enrollment is appropriate. ([Nature][24])
SUBGROUP_NOTES: Intensified surveillance reasonable; discuss trial options for MRD-directed therapy where available.
CONFIDENCE_LEVEL: Moderate
WEB_SEARCH_USED: Yes — searched "cTRAK-TN results," "ctDNA MRD early breast cancer guidelines," confirming no practice-changing data through Feb 2025. ([PubMed][22], [Nature][24])
\---END---

QUESTION_ID: 41
SELECTED_ANSWER: A
EVIDENCE_SUMMARY: ER-low (5%) breast cancers are reported as ER-positive per ASCO/CAP (≥1%), with limited endocrine benefit but they are not considered TNBC for immunotherapy eligibility; thus, neoadjuvant pembrolizumab is not standard here. For a 3–4 cm, grade 3, cN0 tumor, standard neoadjuvant chemotherapy such as AC→T (without pembrolizumab) is appropriate; endocrine therapy can be considered post-op recognizing uncertain benefit. ([documents.cap.org][25], [PubMed][4], [New England Journal of Medicine][7])
KEY_REFERENCES: ASCO/CAP ER testing guideline note for ER-low (2020); ESMO 2024 Early Breast Cancer guideline; KEYNOTE-522 (TNBC-only). ([documents.cap.org][25], [PubMed][4], [New England Journal of Medicine][7])
GUIDELINE_ALIGNMENT: ASCO/CAP (2020) defines ER-low as ER-positive; ESMO 2024 reserves peri-operative pembrolizumab for stage II–III TNBC, not ER-positive disease. ([documents.cap.org][25], [PubMed][4])
SUBGROUP_NOTES: If repeat biomarker testing showed ER <1% (true TNBC), then KN522-style chemo-IO would be appropriate; otherwise treat as ER-positive with chemo and consider endocrine therapy. ([documents.cap.org][25])
CONFIDENCE_LEVEL: High
WEB_SEARCH_USED: Yes — verified ASCO/CAP ER-low definition and ESMO 2024 positioning of IO limited to TNBC. ([documents.cap.org][25], [PubMed][4])
\---END---

[1]: https://www.nejm.org/doi/full/10.1056/NEJMoa2112651?utm_source=chatgpt.com "Event-free Survival with Pembrolizumab in

Early Triple- ..."
[2]: https://www.nursingcenter.com/getattachment/Clinical-Resources/Guideline-Summaries/Breast-Cancer/Breast-Cancer-Guidelines-2024-Update.pdf.aspx?utm_source=chatgpt.com "National Comprehensive Cancer Network (NCCN) Clinical ..."
[3]: https://pmc.ncbi.nlm.nih.gov/articles/PMC9962369/?utm_source=chatgpt.com "Current Treatment Landscape for Early Triple-Negative ..."
[4]: https://pubmed.ncbi.nlm.nih.gov/38101773/?utm_source=chatgpt.com "Early breast cancer: ESMO Clinical Practice Guideline for ..."
[5]: https://www.nature.com/articles/s41416-024-02827-z?utm_source=chatgpt.com "BRCA-mutated breast cancer: the unmet need, challenges ..."
[6]: https://pmc.ncbi.nlm.nih.gov/articles/PMC10084783/?utm_source=chatgpt.com "Integrating Immunotherapy in Early-Stage Triple-Negative ..."
[7]: https://www.nejm.org/doi/full/10.1056/NEJMoa1910549?utm_source=chatgpt.com "Pembrolizumab for Early Triple-Negative Breast Cancer"
[8]: https://www.annalsofoncology.org/cms/10.1016/j.annonc.2023.11.016/attachment/a1223b0f-a9d6-4097-814e-2e2f89cbe7e1/mmc1.docx?utm_source=chatgpt.com "Early breast cancer: ESMO Clinical Practice Guideline for ..."
[9]: https://education.nccn.org/sites/default/files/course/2024-02/05_Santa-Maria_NCCNbc24_v1.pdf?utm_source=chatgpt.com "Updates to the Management of Triple Negative Breast ..."
[10]: https://pmc.ncbi.nlm.nih.gov/articles/PMC11150442/?utm_source=chatgpt.com "Dose dense doxorubicin plus cyclophosphamide in a ..."
[11]: https://pubmed.ncbi.nlm.nih.gov/35961599/?utm_source=chatgpt.com "Neoadjuvant durvalumab improves survival in early triple- ..."
[12]: https://www.annalsofoncology.org/article/S0923-7534%2822%2903791-7/fulltext?utm_source=chatgpt.com "Neoadjuvant durvalumab improves survival in early triple- ..."
[13]: https://pmc.ncbi.nlm.nih.gov/articles/PMC11094404/?utm_source=chatgpt.com "Expert consensus on the clinical application of immunotherapy in breast cancer: 2024"
[14]: https://education.nccn.org/sites/default/files/course/2023-02/2_Neoadjuvant.Adjuvant%20Treatment_NCCNbc23_v2.pdf?utm_source=chatgpt.com "Neoadjuvant/Adjuvant Treatment for Breast Cancer with SABCS Updates"
[15]: https://www.nejm.org/doi/full/10.1056/NEJMoa2409932?utm_source=chatgpt.com "Overall Survival with Pembrolizumab in Early-Stage Triple ..."
[16]: https://pubmed.ncbi.nlm.nih.gov/35093516/?utm_source=chatgpt.com "Long-term efficacy and safety of addition of carboplatin with ..."
[17]: https://www.mdpi.com/2072-6694/14/17/4064?utm_source=chatgpt.com "Updated Neoadjuvant Treatment Landscape for Early ..."
[18]: https://ascopubs.org/doi/10.1200/JCO.2018.78.0858?utm_source=chatgpt.com "Gonadotropin-Releasing Hormone Agonists

During ..."
[19]: https://www.sciencedirect.com/science/article/pii/S0959804924001667?utm_source=chatgpt.com "Fertility and reproductive concerns related to the new ..."
[20]: https://pmc.ncbi.nlm.nih.gov/articles/PMC8630981/?utm_source=chatgpt.com "Effects of PD-1 blockade on ovarian follicles in a ..."
[21]: https://www.onclive.com/view/adjuvant-olaparib-maintains-idfs-benefit-in-brca1-2-her2-breast-cancer?utm_source=chatgpt.com "Adjuvant Olaparib Maintains iDFS Benefit in BRCA1/2+, ..."
[22]: https://pubmed.ncbi.nlm.nih.gov/36423745/?utm_source=chatgpt.com "Results of the c-TRAK TN trial: a clinical trial utilising ctDNA ..."
[23]: https://eprints.gla.ac.uk/286146/?utm_source=chatgpt.com "Results of the c-TRAK TN trial: a clinical trial utilising ctDNA ..."
[24]: https://www.nature.com/articles/s41523-024-00653-3?utm_source=chatgpt.com "Use of ctDNA in early breast cancer: analytical validity and ..."
[25]: https://documents.cap.org/documents/erpgr_methodology_2020.pdf?utm_source=chatgpt.com "Estrogen and Progesterone Receptor Testing in Breast ..."
