# Supplementary material for: Performance of large language models in ambiguous treatment scenarios of early breast cancer
Source: Breast. 2026 Sep 23;90:104932. doi: 10.1016/j.breast.2026.104932 (PMC13641045; doi:10.1016/j.breast.2026.104932)
Supplement: Supplementary file 1 [file mmc1.zip › Suppl.Mat. - Anton Oseledchyk - Prompts, LLM Responses, R-Code/tnbc_response_claude.pdf]

QUESTION_ID: 25
SELECTED_ANSWER: A
EVIDENCE_SUMMARY: For a 1.1 cm TNBC with 1 positive lymph node (T1bN1a, stage IIA), this does not meet criteria for KEYNOTE-522 (requires T1c or larger). Standard AC/T chemotherapy is appropriate for this lower-risk node-positive disease, without immunotherapy given the small tumor size.
KEY_REFERENCES: KEYNOTE-522 (enrollment criteria T1c/N1-2 or T2-4/N0-2), NCCN Guidelines 2025, St. Gallen consensus
GUIDELINE_ALIGNMENT: NCCN v4.2025 recommends AC/T for T1b node-positive TNBC not meeting KEYNOTE-522 criteria
SUBGROUP_NOTES: Small primary tumor (1.1 cm) with limited nodal burden makes this lower risk than typical KEYNOTE-522 population
CONFIDENCE_LEVEL: High
WEB_SEARCH_USED: Yes
---END---

QUESTION_ID: 26
SELECTED_ANSWER: C
EVIDENCE_SUMMARY: For a 1.1 cm TNBC with 3 positive lymph nodes (T1bN1a, stage IIB), the higher nodal burden increases risk substantially. Though technically T1b is below KEYNOTE-522 criteria (T1c required), the 3 positive nodes create high-risk disease warranting consideration of TCb/AC/pembrolizumab given the poor prognosis.
KEY_REFERENCES: KEYNOTE-522 trial, NCCN Guidelines v4.2025, clinical expert consensus for high-risk exceptions
GUIDELINE_ALIGNMENT: NCCN v4.2025 recommends considering immunotherapy for high-risk TNBC with multiple positive nodes
SUBGROUP_NOTES: Higher nodal burden (3 nodes) creates exceptional risk despite small primary tumor
CONFIDENCE_LEVEL: Moderate
WEB_SEARCH_USED: Yes
---END---

QUESTION_ID: 27
SELECTED_ANSWER: C
EVIDENCE_SUMMARY: KEYNOTE-522 used weekly paclitaxel with every-3-week carboplatin followed by every-3-week AC initially, though dose-dense AC (every 2 weeks) is now commonly used based on established evidence for improved outcomes. The combination of weekly paclitaxel/q3w carboplatin followed by dose-dense AC represents current standard practice.
KEY_REFERENCES: KEYNOTE-522 protocol, CALGB 9741 (dose-dense AC), NCCN Guidelines 2025
GUIDELINE_ALIGNMENT: NCCN v4.2025 supports dose-dense AC scheduling when using growth factor support
SUBGROUP_NOTES: Dose-dense scheduling requires G-CSF support and appropriate patient fitness
CONFIDENCE_LEVEL: High
WEB_SEARCH_USED: Yes
---END---

QUESTION_ID: 28

SELECTED_ANSWER: C
EVIDENCE_SUMMARY: GeparNuevo used durvalumab only in the neoadjuvant setting without adjuvant continuation, unlike KEYNOTE-522 which used pembrolizumab both neoadjuvantly and adjuvantly. Since the patient received durvalumab per GeparNuevo protocol and achieved pCR, no adjuvant checkpoint inhibitor is indicated per that trial design.
KEY_REFERENCES: GeparNuevo trial design, KEYNOTE-522 (different protocol), current practice guidelines
GUIDELINE_ALIGNMENT: Follow specific trial protocol used; GeparNuevo did not include adjuvant immunotherapy
SUBGROUP_NOTES: pCR achievement indicates excellent prognosis regardless of adjuvant immunotherapy
CONFIDENCE_LEVEL: High
WEB_SEARCH_USED: Yes
---END---

QUESTION_ID: 29
SELECTED_ANSWER: A
EVIDENCE_SUMMARY: Per KEYNOTE-522 protocol, pembrolizumab should continue in the adjuvant setting for up to 9 cycles (27 weeks) regardless of pathologic response. CREATE-X data supports capecitabine for residual disease, but combining with pembrolizumab lacks safety data and is not standard practice.
KEY_REFERENCES: KEYNOTE-522 protocol (adjuvant pembrolizumab for 9 cycles), CREATE-X trial, expert consensus on avoiding untested combinations
GUIDELINE_ALIGNMENT: NCCN v4.2025 recommends completing pembrolizumab per KEYNOTE-522; capecitabine consideration remains controversial
SUBGROUP_NOTES: Small volume residual disease (<1 cm, node-negative) has better prognosis than larger residual burden
CONFIDENCE_LEVEL: High
WEB_SEARCH_USED: Yes
---END---

QUESTION_ID: 30
SELECTED_ANSWER: A
EVIDENCE_SUMMARY: For node-positive TNBC with contraindications to immunotherapy, AC/T remains the standard chemotherapy backbone. TCb provides similar efficacy but carboplatin adds toxicity without clear benefit over anthracycline-taxane combination when immunotherapy cannot be used.
KEY_REFERENCES: CALGB 40101, ABC trials, NCCN Guidelines 2025
GUIDELINE_ALIGNMENT: NCCN v4.2025 lists AC/T as preferred chemotherapy for TNBC when immunotherapy contraindicated
SUBGROUP_NOTES: Ensure no cardiac contraindications to anthracyclines; consider TC if anthracycline contraindicated
CONFIDENCE_LEVEL: High
WEB_SEARCH_USED: Yes
---END---

QUESTION_ID: 31
SELECTED_ANSWER: B
EVIDENCE_SUMMARY: Limited data exists on immunotherapy's fertility

impact, with preclinical studies suggesting possible effects on ovarian reserve. However, given the significant survival benefit from KEYNOTE-522 in stage 2 node-positive TNBC, most experts favor treatment with pembrolizumab while acknowledging uncertainty about fertility effects.
KEY_REFERENCES: KEYNOTE-522 survival data, limited fertility studies with checkpoint inhibitors, expert consensus papers 2024
GUIDELINE_ALIGNMENT: No specific guidelines address fertility with immunotherapy; ASCO recommends discussing unknown risks
SUBGROUP_NOTES: Young age requires careful fertility counseling; GnRH agonists and fertility preservation remain important
CONFIDENCE_LEVEL: Moderate
WEB_SEARCH_USED: Yes
---END---

QUESTION_ID: 32
SELECTED_ANSWER: B
EVIDENCE_SUMMARY: For BRCA-mutated TNBC with residual disease after KEYNOTE-522 regimen, both adjuvant pembrolizumab (per protocol) and olaparib (per OlympiA trial) are indicated. While no direct combination data exists, expert consensus supports sequential use given high recurrence risk.
KEY_REFERENCES: KEYNOTE-522 (pembrolizumab continuation), OlympiA trial (olaparib for BRCA+ with residual disease), expert consensus 2024
GUIDELINE_ALIGNMENT: NCCN v4.2025 recommends both agents for eligible patients; sequencing not definitively established
SUBGROUP_NOTES: Capecitabine contraindicated with olaparib due to overlapping toxicity; high-risk population needs aggressive treatment
CONFIDENCE_LEVEL: Moderate
WEB_SEARCH_USED: Yes
---END---

QUESTION_ID: 33
SELECTED_ANSWER: D
EVIDENCE_SUMMARY: ctDNA positivity after surgery indicates molecular residual disease but treatment interventions based solely on ctDNA remain investigational. The c-TRAK TN trial showed high metastatic disease rates with ctDNA detection, but no proven benefit from preemptive treatment in stage 1 disease achieving pCR.
KEY_REFERENCES: c-TRAK TN trial, ZEST trial (terminated), current ctDNA study protocols
GUIDELINE_ALIGNMENT: No current guidelines recommend treatment based solely on ctDNA positivity after pCR
SUBGROUP_NOTES: Stage 1 disease with pCR has excellent prognosis despite ctDNA positivity; close monitoring warranted
CONFIDENCE_LEVEL: High
WEB_SEARCH_USED: Yes
---END---

QUESTION_ID: 41
SELECTED_ANSWER: C
EVIDENCE_SUMMARY: ER-low positive (5%) tumors behave similarly to

TNBC with comparable pCR rates and outcomes when treated with chemotherapy. Current evidence and guidelines increasingly support treating ER 1-9% tumors as TNBC, particularly with grade 3 features, making pembrolizumab with chemotherapy appropriate.
KEY_REFERENCES: Swedish population study 2024, KEYNOTE-522 subgroup analyses, emerging ER-low consensus
GUIDELINE_ALIGNMENT: Evolving guidelines recognize ER-low (1-9%) as functionally triple-negative; NCCN considering updates
SUBGROUP_NOTES: Grade 3 features and very low ER (5%) support TNBC-like treatment approach
CONFIDENCE_LEVEL: Moderate
WEB_SEARCH_USED: Yes
---END---
