# Supplementary material for: Performance of large language models in ambiguous treatment scenarios of early breast cancer
Source: Breast. 2026 Sep 23;90:104932. doi: 10.1016/j.breast.2026.104932 (PMC13641045; doi:10.1016/j.breast.2026.104932)
Supplement: Supplementary file 1 [file mmc1.zip › Suppl.Mat. - Anton Oseledchyk - Prompts, LLM Responses, R-Code/tnbc_response_deepseek.pdf]

QUESTION_ID: 25
SELECTED_ANSWER: C
EVIDENCE_SUMMARY: For node-positive TNBC (even with 1 positive node), the KEYNOTE-522 regimen (pembrolizumab with neoadjuvant/ adjuvant chemotherapy) is standard based on improved event-free survival (EFS) and pathological complete response (pCR) rates. Recent NCCN Guidelines (v3.2025) continue to recommend this regimen for stage II-III TNBC, which includes node-positive disease. The benefit is clear even for smaller tumors when nodes are involved.
KEY_REFERENCES: KEYNOTE-522 trial; NCCN Guidelines Breast Cancer v3.2025
GUIDELINE_ALIGNMENT: NCCN (2025) and ESMO guidelines recommend pembrolizumab with chemotherapy (TCb/AC) for stage II-III TNBC.
SUBGROUP_NOTES: This patient has node-positive disease (1/3 nodes), which places her in stage II and makes her eligible for immunotherapy-based regimen.
CONFIDENCE_LEVEL: High
WEB_SEARCH_USED: Yes - Searched for NCCN Guidelines v3.2025 which continue to recommend pembrolizumab with chemotherapy for node-positive TNBC.

---END---

QUESTION_ID: 26
SELECTED_ANSWER: C
EVIDENCE_SUMMARY: For TNBC with 3 positive lymph nodes (stage II-III), the KEYNOTE-522 regimen with pembrolizumab plus chemotherapy is strongly indicated based on significant EFS benefit. NCCN Guidelines (v3.2025) maintain this recommendation for node-positive TNBC regardless of the number of positive nodes (within stage II-III). The higher nodal burden further supports the need for aggressive therapy.
KEY_REFERENCES: KEYNOTE-522 trial; NCCN Guidelines Breast Cancer v3.2025
GUIDELINE_ALIGNMENT: NCCN (2025) and ESMO guidelines recommend TCb/ AC/pembrolizumab for stage II-III TNBC.
SUBGROUP_NOTES: Higher nodal burden (3/5 nodes) increases recurrence risk, making immunotherapy even more crucial.
CONFIDENCE_LEVEL: High
WEB_SEARCH_USED: Yes - Confirmed NCCN v3.2025 recommends pembrolizumab-based regimen for all stage II-III TNBC, including those with multiple positive nodes.

---END---

QUESTION_ID: 27
SELECTED_ANSWER: A
EVIDENCE_SUMMARY: The KEYNOTE-522 trial protocol used weekly paclitaxel (80 mg/m^2) with every-3-week carboplatin (AUC 5), followed by every-3-week AC (doxorubicin/cyclophosphamide). This schedule is evidence-based and remains the standard in NCCN Guidelines (v3.2025). Alternative schedules (e.g., weekly carboplatin or dose-dense AC) are not supported by the trial data.
KEY_REFERENCES: KEYNOTE-522 trial; NCCN Guidelines Breast Cancer

v3.2025
GUIDELINE_ALIGNMENT: NCCN (2025) guidelines specify the KEYNOTE-522 chemotherapy schedule for TNBC treated with pembrolizumab.
SUBGROUP_NOTES: The every-3-week carboplatin and AC schedule balances efficacy with manageable toxicity.
CONFIDENCE_LEVEL: High
WEB_SEARCH_USED: No - Based on the established KEYNOTE-522 protocol; no new evidence through Feb 2025 changes this.

---END---

QUESTION_ID: 28
SELECTED_ANSWER: C
EVIDENCE_SUMMARY: For patients who achieve pCR after neoadjuvant chemotherapy (with or without checkpoint inhibitor), no further adjuvant checkpoint inhibition is recommended. The GeparNuevo trial did not show a significant benefit for continued adjuvant durvalumab after pCR. NCCN Guidelines (v3.2025) do not recommend adjuvant checkpoint inhibitor beyond the neoadjuvant/adjuvant course if pCR is achieved.
KEY_REFERENCES: GeparNuevo trial; KEYNOTE-522 trial; NCCN Guidelines Breast Cancer v3.2025
GUIDELINE_ALIGNMENT: NCCN (2025) guidelines do not recommend adjuvant checkpoint inhibitor after pCR.
SUBGROUP_NOTES: pCR indicates excellent prognosis; additional immunotherapy may add toxicity without proven benefit.
CONFIDENCE_LEVEL: High
WEB_SEARCH_USED: No - Based on established trial data; no new evidence through Feb 2025 supports adjuvant checkpoint inhibitor after pCR.

---END---

QUESTION_ID: 29
SELECTED_ANSWER: A
EVIDENCE_SUMMARY: For residual disease after neoadjuvant KEYNOTE-522 regimen, adjuvant pembrolizumab should be completed (total of 9 cycles, including neoadjuvant doses). The CREATE-X trial supports capecitabine for residual disease, but its role after immunotherapy is not defined. NCCN Guidelines (v3.2025) recommend continuing adjuvant pembrolizumab for these patients.
KEY_REFERENCES: KEYNOTE-522 trial; CREATE-X trial; NCCN Guidelines Breast Cancer v3.2025
GUIDELINE_ALIGNMENT: NCCN (2025) guidelines recommend completing adjuvant pembrolizumab for patients who started it neoadjuvantly, regardless of response.
SUBGROUP_NOTES: The small residual burden (<1 cm, node-negative) may have better prognosis, but completing immunotherapy is standard.
CONFIDENCE_LEVEL: High
WEB_SEARCH_USED: Yes - Verified NCCN v3.2025 maintains recommendation to complete adjuvant pembrolizumab after neoadjuvant KEYNOTE-522.

---END---

QUESTION_ID: 30
SELECTED_ANSWER: A
EVIDENCE_SUMMARY: For patients with contraindications to checkpoint inhibitors, anthracycline-based regimens like AC/T (dose-dense doxorubicin/cyclophosphamide followed by paclitaxel) are preferred. TCb/AC is specifically designed for use with pembrolizumab in KEYNOTE-522. NCCN Guidelines (v3.2025) list AC/T as a standard option for TNBC when immunotherapy is not used.
KEY_REFERENCES: ECOG 1199; NCCN Guidelines Breast Cancer v3.2025
GUIDELINE_ALIGNMENT: NCCN (2025) guidelines recommend AC/T or similar anthracycline/taxane regimens for TNBC when immunotherapy is contraindicated.
SUBGROUP_NOTES: Dose-dense AC/T has shown survival benefits in earlier trials and remains a backbone regimen.
CONFIDENCE_LEVEL: High
WEB_SEARCH_USED: Yes - Confirmed NCCN v3.2025 lists AC/T as a standard chemotherapy option for TNBC without immunotherapy.

---END---

QUESTION_ID: 31
SELECTED_ANSWER: B
EVIDENCE_SUMMARY: The impact of immunotherapy (pembrolizumab) on future fertility is not fully known, but it is not contraindicated in patients desiring fertility. Given the significant survival benefit in TNBC, treatment with pembrolizumab is favored. NCCN Guidelines (v3.2025) recommend fertility preservation and GnRH agonists during chemotherapy but do not restrict immunotherapy due to fertility concerns.
KEY_REFERENCES: KEYNOTE-522 trial; NCCN Guidelines Breast Cancer v3.2025
GUIDELINE_ALIGNMENT: NCCN (2025) guidelines support fertility preservation measures and recommend standard oncologic care including immunotherapy for eligible patients.
SUBGROUP_NOTES: Young age and fertility concerns require multidisciplinary approach, but should not preclude effective therapy.
CONFIDENCE_LEVEL: Moderate
WEB_SEARCH_USED: Yes - Searched for fertility and immunotherapy guidelines; NCCN v3.2025 does not list immunotherapy as a contraindication for fertility concerns.

---END---

QUESTION_ID: 32
SELECTED_ANSWER: B
EVIDENCE_SUMMARY: For BRCA-mutant TNBC with residual disease after neoadjuvant chemotherapy, adjuvant olaparib is recommended based on the OlympiA trial, which showed invasive disease-free survival benefit. adjuvant pembrolizumab is also indicated if not completed during neoadjuvant therapy (per KEYNOTE-522). NCCN Guidelines (v3.2025) recommend both agents sequentially for eligible patients.
KEY_REFERENCES: OlympiA trial; KEYNOTE-522 trial; NCCN Guidelines

Breast Cancer v3.2025
GUIDELINE_ALIGNMENT: NCCN (2025) guidelines recommend adjuvant olaparib for BRCA-mutant patients with residual disease after neoadjuvant chemotherapy, and pembrolizumab should be completed if started.
SUBGROUP_NOTES: BRCA mutation adds rationale for PARP inhibitor therapy. The combination of pembrolizumab and olaparib is not directly studied but can be sequenced.
CONFIDENCE_LEVEL: High
WEB_SEARCH_USED: Yes - Confirmed NCCN v3.2025 recommends adjuvant olaparib for BRCA-mutant patients with residual disease after neoadjuvant chemo.

---END---

QUESTION_ID: 33
SELECTED_ANSWER: D
EVIDENCE_SUMMARY: The role of circulating tumor DNA (ctDNA) detection after pCR is not yet standard for guiding therapy. Trials like c-TRAK-TN are ongoing, but current guidelines (NCCN v3.2025) do not recommend additional therapy based solely on positive ctDNA after pCR in the absence of radiographic evidence of disease. The excellent prognosis of pCR does not warrant deviation from standard care.
KEY_REFERENCES: c-TRAK-TN trial (ongoing); NCCN Guidelines Breast Cancer v3.2025
GUIDELINE_ALIGNMENT: NCCN (2025) guidelines do not recommend additional therapy for pCR patients based on ctDNA positivity alone.
SUBGROUP_NOTES: ctDNA positivity may indicate molecular residual disease, but its clinical utility is not established; enrollment in clinical trials is preferred.
CONFIDENCE_LEVEL: Moderate
WEB_SEARCH_USED: Yes - Searched for ctDNA guidelines; NCCN v3.2025 does not endorse treatment based solely on ctDNA after pCR.

---END---

QUESTION_ID: 41
SELECTED_ANSWER: C
EVIDENCE_SUMMARY: For ER low-positive (1-10%) tumors, NCCN Guidelines (v3.2025) recommend managing them as TNBC if they exhibit clinical behavior similar to TNBC (e.g., high grade, low ER expression). For this 3-4 cm, grade 3, node-negative tumor, neoadjuvant pembrolizumab with chemotherapy (per KEYNOTE-522) is appropriate. The regimen should include a taxane followed by AC, concurrent with pembrolizumab.
KEY_REFERENCES: KEYNOTE-522 trial; NCCN Guidelines Breast Cancer v3.2025
GUIDELINE_ALIGNMENT: NCCN (2025) guidelines state that ER low-positive tumors may be treated as TNBC if clinically appropriate, and recommend pembrolizumab-based therapy for stage II-III TNBC.
SUBGROUP_NOTES: ER low-positive tumors may have response patterns similar to TNBC when ER is <10%; neoadjuvant therapy allows response assessment.

CONFIDENCE_LEVEL: High
WEB_SEARCH_USED: Yes – Confirmed NCCN v3.2025 recommends considering ER low-positive (1–10%) tumors as TNBC for treatment decisions if clinically appropriate.

---END---
