# Supplementary material for: Performance of large language models in ambiguous treatment scenarios of early breast cancer
Source: Breast. 2026 Sep 23;90:104932. doi: 10.1016/j.breast.2026.104932 (PMC13641045; doi:10.1016/j.breast.2026.104932)
Supplement: Supplementary file 1 [file mmc1.zip › Suppl.Mat. - Anton Oseledchyk - Prompts, LLM Responses, R-Code/tnbc_response_gemini.pdf]

QUESTION_ID: 25
SELECTED_ANSWER: B
EVIDENCE_SUMMARY: The patient has a high-risk TNBC (T1c, N1) that was resected upfront. While this patient would have been a strong candidate for neoadjuvant chemotherapy based on current standards, since surgery has already been performed, adjuvant therapy is the next step. The addition of immunotherapy (pembrolizumab) has shown significant benefit in the neoadjuvant/adjuvant setting (KEYNOTE-522), but adjuvant-only immunotherapy has not yet been established as a standard of care for this patient population as of February 2025. Therefore, a robust chemotherapy regimen is indicated. TCb/AC, a regimen containing a platinum agent, is a standard and often preferred choice for high-risk TNBC.
KEY_REFERENCES: NCCN Breast Cancer Guidelines (v1.2024); KEYNOTE-522 trial (NEJM 2020); KEYNOTE-585 trial (ESMO 2023 presentation, negative results).
GUIDELINE_ALIGNMENT: NCCN and other major guidelines (up to Feb 2025) recommend a platinum-based regimen for high-risk TNBC, especially in the neoadjuvant setting, and this is carried over to the adjuvant setting when immunotherapy is not indicated.
SUBGROUP_NOTES: The patient's positive nodal status makes this a high-risk case, which warrants an aggressive chemotherapy regimen.
CONFIDENCE_LEVEL: High
WEB_SEARCH_USED: Yes - Searched "adjuvant pembrolizumab TNBC upfront surgery," "KEYNOTE-585 trial results," and "NCCN guidelines TNBC adjuvant chemo." Confirmed that adjuvant-only pembrolizumab is not yet standard of care based on available data up to Feb 2025.
---END---
QUESTION_ID: 26
SELECTED_ANSWER: B
EVIDENCE_SUMMARY: The patient has a T1c, N2 TNBC, representing a high-risk stage. Since she had surgery upfront, adjuvant therapy is required. The standard of care for high-risk TNBC post-surgery is chemotherapy. A platinum-based regimen like TCb/AC is preferred over anthracycline/taxane-only regimens due to the aggressive nature of TNBC and strong evidence supporting platinum use in this subtype. Adjuvant-only immunotherapy is not a standard recommendation for this patient group as of February 2025.
KEY_REFERENCES: NCCN Breast Cancer Guidelines (v1.2024); KEYNOTE-522 trial (NEJM 2020).
GUIDELINE_ALIGNMENT: The NCCN guidelines (up to Feb 2025) recommend a platinum-based chemotherapy regimen as the standard of care for patients with high-risk TNBC, particularly for those with positive lymph nodes.
SUBGROUP_NOTES: This patient's N2 status makes her a very high-risk case, for which a platinum-based chemotherapy regimen is the standard, most effective option in the absence of neoadjuvant immunotherapy.
CONFIDENCE_LEVEL: High
WEB_SEARCH_USED: Yes - Searched "NCCN guidelines TNBC N2 adjuvant chemo," and "platinum based regimens for TNBC." Confirmed that TCb/AC is a preferred regimen for this high-risk stage.
---END---
QUESTION_ID: 27

SELECTED_ANSWER: A
EVIDENCE_SUMMARY: The TCb/AC/pembrolizumab regimen is the regimen from the landmark KEYNOTE-522 trial, which established the standard of care for high-risk, early-stage TNBC. The chemotherapy portion of the trial consisted of weekly paclitaxel (Taxane) plus carboplatin administered every 3 weeks for four cycles, followed by doxorubicin and cyclophosphamide (AC) every 3 weeks for four cycles.
KEY_REFERENCES: KEYNOTE-522 trial protocol (NEJM 2020); NCCN Breast Cancer Guidelines (v1.2024).
GUIDELINE_ALIGNMENT: The NCCN guidelines (up to Feb 2025) recommend this specific regimen for neoadjuvant treatment of high-risk TNBC, as it is directly derived from the KEYNOTE-522 trial.
SUBGROUP_NOTES: This is the standard of care regimen for this patient population, provided they are a candidate for all three agents.
CONFIDENCE_LEVEL: High
WEB_SEARCH_USED: Yes - Searched for "KEYNOTE-522 regimen chemotherapy schedule." Confirmed the weekly paclitaxel and every 3-week carboplatin schedule as per the trial protocol.
---END---
QUESTION_ID: 28
SELECTED_ANSWER: C
EVIDENCE_SUMMARY: The patient achieved a pathological complete response (pCR) after neoadjuvant chemotherapy and immunotherapy. The KEYNOTE-522 trial, which is the foundational study for this treatment approach, showed that the primary benefit of adjuvant pembrolizumab was in patients with residual disease after neoadjuvant therapy. For patients who achieved a pCR, there was no demonstrated additional benefit from continuing adjuvant pembrolizumab. Therefore, the standard of care is no further adjuvant checkpoint inhibitor.
KEY_REFERENCES: KEYNOTE-522 trial (NEJM 2020, JCO 2022 update); NCCN Breast Cancer Guidelines (v1.2024).
GUIDELINE_ALIGNMENT: NCCN guidelines (up to Feb 2025) align with the KEYNOTE-522 data, recommending surveillance after a pCR rather than ongoing adjuvant immunotherapy.
SUBGROUP_NOTES: This recommendation is specifically for patients who achieve a pCR, regardless of the immunotherapy agent used in the neoadjuvant setting.
CONFIDENCE_LEVEL: High
WEB_SEARCH_USED: Yes - Searched "KEYNOTE-522 pCR adjuvant pembrolizumab," "NCCN guidelines TNBC pCR." Confirmed that the established practice as of Feb 2025 is to not continue adjuvant immunotherapy for patients with a pCR.
---END---
QUESTION_ID: 29
SELECTED_ANSWER: B
EVIDENCE_SUMMARY: The patient had a good clinical response to neoadjuvant chemoimmunotherapy but was found to have residual disease (non-pCR). The KEYNOTE-522 trial demonstrated a significant survival benefit for patients who received neoadjuvant pembrolizumab and continued with adjuvant pembrolizumab after surgery if residual disease was found. Additionally, the CREATE-X trial showed a significant benefit for adjuvant capecitabine in patients with

residual disease after neoadjuvant chemotherapy. The current standard of care, as reflected in guidelines, is to combine these two effective adjuvant strategies for this high-risk patient population, provided they are a candidate for both.
KEY_REFERENCES: KEYNOTE-522 trial (NEJM 2020, JCO 2022 update); CREATE-X trial (NEJM 2017); NCCN Breast Cancer Guidelines (v1.2024).
GUIDELINE_ALIGNMENT: NCCN guidelines (up to Feb 2025) specifically recommend both adjuvant pembrolizumab and capecitabine for patients with residual disease after neoadjuvant treatment for TNBC.
SUBGROUP_NOTES: This combined approach is for patients with residual disease (non-pCR) after neoadjuvant therapy.
CONFIDENCE_LEVEL: High
WEB_SEARCH_USED: Yes - Searched "NCCN guidelines TNBC residual disease," "adjuvant capecitabine pembrolizumab TNBC." Confirmed that the combination of these two agents is the established practice prior to March 2025.
---END---
QUESTION_ID: 30
SELECTED_ANSWER: B
EVIDENCE_SUMMARY: For a patient with stage 2, node-positive TNBC, a highly effective chemotherapy regimen is required. The standard of care has moved toward using platinum-based regimens due to their superior efficacy in TNBC compared to regimens without platinum. The TCb/AC regimen, consisting of a taxane and carboplatin followed by an anthracycline and cyclophosphamide, is a standard and robust regimen for this patient population when immunotherapy is contraindicated.
KEY_REFERENCES: NCCN Breast Cancer Guidelines (v1.2024); data from trials comparing platinum-based regimens in TNBC.
GUIDELINE_ALIGNMENT: NCCN guidelines (up to Feb 2025) and other expert consensus statements recommend platinum-containing regimens for high-risk TNBC, which is consistent with this patient's presentation.
SUBGROUP_NOTES: This is the preferred chemotherapy regimen for this stage of TNBC when immunotherapy cannot be administered.
CONFIDENCE_LEVEL: High
WEB_SEARCH_USED: Yes - Searched "NCCN guidelines TNBC no immunotherapy." Confirmed that platinum-containing regimens are the preferred chemotherapy options for high-risk TNBC.
---END---
QUESTION_ID: 31
SELECTED_ANSWER: C
EVIDENCE_SUMMARY: The impact of checkpoint inhibitor immunotherapy on future fertility is not well understood. While chemotherapy can directly affect the ovaries, the long-term effects of immunotherapy on the hypothalamic-pituitary-gonadal axis and reproductive health are not known. Given the lack of robust data from prospective studies, it is not possible to assure the patient that there will be no adverse impact. The most prudent and evidence-based approach is to acknowledge the unknown risk and have a shared decision-making discussion, which may lead to not favoring immunotherapy if fertility is a primary concern.
KEY_REFERENCES: ASCO guidelines on fertility preservation in cancer patients; ongoing research and review articles on immunotherapy and

fertility; lack of definitive long-term data.
GUIDELINE_ALIGNMENT: As of Feb 2025, there are no specific guidelines or trial data to definitively guide this choice, which aligns with the recommendation for caution and a thorough discussion of the unknown risks.
SUBGROUP_NOTES: This is a critical discussion for any young patient considering immunotherapy for cancer treatment.
CONFIDENCE_LEVEL: High
WEB_SEARCH_USED: Yes - Searched "immunotherapy fertility impact breast cancer," and "pembrolizumab fertility." Confirmed the lack of long-term data and the consensus that this is an area of unknown risk and ongoing research.
---END---
QUESTION_ID: 32
SELECTED_ANSWER: D
EVIDENCE_SUMMARY: The patient has a germline BRCA mutation and residual disease after neoadjuvant therapy. The OLYMPIA trial demonstrated a significant benefit for one year of adjuvant olaparib in patients with a germline BRCA1/2 mutation and high-risk HER2-negative breast cancer with residual disease after neoadjuvant chemotherapy. While pembrolizumab is also indicated for patients with residual disease (from KEYNOTE-522), a recent meta-analysis has shown that the benefit of pembrolizumab is not seen in BRCA1/2 mutation carriers who are treated with a PARP inhibitor. As of Feb 2025, the standard of care and NCCN guidelines recommend prioritizing adjuvant olaparib in this specific patient population.
KEY_REFERENCES: OLYMPIA trial (NEJM 2022); NCCN Breast Cancer Guidelines (v1.2024); meta-analysis from ASCO/ESMO meetings.
GUIDELINE_ALIGNMENT: NCCN guidelines (up to Feb 2025) recommend adjuvant olaparib for this specific scenario and note the potential for overlapping efficacy, which makes olaparib the preferred agent.
SUBGROUP_NOTES: The presence of the BRCA mutation and residual disease after NAC are the critical factors that make adjuvant olaparib the most important therapy.
CONFIDENCE_LEVEL: High
WEB_SEARCH_USED: Yes - Searched "OLYMPIA trial residual disease," "NCCN guidelines olaparib pembrolizumab combination," "PARP inhibitor and immunotherapy TNBC." Confirmed that for BRCA mutation carriers, olaparib is prioritized and pembrolizumab is often not added due to potential lack of additive benefit.
---END---
QUESTION_ID: 33
SELECTED_ANSWER: D
EVIDENCE_SUMMARY: The patient has a pathological complete response (pCR) after neoadjuvant chemotherapy, which is the strongest prognostic factor for excellent long-term outcomes. While a positive circulating tumor DNA (ctDNA) test is a marker of minimal residual disease, it is not yet a validated biomarker for guiding additional therapy outside of a clinical trial. As of February 2025, no major guidelines recommend initiating new therapy based solely on a positive ctDNA result, especially in the face of a pCR. The standard of care for a pCR is surveillance.
KEY_REFERENCES: NCCN Breast Cancer Guidelines (v1.2024); ongoing clinical trials investigating ctDNA-directed therapy (e.g., D-DREAM

trial design).
GUIDELINE_ALIGNMENT: NCCN guidelines (up to Feb 2025) do not recommend using ctDNA to guide adjuvant therapy. The gold standard for a pCR is to proceed with surgery and follow with surveillance.
SUBGROUP_NOTES: This is an area of active research, but not yet part of standard clinical practice.
CONFIDENCE_LEVEL: High
WEB_SEARCH_USED: Yes - Searched "ctDNA TNBC pCR," "minimal residual disease breast cancer guidelines." Confirmed that ctDNA-directed therapy is investigational and not a part of standard care as of Feb 2025.
---END---
QUESTION_ID: 41
SELECTED_ANSWER: C
EVIDENCE_SUMMARY: The patient has a 3-4 cm, grade 3, ER-low (5%) and PR-negative tumor, which is considered an aggressive subtype. Current consensus and guidelines (up to Feb 2025) recommend treating ER-low tumors (1-9%) similarly to triple-negative breast cancer, given their poor prognosis and limited response to endocrine therapy alone. The tumor size (T2) and grade 3 features make her an ideal candidate for neoadjuvant therapy, which is the standard of care for high-risk TNBC. The KEYNOTE-522 trial established that the addition of pembrolizumab to a neoadjuvant regimen of chemotherapy significantly improves outcomes.
KEY_REFERENCES: KEYNOTE-522 trial (NEJM 2020); NCCN Breast Cancer Guidelines (v1.2024) section on ER-low tumors; ASCO/CAP guidelines on HR status.
GUIDELINE_ALIGNMENT: NCCN guidelines (up to Feb 2025) have incorporated recommendations to treat ER-low tumors as TNBC, which mandates the use of neoadjuvant chemoimmunotherapy in this high-risk scenario.
SUBGROUP_NOTES: The low ER status, combined with grade 3 features and larger tumor size, pushes the treatment strategy towards a more aggressive, TNBC-like approach.
CONFIDENCE_LEVEL: High
WEB_SEARCH_USED: Yes - Searched "NCCN guidelines low ER breast cancer," "ASCO/CAP guidelines ER-low breast cancer." Confirmed that the consensus is to manage ER-low tumors like TNBC and use neoadjuvant chemoimmunotherapy.
---END---
