# Supplementary material for: Performance of large language models in ambiguous treatment scenarios of early breast cancer
Source: Breast. 2026 Sep 23;90:104932. doi: 10.1016/j.breast.2026.104932 (PMC13641045; doi:10.1016/j.breast.2026.104932)
Supplement: Supplementary file 2 [file mmc2.zip › ybrst_104932_Suppl.Mat. - Anton Oseledchyk - Interactive Table_mmc2.html]

St. Gallen AI Consensus - Interactive Table


# St. Gallen AI Consensus

Interactive Table with 81 Expert Questions and AI Analysis

Topic:

All Topics
Axillary
Radiation
TNBC
HER2+
HR+
Follow-up

AI Consensus:

All Levels
1 of 4
2 of 4
3 of 4
4 of 4

⭐
Claude

■
ChatGPT

▲
Gemini

●
DeepSeek

**81** / 81 questions

| ID / Slide | Expert Consensus strong? | AI Consensus | Topic | Question text | A | B | C | D | E | F | G |
| --- | --- | --- | --- | --- | --- | --- | --- | --- | --- | --- | --- |
| Loading data... | | | | | | | | | | | |

×
